# Supplementary figures and images for: miR-605-3p may affect caerulein-induced ductal cell injury and pyroptosis in acute pancreatitis by targeting the DUOX2/NLRP3/NF-κB pathway (part 1 of 3)
Source: PeerJ. 2024 Aug 30;12:e17874. doi: 10.7717/peerj.17874 (PMC11368084; doi:10.7717/peerj.17874)

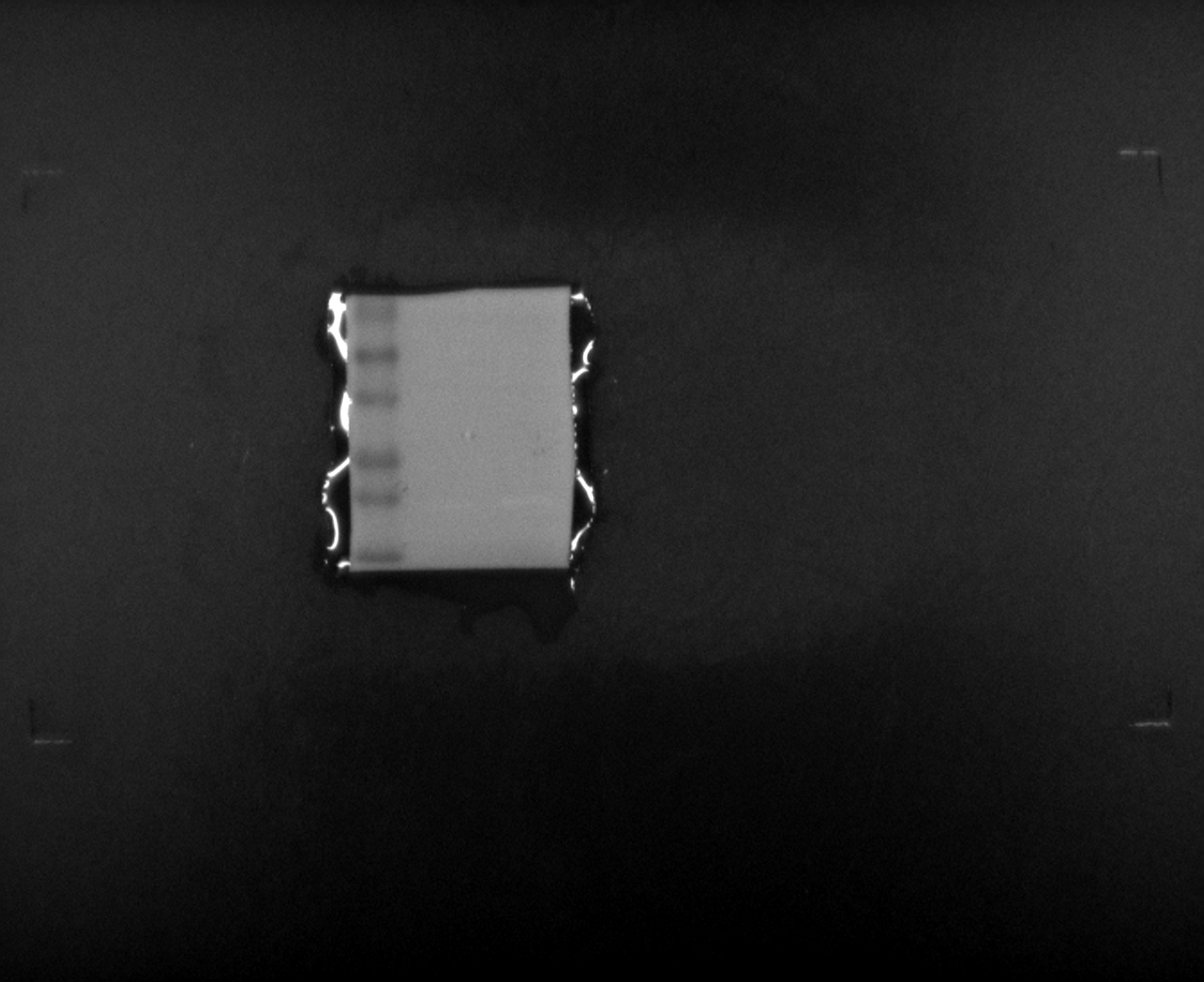

Supplement: Supplemental Information 2 [file peerj-12-17874-s002.zip › fig 1G/BAX (1).tif]

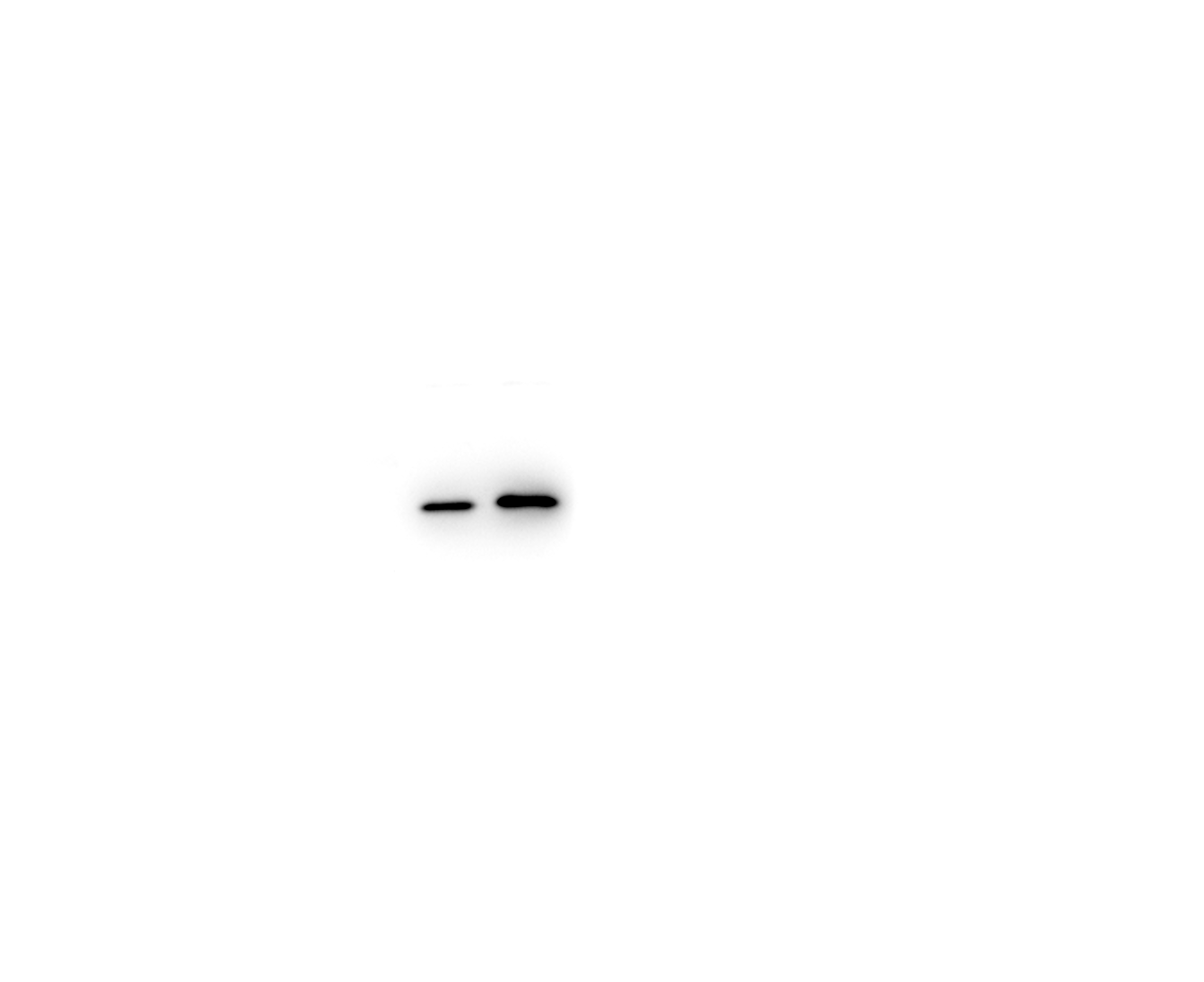

Supplement: Supplemental Information 2 [file peerj-12-17874-s002.zip › fig 1G/BAX (2).tif]

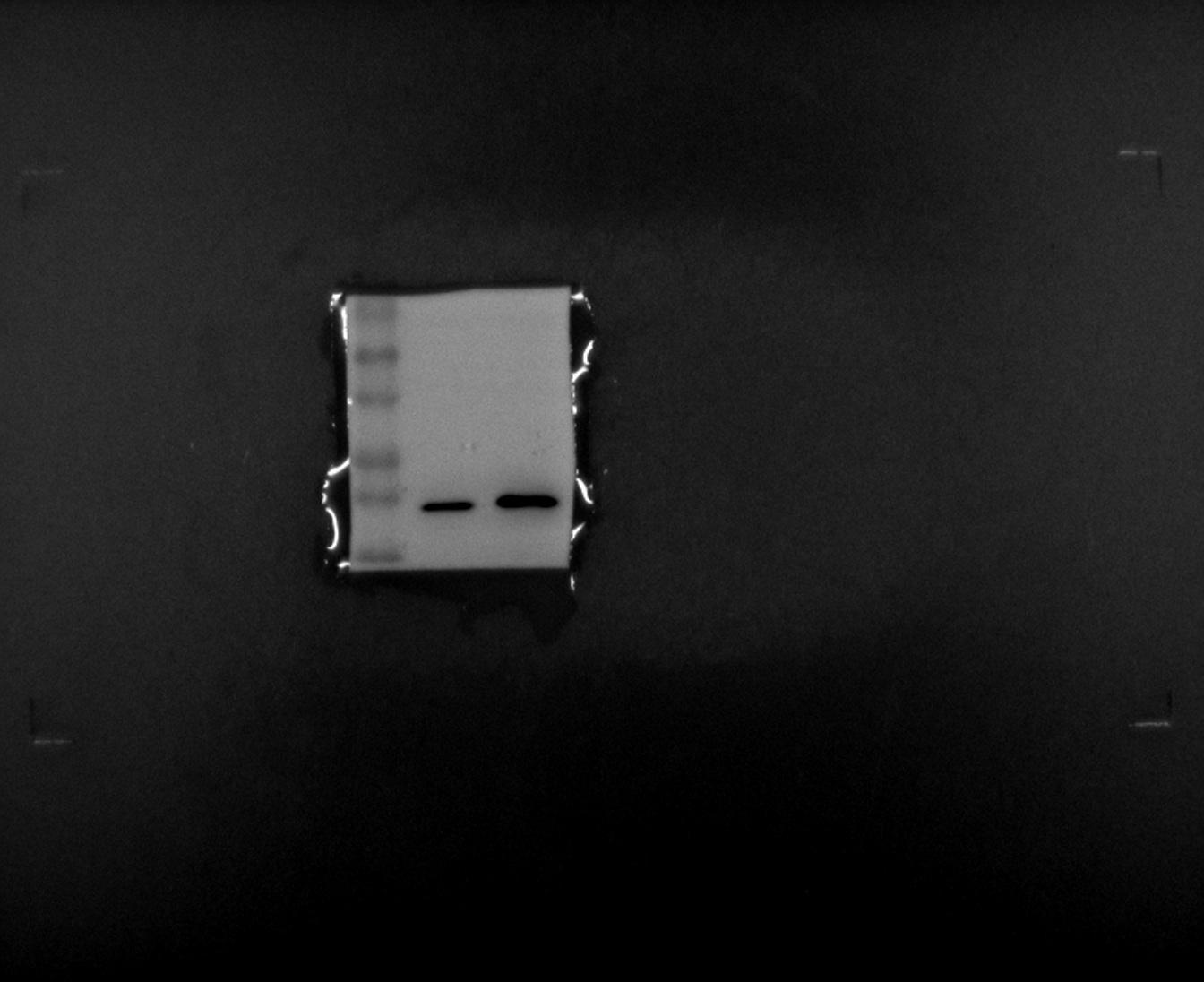

Supplement: Supplemental Information 2 [file peerj-12-17874-s002.zip › fig 1G/BAX (3).tif]

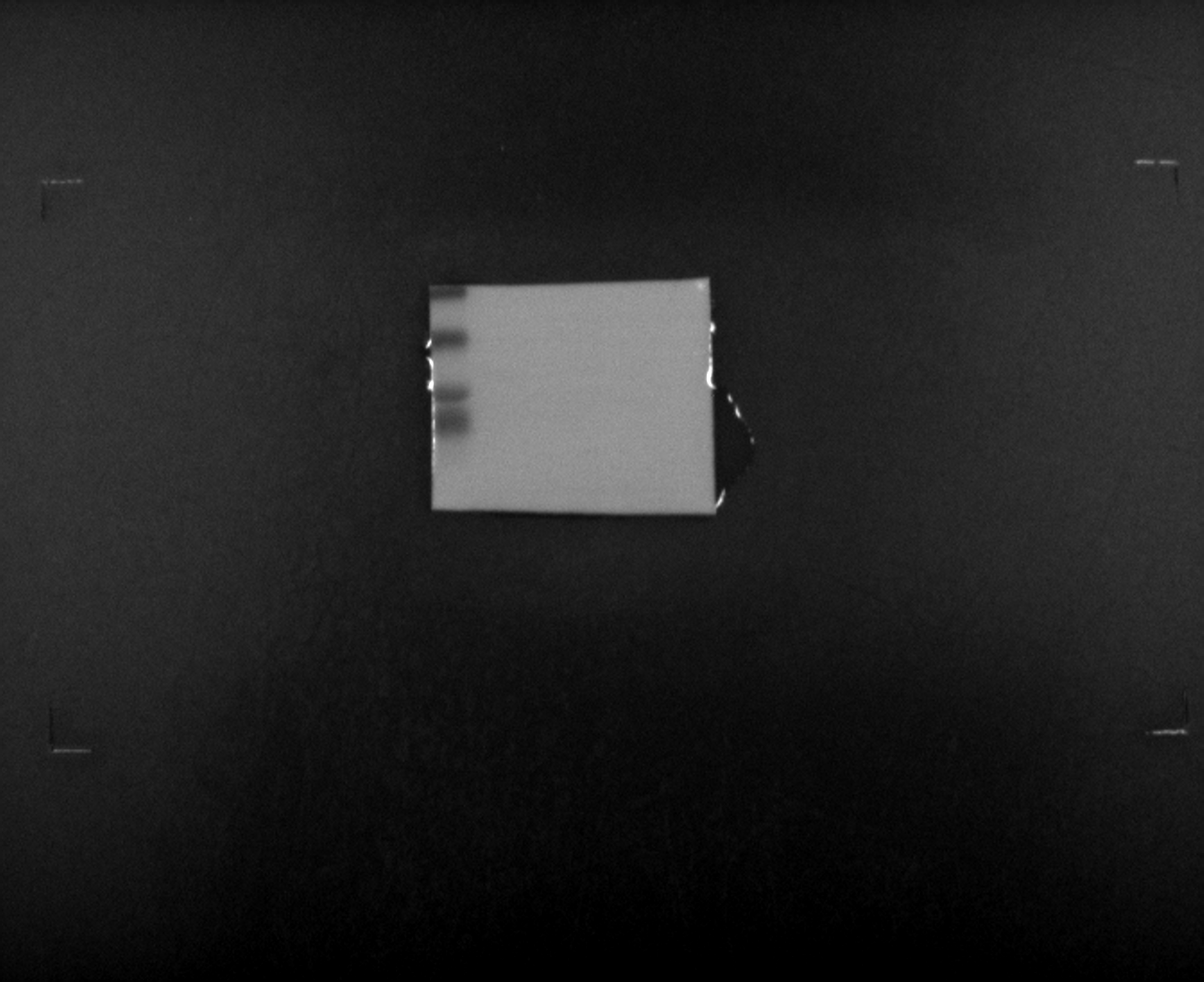

Supplement: Supplemental Information 2 [file peerj-12-17874-s002.zip › fig 1G/bax-2 (1).tif]

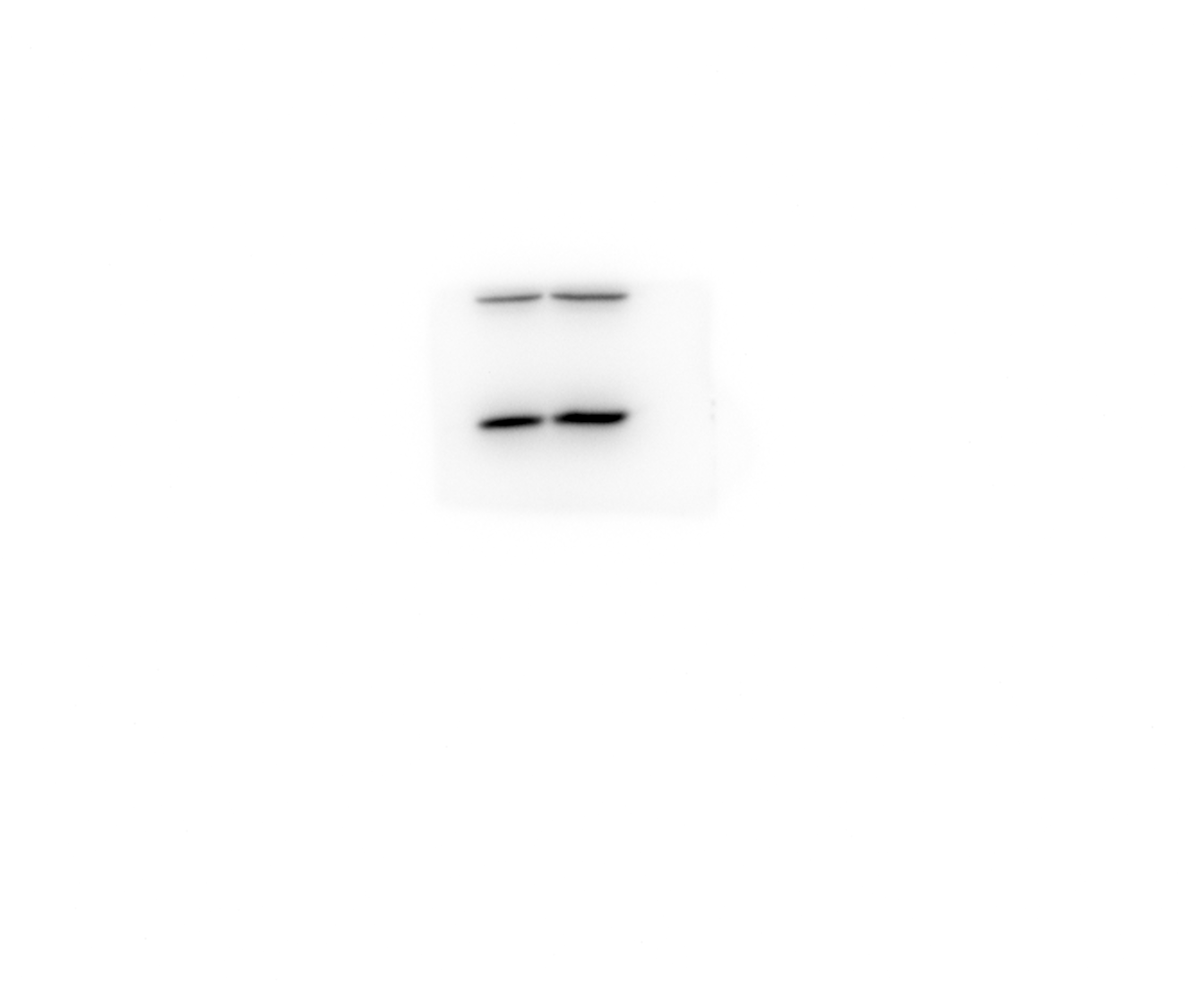

Supplement: Supplemental Information 2 [file peerj-12-17874-s002.zip › fig 1G/bax-2 (2).tif]

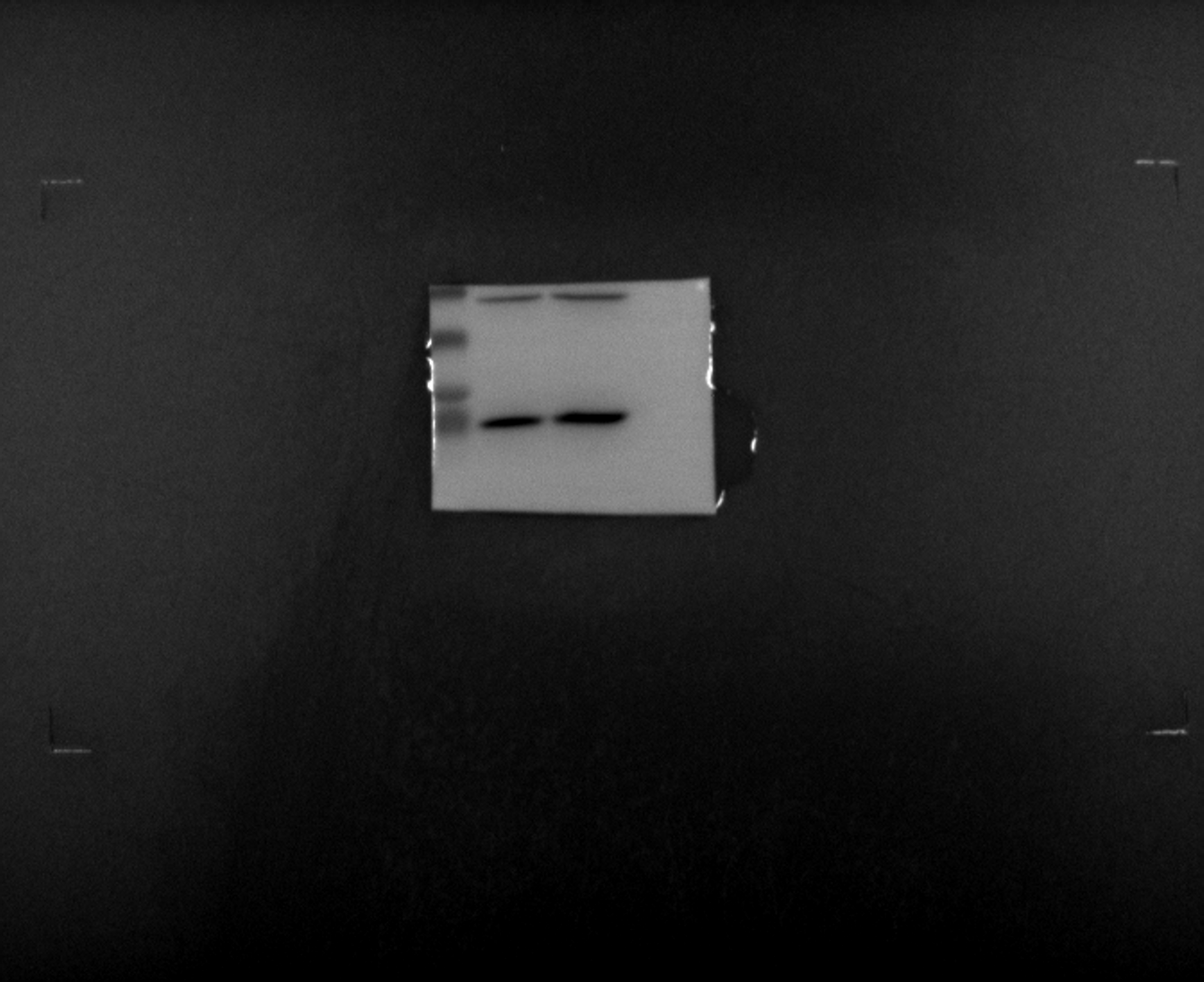

Supplement: Supplemental Information 2 [file peerj-12-17874-s002.zip › fig 1G/bax-2 (3).tif]

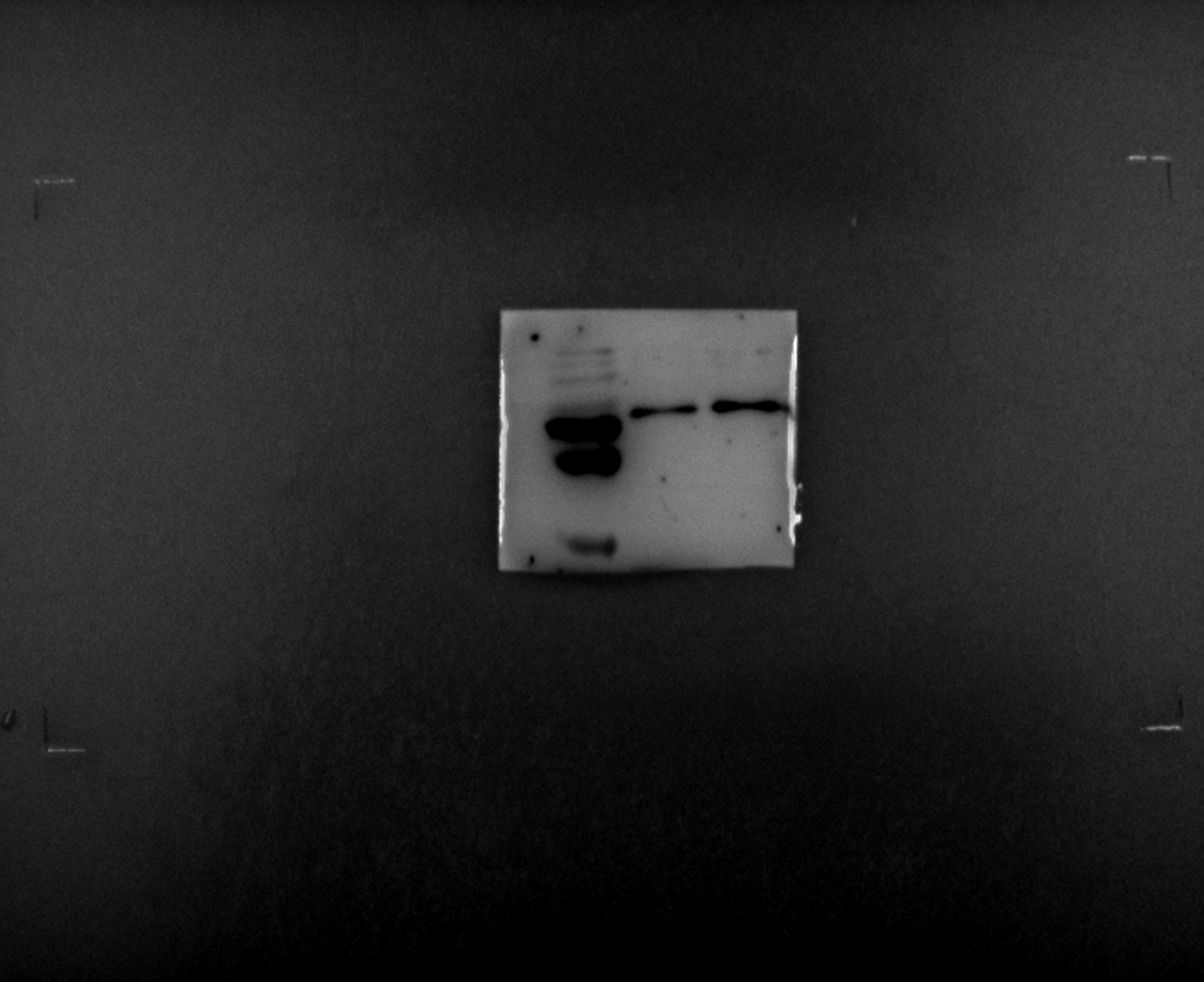

Supplement: Supplemental Information 2 [file peerj-12-17874-s002.zip › fig 1G/bax-3 (1).tif]

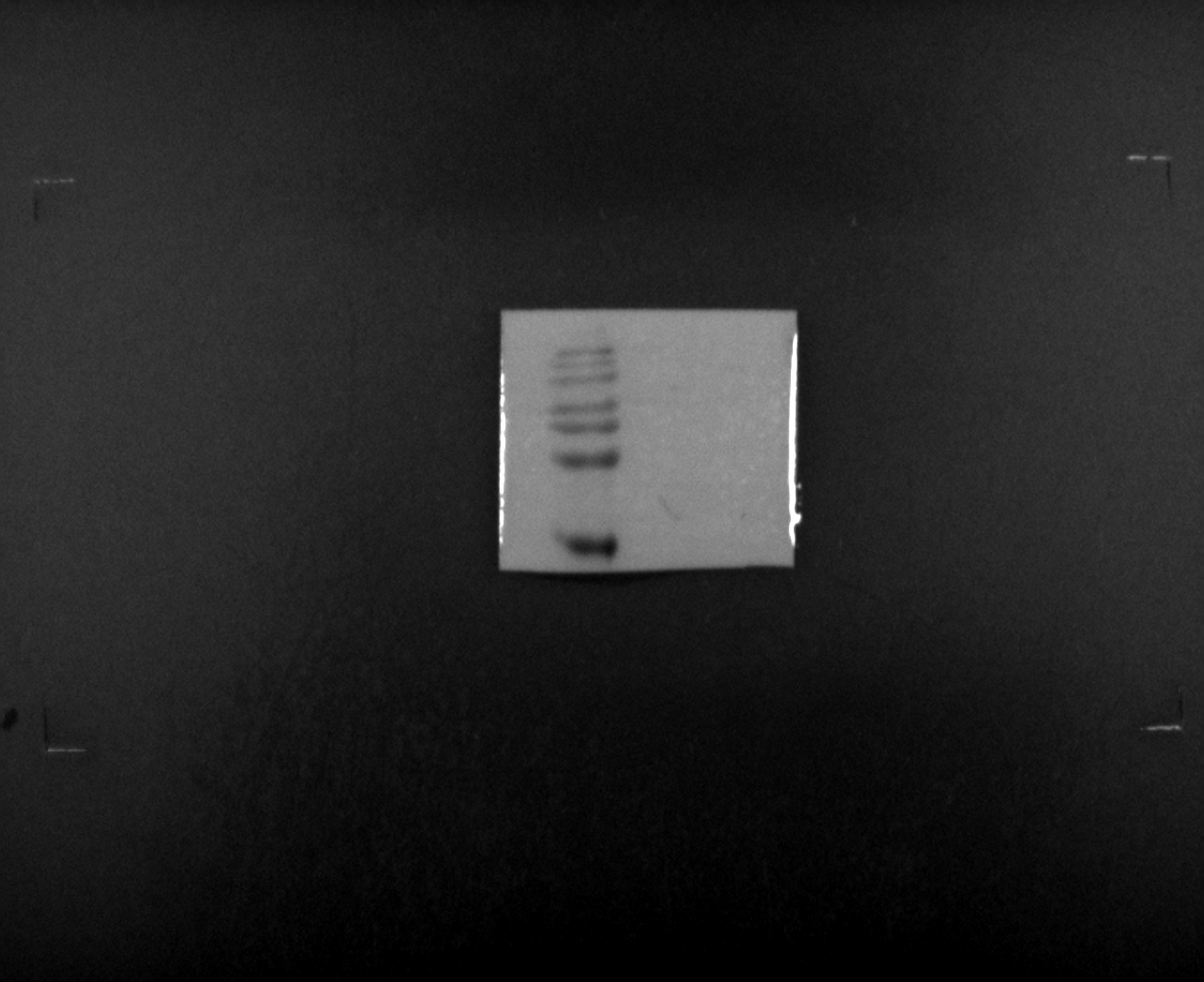

Supplement: Supplemental Information 2 [file peerj-12-17874-s002.zip › fig 1G/bax-3 (2).tif]

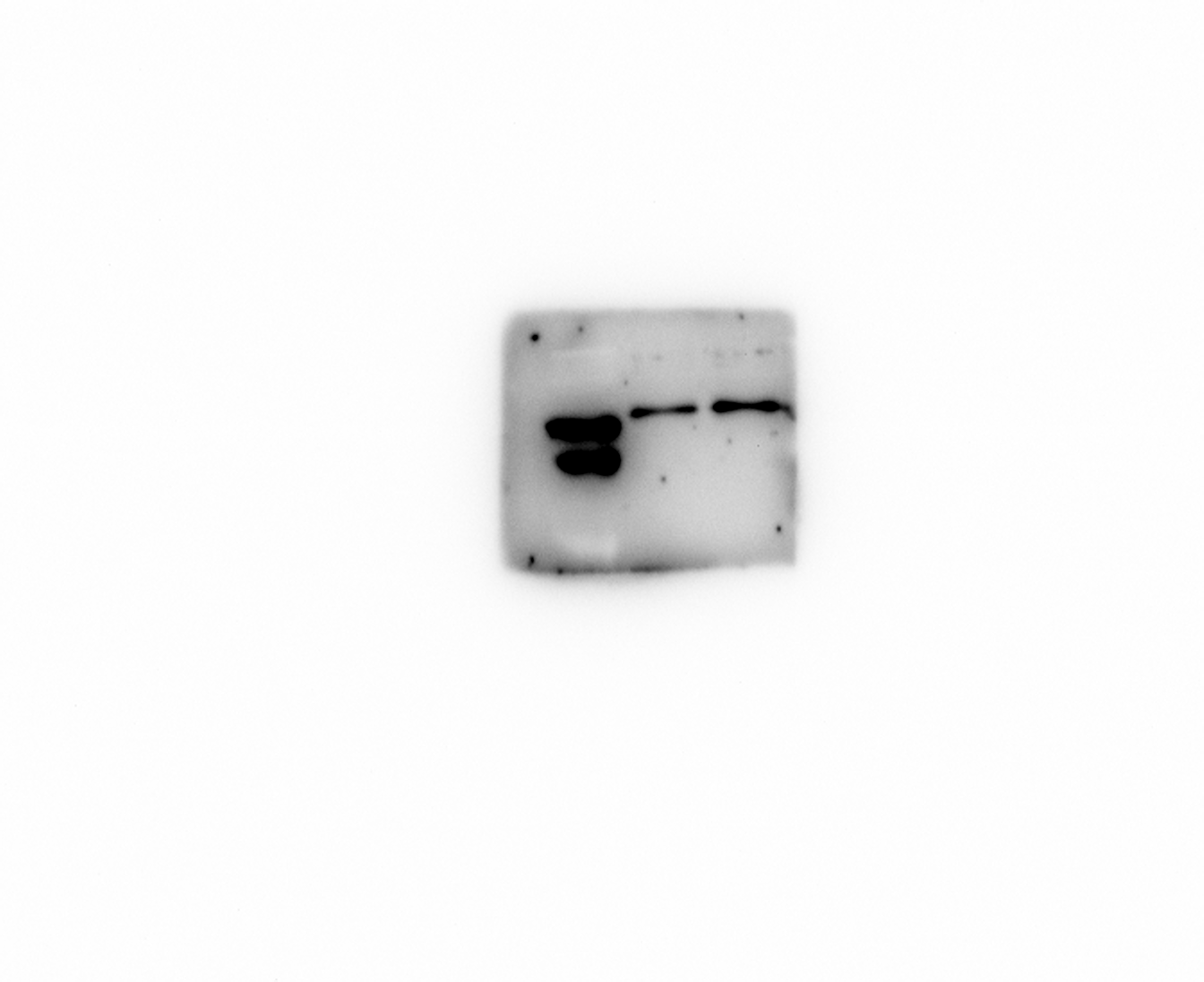

Supplement: Supplemental Information 2 [file peerj-12-17874-s002.zip › fig 1G/bax-3 (3).tif]

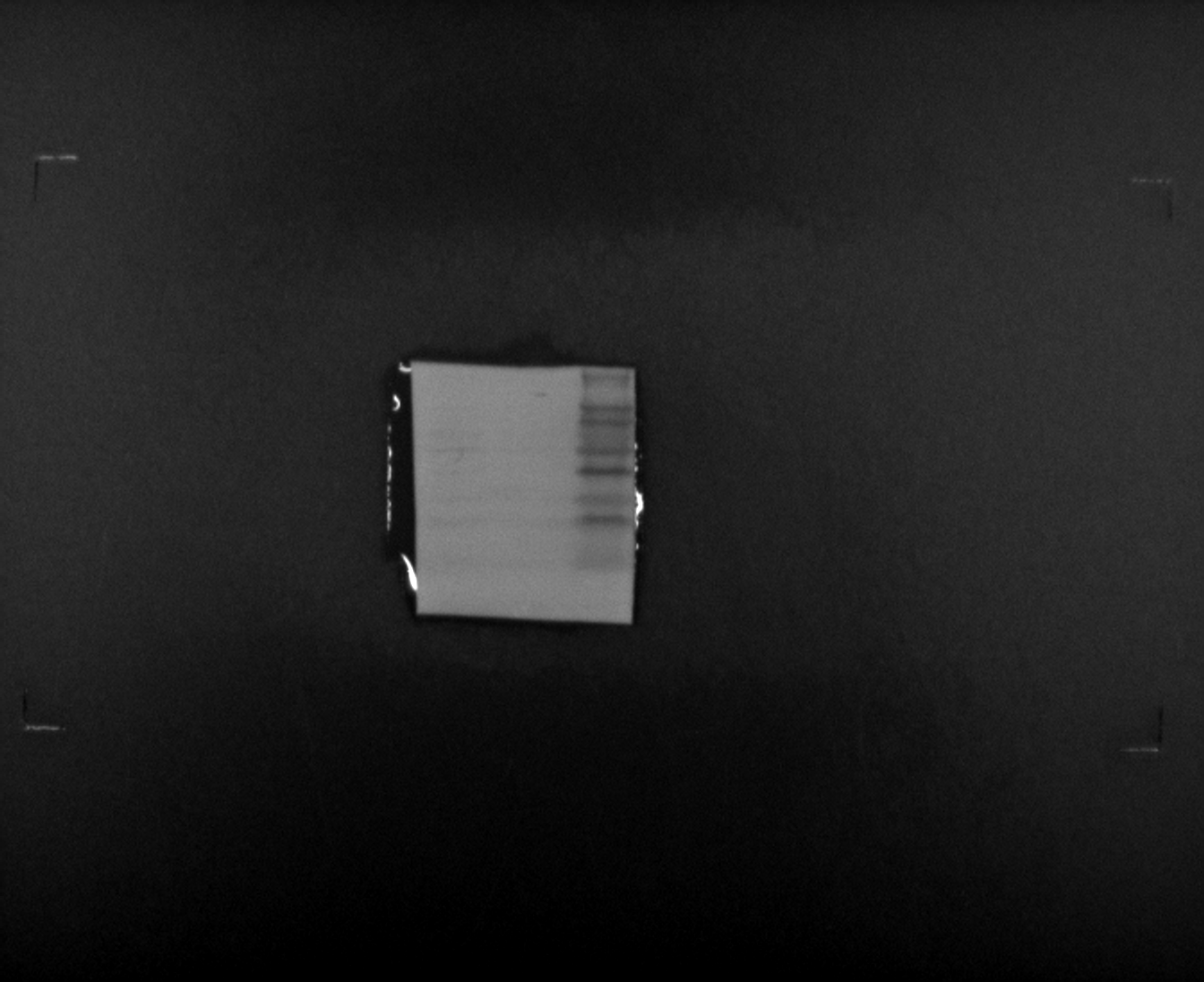

Supplement: Supplemental Information 2 [file peerj-12-17874-s002.zip › fig 1G/bcl2 (1).tif]

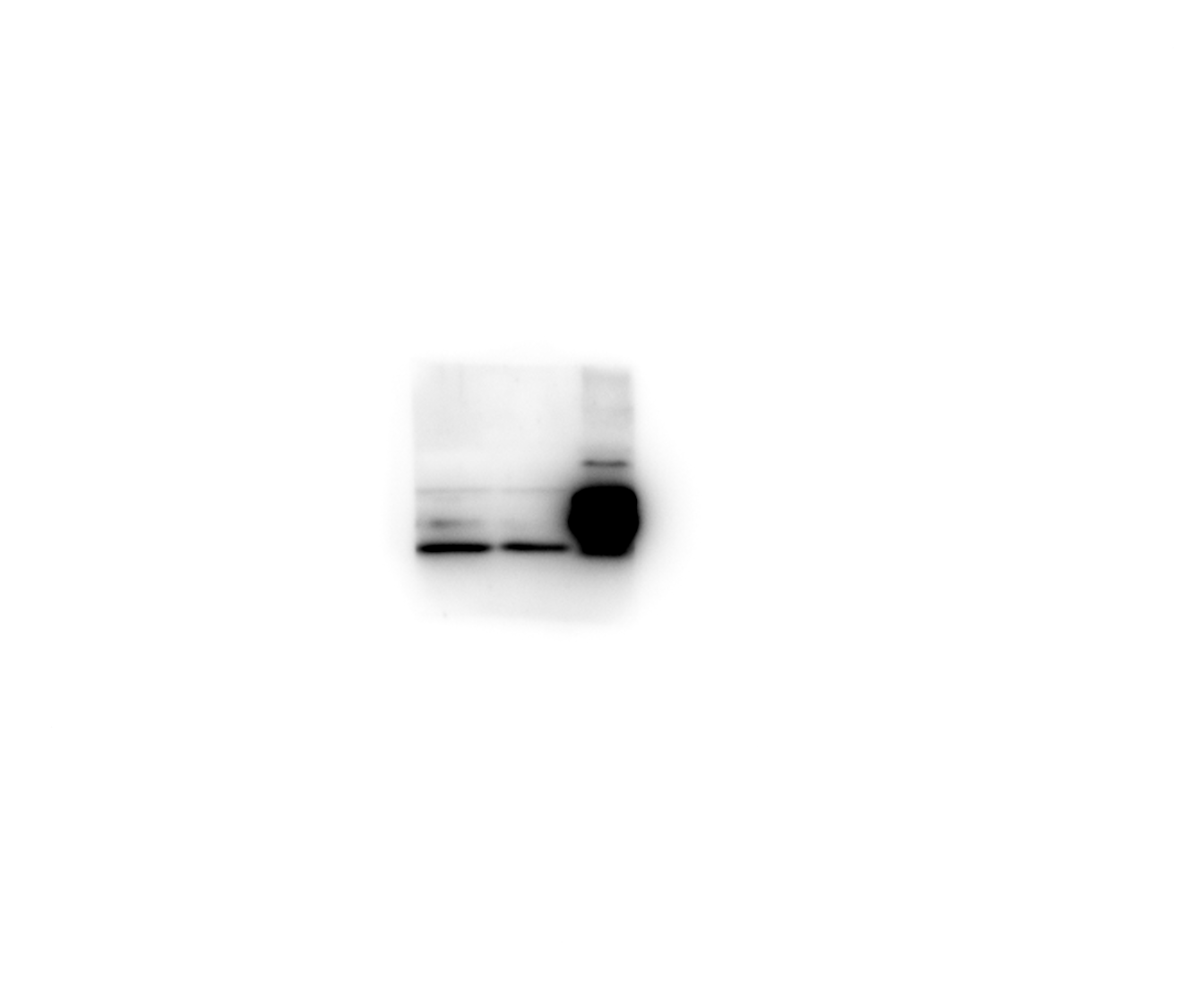

Supplement: Supplemental Information 2 [file peerj-12-17874-s002.zip › fig 1G/bcl2 (2).tif]

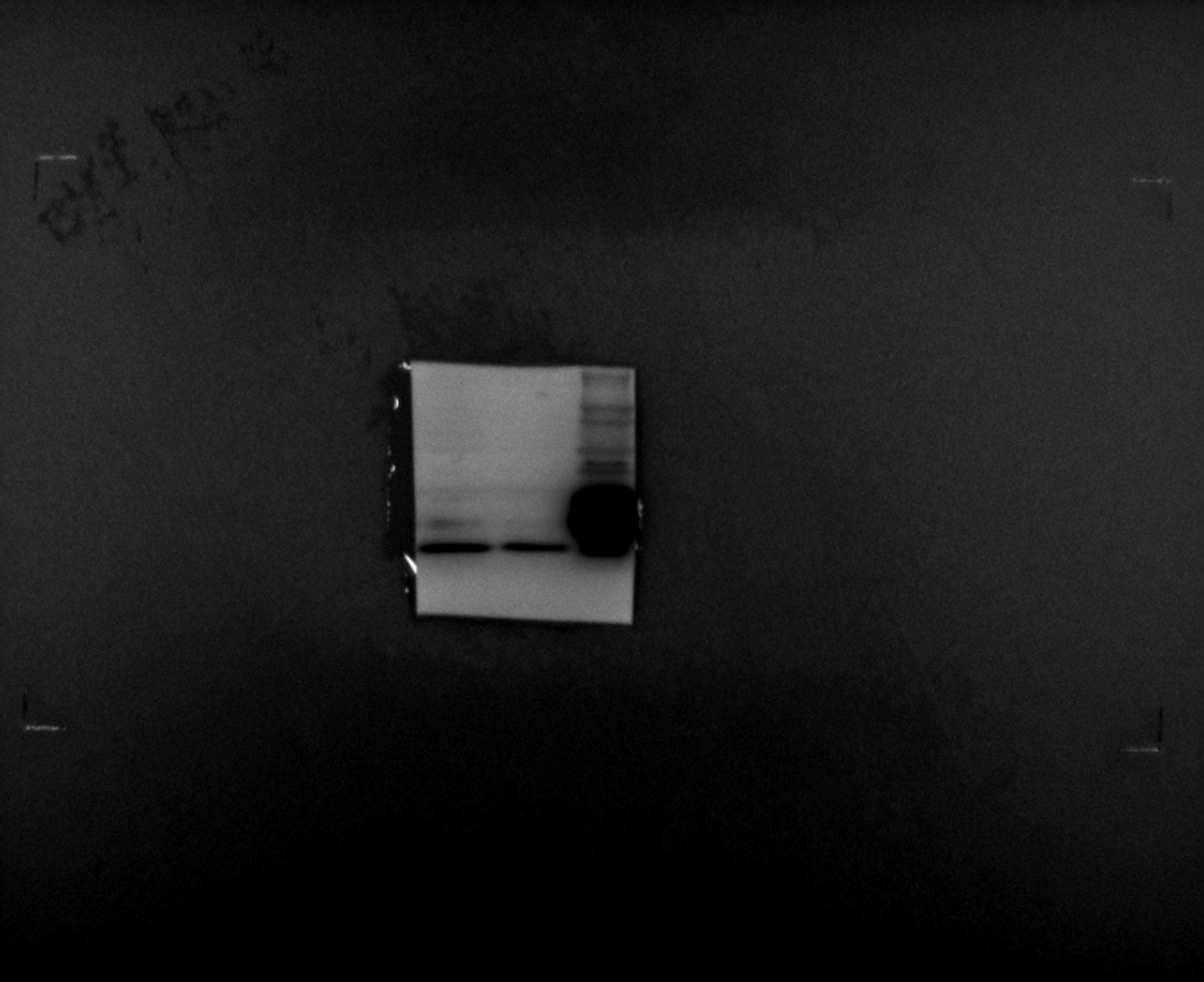

Supplement: Supplemental Information 2 [file peerj-12-17874-s002.zip › fig 1G/bcl2 (3).tif]

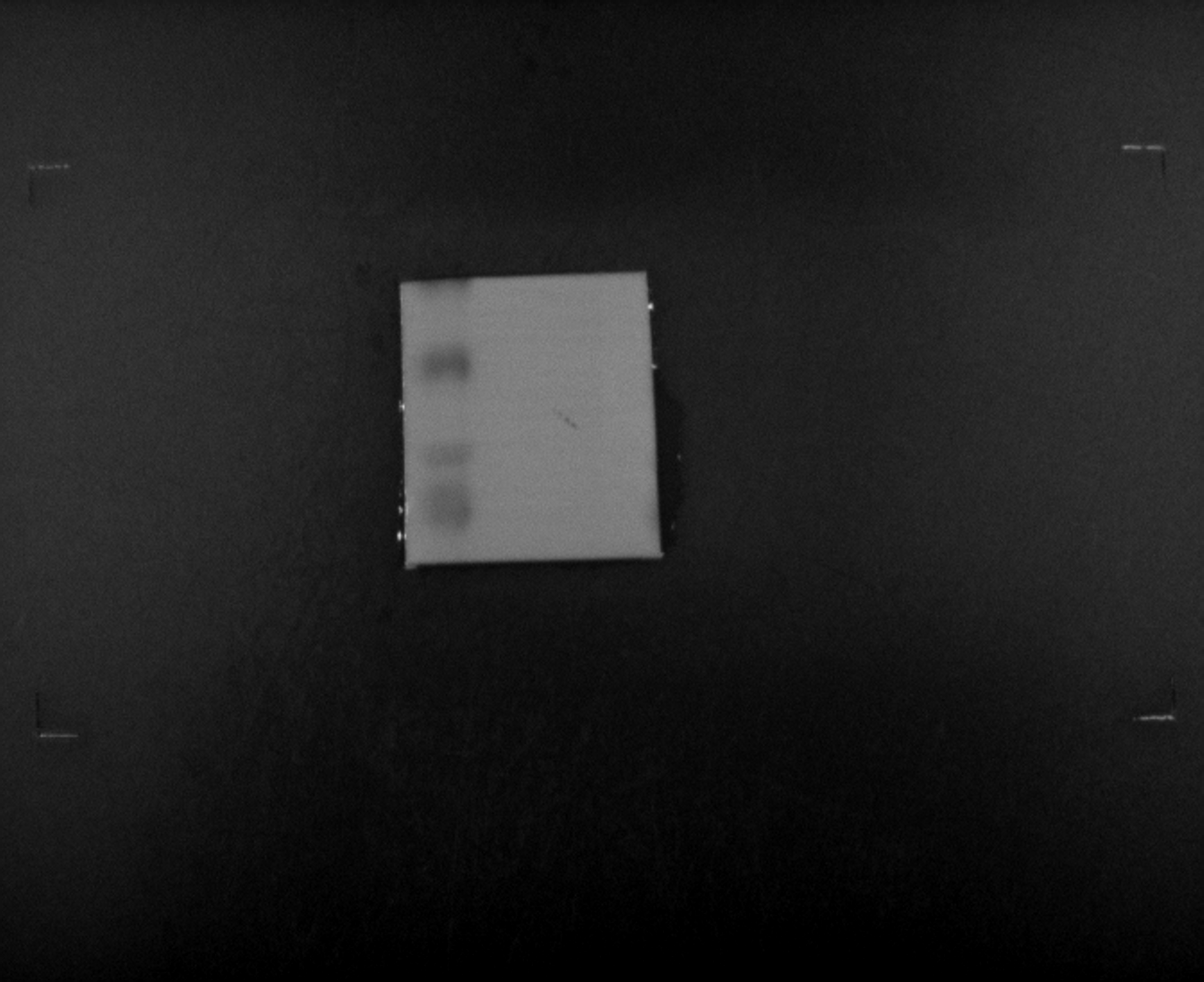

Supplement: Supplemental Information 2 [file peerj-12-17874-s002.zip › fig 1G/bcl2-2 (1).tif]

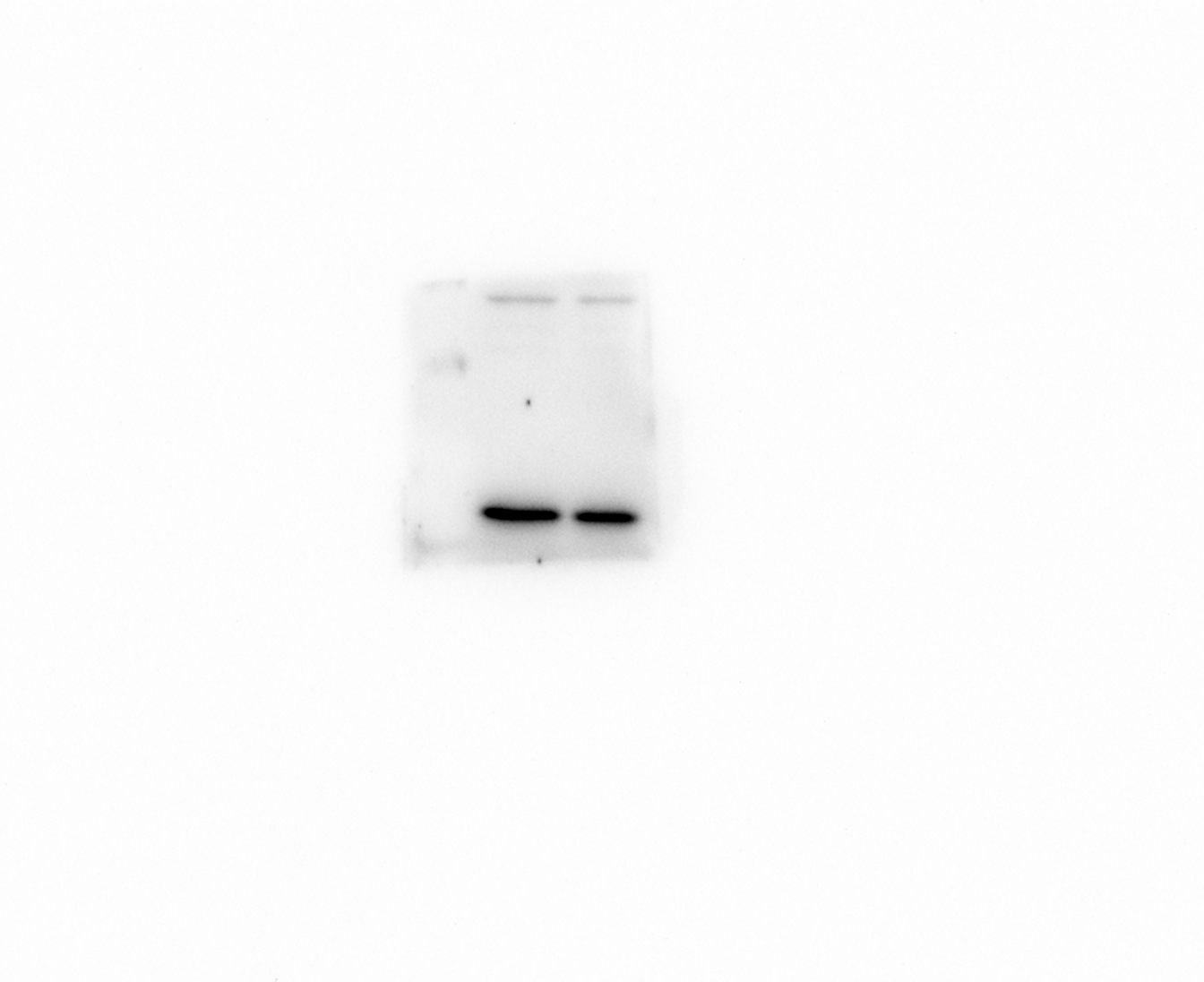

Supplement: Supplemental Information 2 [file peerj-12-17874-s002.zip › fig 1G/bcl2-2 (2).tif]

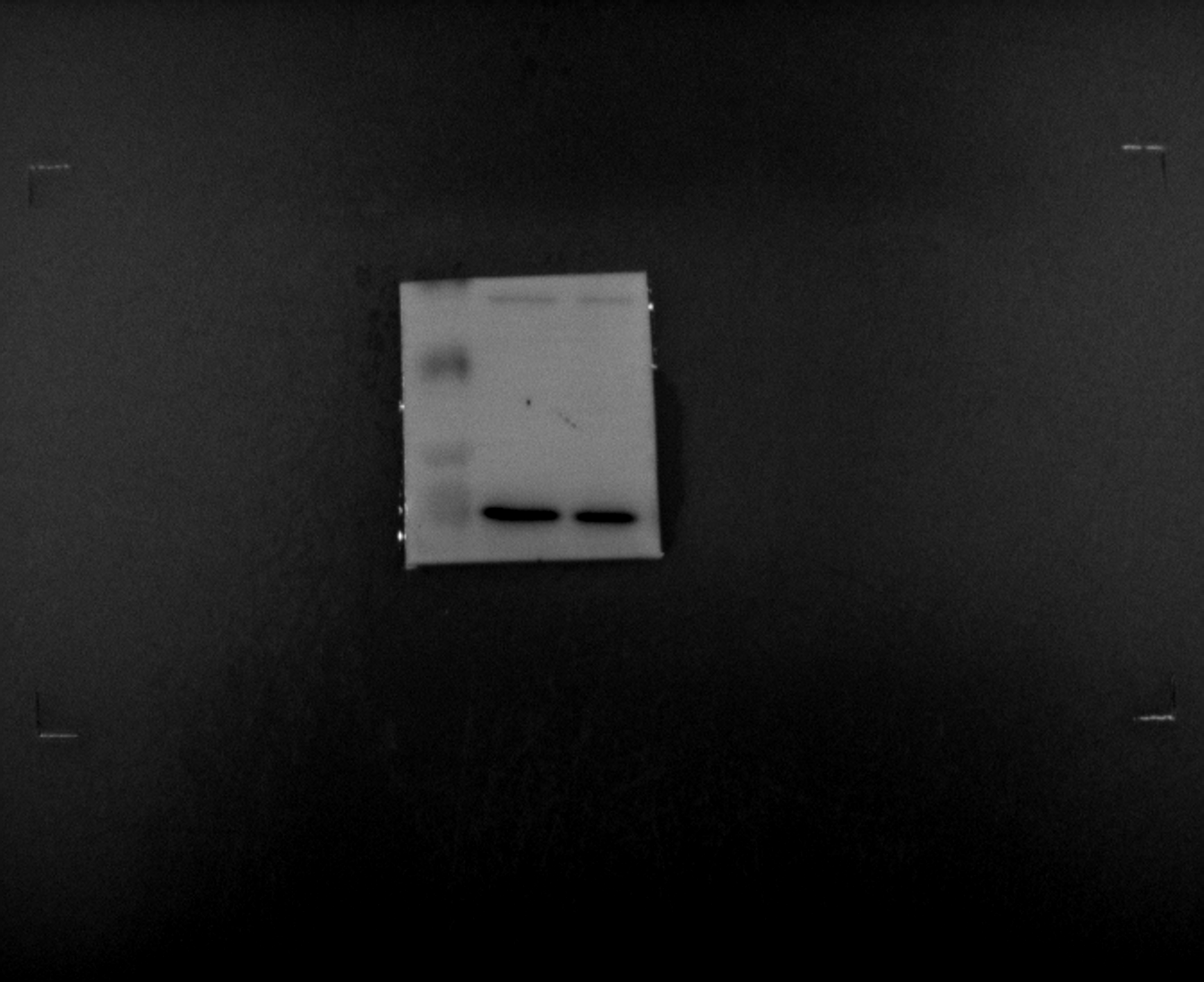

Supplement: Supplemental Information 2 [file peerj-12-17874-s002.zip › fig 1G/bcl2-2 (3).tif]

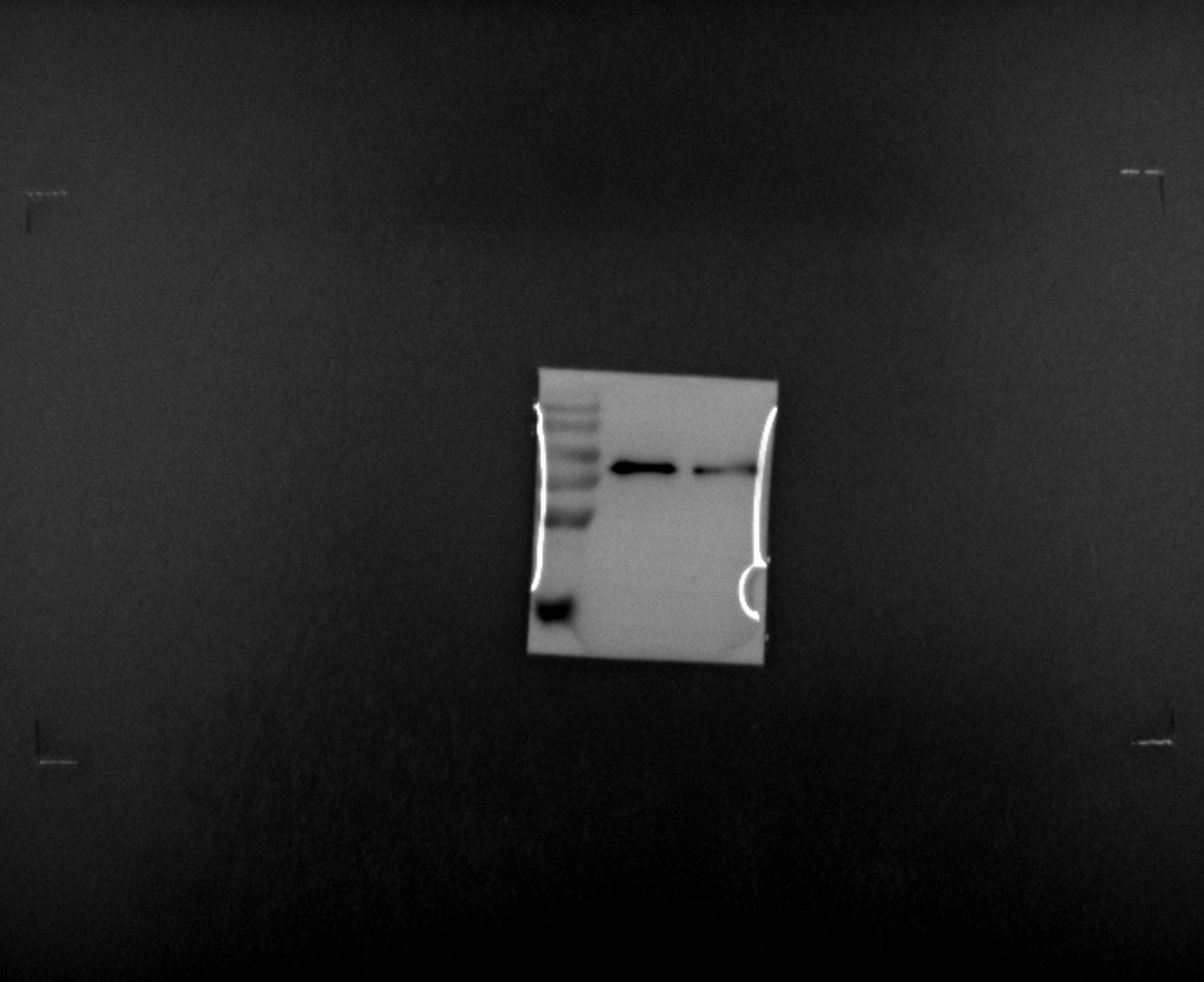

Supplement: Supplemental Information 2 [file peerj-12-17874-s002.zip › fig 1G/bcl2-3 (1).tif]

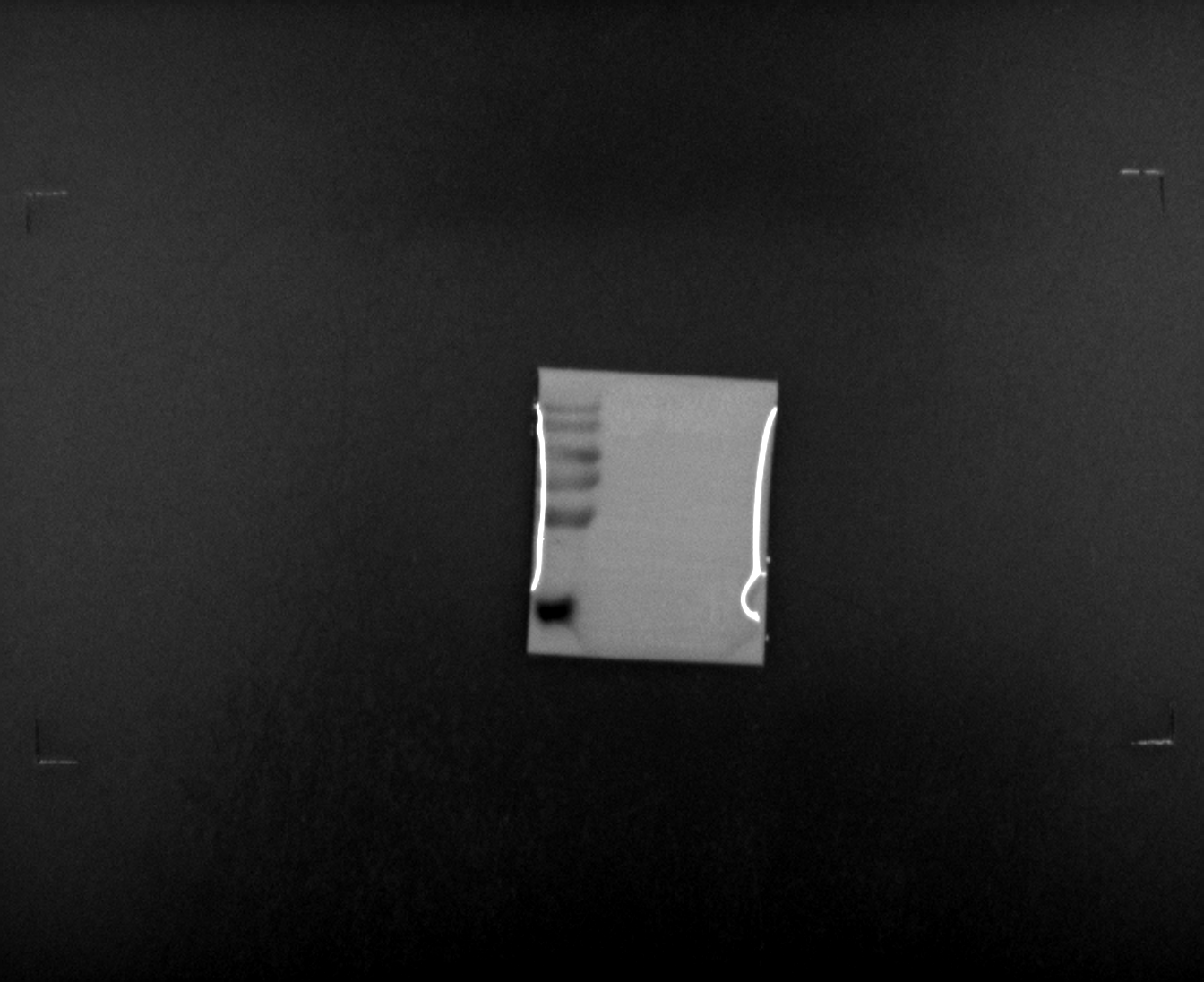

Supplement: Supplemental Information 2 [file peerj-12-17874-s002.zip › fig 1G/bcl2-3 (2).tif]

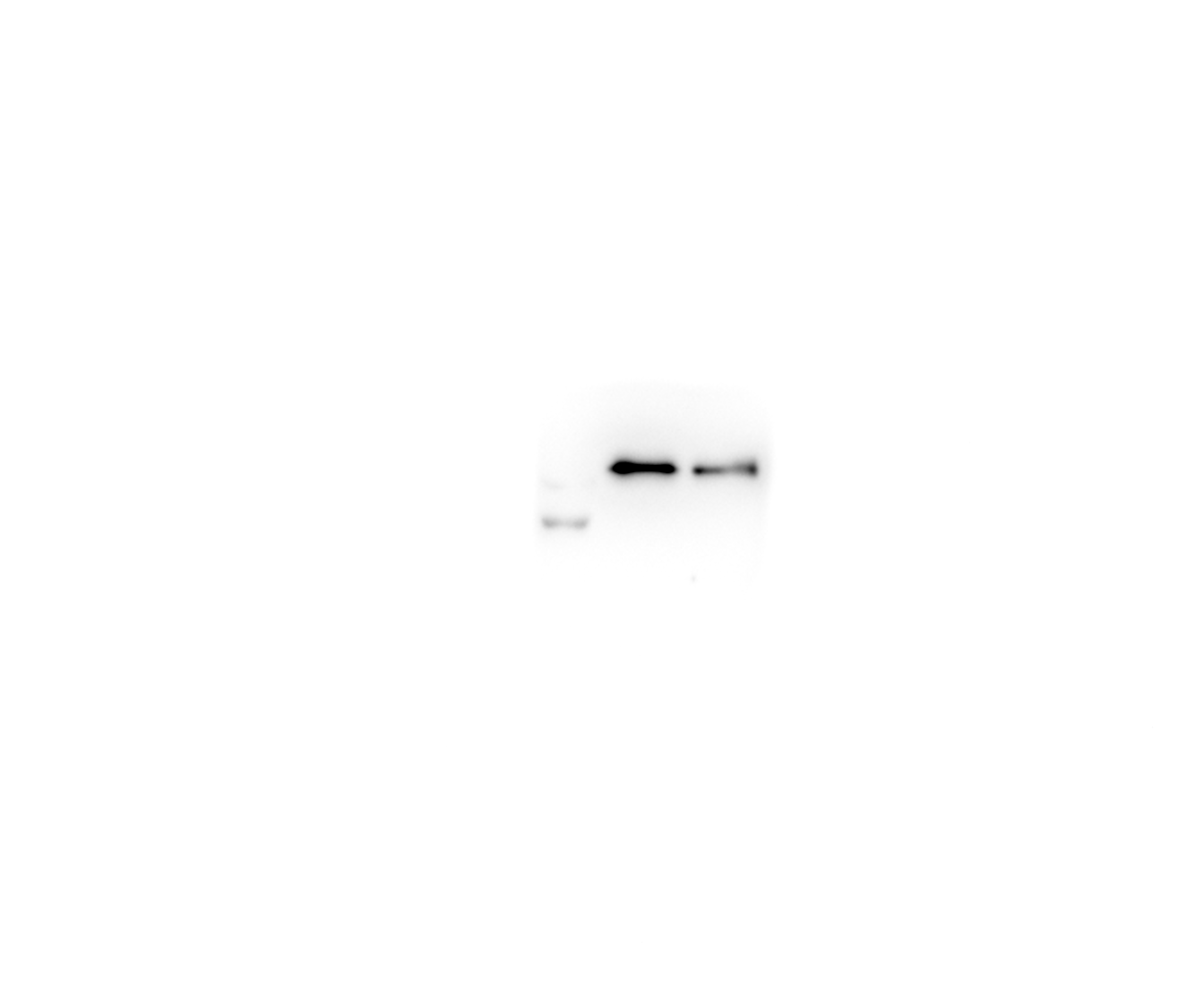

Supplement: Supplemental Information 2 [file peerj-12-17874-s002.zip › fig 1G/bcl2-3 (3).tif]

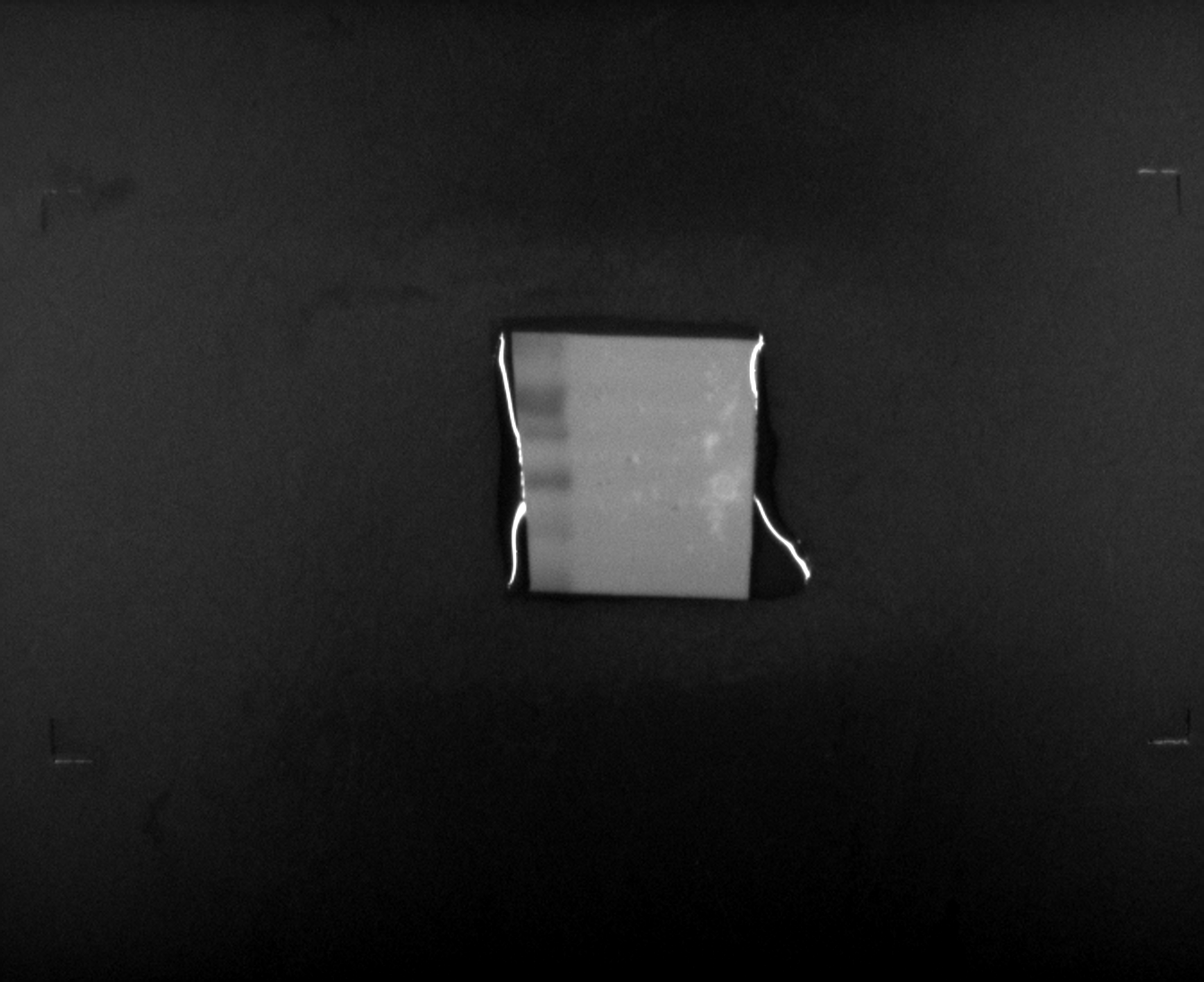

Supplement: Supplemental Information 2 [file peerj-12-17874-s002.zip › fig 1G/CASPASE1 (1).tif]

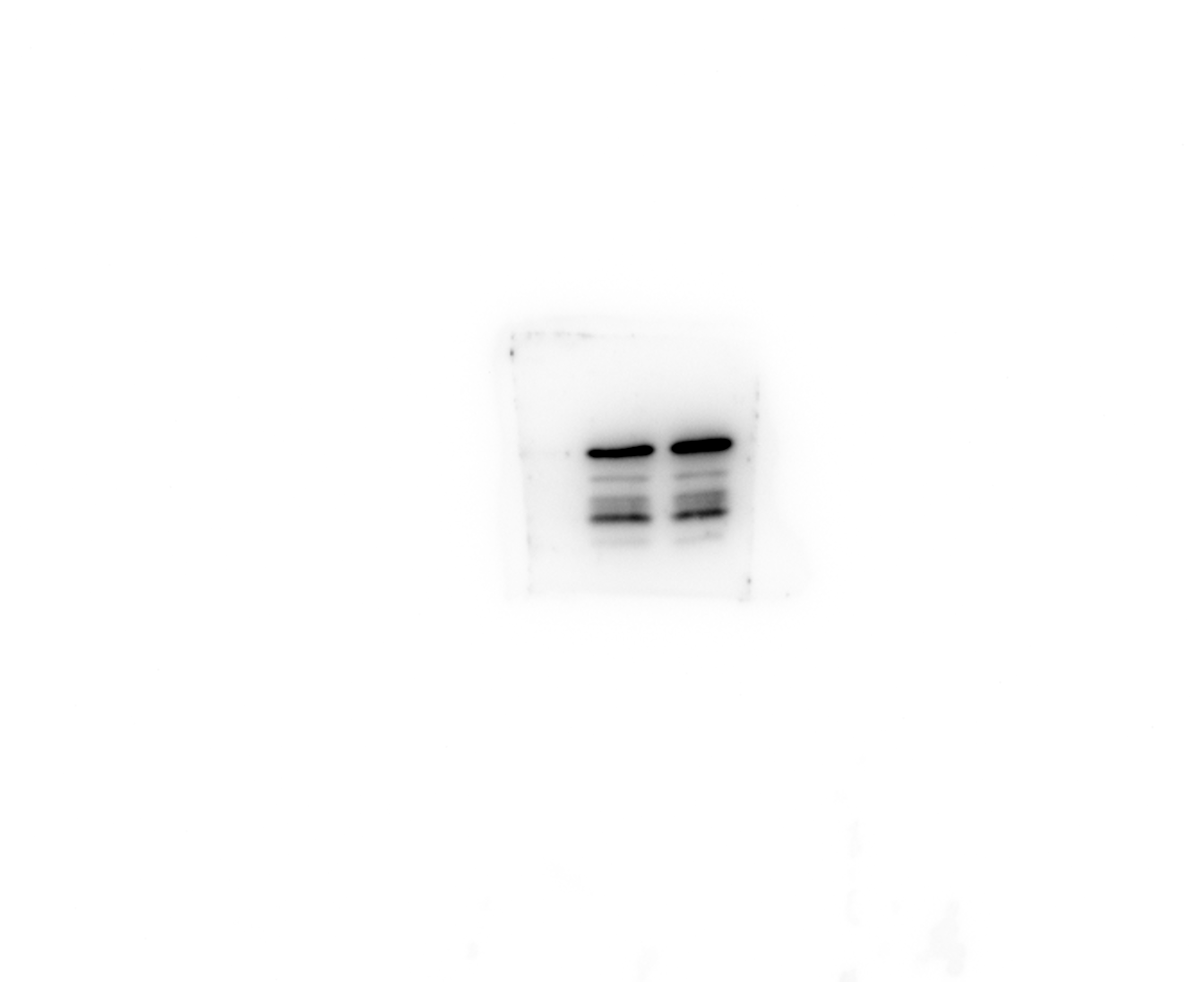

Supplement: Supplemental Information 2 [file peerj-12-17874-s002.zip › fig 1G/CASPASE1 (2).tif]

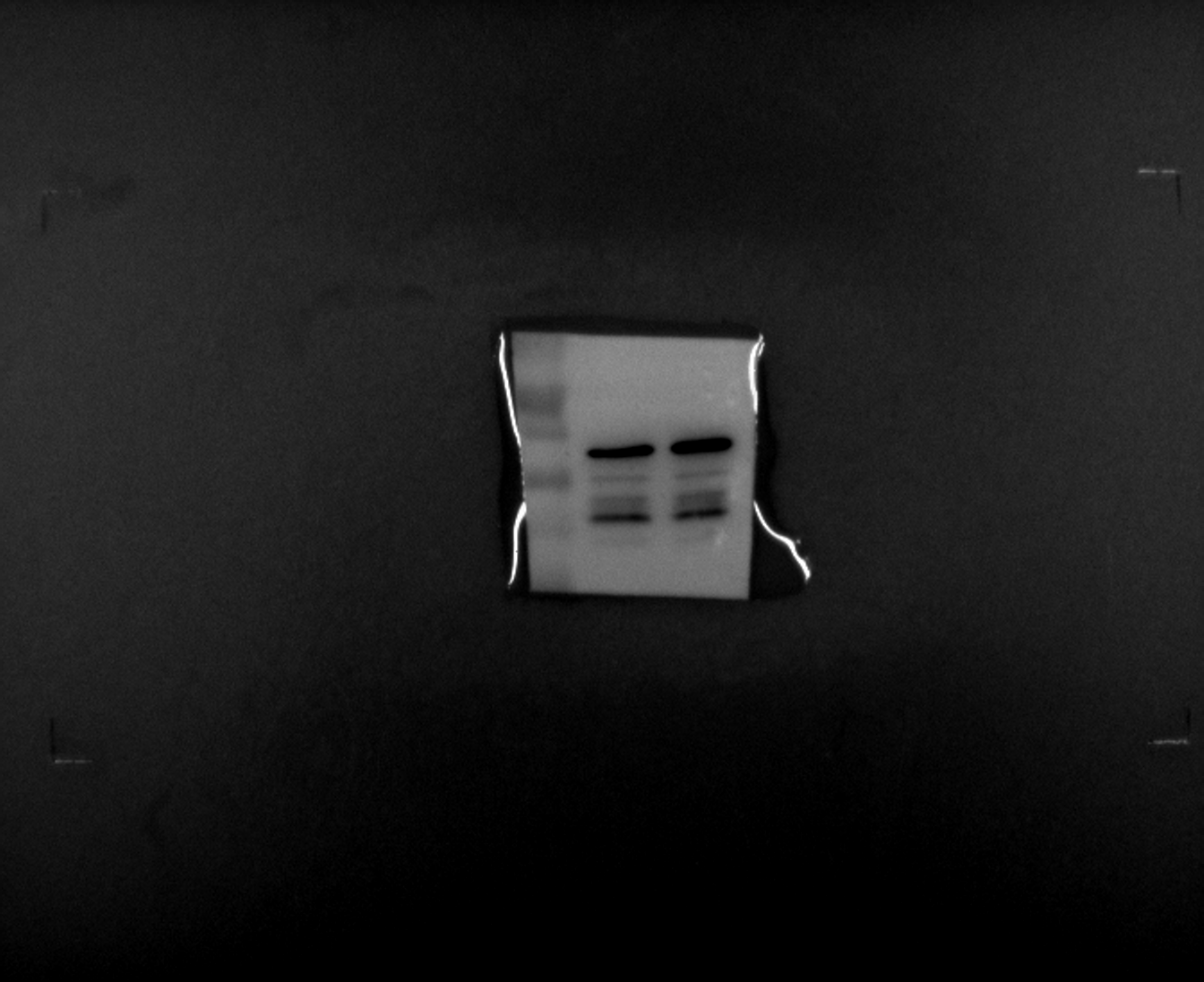

Supplement: Supplemental Information 2 [file peerj-12-17874-s002.zip › fig 1G/CASPASE1 (3).tif]

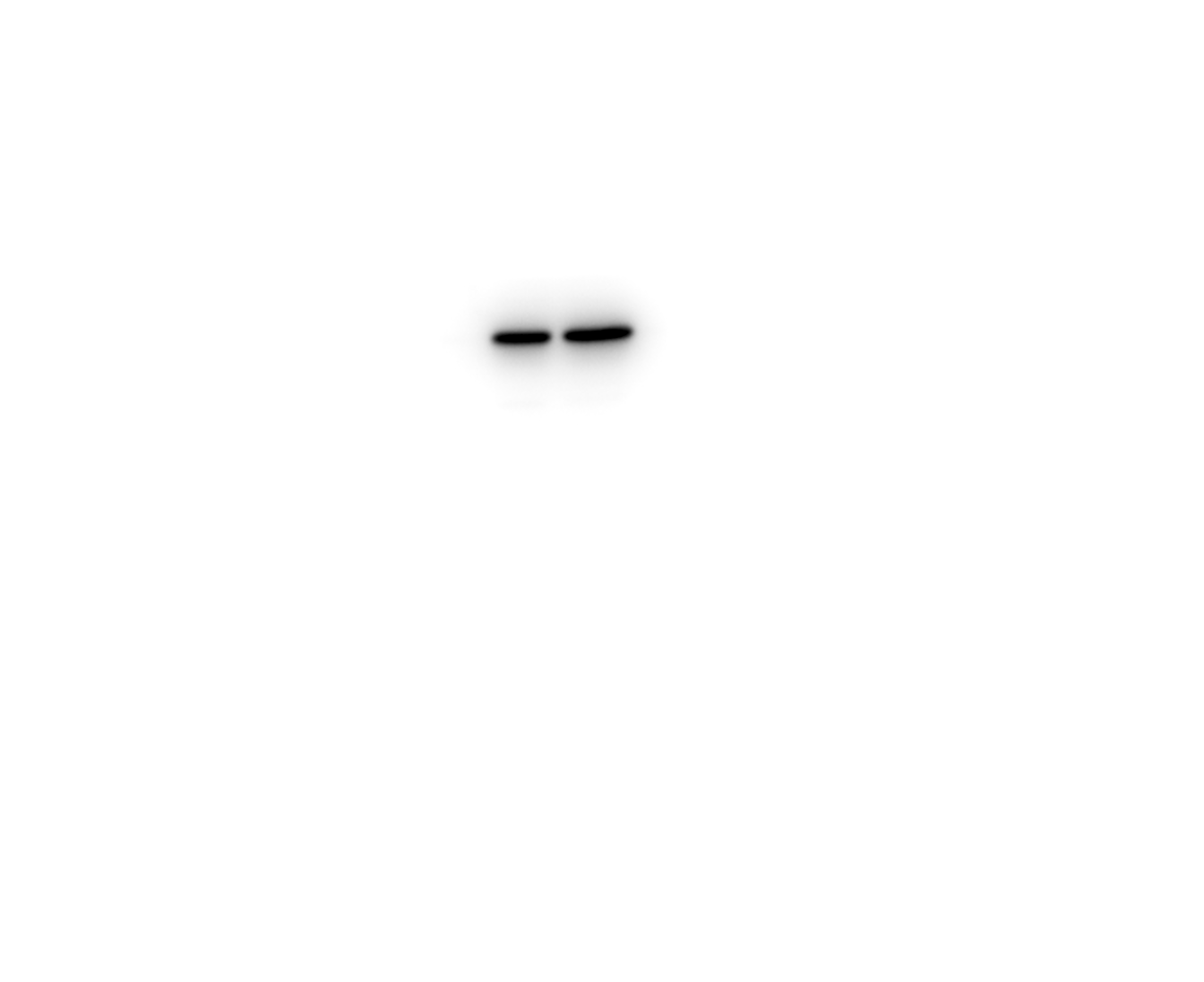

Supplement: Supplemental Information 2 [file peerj-12-17874-s002.zip › fig 1G/caspase1-2 (1).tif]

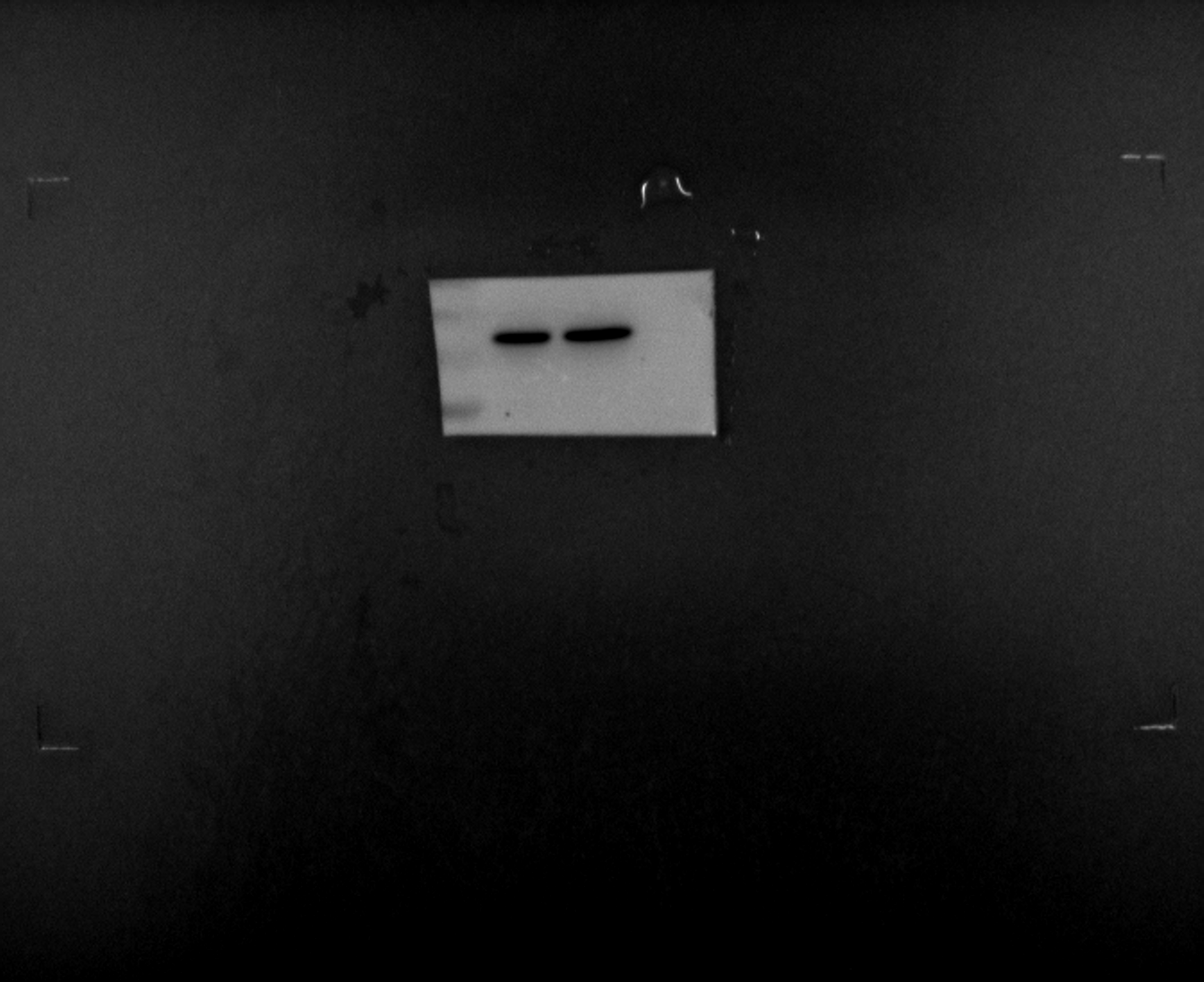

Supplement: Supplemental Information 2 [file peerj-12-17874-s002.zip › fig 1G/caspase1-2 (2).tif]

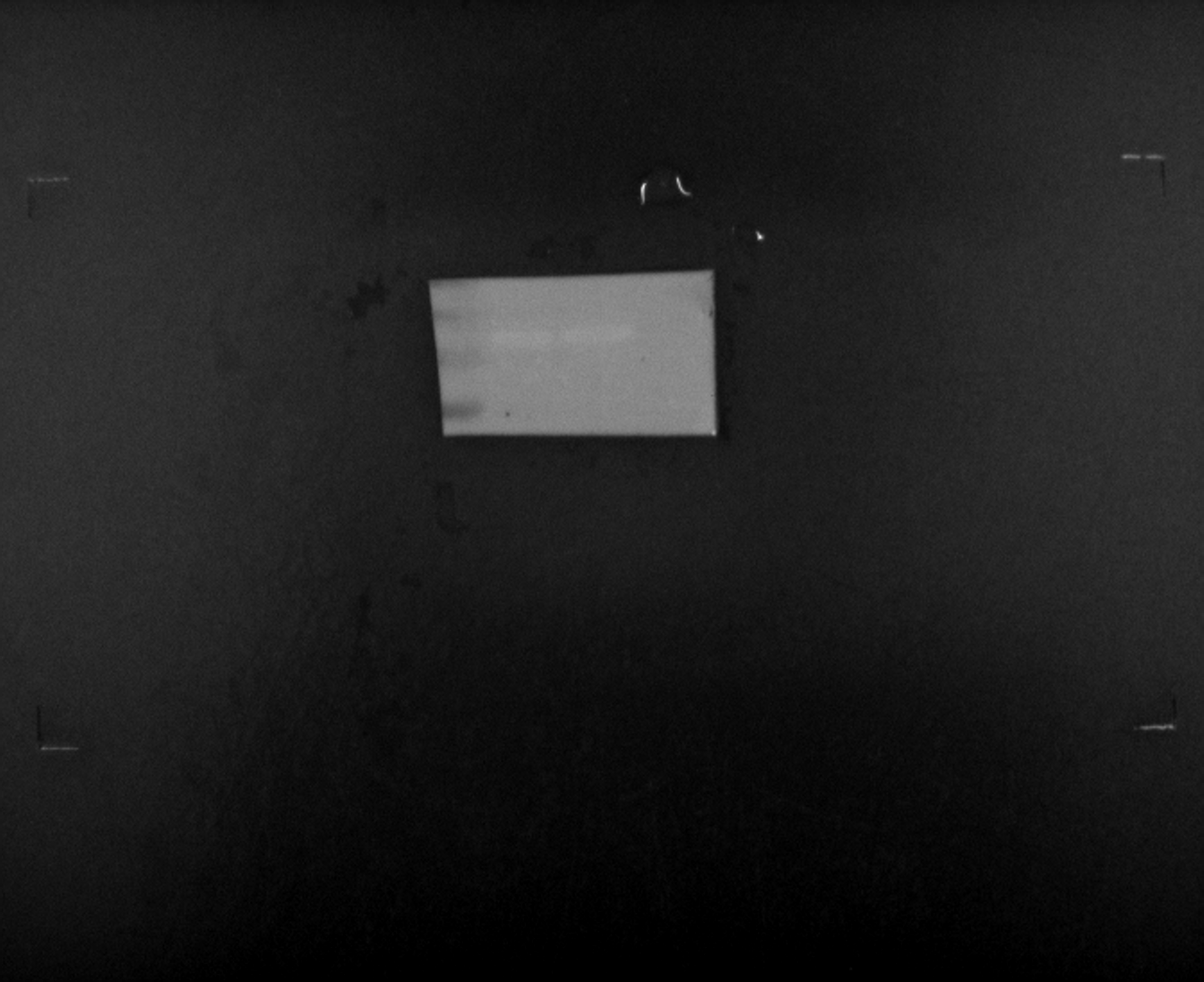

Supplement: Supplemental Information 2 [file peerj-12-17874-s002.zip › fig 1G/caspase1-2 (3).tif]

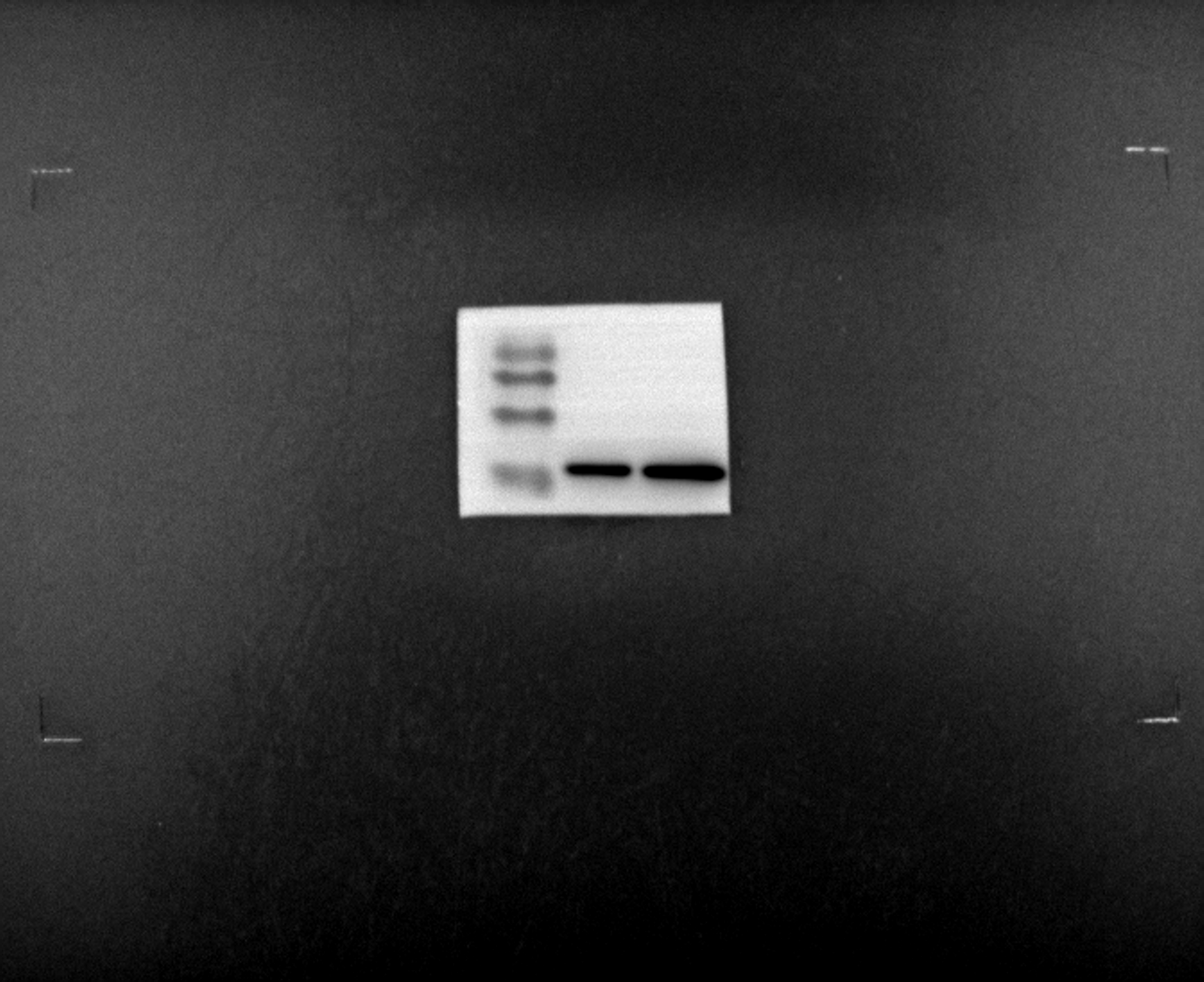

Supplement: Supplemental Information 2 [file peerj-12-17874-s002.zip › fig 1G/caspase1-3 (1).tif]

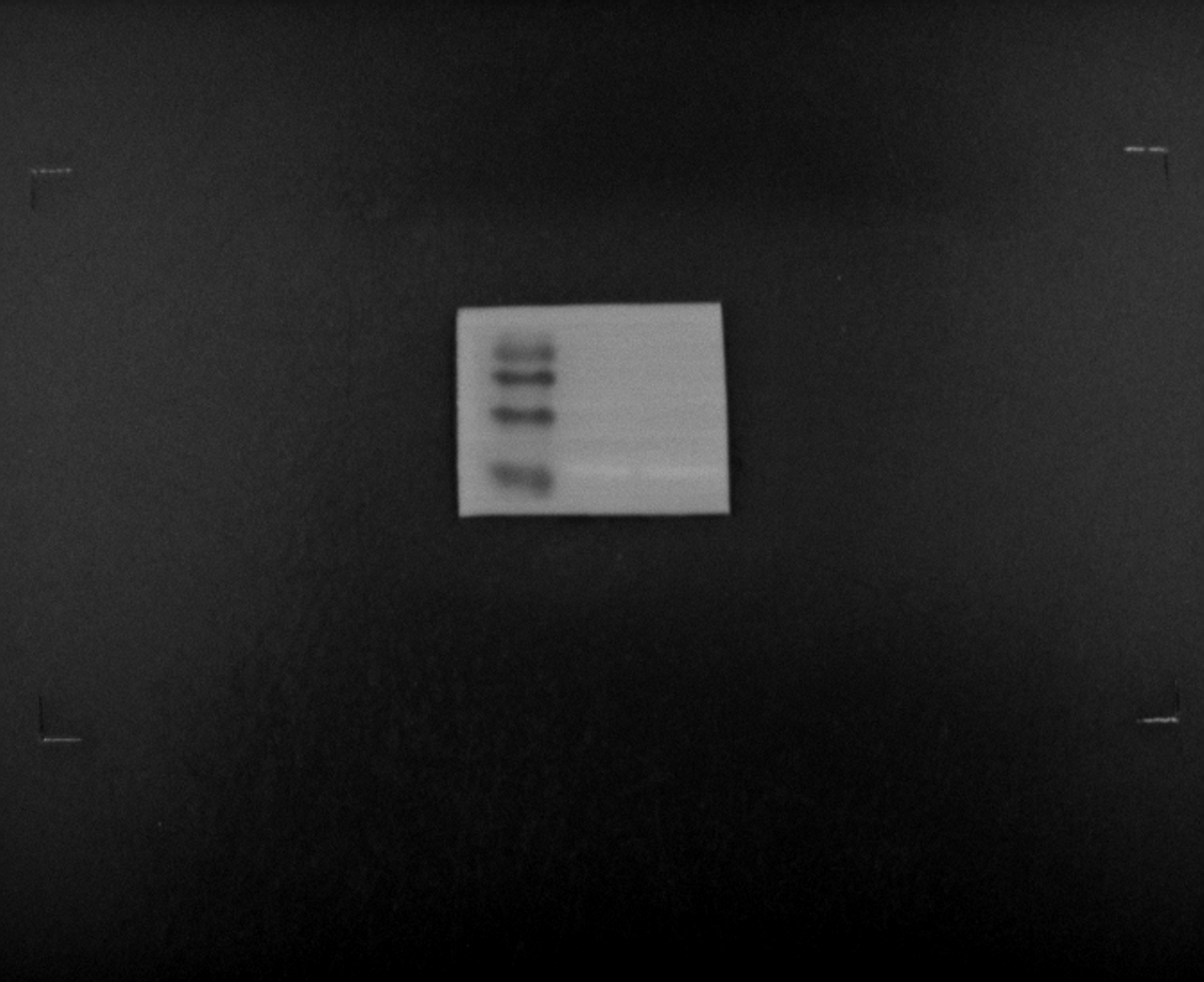

Supplement: Supplemental Information 2 [file peerj-12-17874-s002.zip › fig 1G/caspase1-3 (2).tif]

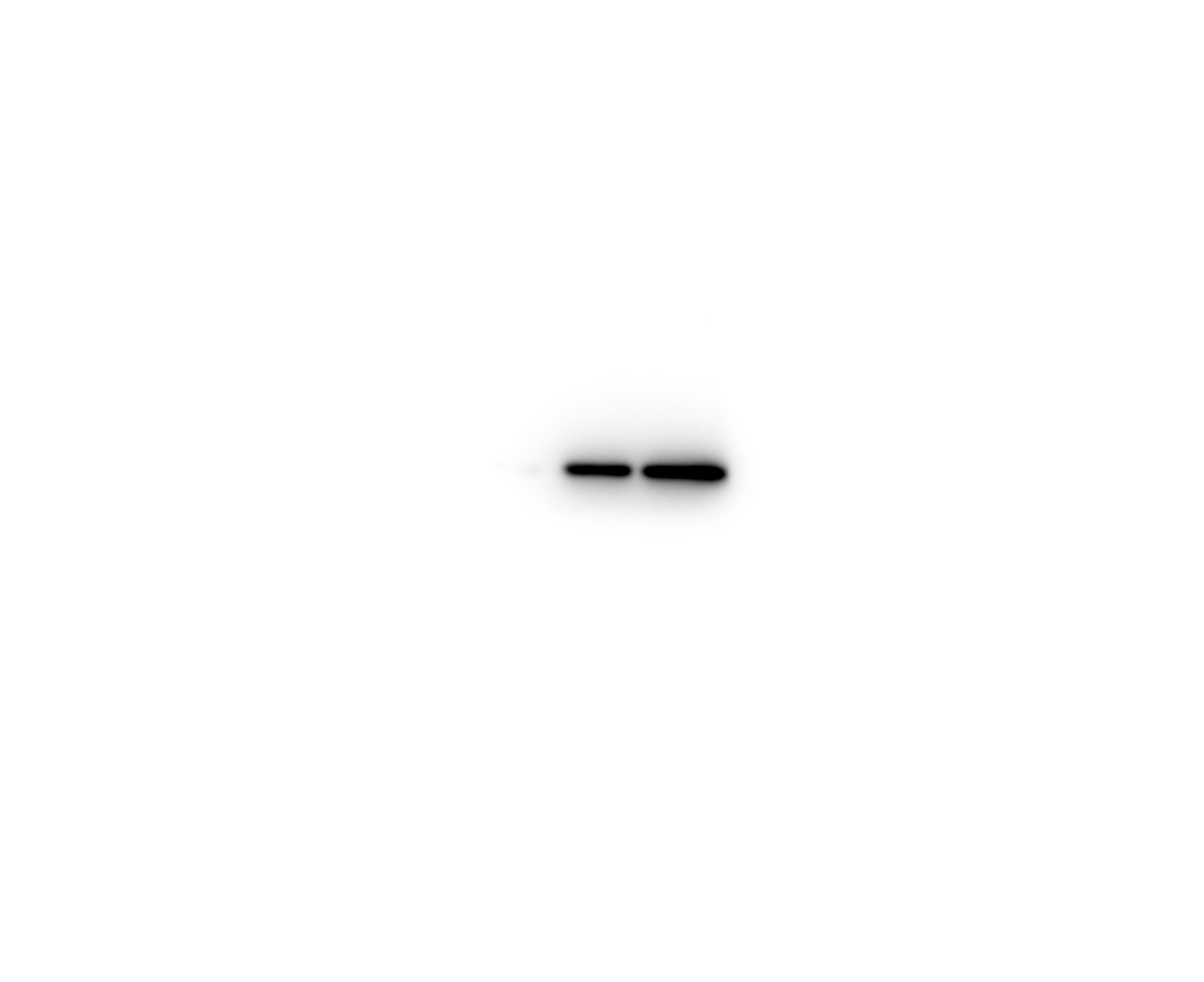

Supplement: Supplemental Information 2 [file peerj-12-17874-s002.zip › fig 1G/caspase1-3 (3).tif]

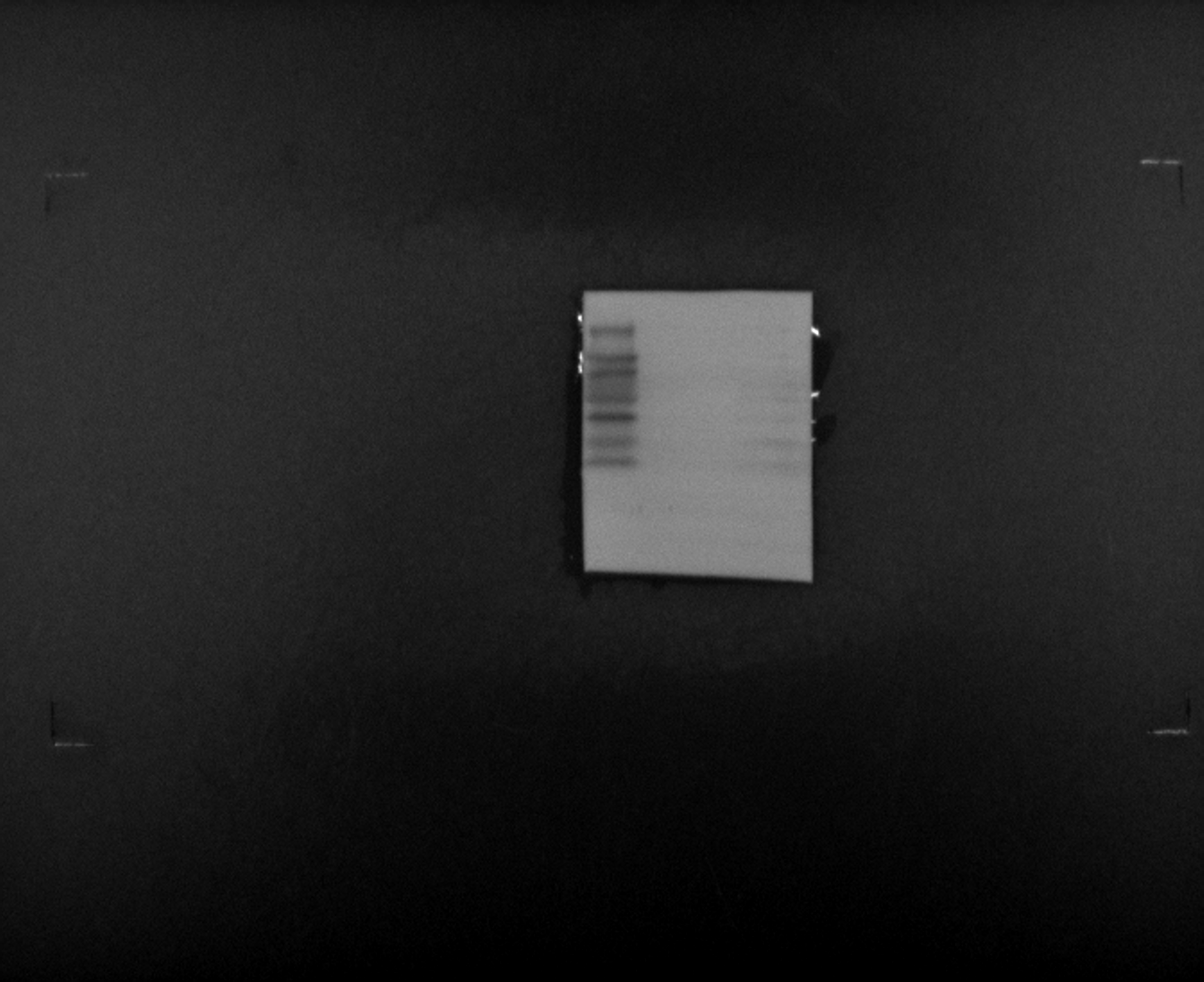

Supplement: Supplemental Information 2 [file peerj-12-17874-s002.zip › fig 1G/duox2 (1).tif]

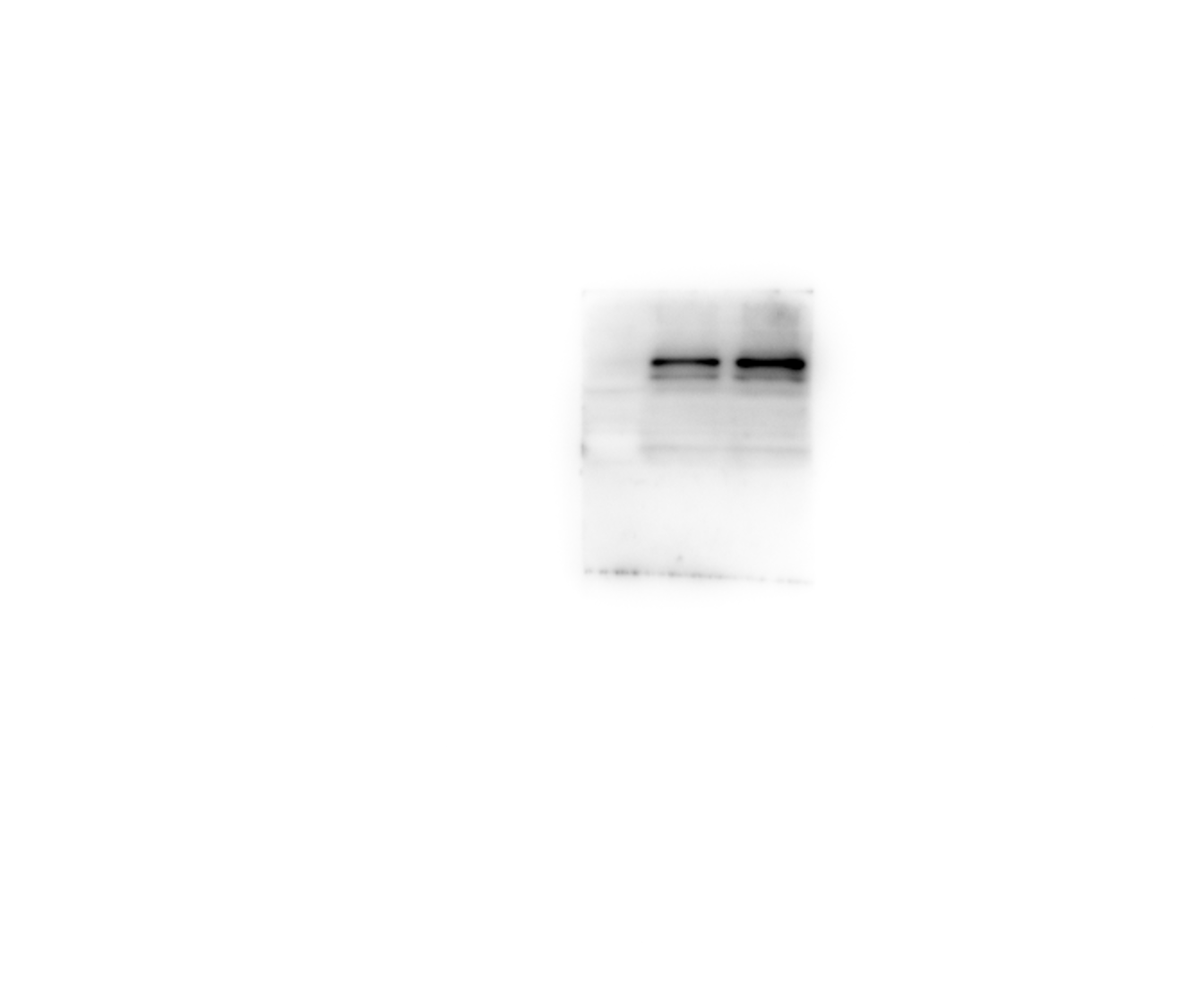

Supplement: Supplemental Information 2 [file peerj-12-17874-s002.zip › fig 1G/duox2 (2).tif]

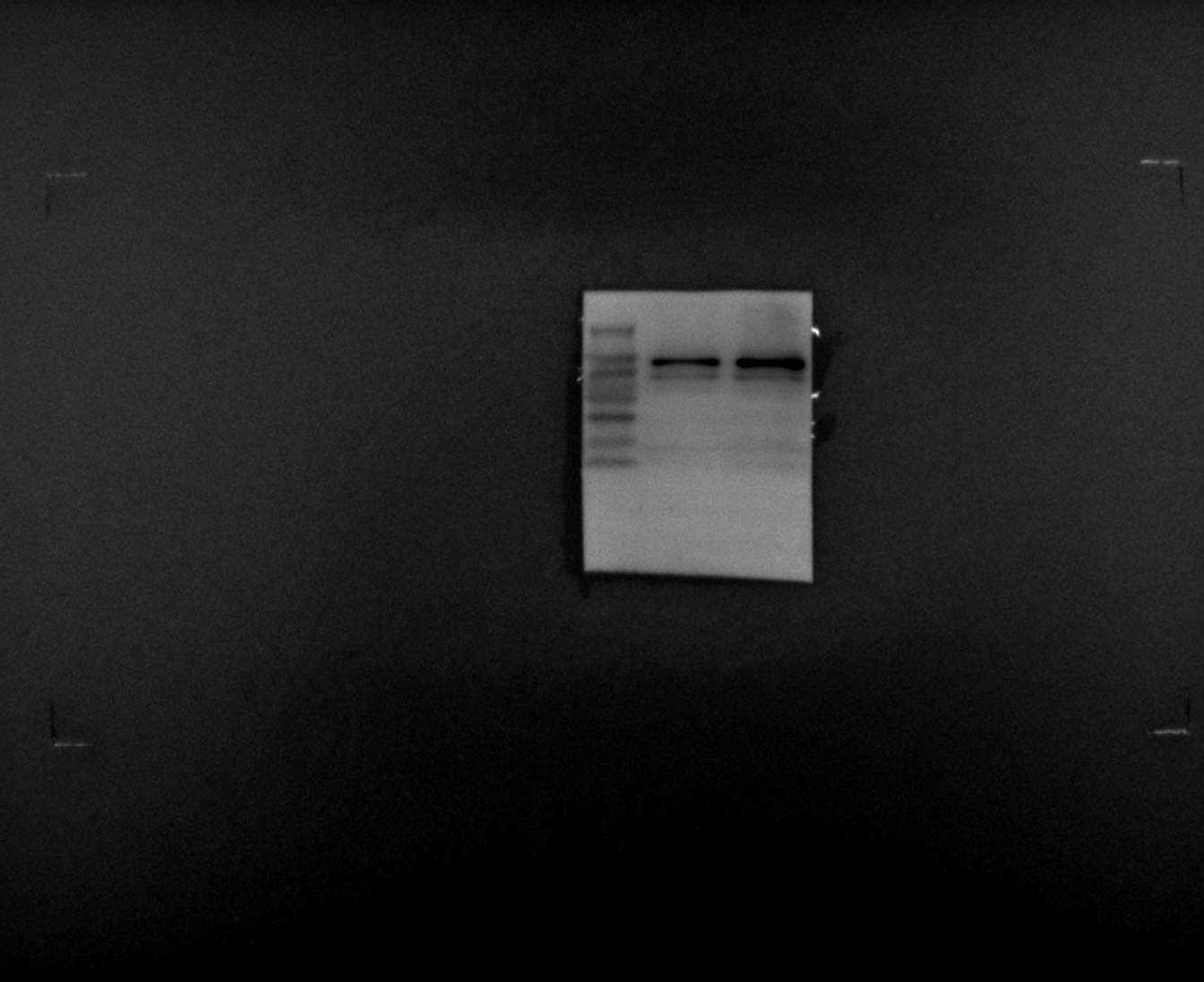

Supplement: Supplemental Information 2 [file peerj-12-17874-s002.zip › fig 1G/duox2 (3).tif]

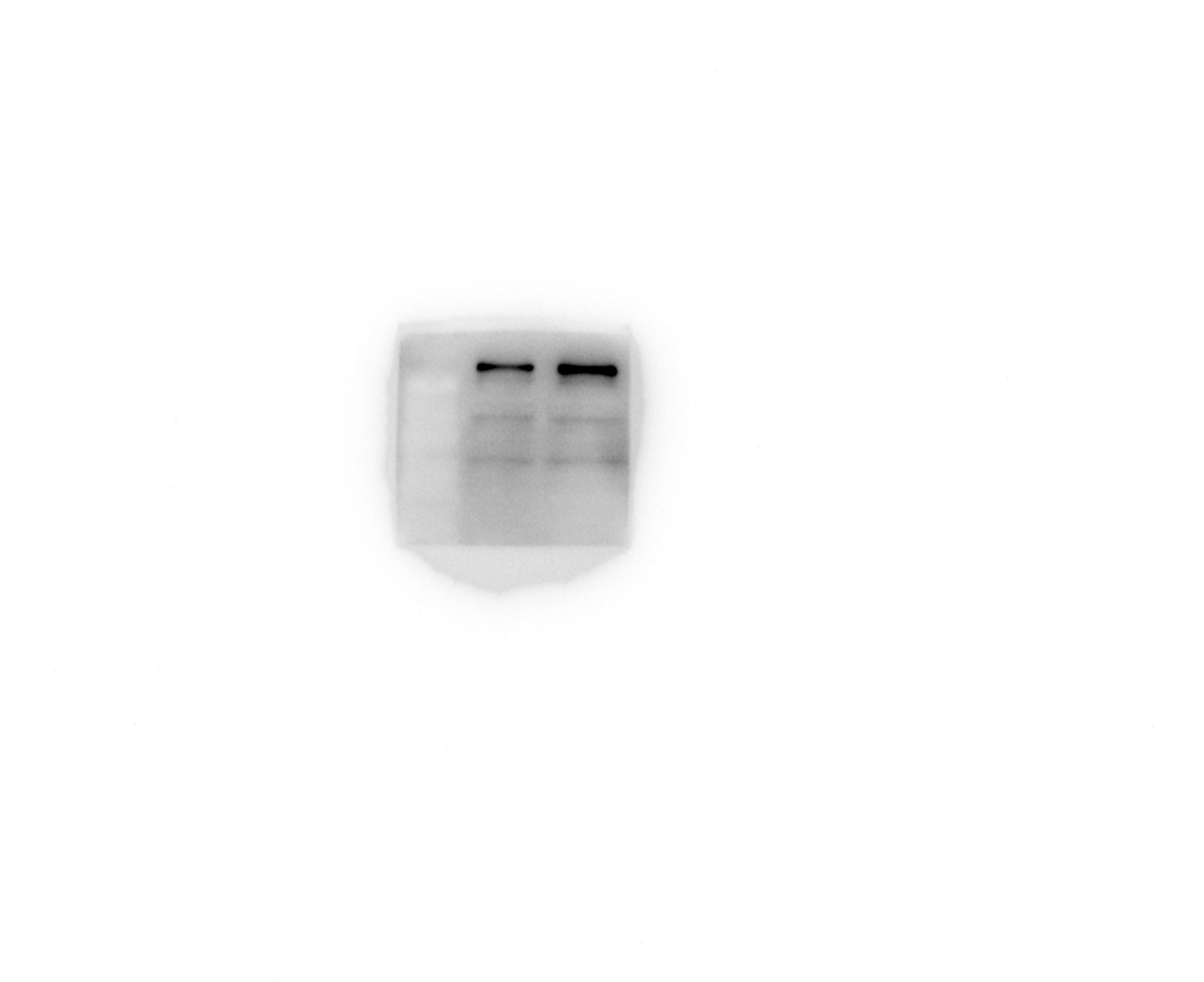

Supplement: Supplemental Information 2 [file peerj-12-17874-s002.zip › fig 1G/DUOX2-2 (1).tif]

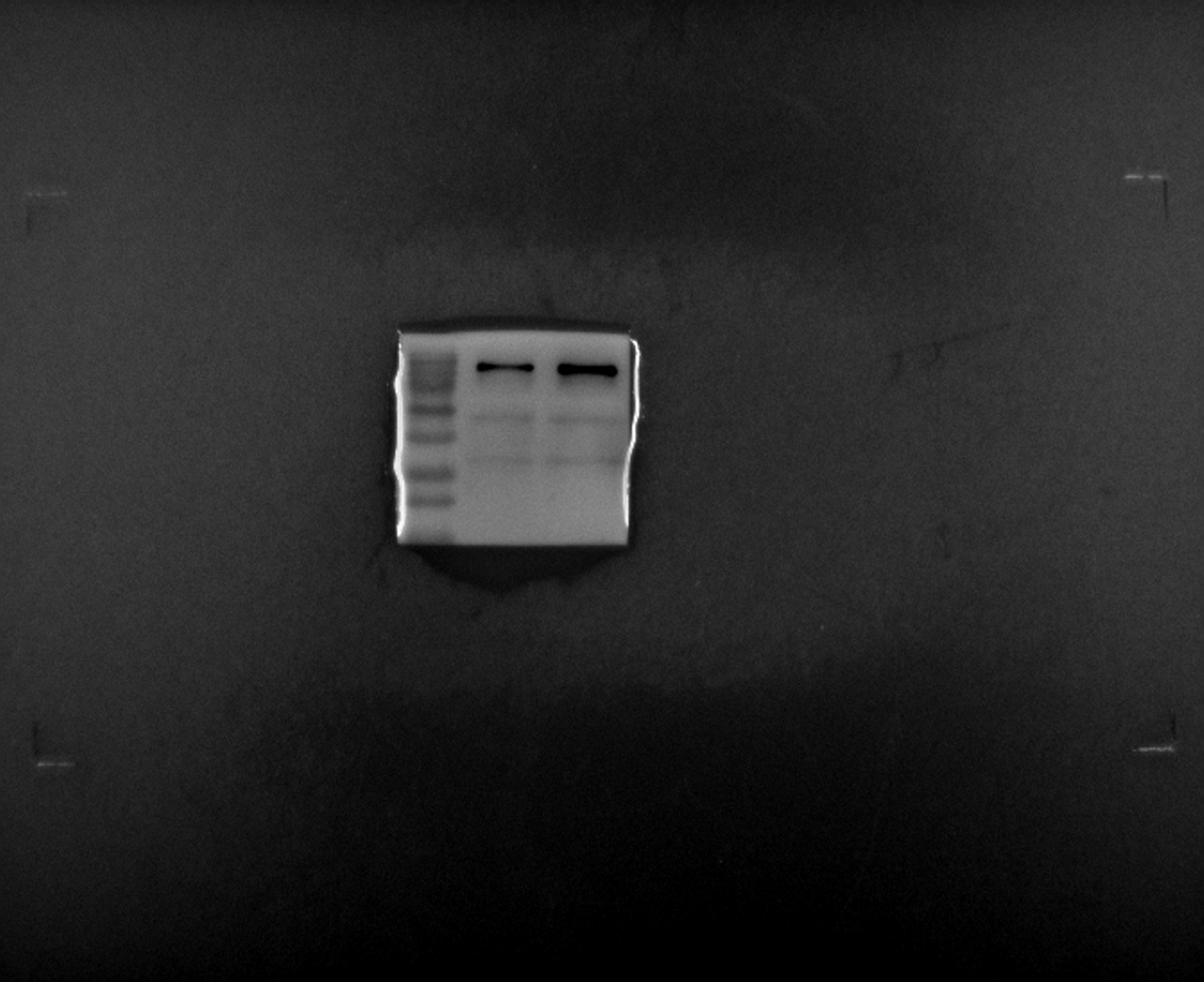

Supplement: Supplemental Information 2 [file peerj-12-17874-s002.zip › fig 1G/DUOX2-2 (2).tif]

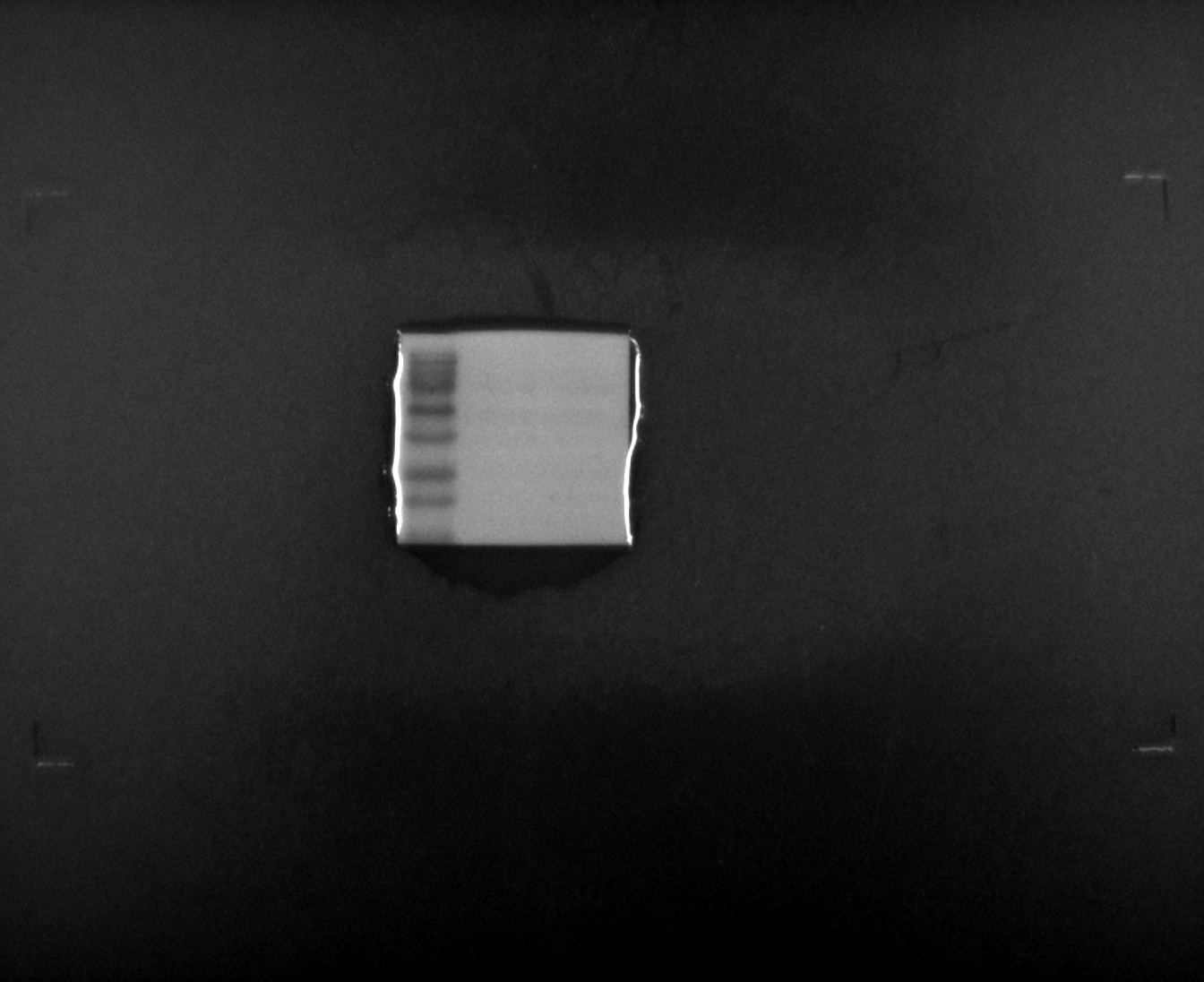

Supplement: Supplemental Information 2 [file peerj-12-17874-s002.zip › fig 1G/DUOX2-2 (3).tif]

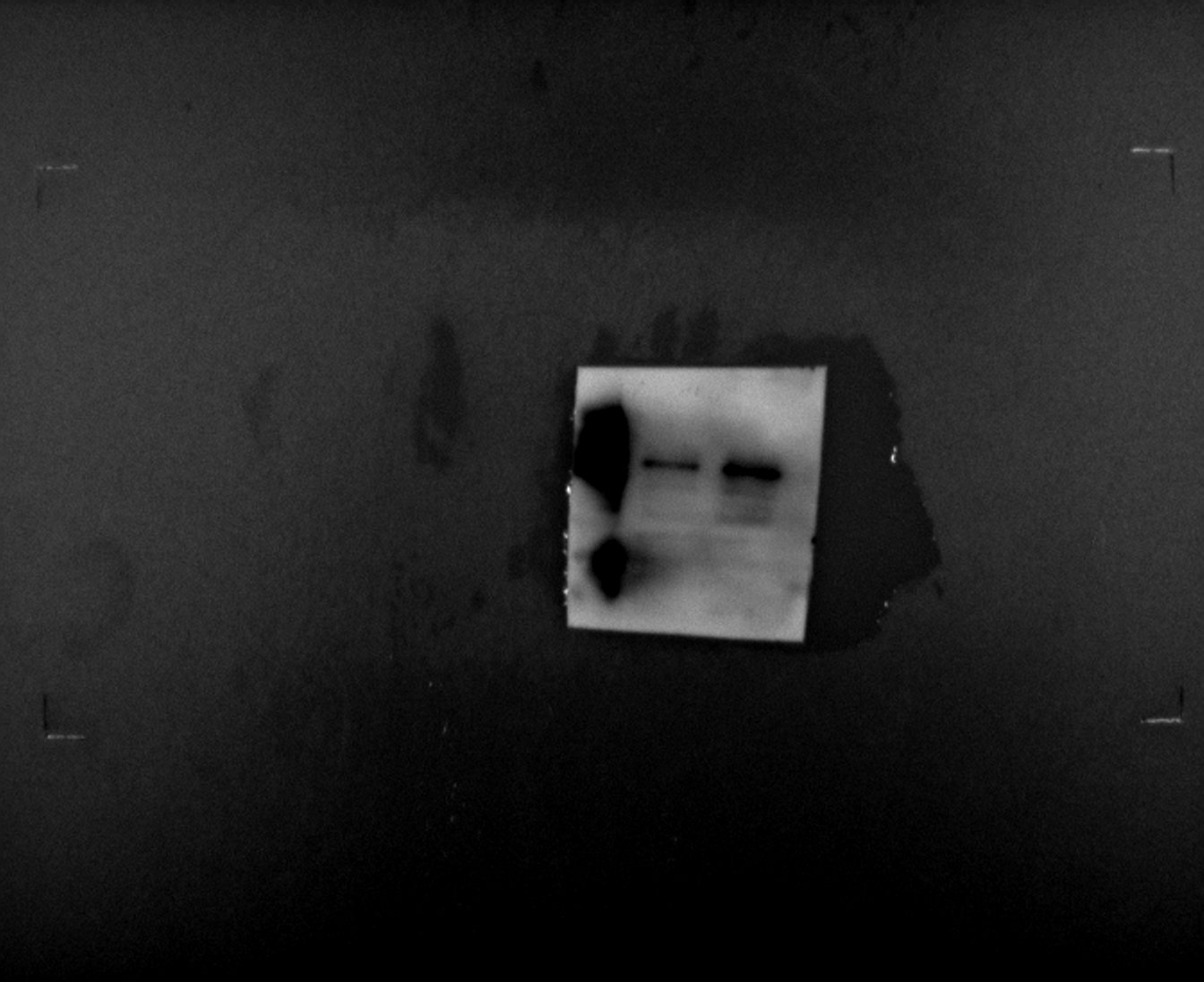

Supplement: Supplemental Information 2 [file peerj-12-17874-s002.zip › fig 1G/DUOX2-3 (1).tif]

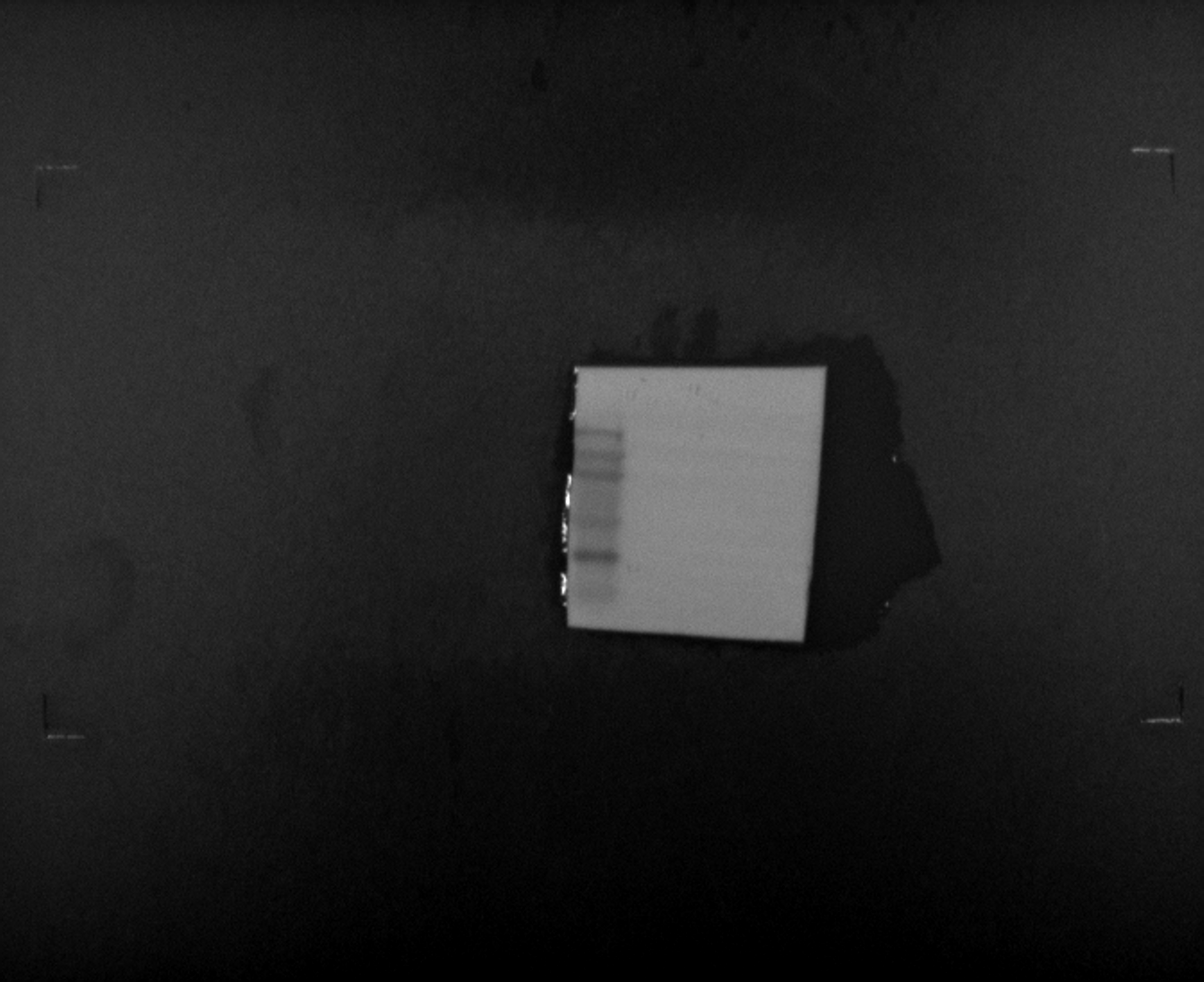

Supplement: Supplemental Information 2 [file peerj-12-17874-s002.zip › fig 1G/DUOX2-3 (2).tif]

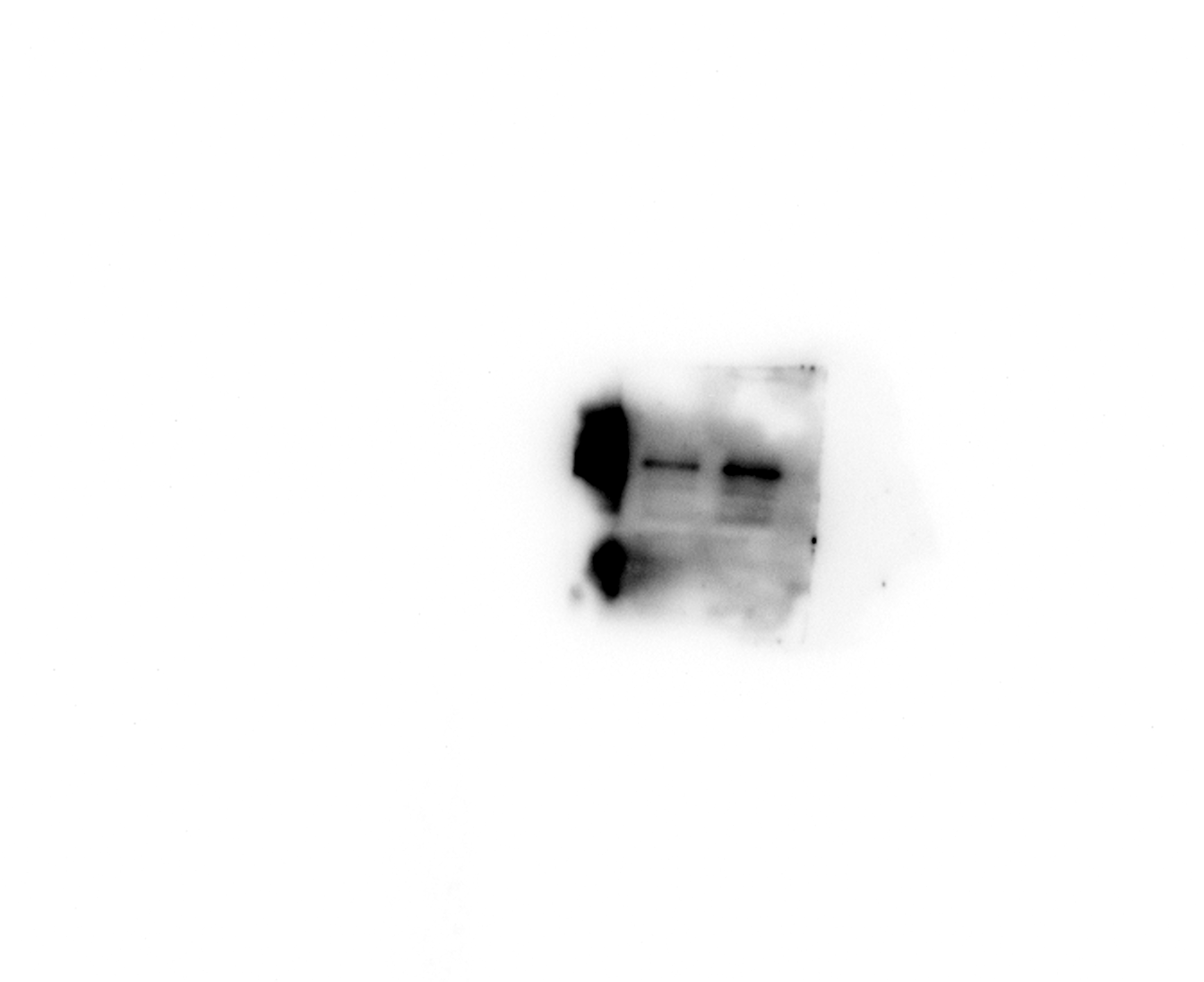

Supplement: Supplemental Information 2 [file peerj-12-17874-s002.zip › fig 1G/DUOX2-3 (3).tif]

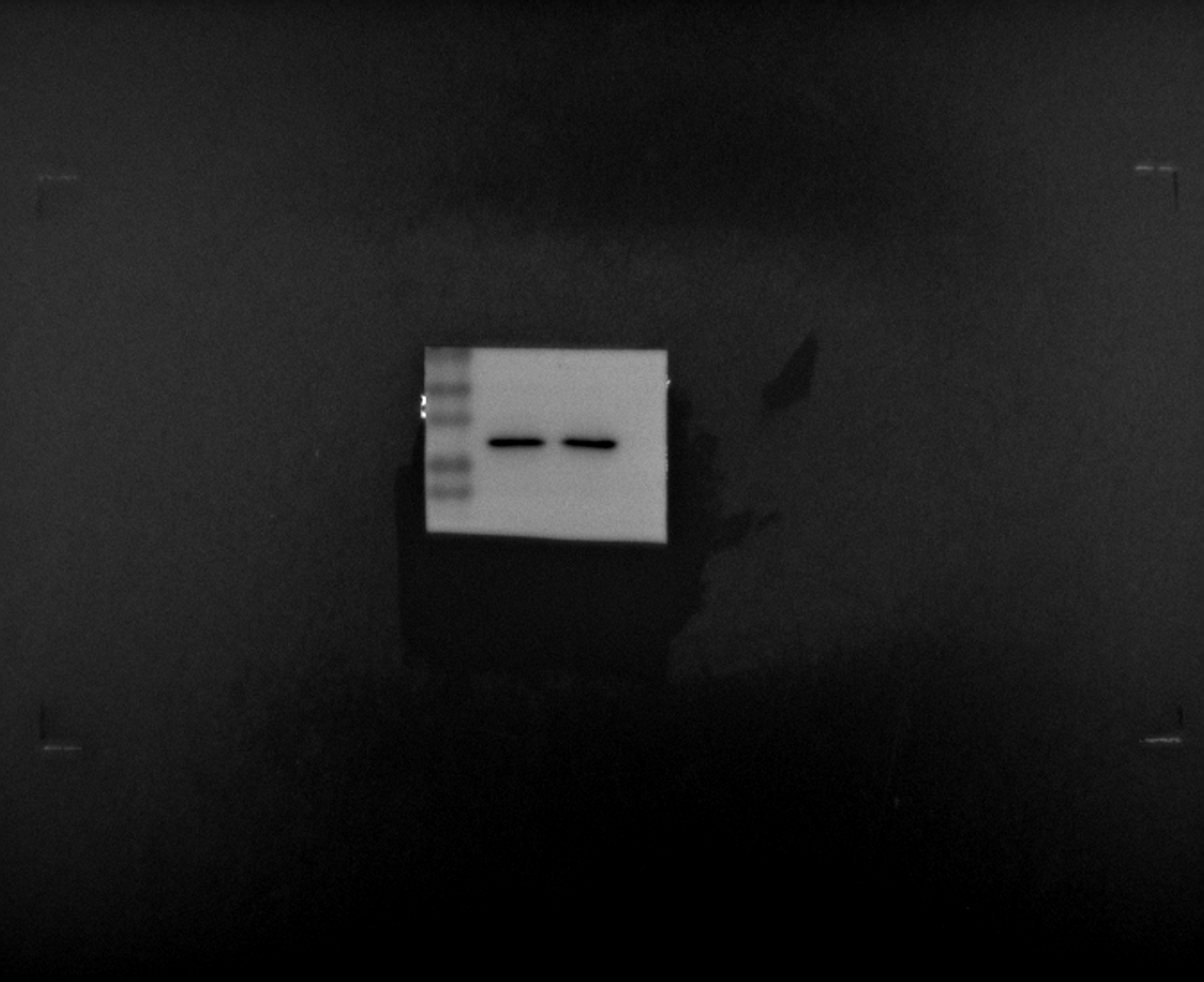

Supplement: Supplemental Information 2 [file peerj-12-17874-s002.zip › fig 1G/GAPDH (1).tif]

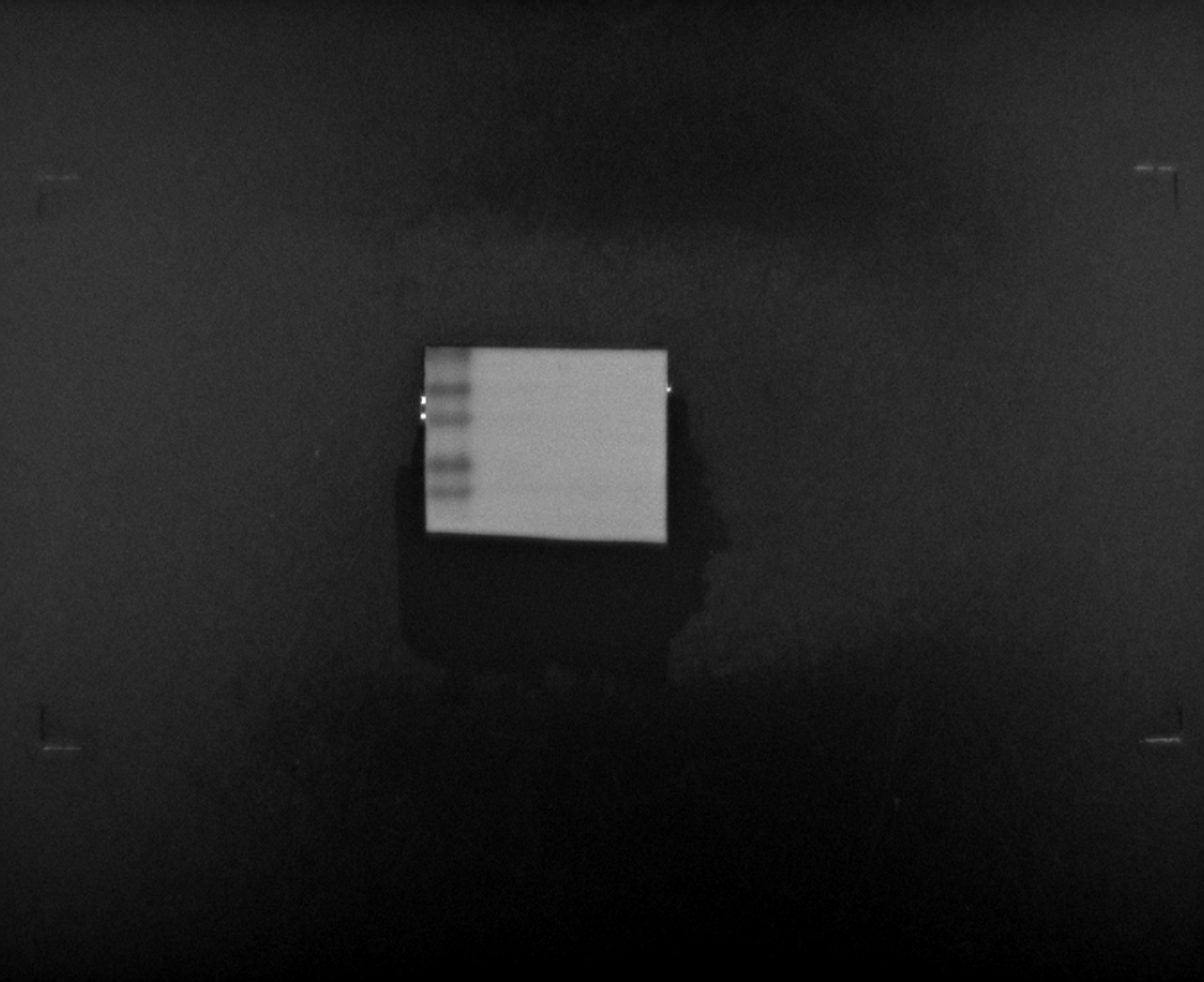

Supplement: Supplemental Information 2 [file peerj-12-17874-s002.zip › fig 1G/GAPDH (2).tif]

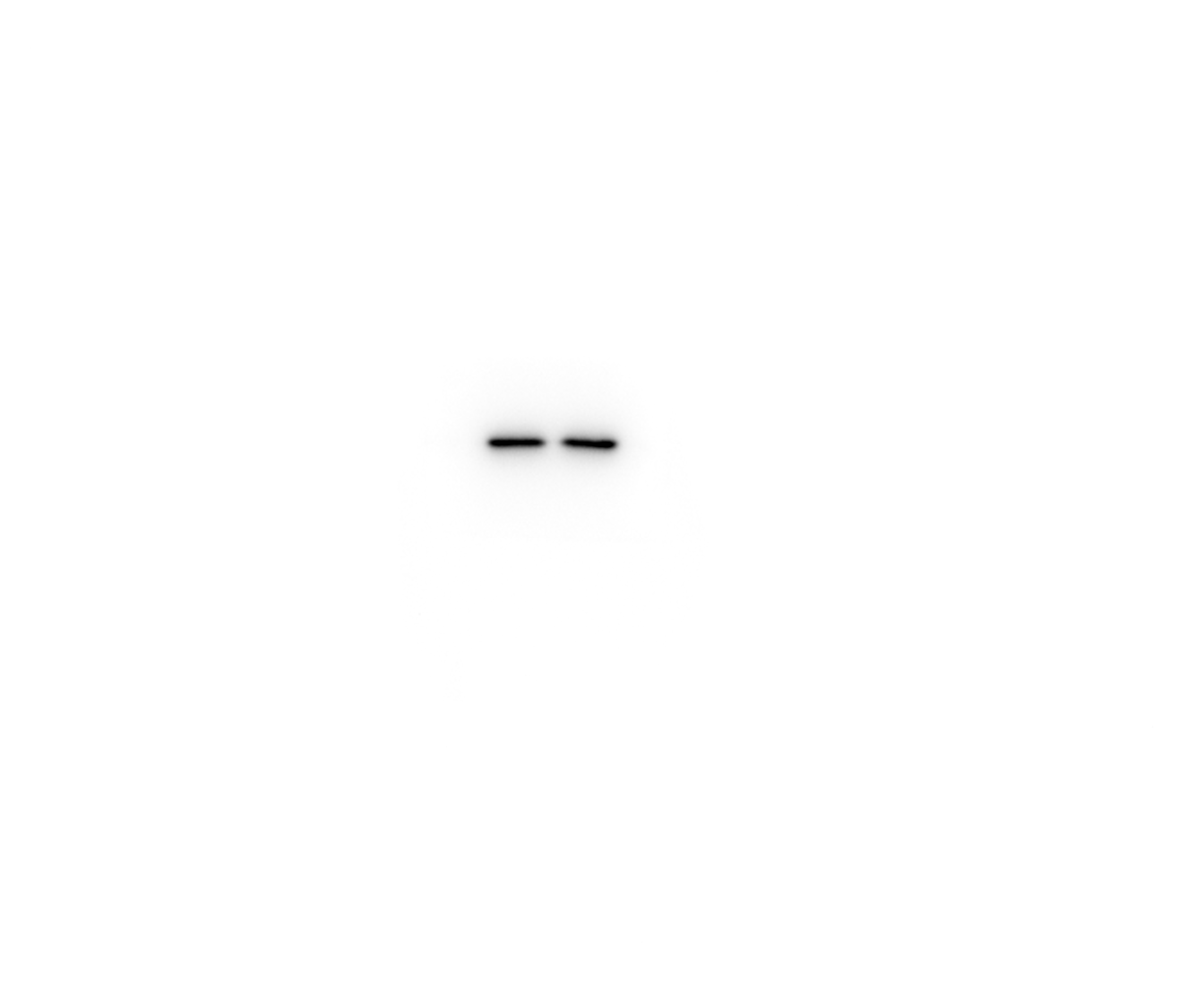

Supplement: Supplemental Information 2 [file peerj-12-17874-s002.zip › fig 1G/GAPDH (3).tif]

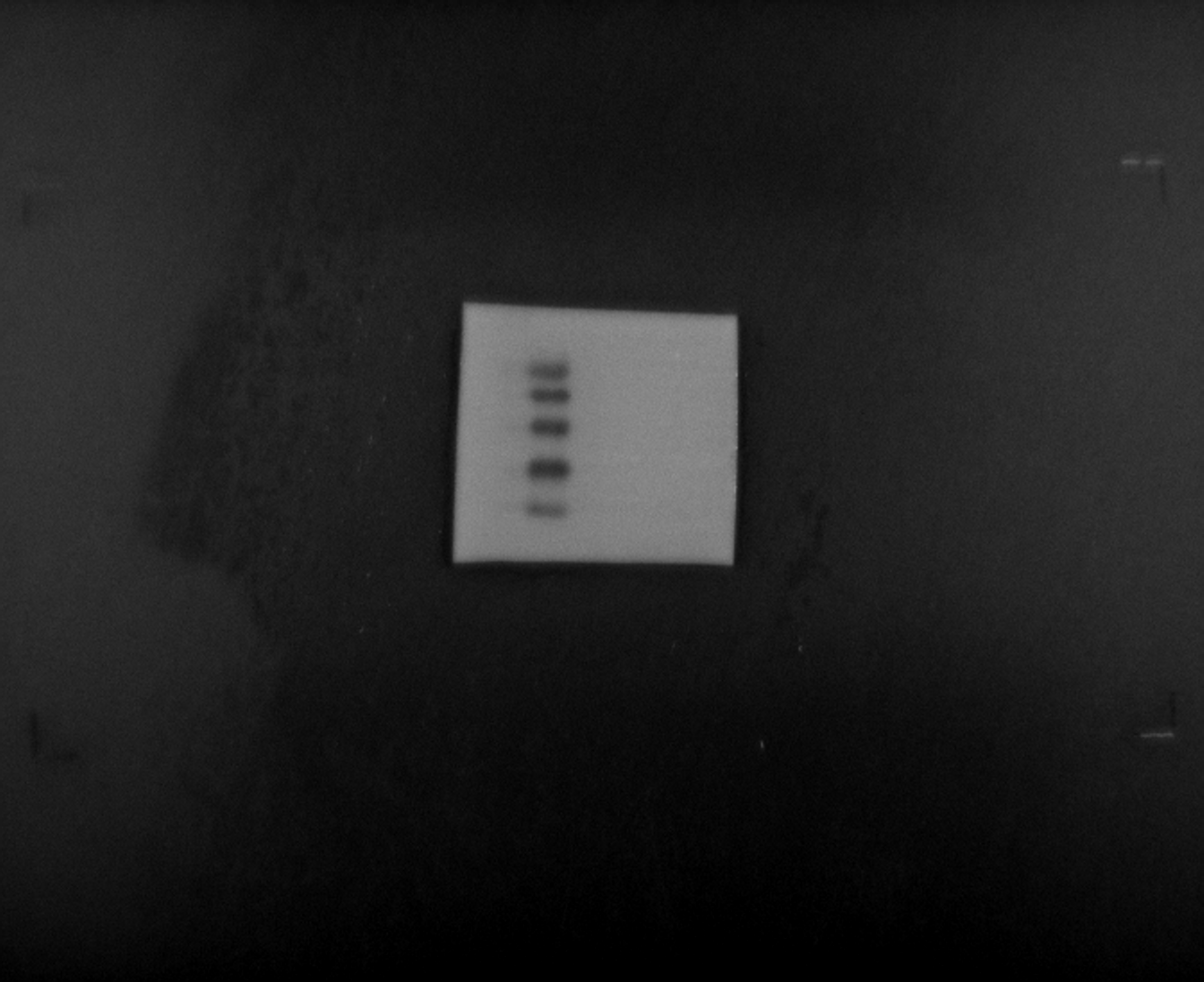

Supplement: Supplemental Information 2 [file peerj-12-17874-s002.zip › fig 1G/GAPDH-2 (1).tif]

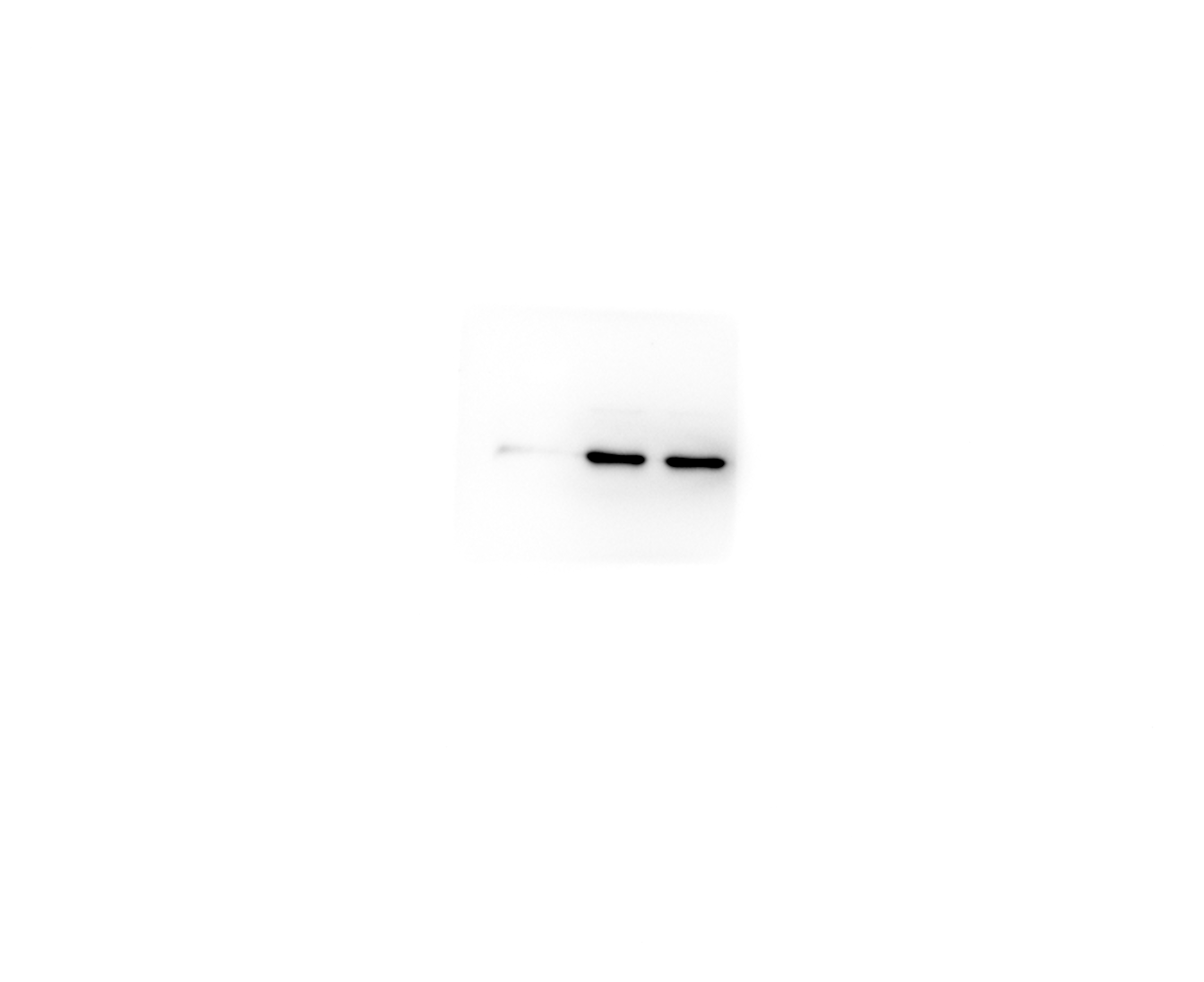

Supplement: Supplemental Information 2 [file peerj-12-17874-s002.zip › fig 1G/GAPDH-2 (2).tif]

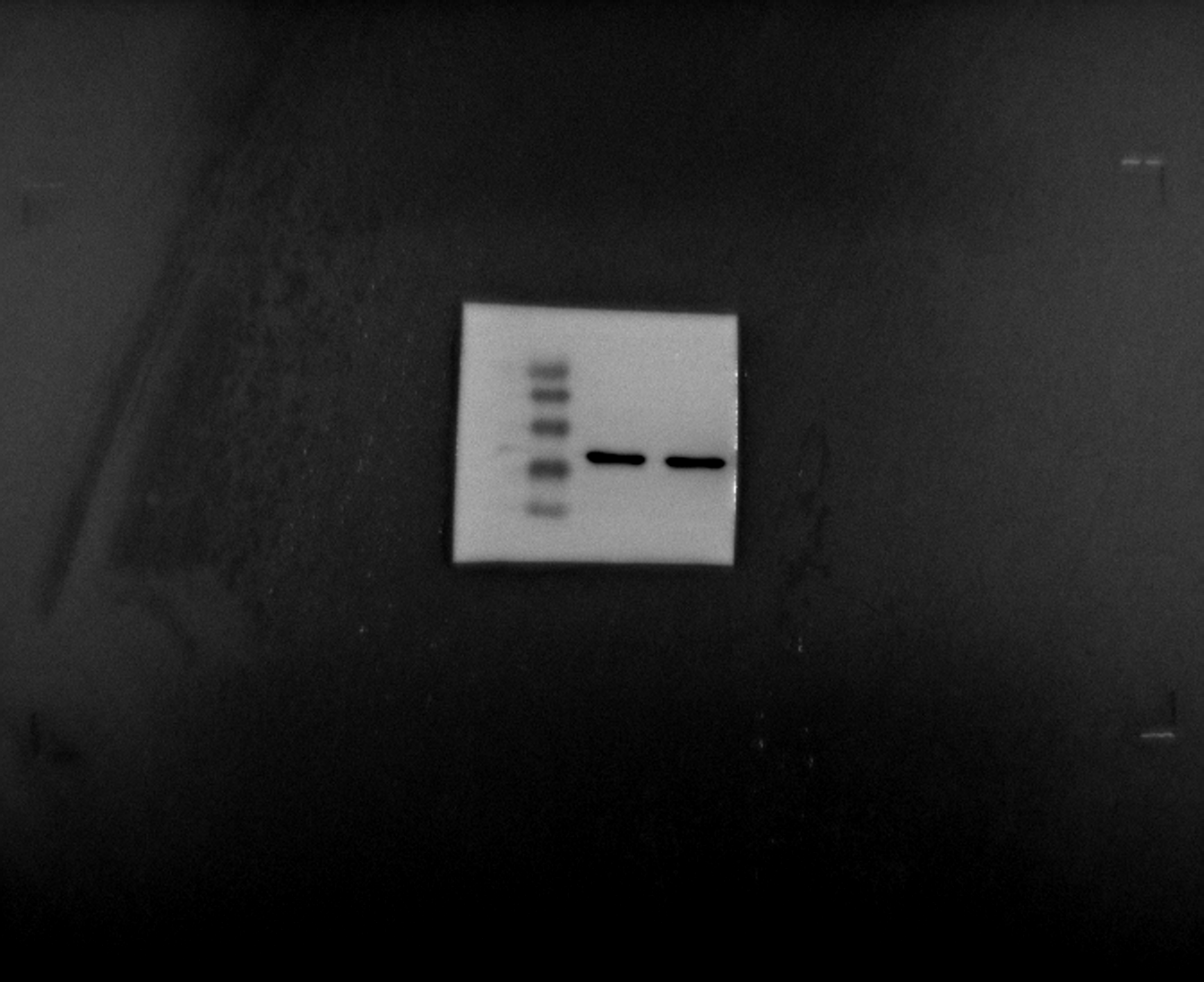

Supplement: Supplemental Information 2 [file peerj-12-17874-s002.zip › fig 1G/GAPDH-2 (3).tif]

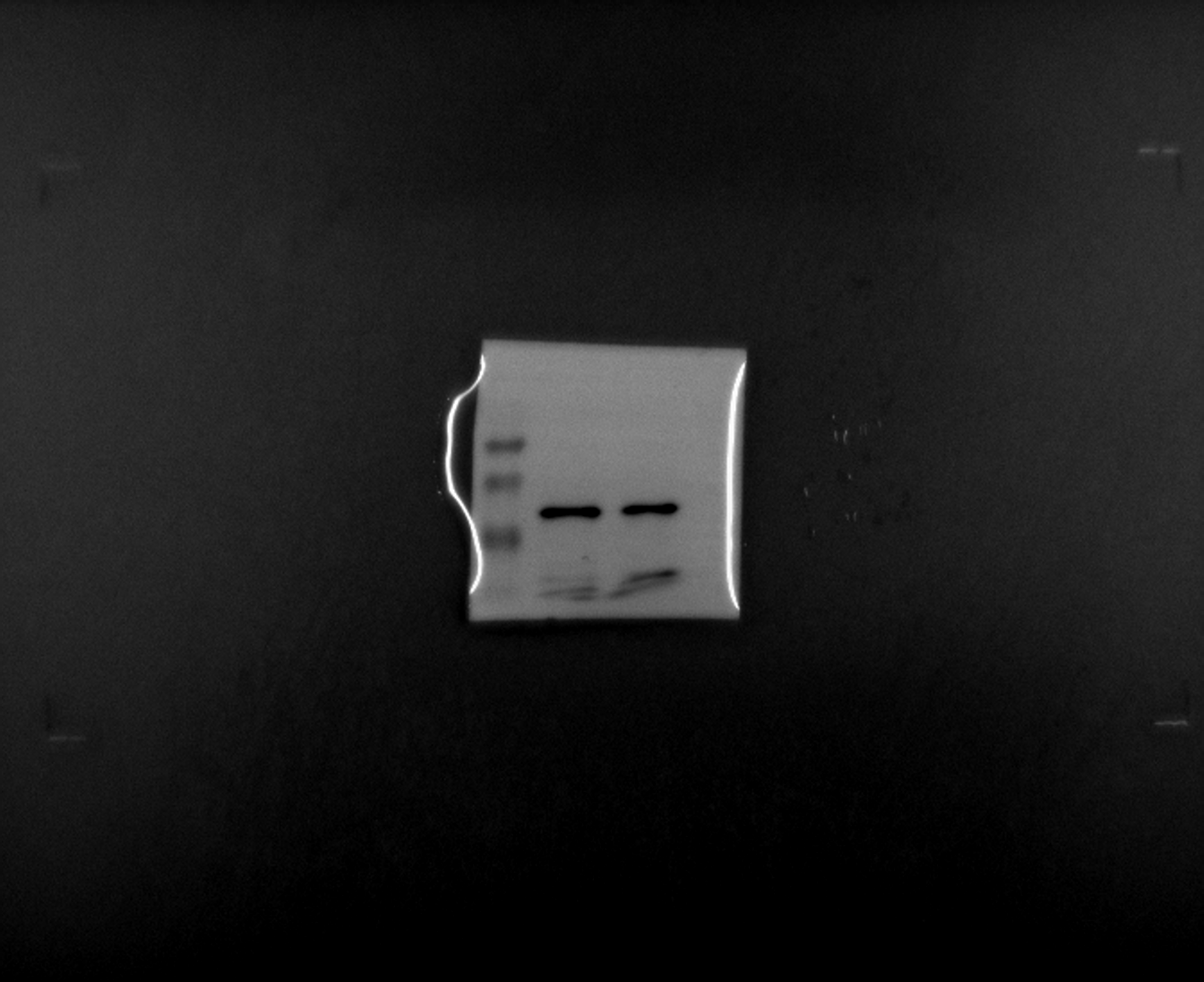

Supplement: Supplemental Information 2 [file peerj-12-17874-s002.zip › fig 1G/GAPDH-3 (1).tif]

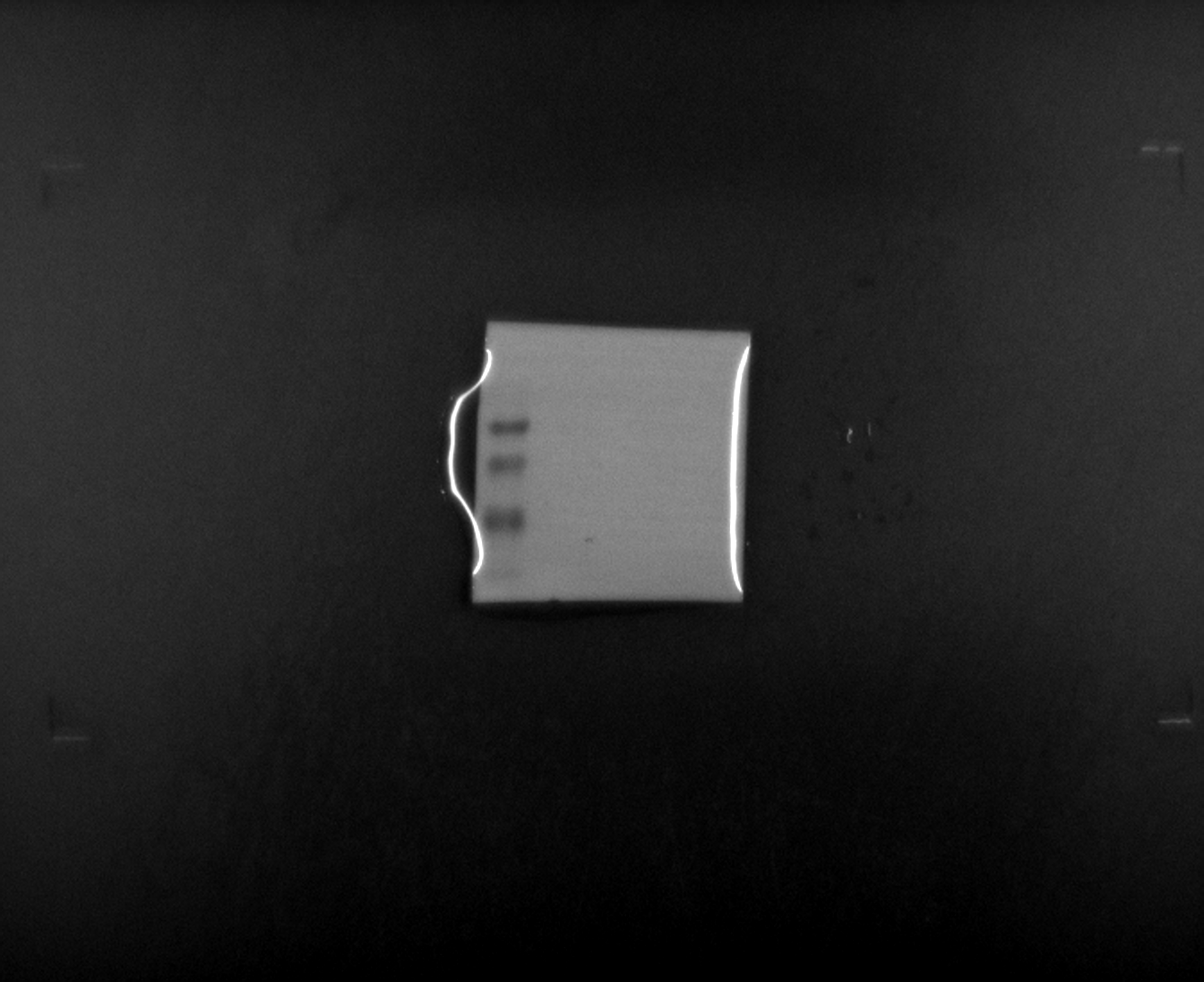

Supplement: Supplemental Information 2 [file peerj-12-17874-s002.zip › fig 1G/GAPDH-3 (2).tif]

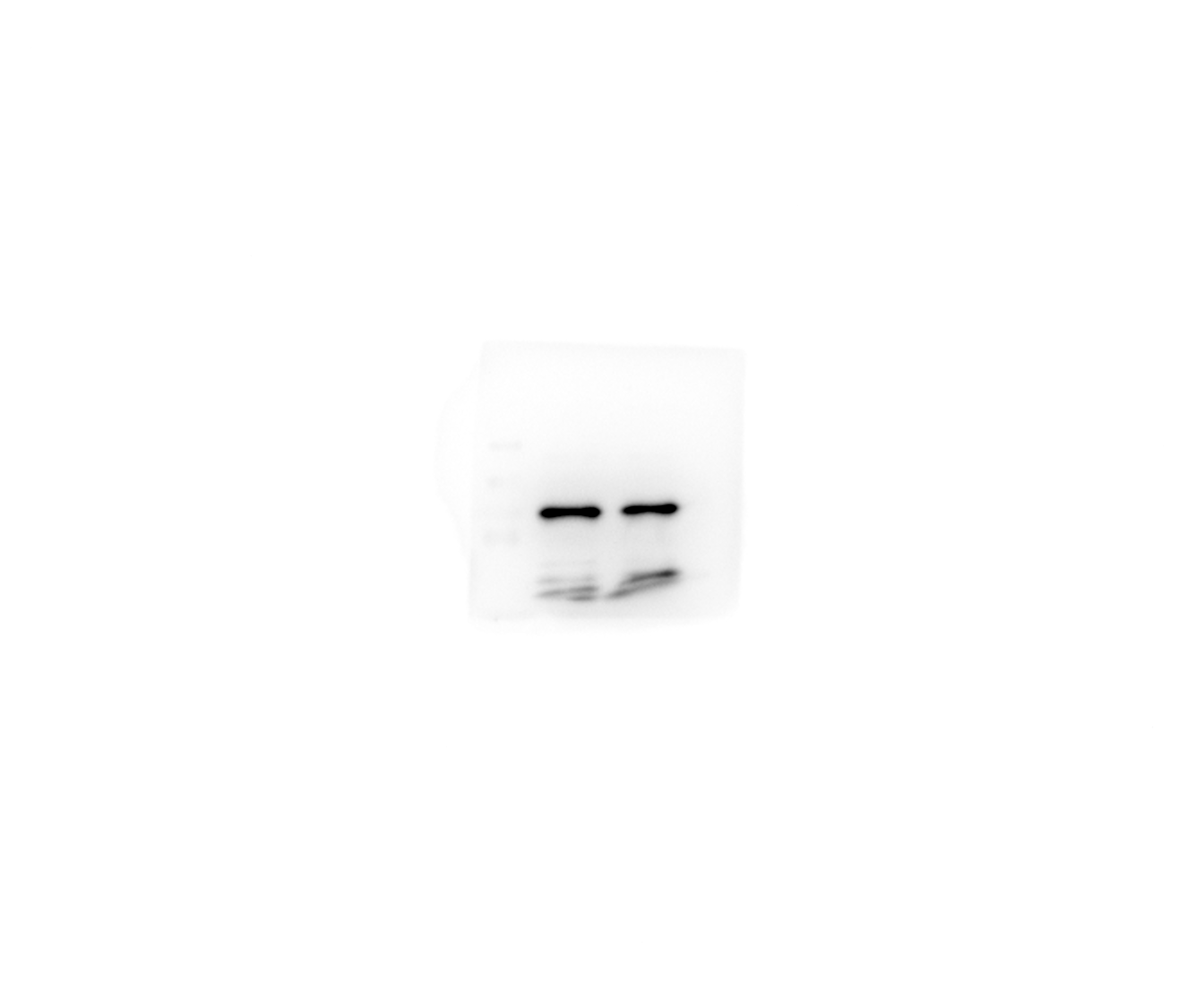

Supplement: Supplemental Information 2 [file peerj-12-17874-s002.zip › fig 1G/GAPDH-3 (3).tif]

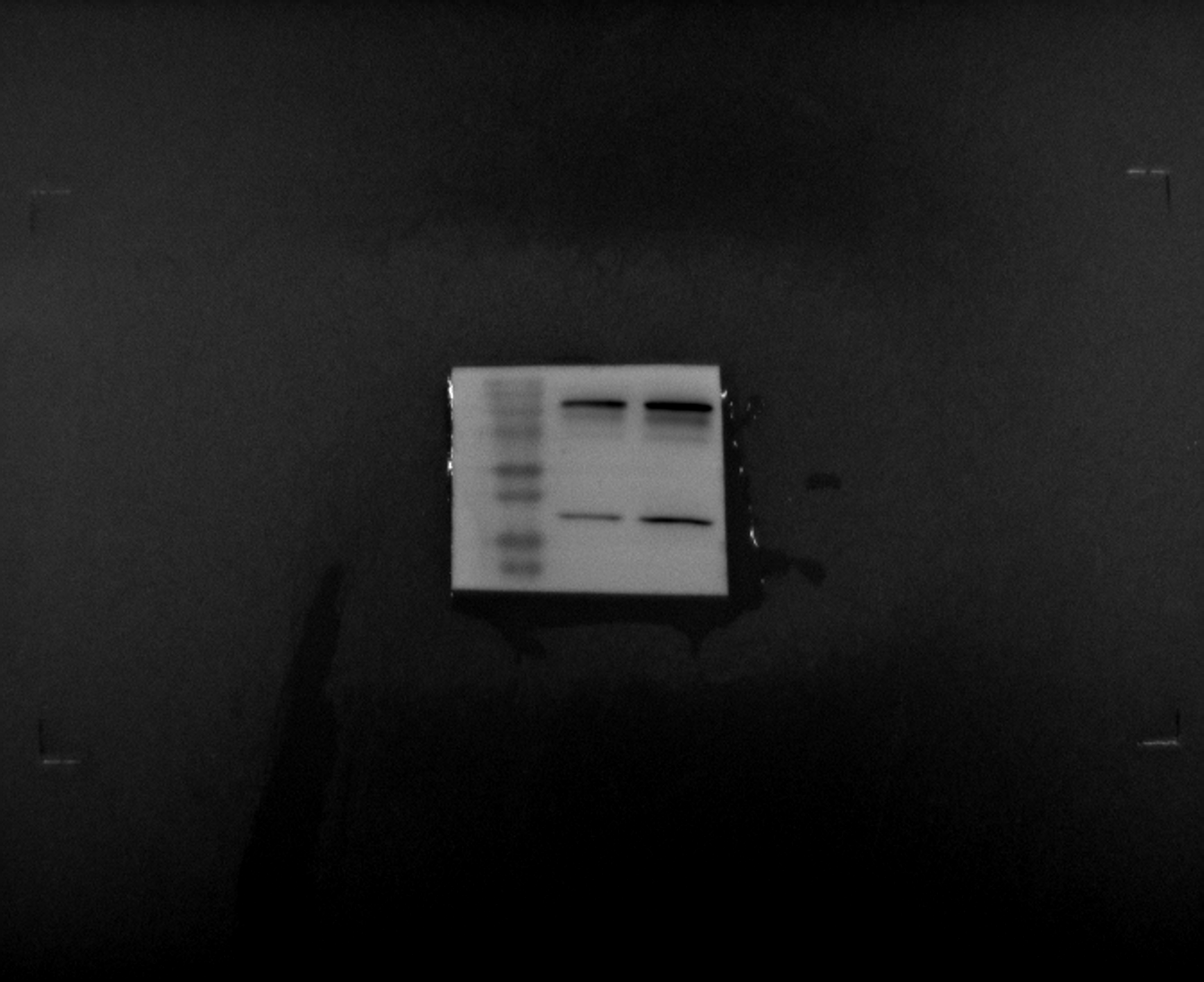

Supplement: Supplemental Information 2 [file peerj-12-17874-s002.zip › fig 1G/nlrp3 (1).tif]

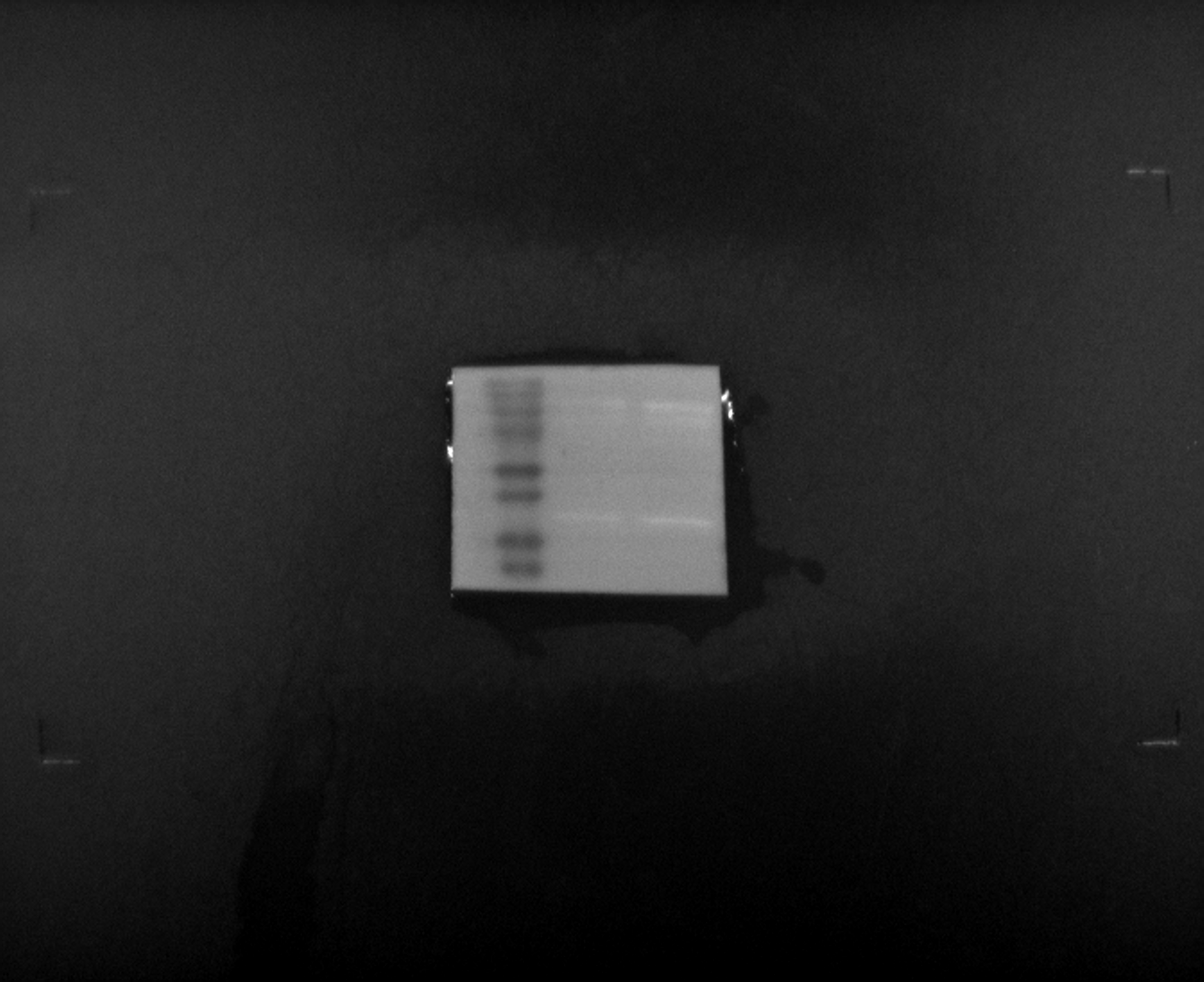

Supplement: Supplemental Information 2 [file peerj-12-17874-s002.zip › fig 1G/nlrp3 (2).tif]

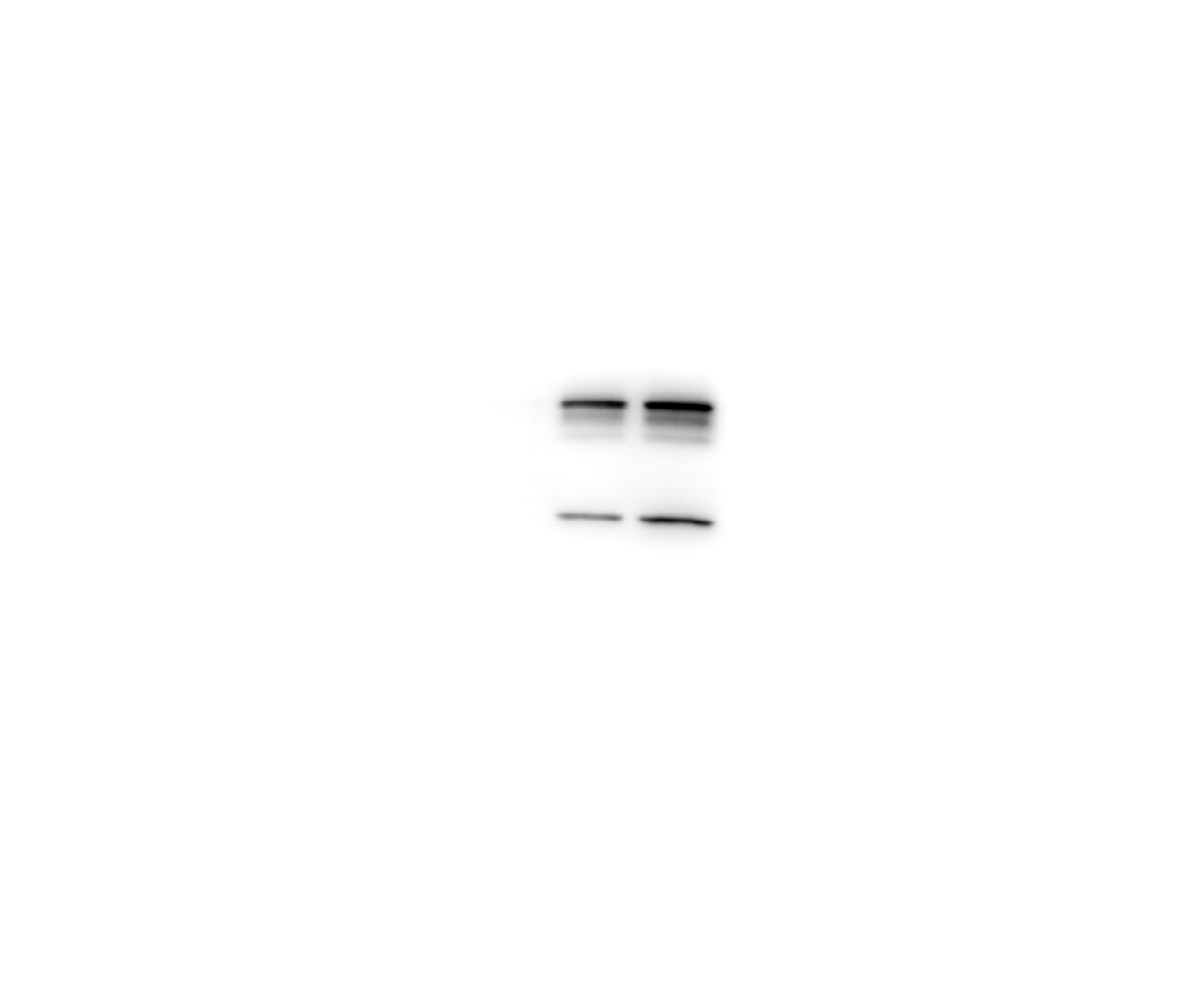

Supplement: Supplemental Information 2 [file peerj-12-17874-s002.zip › fig 1G/nlrp3 (3).tif]

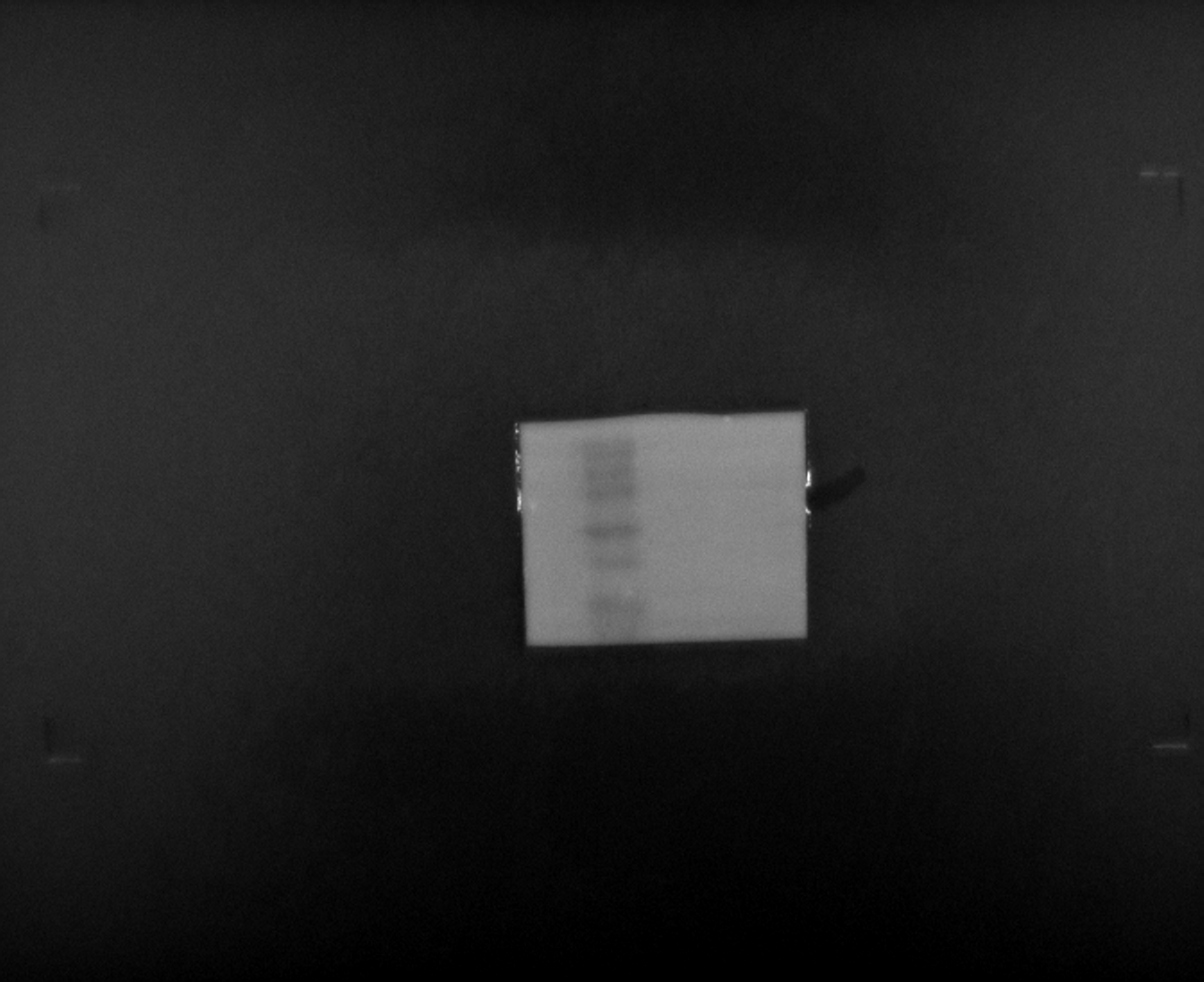

Supplement: Supplemental Information 2 [file peerj-12-17874-s002.zip › fig 1G/NRLP3-2 (1).tif]

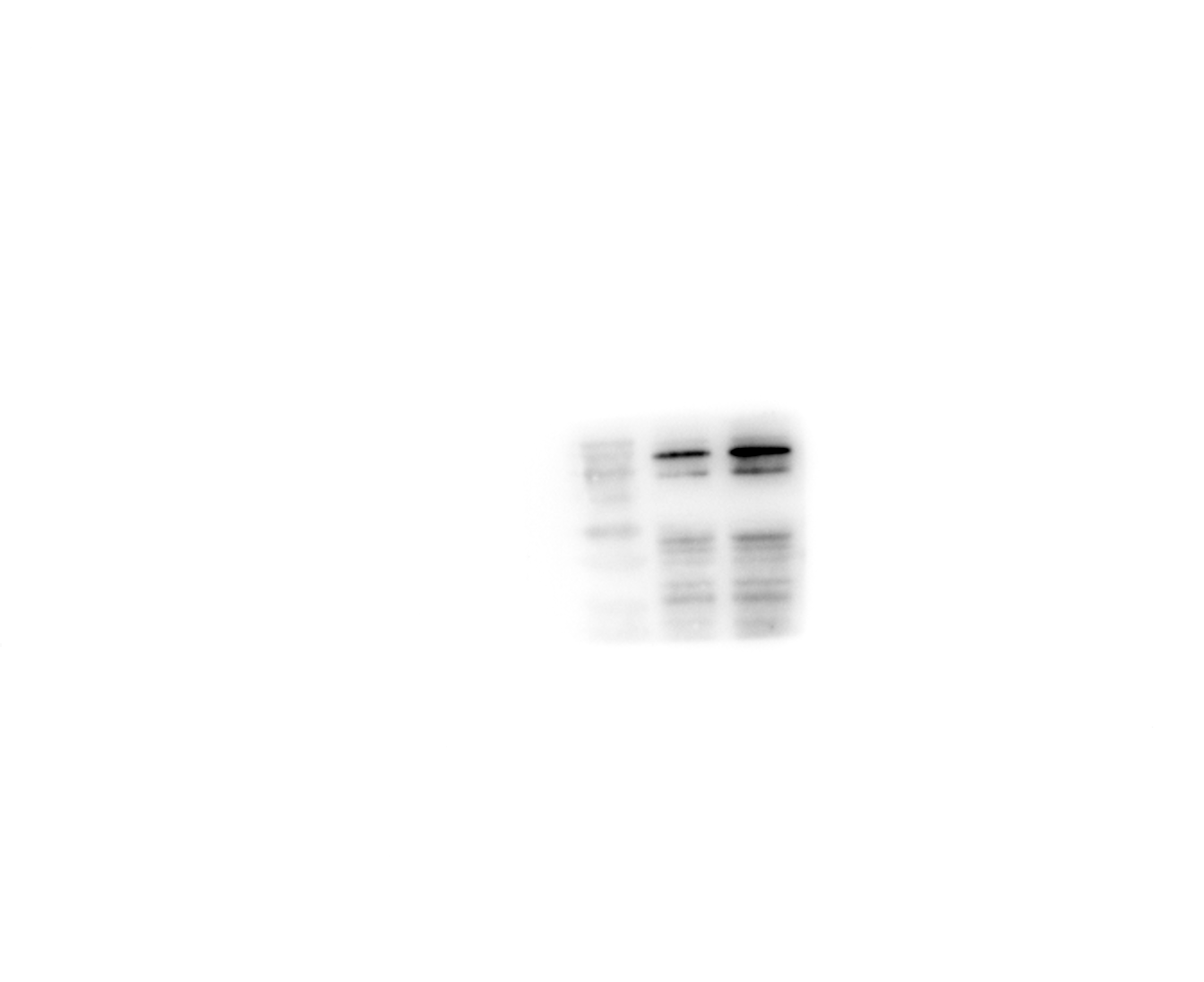

Supplement: Supplemental Information 2 [file peerj-12-17874-s002.zip › fig 1G/NRLP3-2 (2).tif]

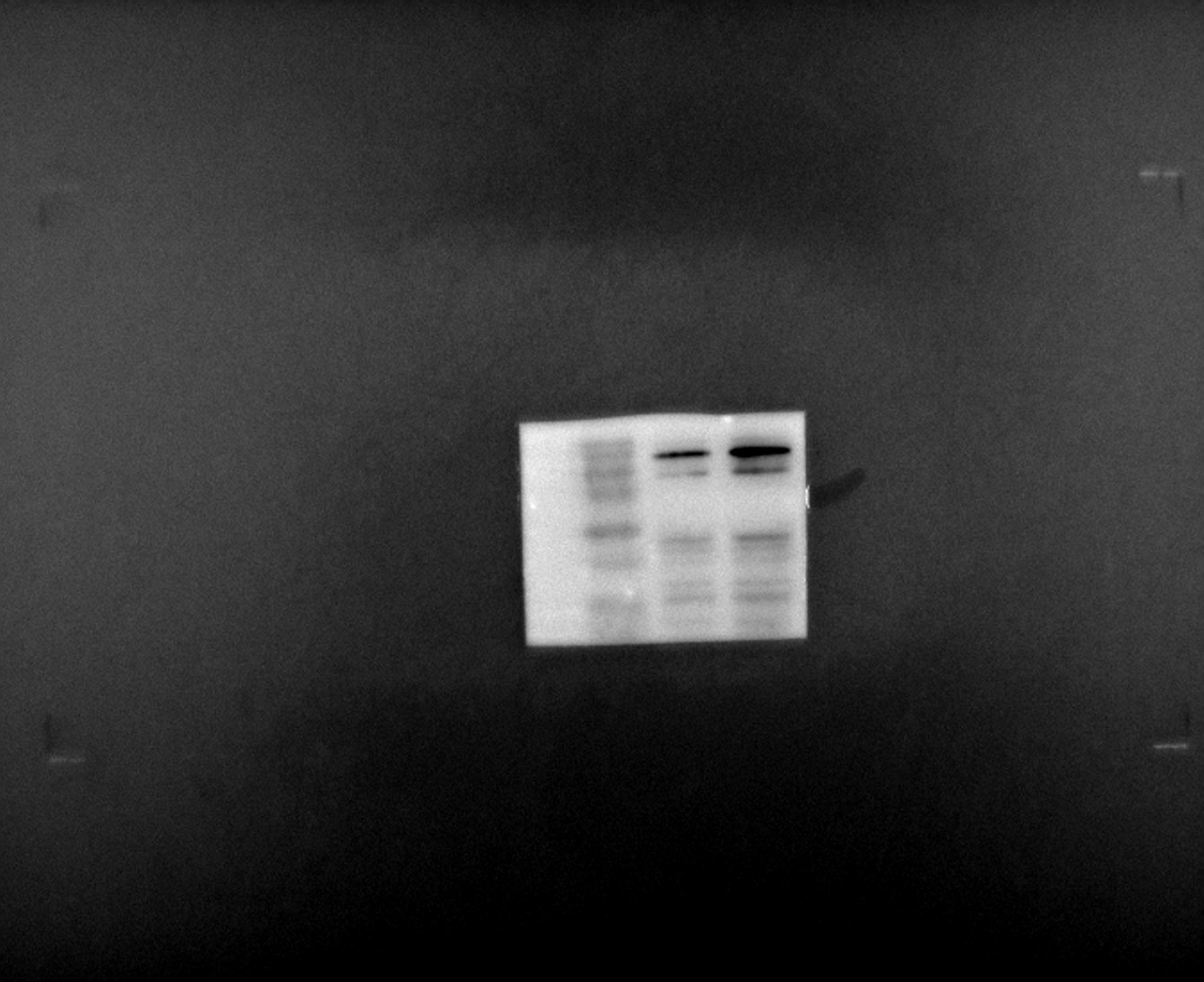

Supplement: Supplemental Information 2 [file peerj-12-17874-s002.zip › fig 1G/NRLP3-2 (3).tif]

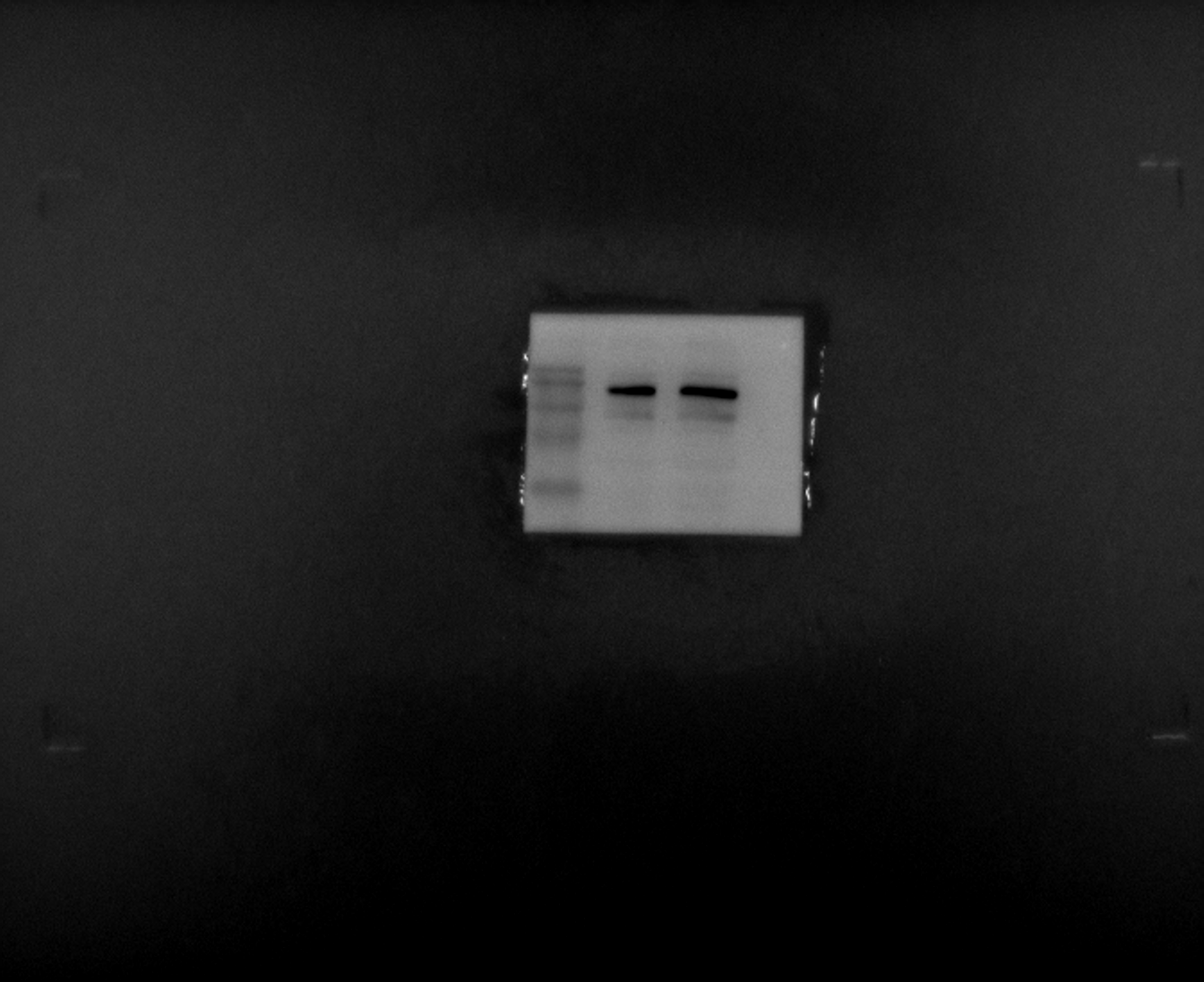

Supplement: Supplemental Information 2 [file peerj-12-17874-s002.zip › fig 1G/NRLP3-3 (1).tif]

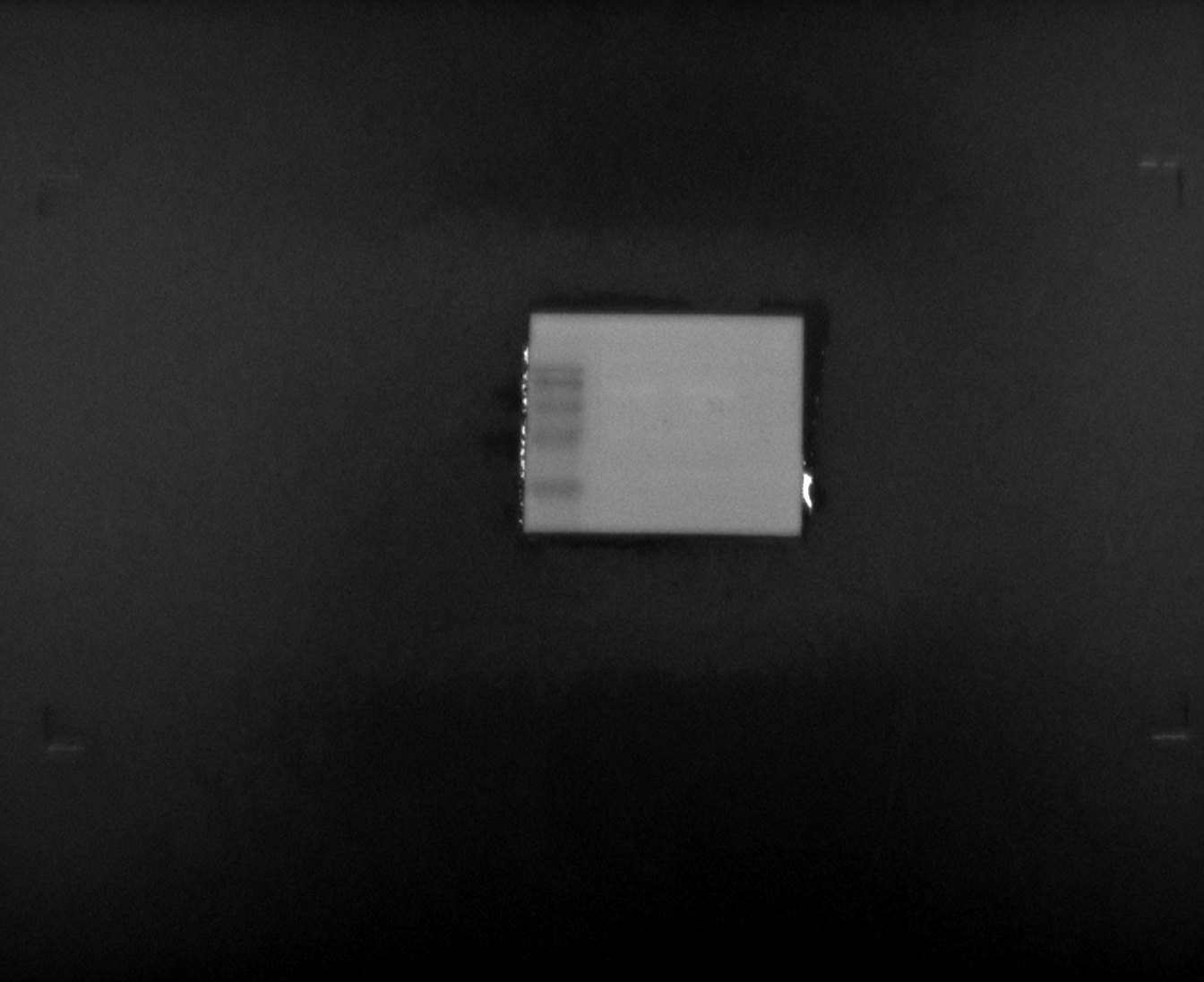

Supplement: Supplemental Information 2 [file peerj-12-17874-s002.zip › fig 1G/NRLP3-3 (2).tif]

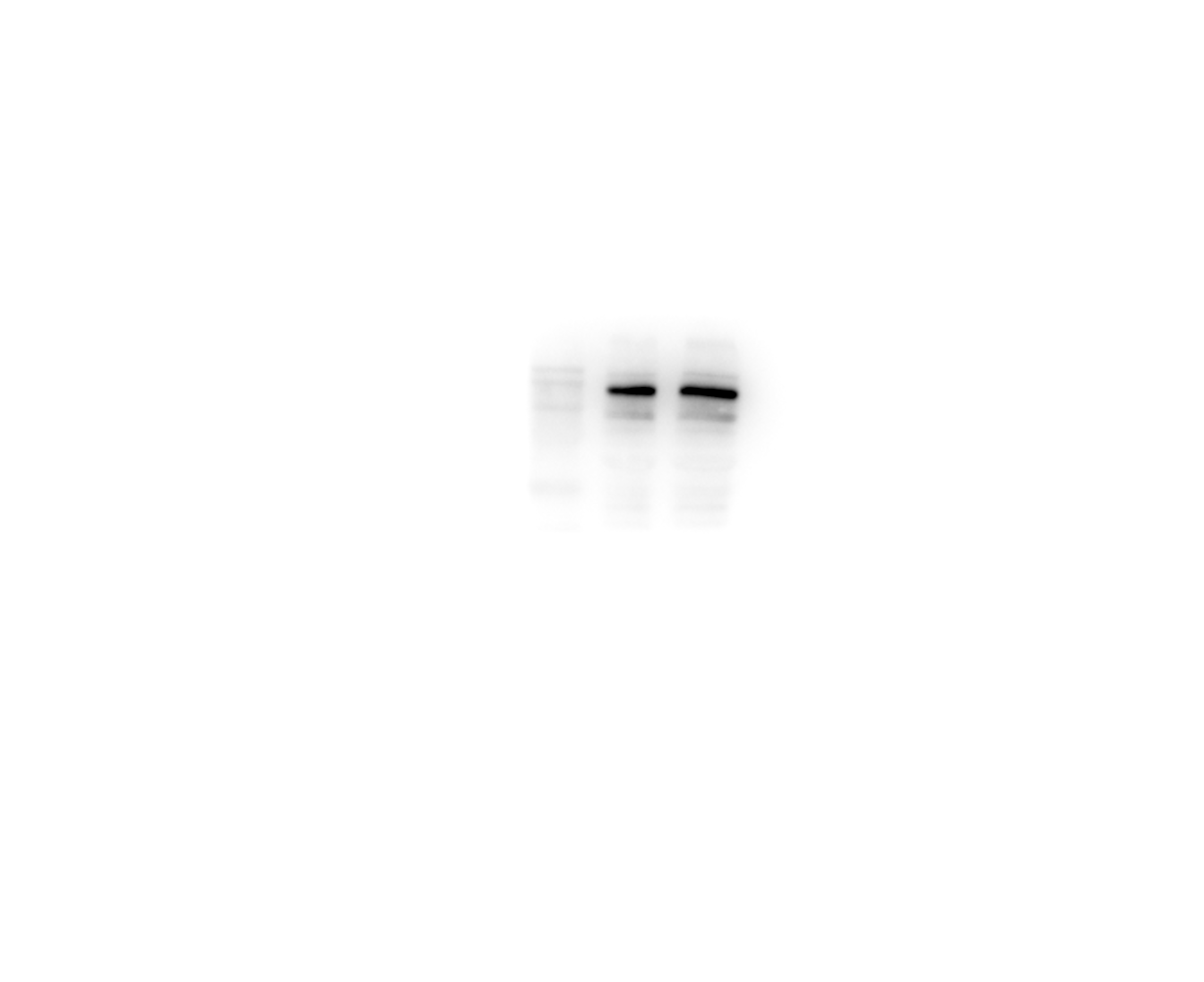

Supplement: Supplemental Information 2 [file peerj-12-17874-s002.zip › fig 1G/NRLP3-3 (3).tif]

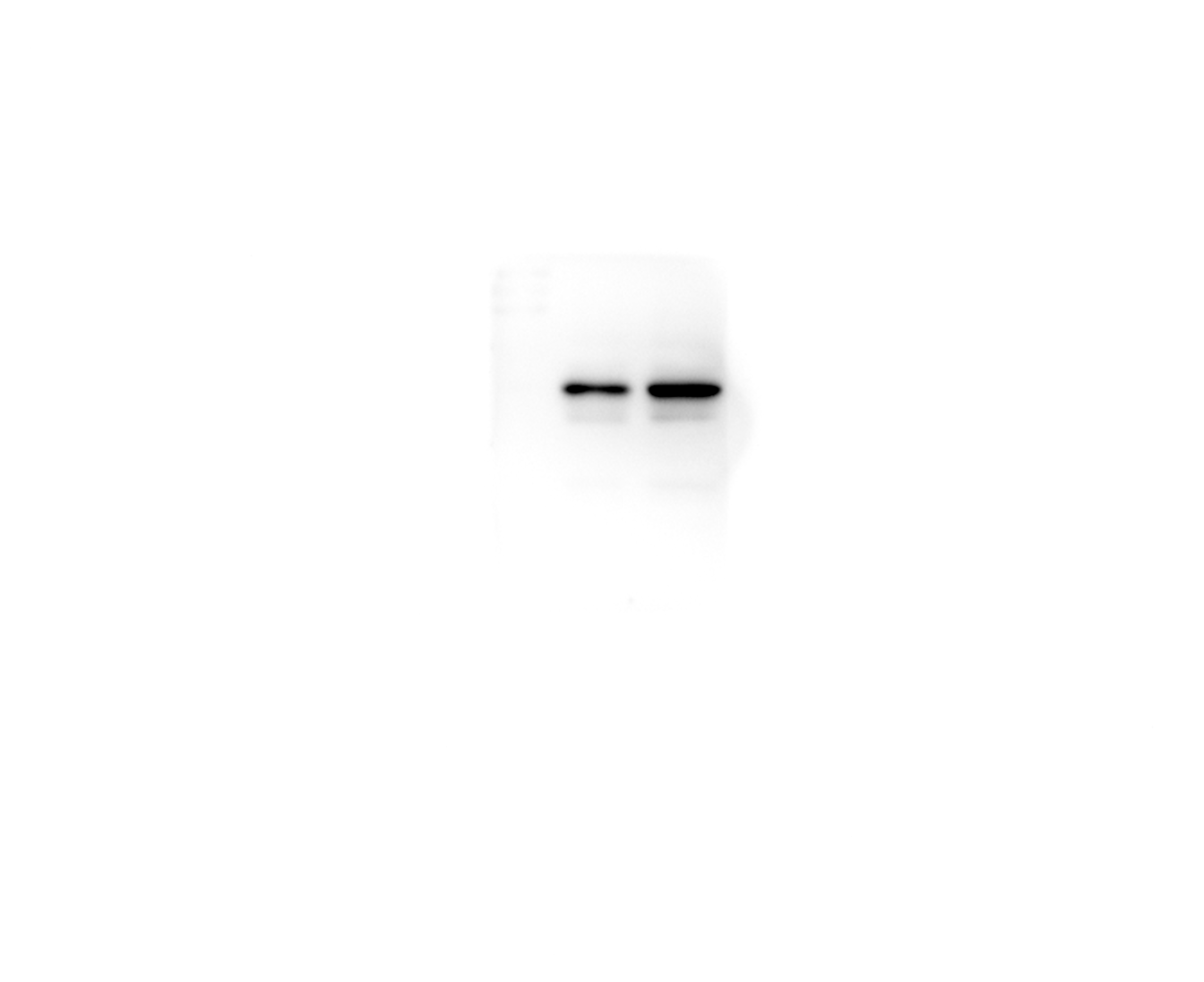

Supplement: Supplemental Information 2 [file peerj-12-17874-s002.zip › fig 1G/p-p65 (1).tif]

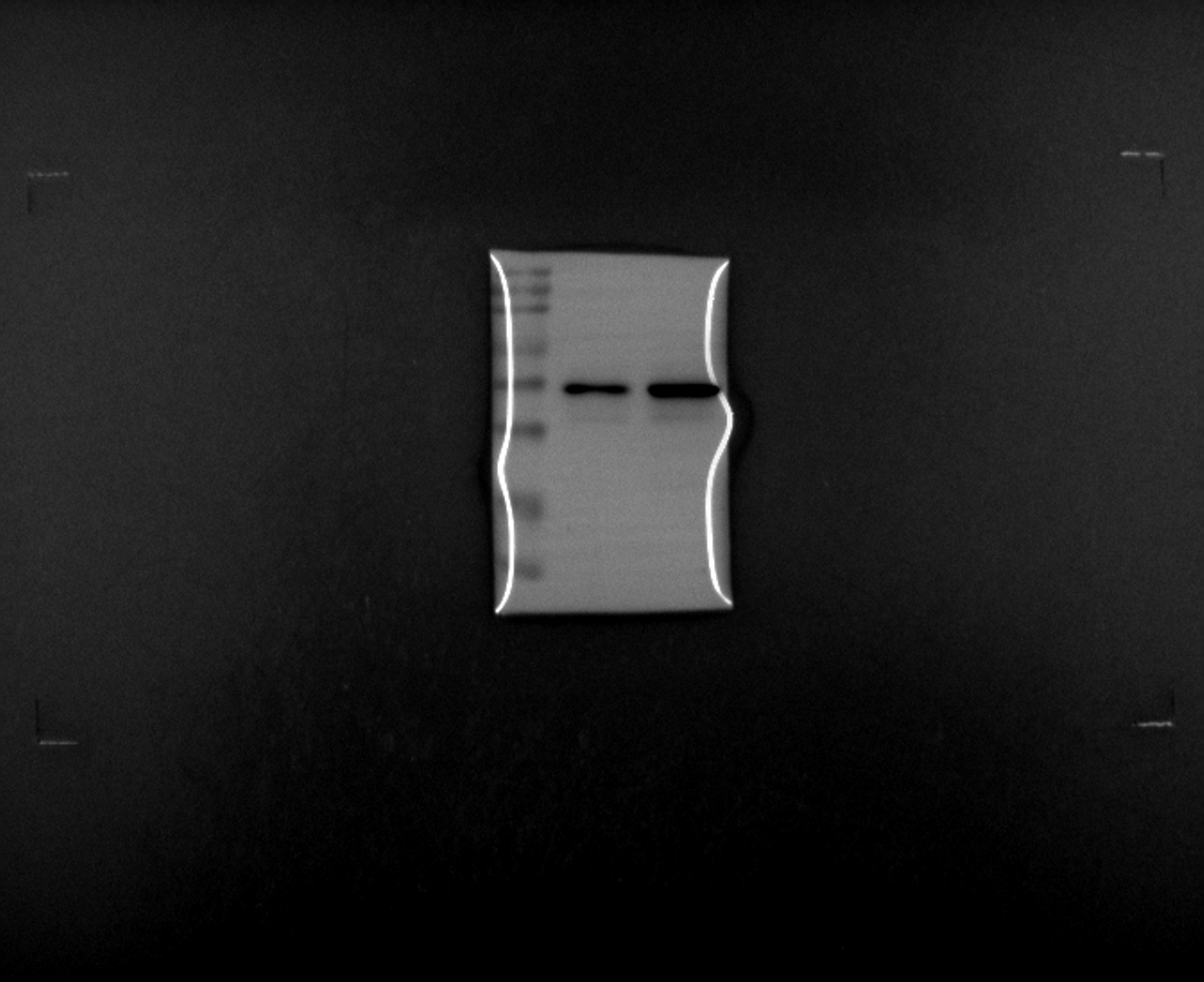

Supplement: Supplemental Information 2 [file peerj-12-17874-s002.zip › fig 1G/p-p65 (2).tif]

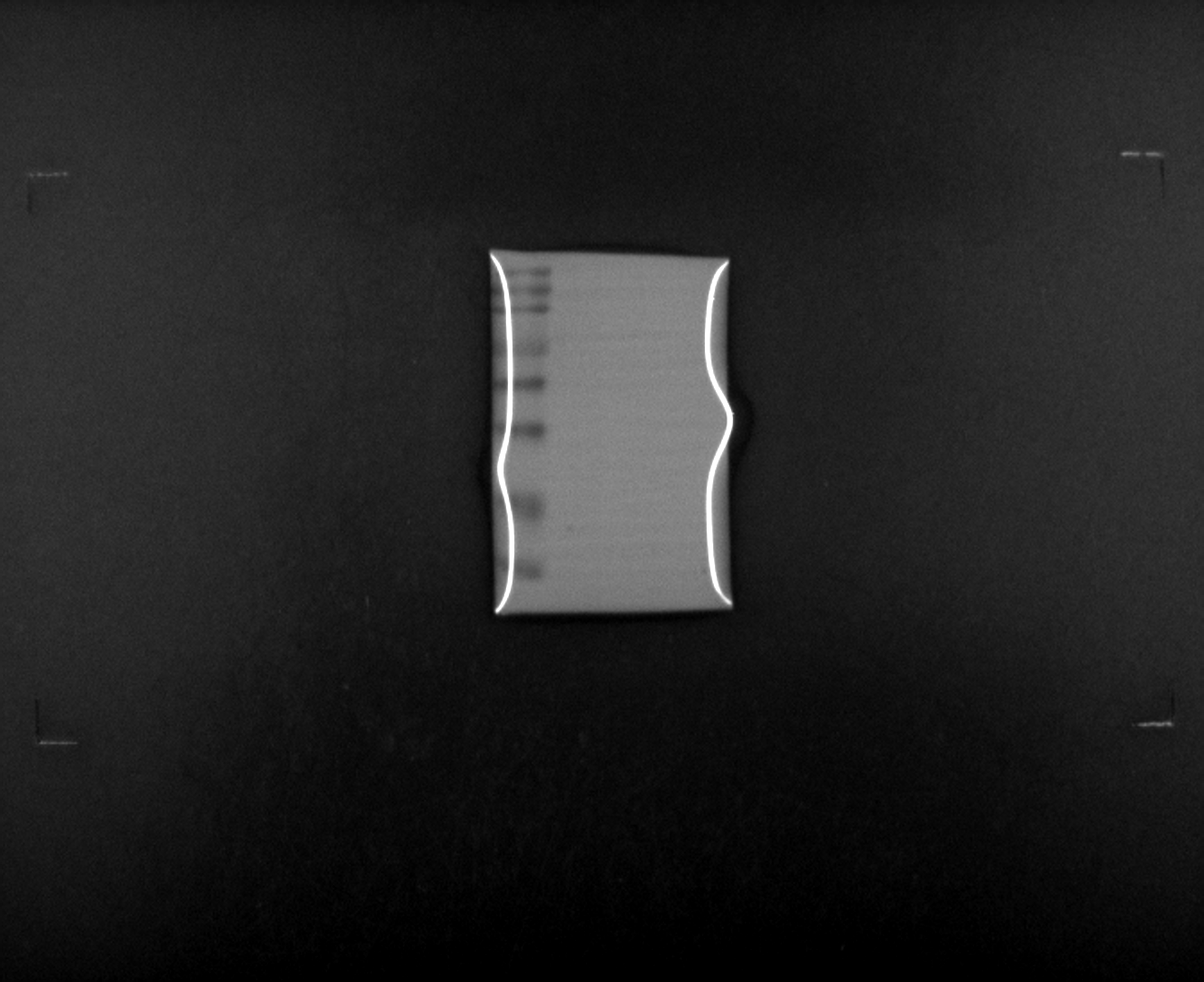

Supplement: Supplemental Information 2 [file peerj-12-17874-s002.zip › fig 1G/p-p65 (3).tif]

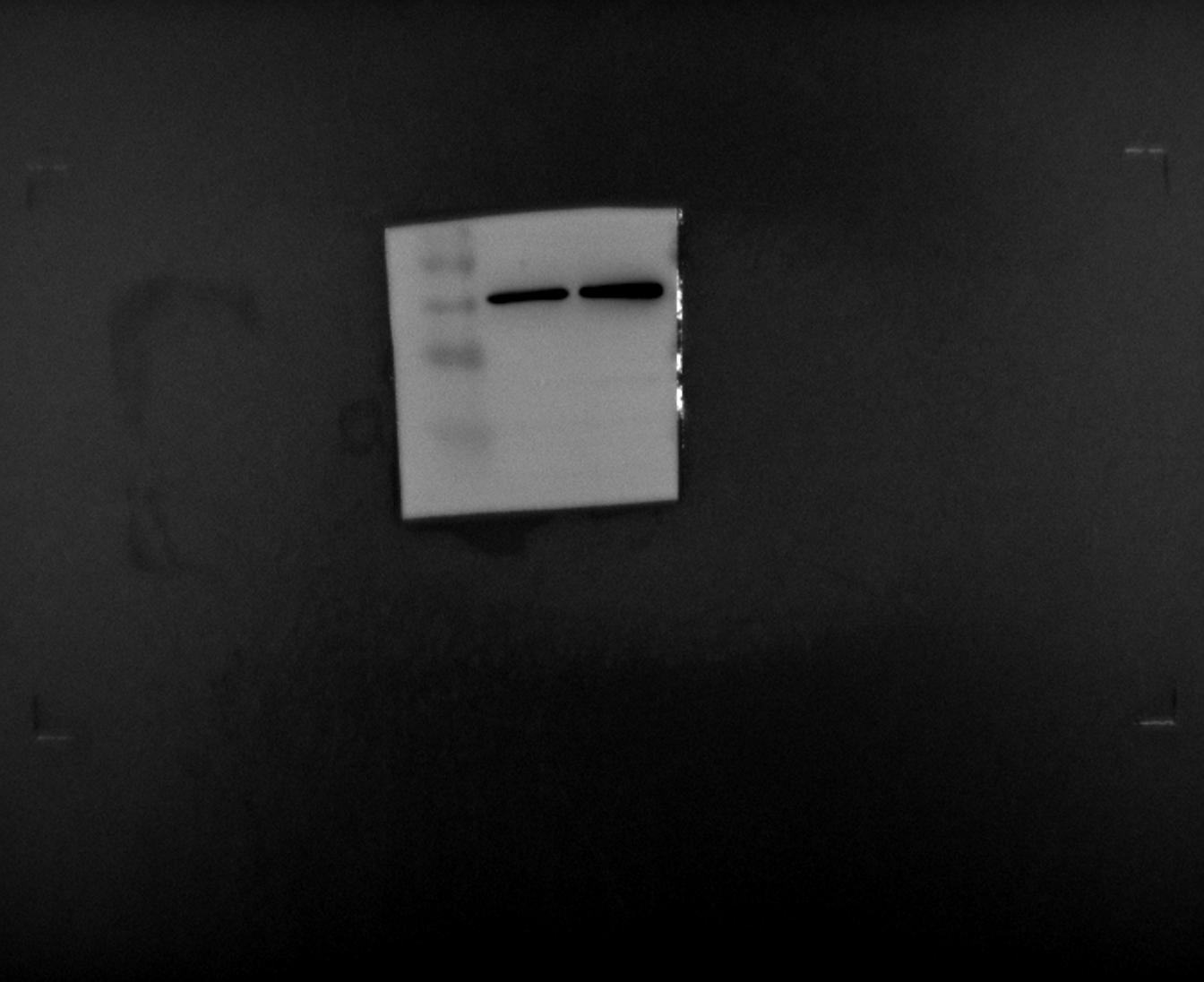

Supplement: Supplemental Information 2 [file peerj-12-17874-s002.zip › fig 1G/p-p65-1 (1).tif]

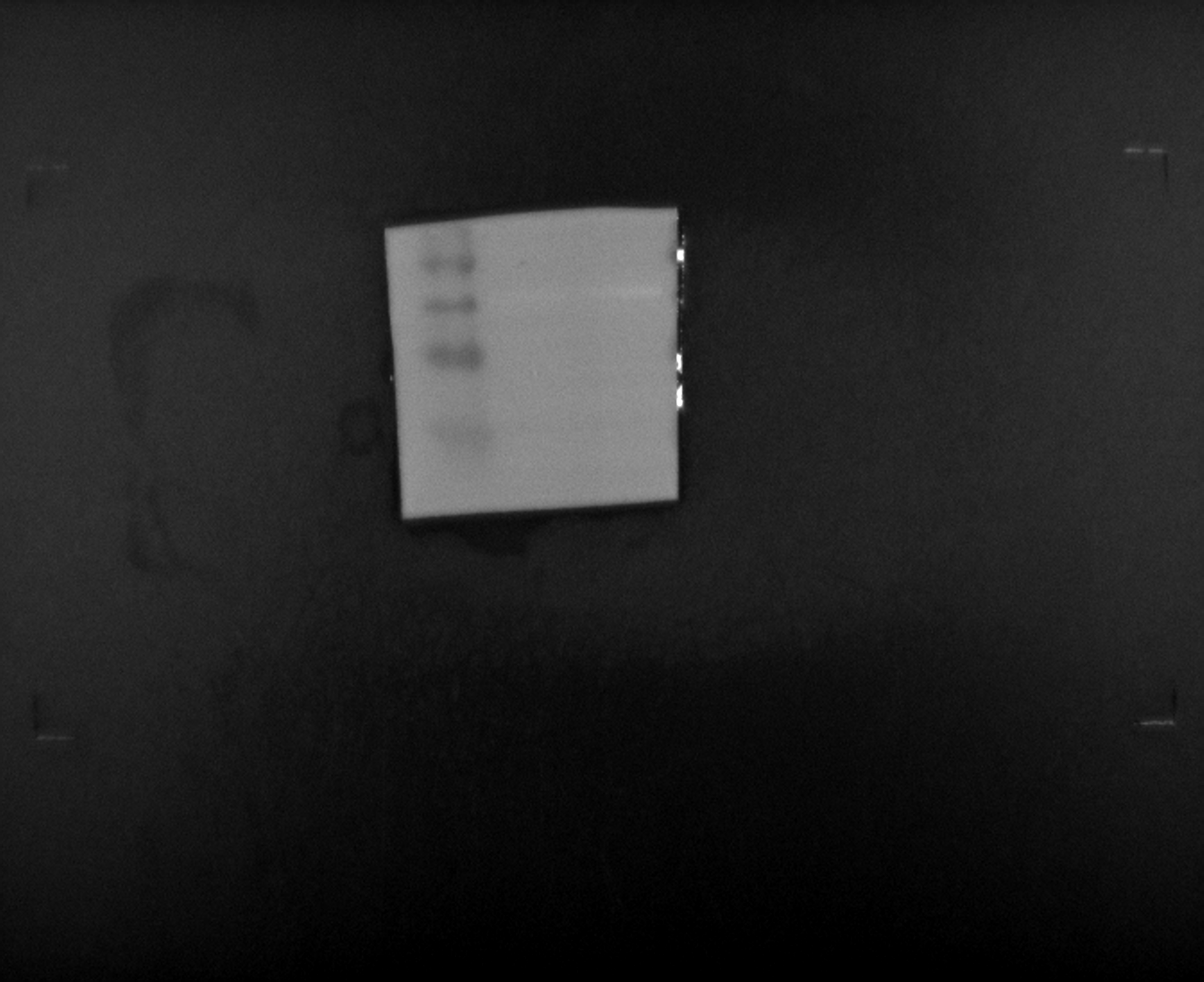

Supplement: Supplemental Information 2 [file peerj-12-17874-s002.zip › fig 1G/p-p65-1 (2).tif]

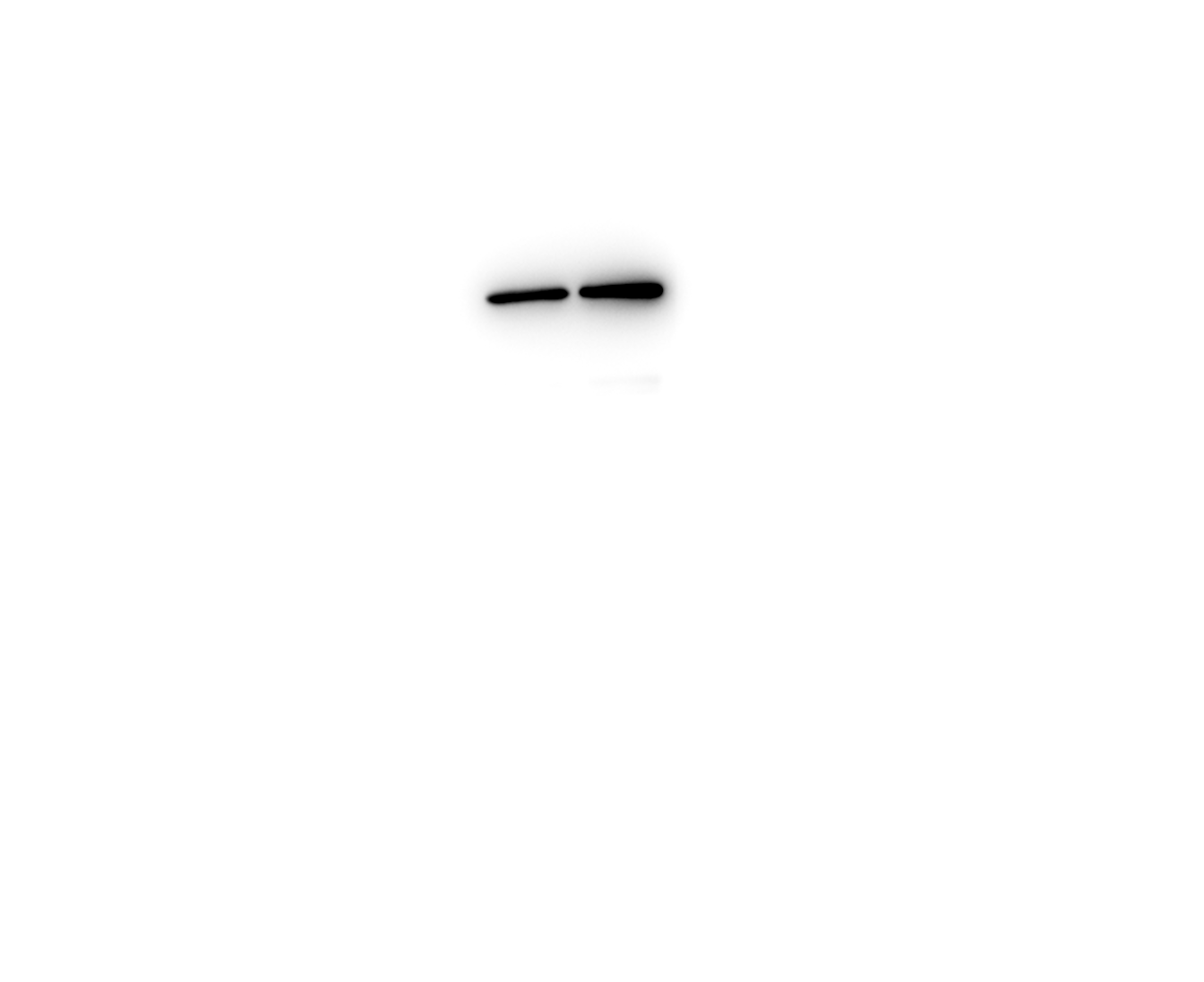

Supplement: Supplemental Information 2 [file peerj-12-17874-s002.zip › fig 1G/p-p65-1 (3).tif]

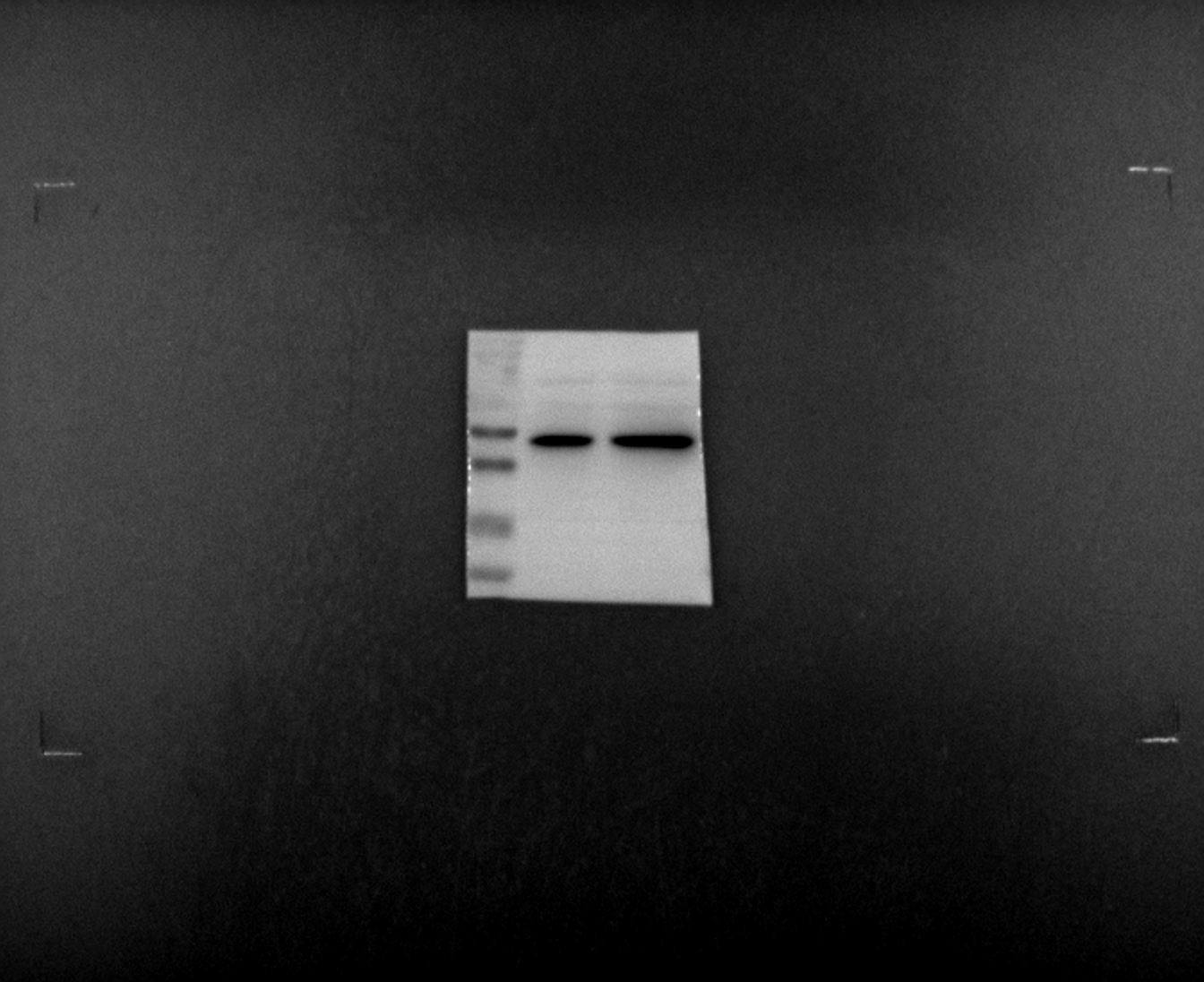

Supplement: Supplemental Information 2 [file peerj-12-17874-s002.zip › fig 1G/p-p65-2 (1).tif]

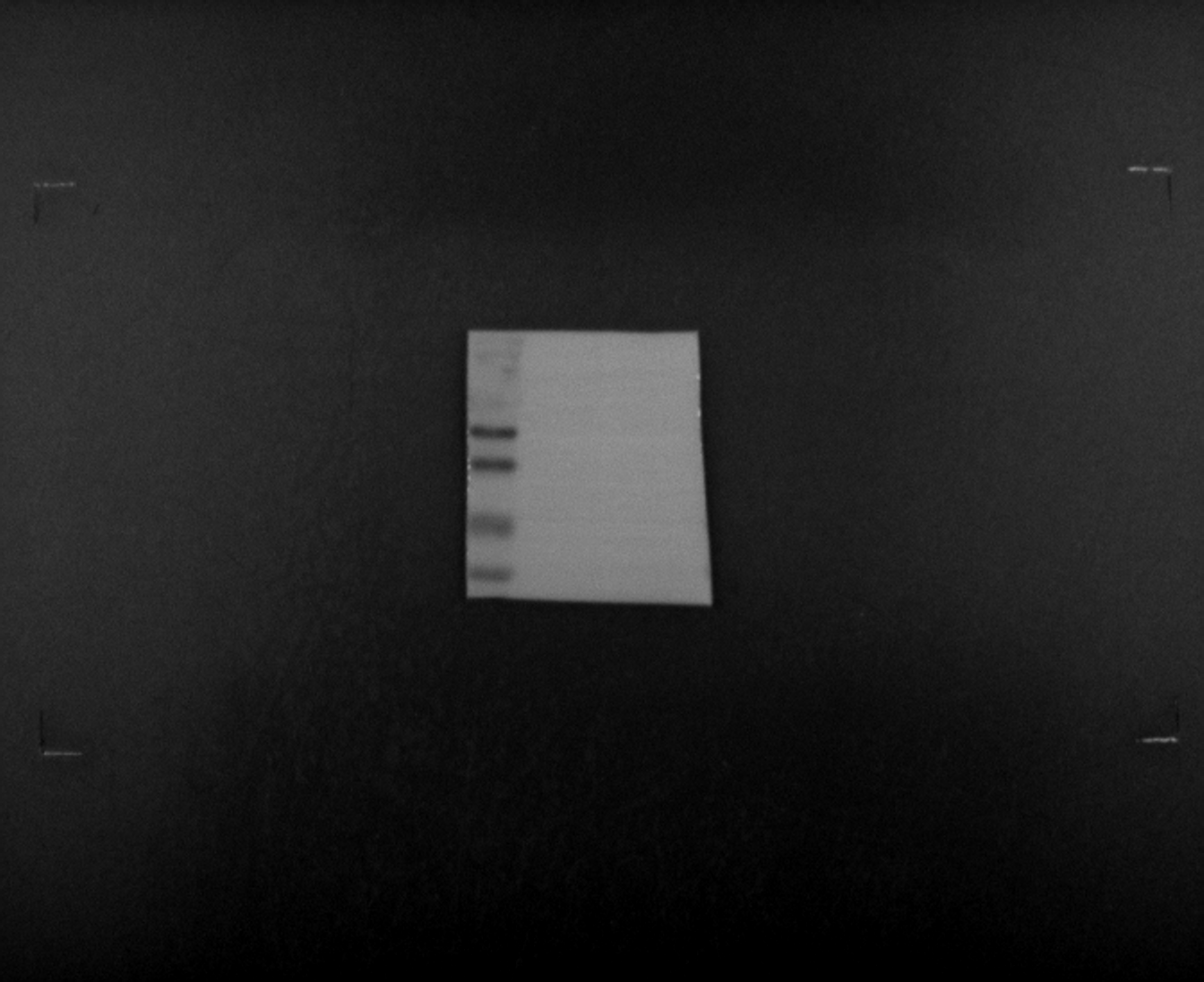

Supplement: Supplemental Information 2 [file peerj-12-17874-s002.zip › fig 1G/p-p65-2 (2).tif]

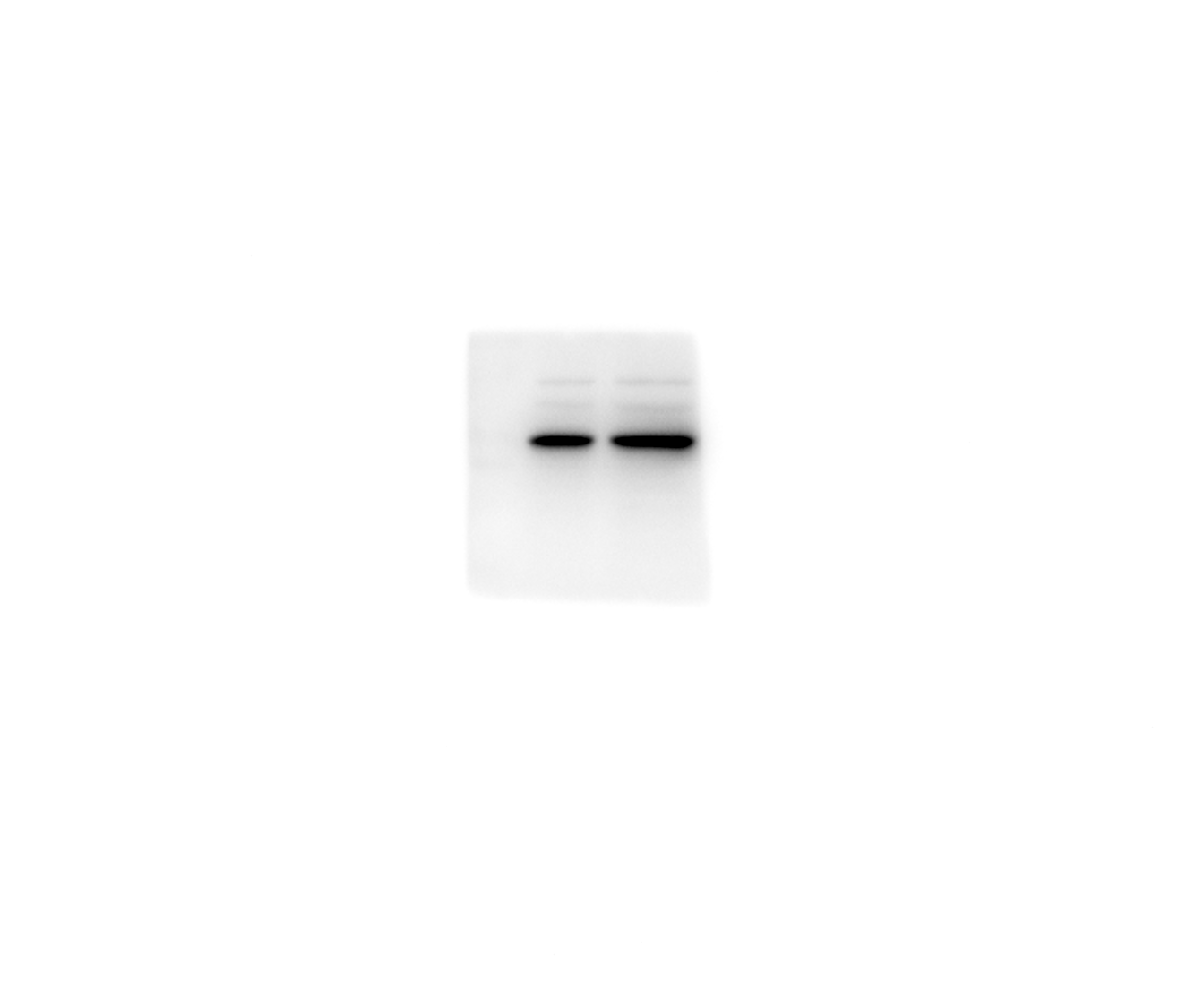

Supplement: Supplemental Information 2 [file peerj-12-17874-s002.zip › fig 1G/p-p65-2 (3).tif]

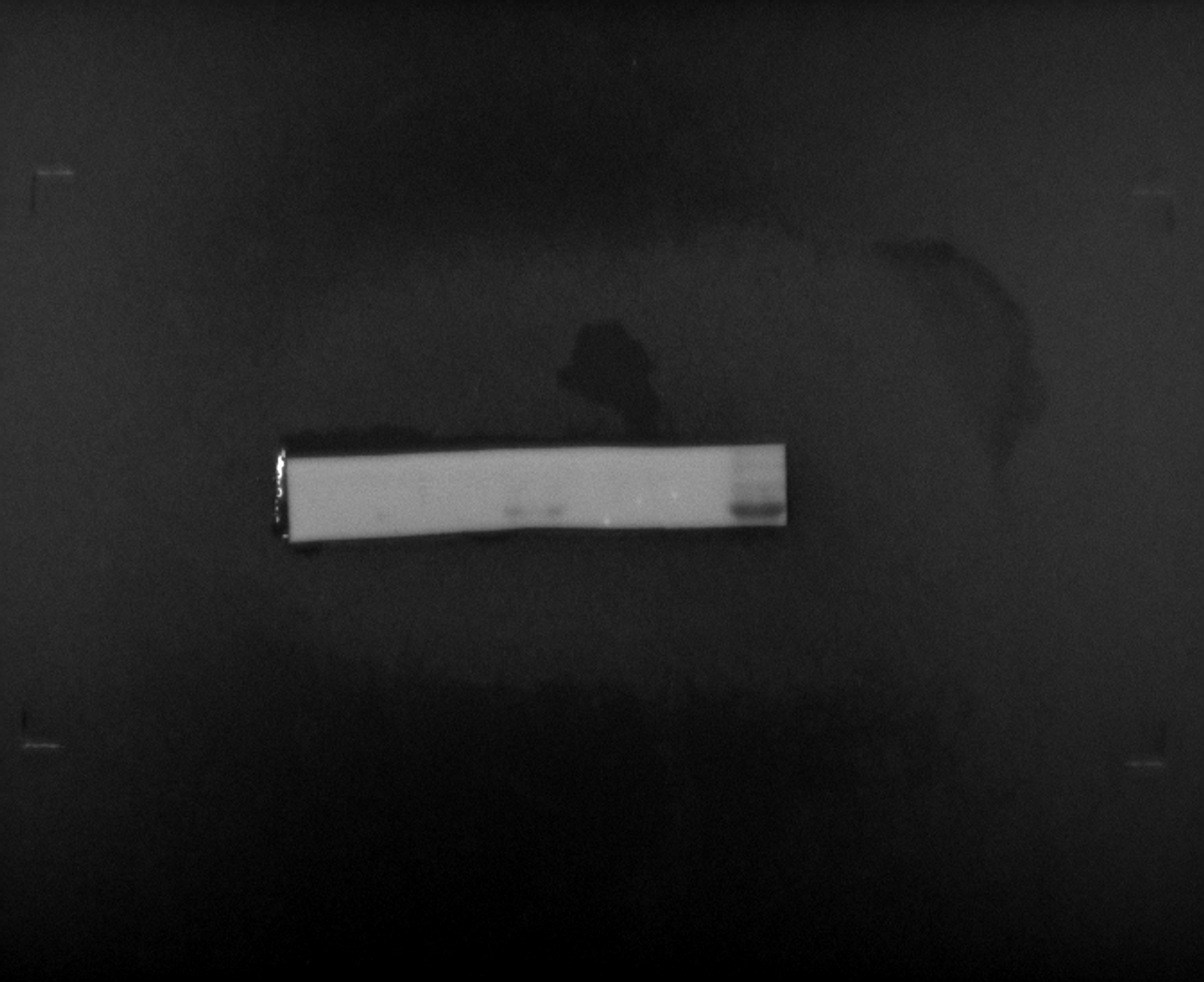

Supplement: Supplemental Information 3 [file peerj-12-17874-s003.zip › fig 2B/duox2 (1).tif]

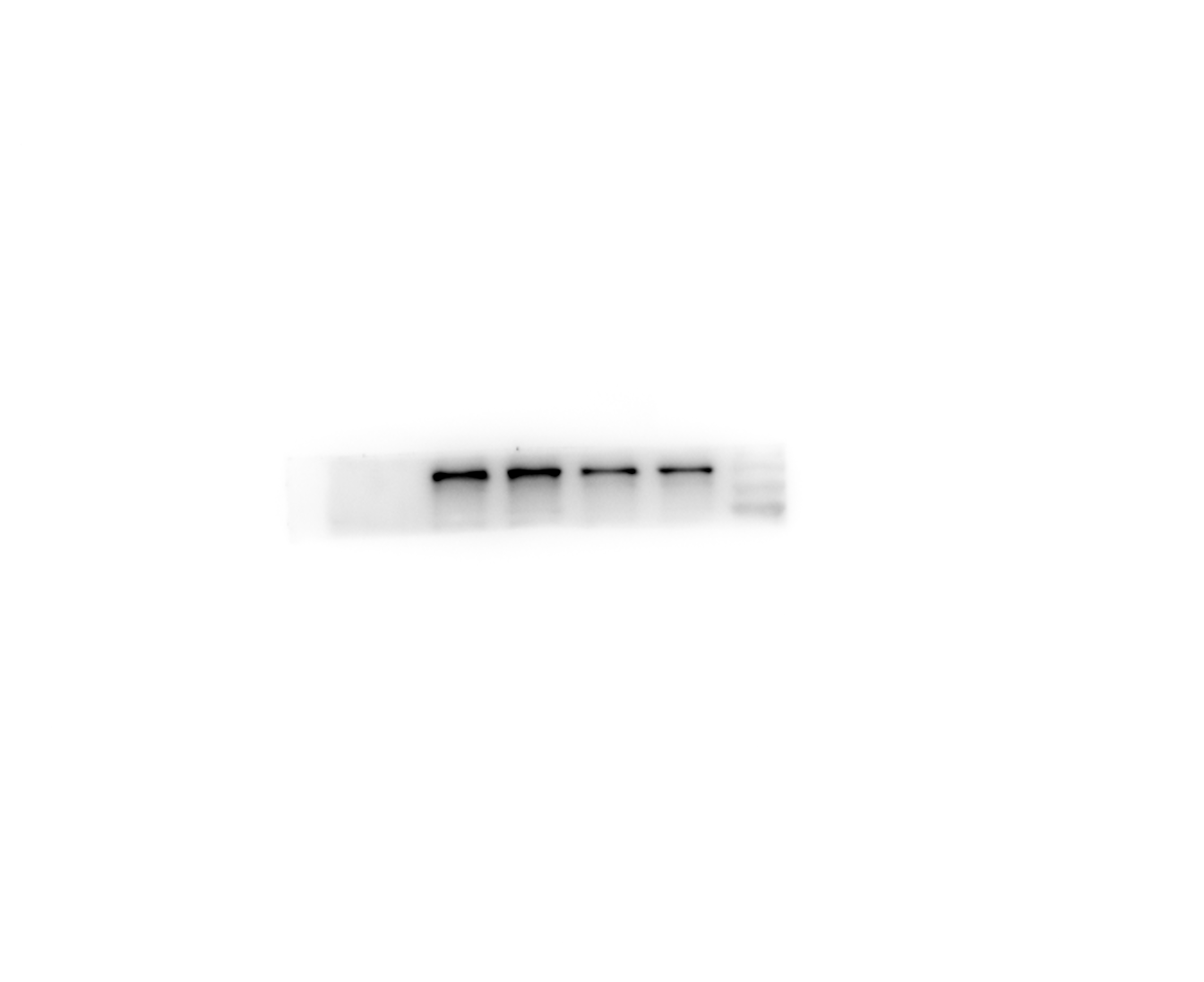

Supplement: Supplemental Information 3 [file peerj-12-17874-s003.zip › fig 2B/duox2 (2).tif]

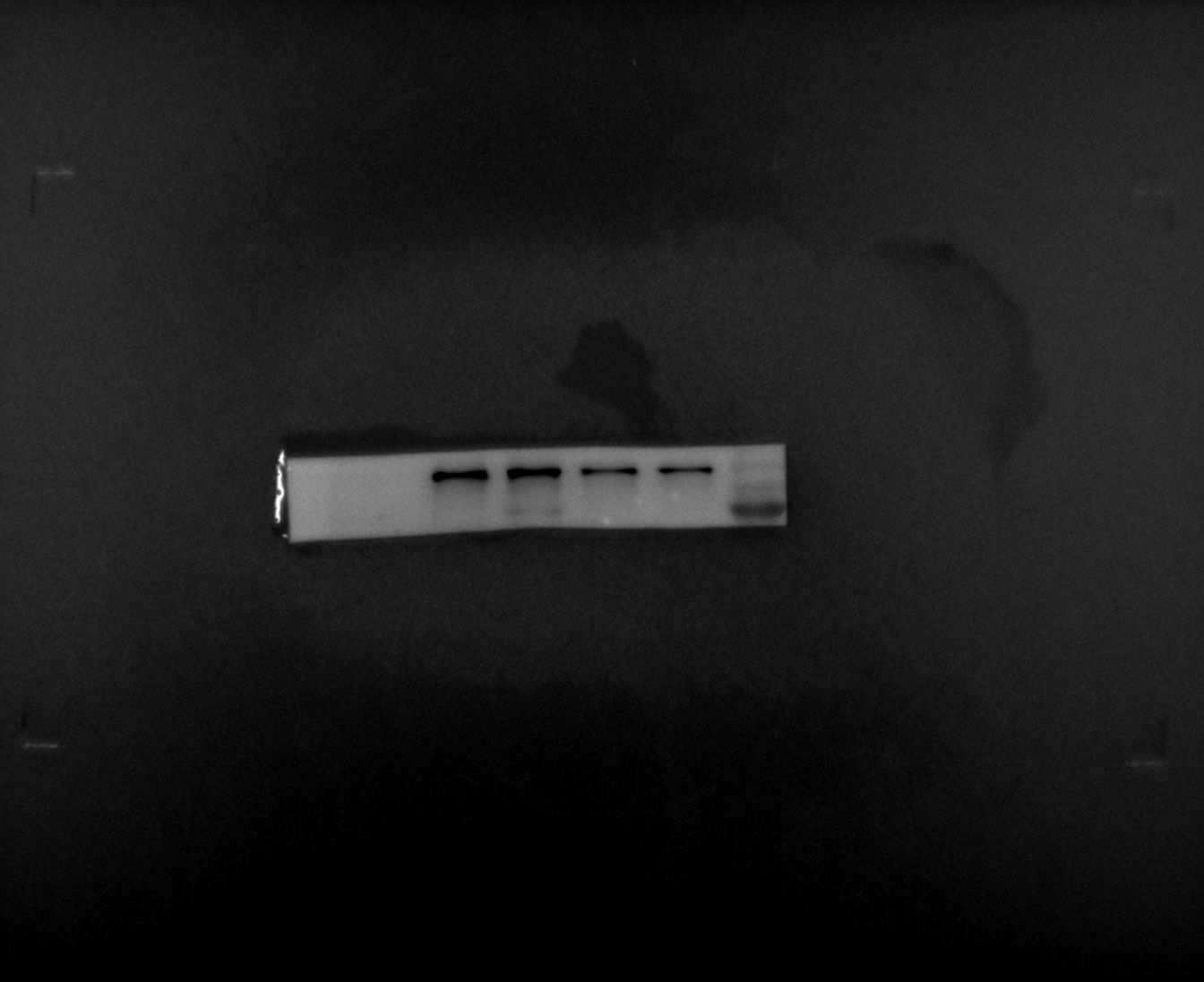

Supplement: Supplemental Information 3 [file peerj-12-17874-s003.zip › fig 2B/duox2 (3).tif]

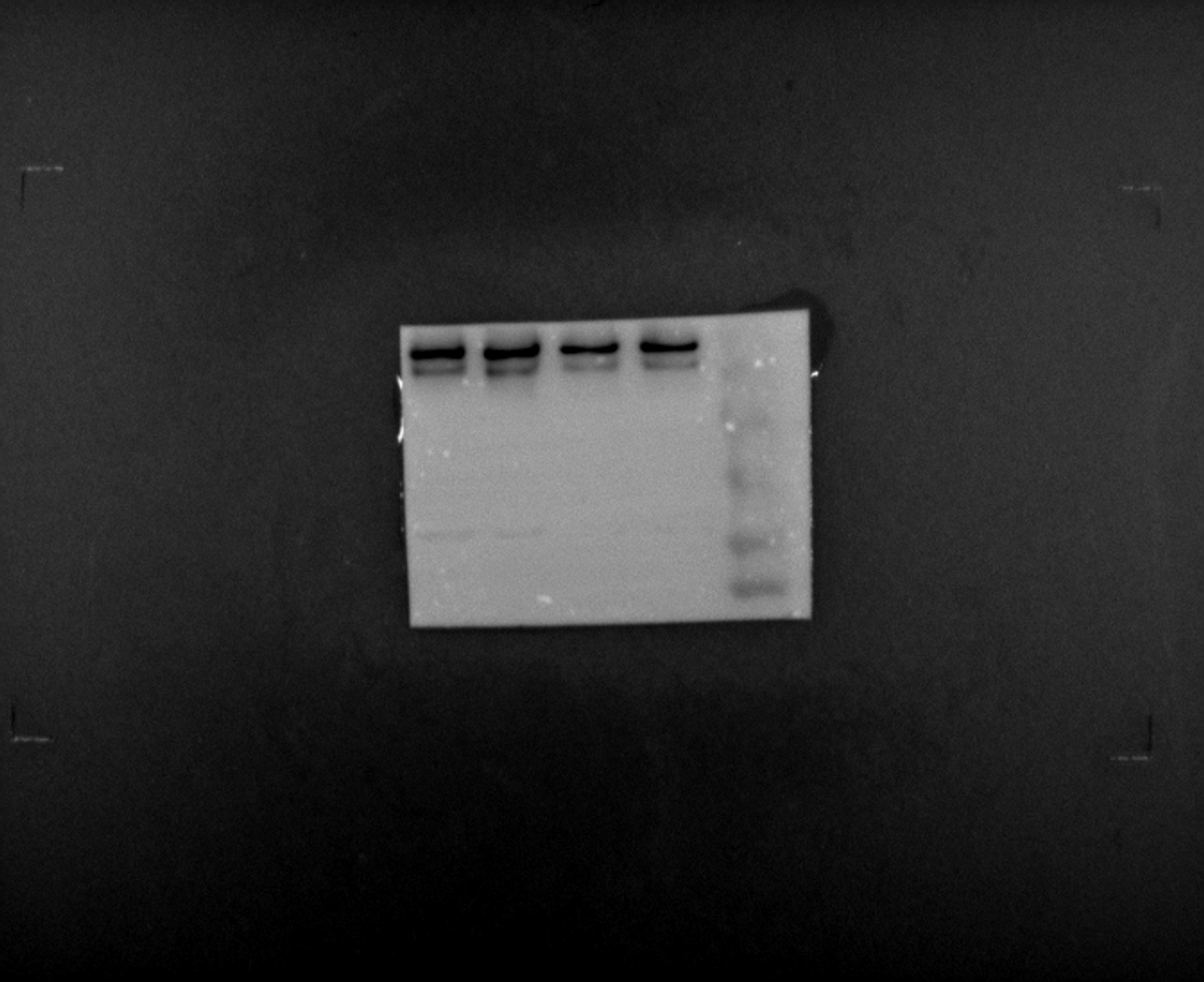

Supplement: Supplemental Information 3 [file peerj-12-17874-s003.zip › fig 2B/DUOX2-2 (1).tif]

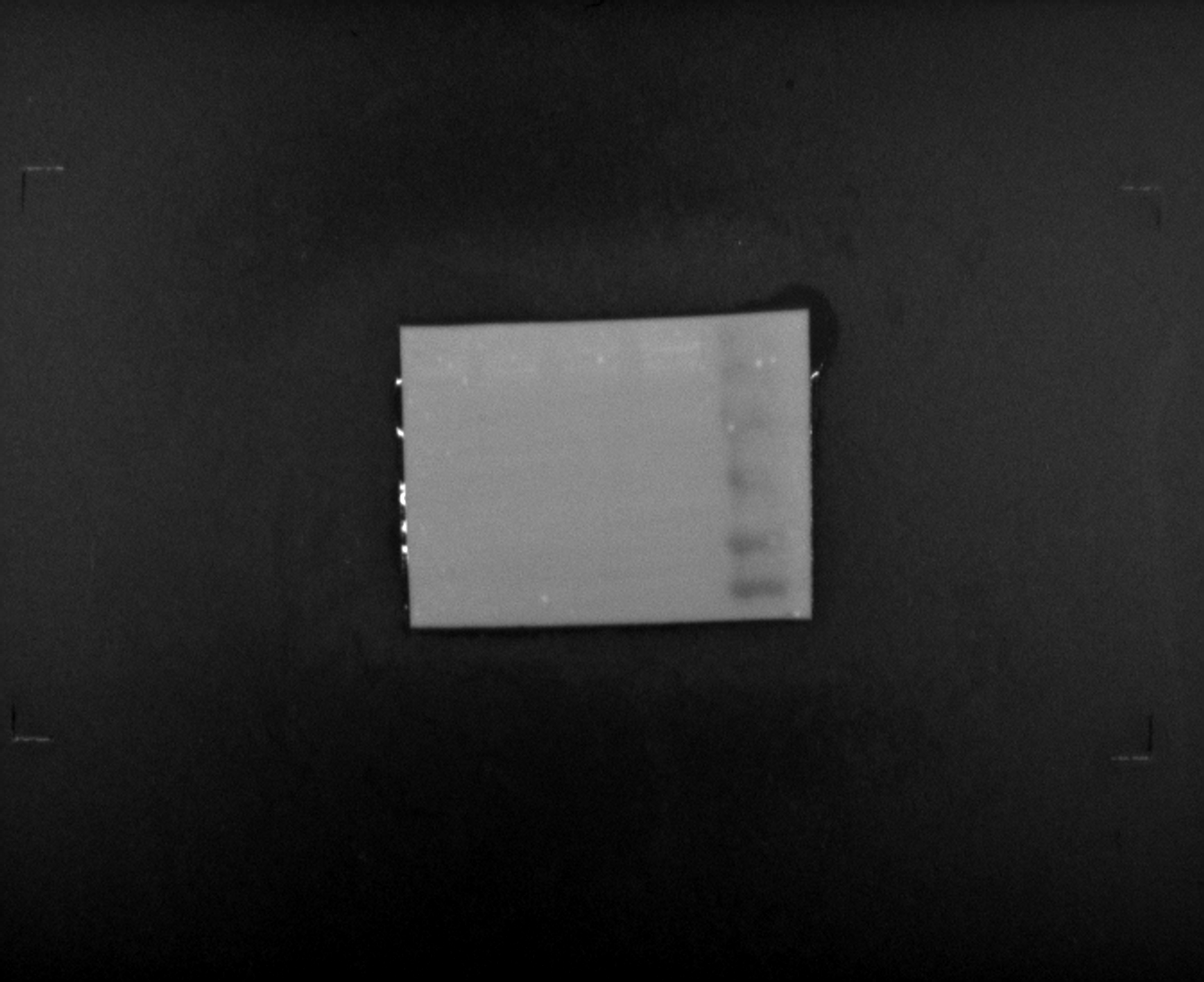

Supplement: Supplemental Information 3 [file peerj-12-17874-s003.zip › fig 2B/DUOX2-2 (2).tif]

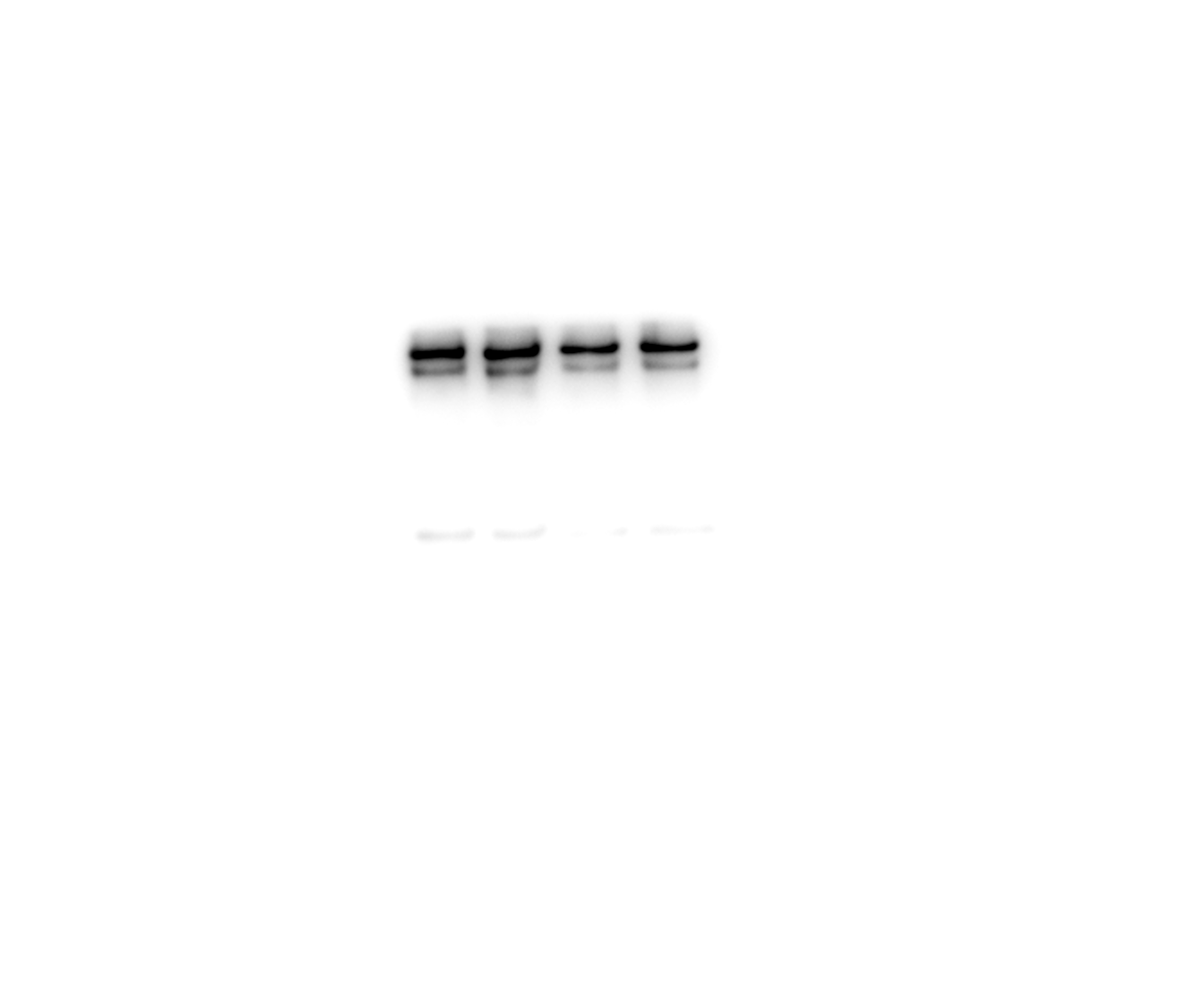

Supplement: Supplemental Information 3 [file peerj-12-17874-s003.zip › fig 2B/DUOX2-2 (3).tif]

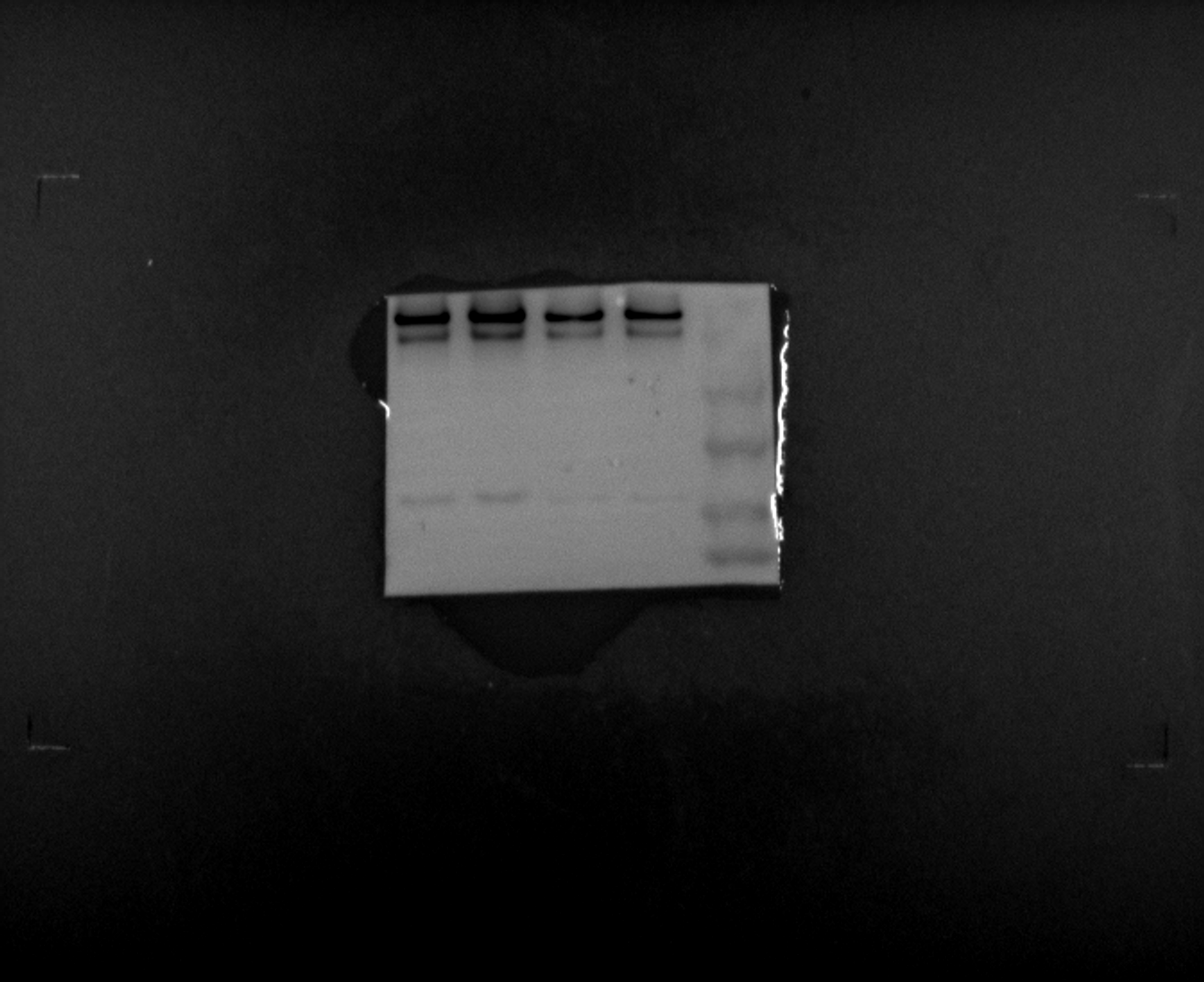

Supplement: Supplemental Information 3 [file peerj-12-17874-s003.zip › fig 2B/DUOX2-3 (1).tif]

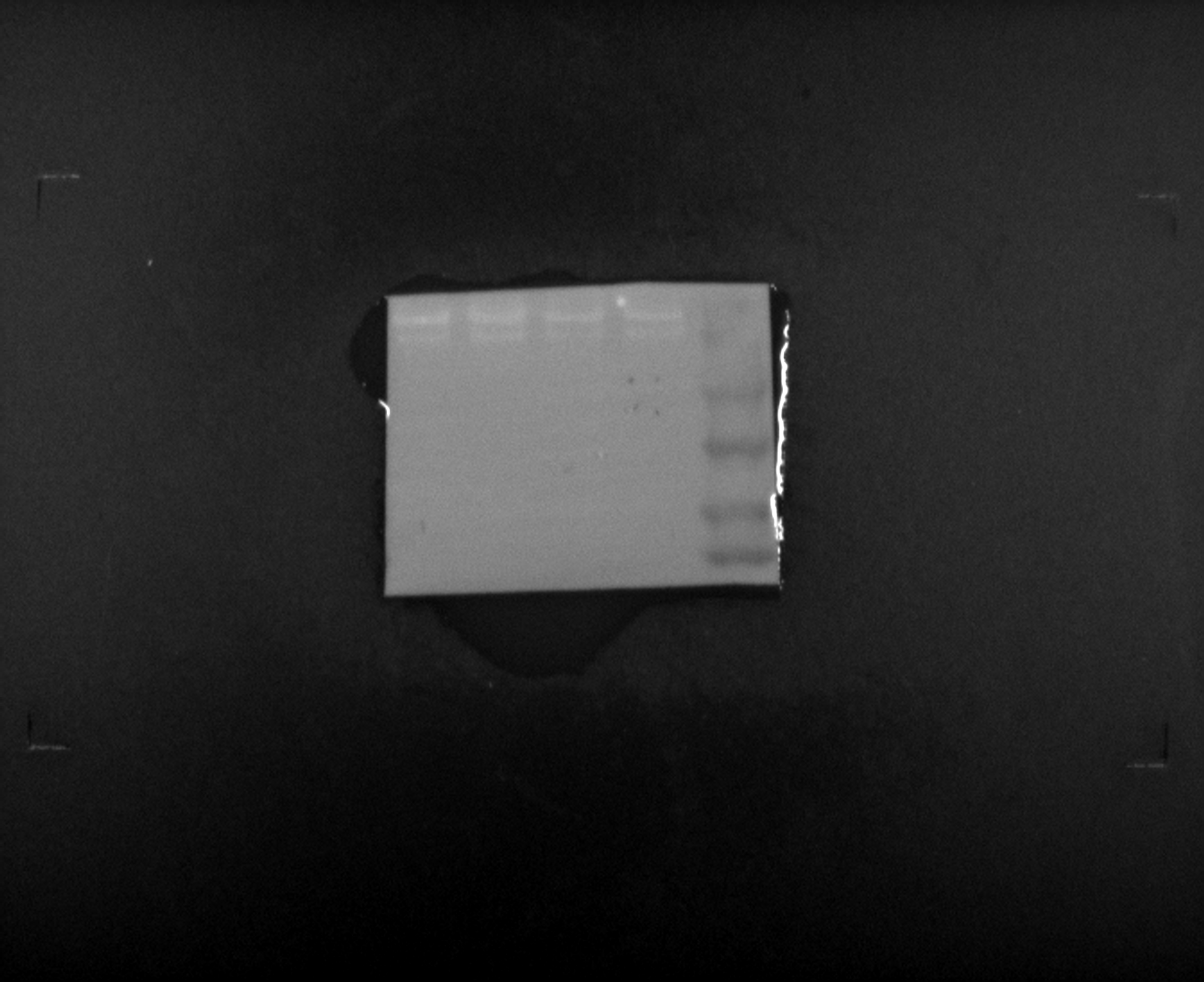

Supplement: Supplemental Information 3 [file peerj-12-17874-s003.zip › fig 2B/DUOX2-3 (2).tif]

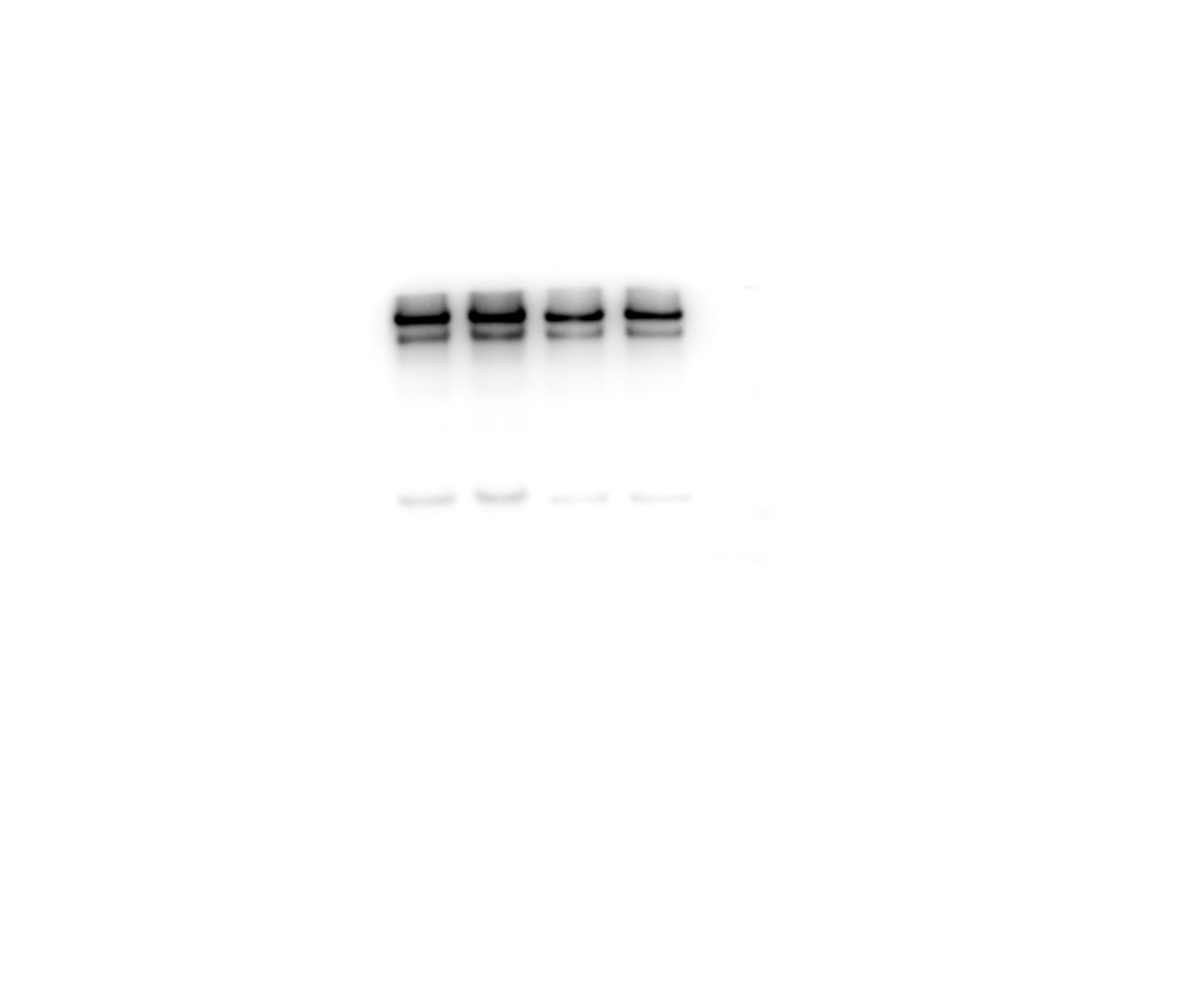

Supplement: Supplemental Information 3 [file peerj-12-17874-s003.zip › fig 2B/DUOX2-3 (3).tif]

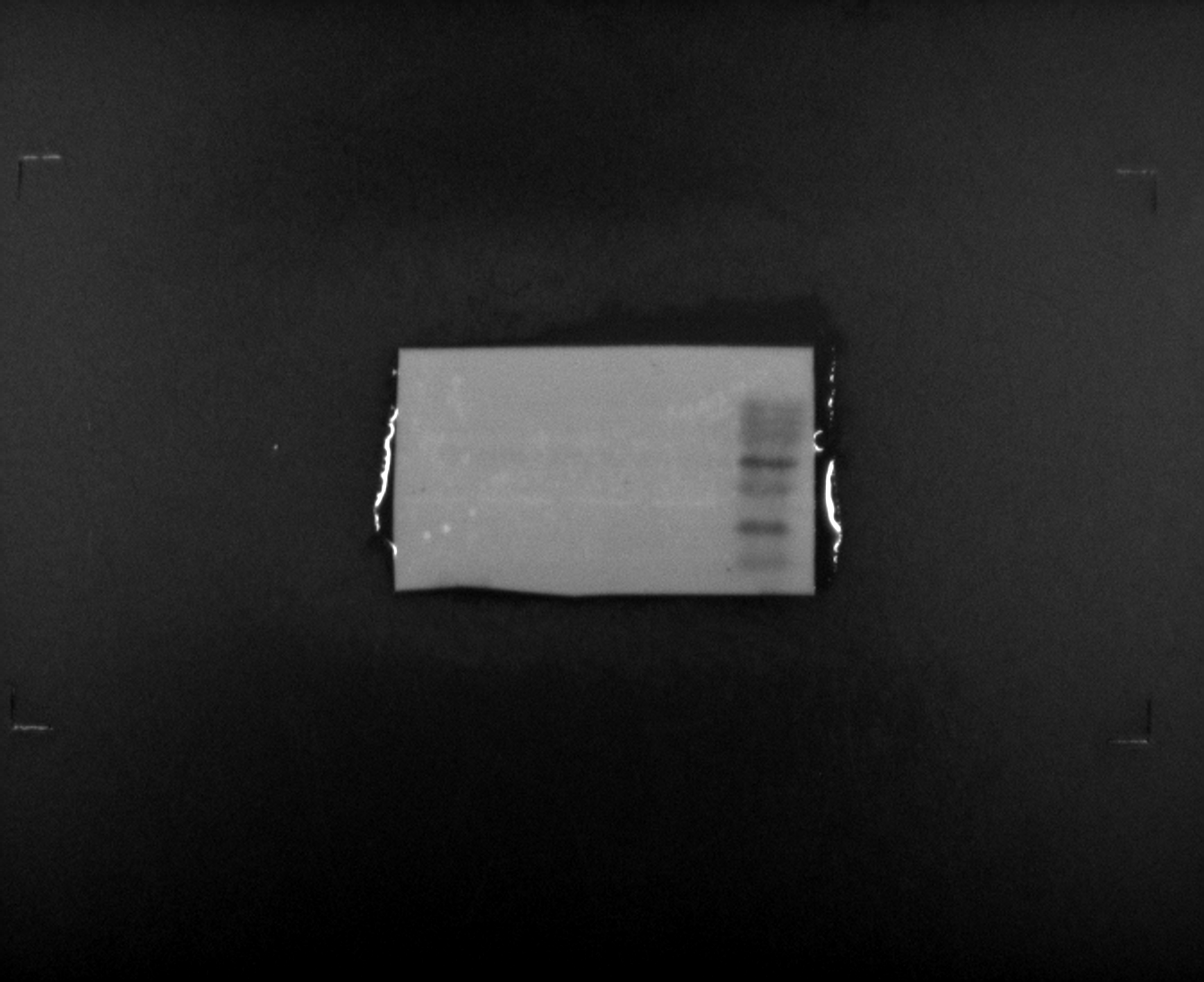

Supplement: Supplemental Information 3 [file peerj-12-17874-s003.zip › fig 2B/GAPDH-1 (1).tif]

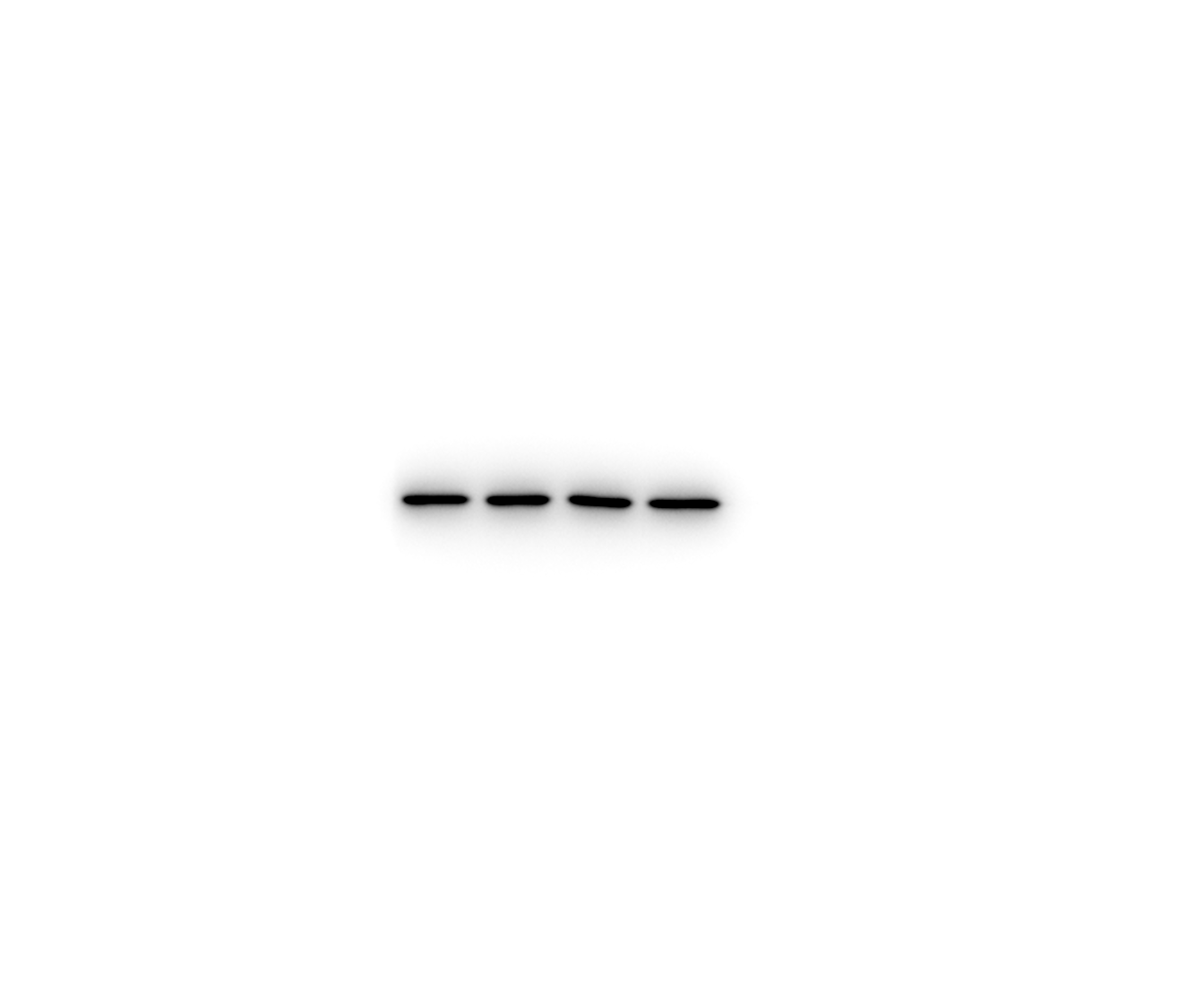

Supplement: Supplemental Information 3 [file peerj-12-17874-s003.zip › fig 2B/GAPDH-1 (2).tif]

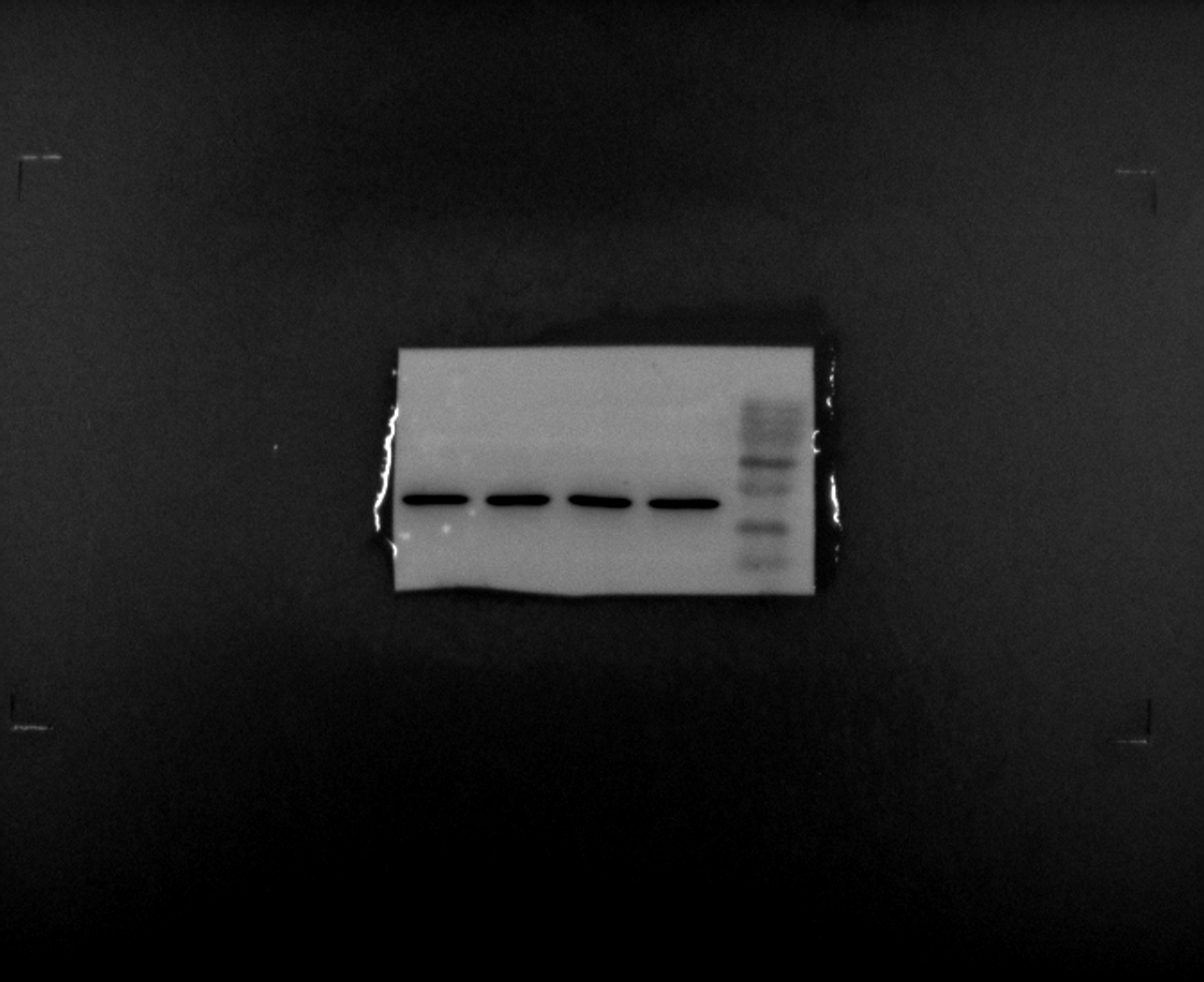

Supplement: Supplemental Information 3 [file peerj-12-17874-s003.zip › fig 2B/GAPDH-1 (3).tif]

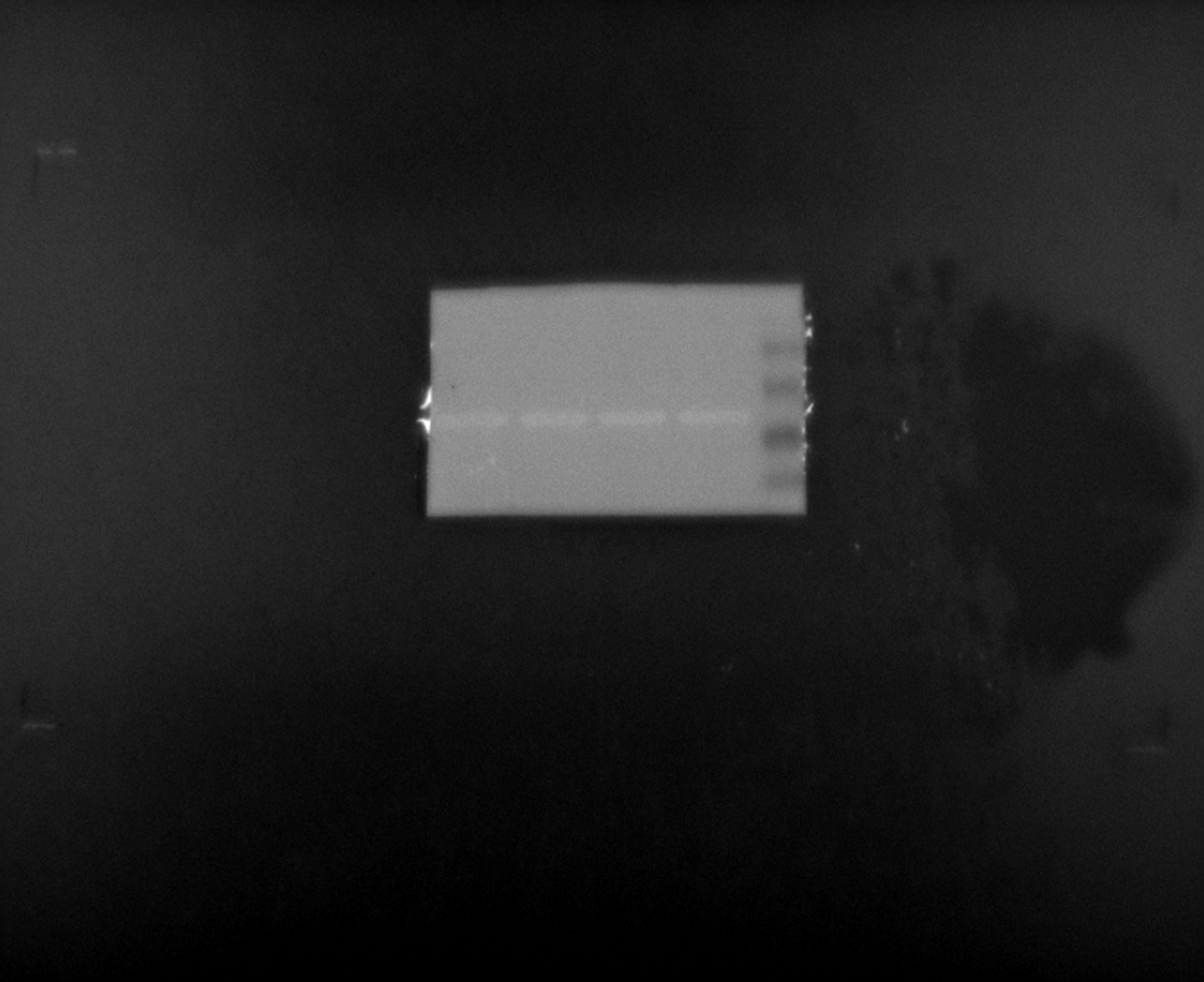

Supplement: Supplemental Information 3 [file peerj-12-17874-s003.zip › fig 2B/GAPDH-2 (1).tif]

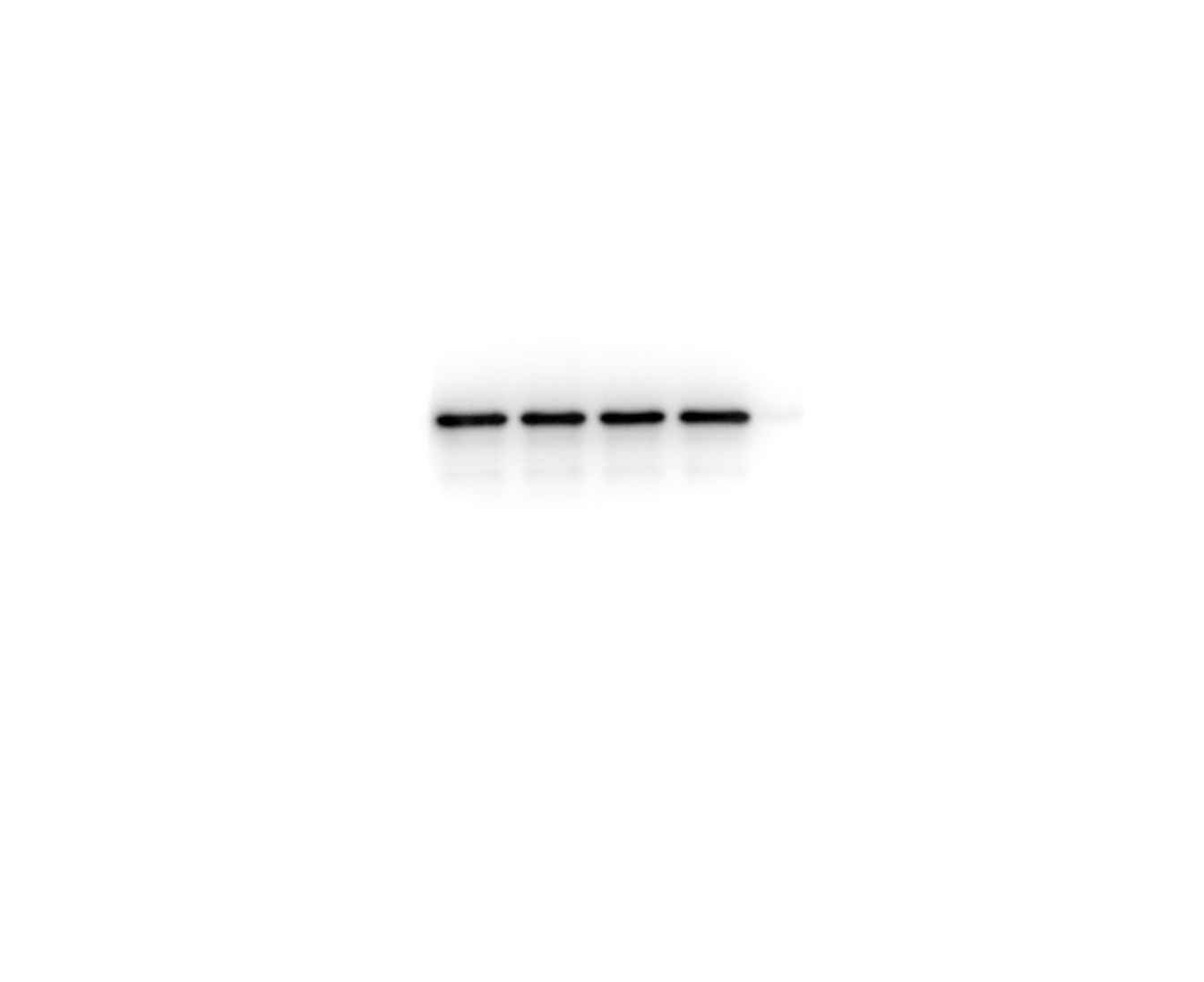

Supplement: Supplemental Information 3 [file peerj-12-17874-s003.zip › fig 2B/GAPDH-2 (2).tif]

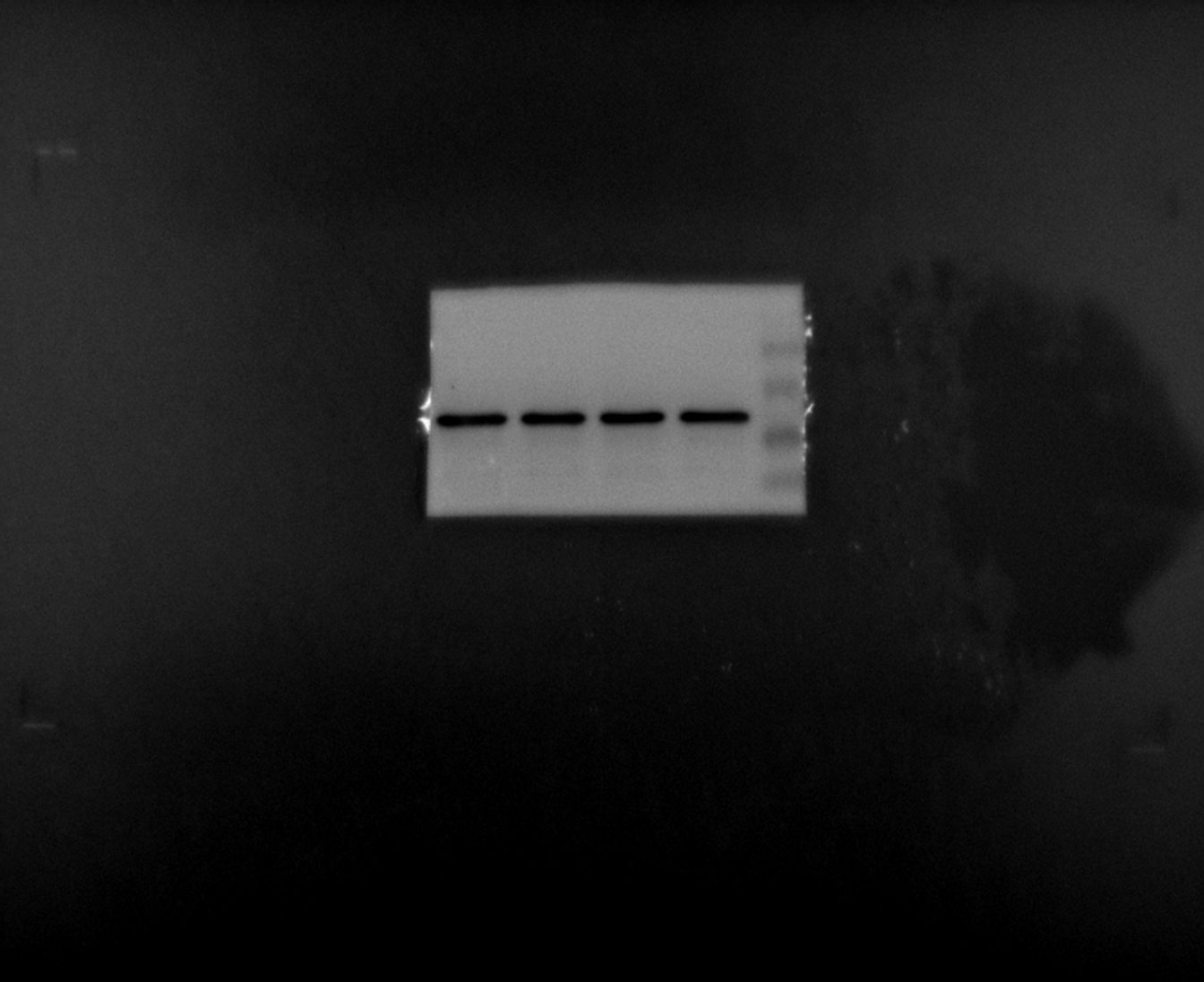

Supplement: Supplemental Information 3 [file peerj-12-17874-s003.zip › fig 2B/GAPDH-2 (3).tif]

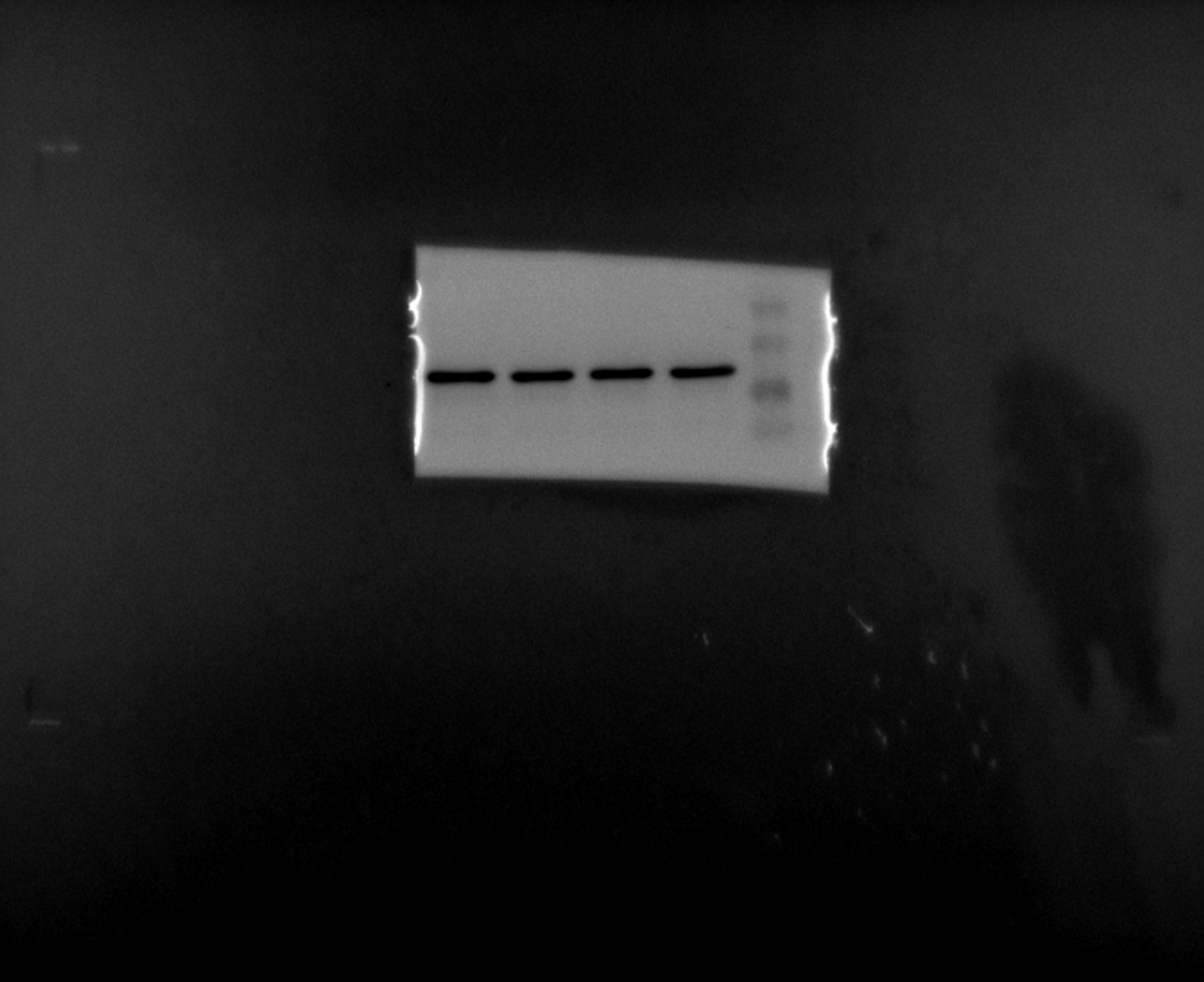

Supplement: Supplemental Information 3 [file peerj-12-17874-s003.zip › fig 2B/GAPDH-3 (1).tif]

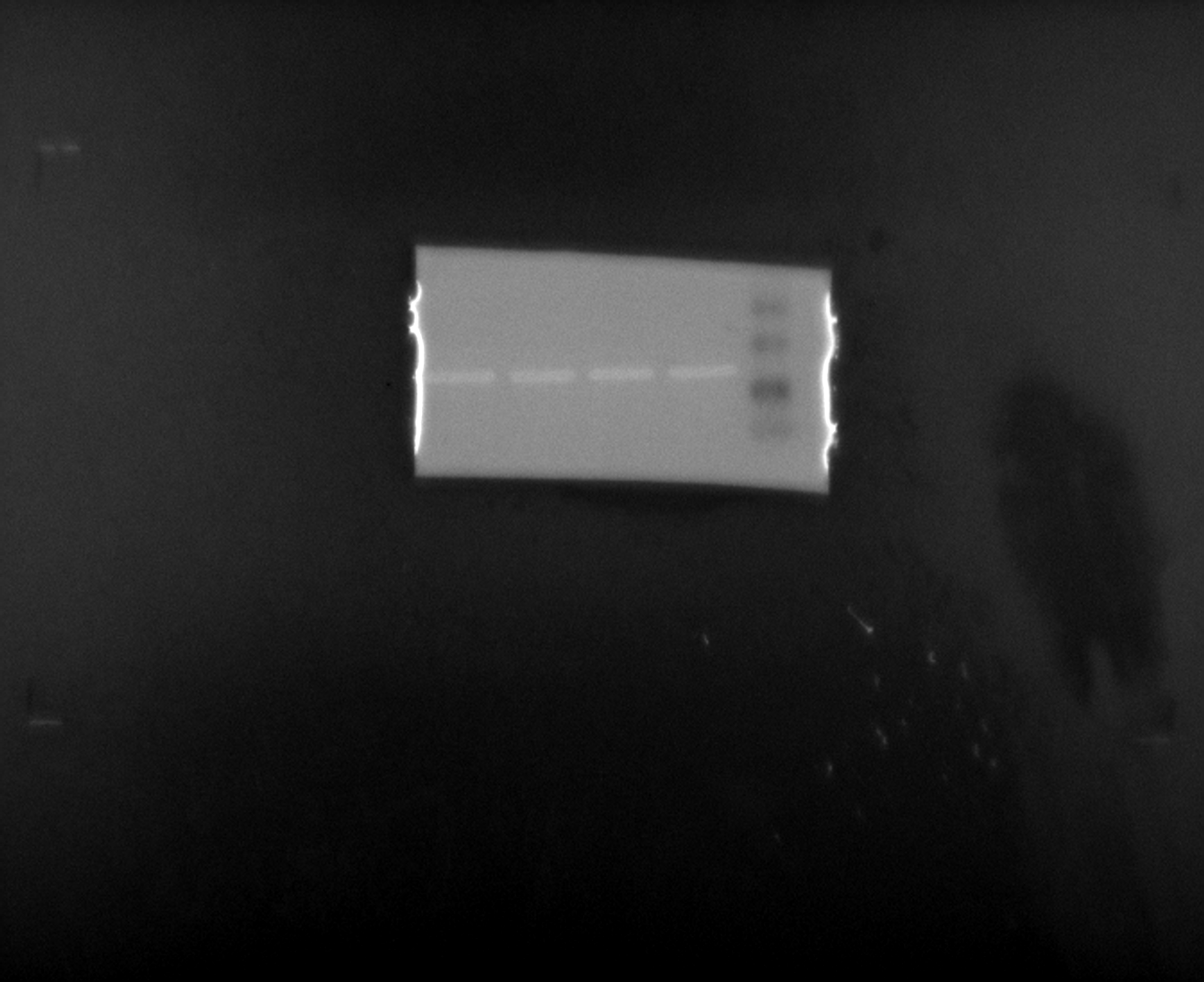

Supplement: Supplemental Information 3 [file peerj-12-17874-s003.zip › fig 2B/GAPDH-3 (2).tif]

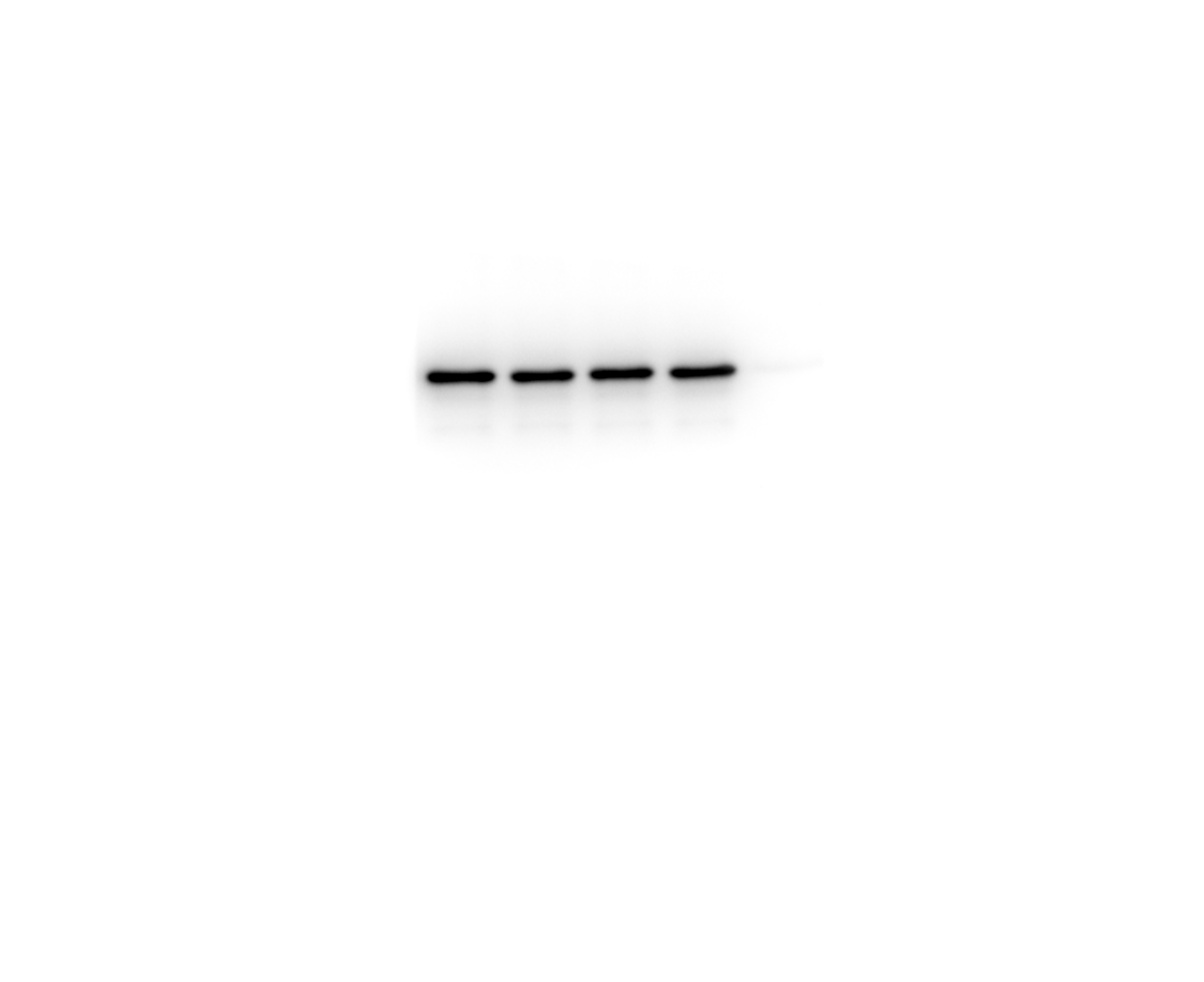

Supplement: Supplemental Information 3 [file peerj-12-17874-s003.zip › fig 2B/GAPDH-3 (3).tif]

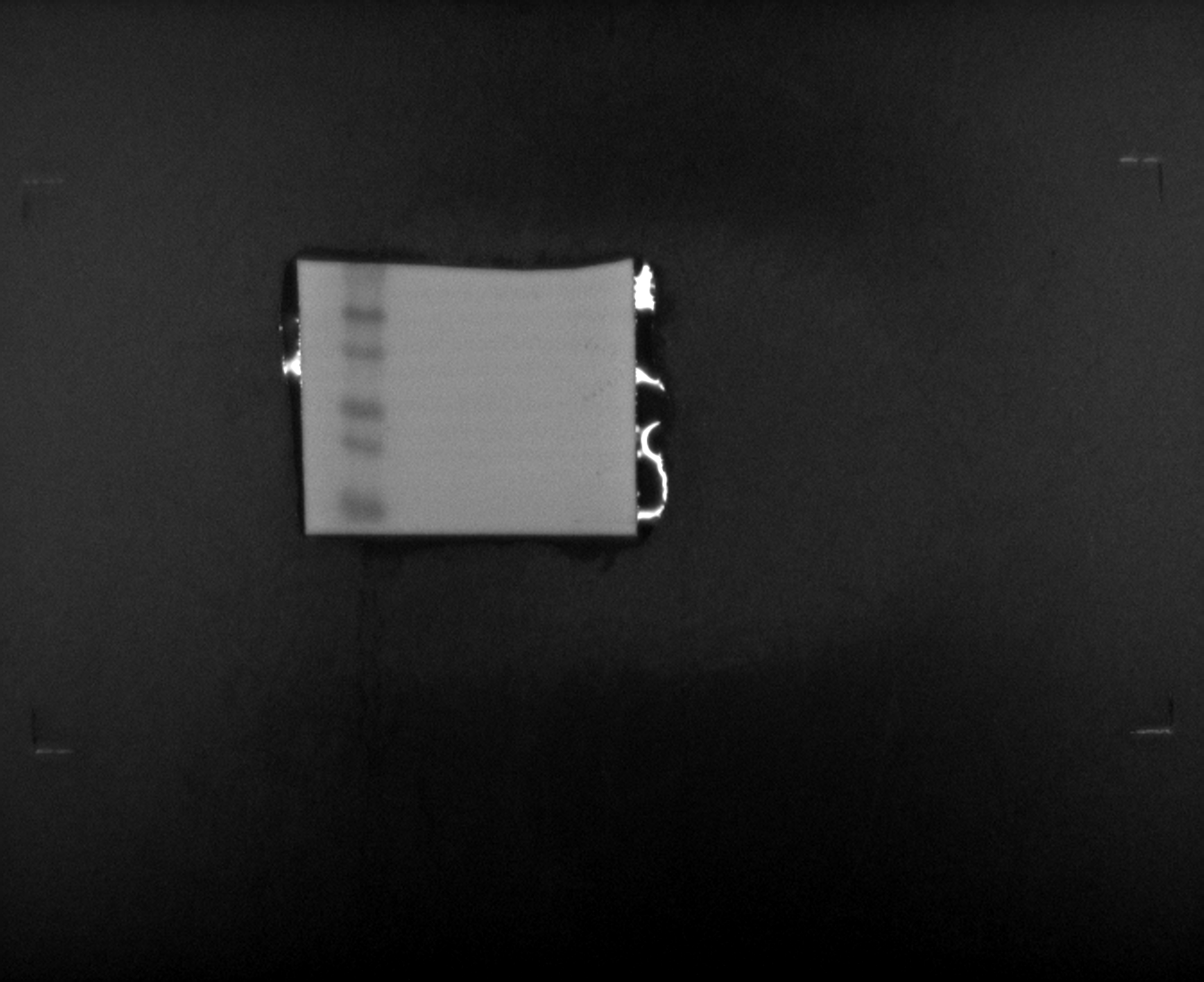

Supplement: Supplemental Information 4 [file peerj-12-17874-s004.zip › fig 2F/BAX (1).tif]

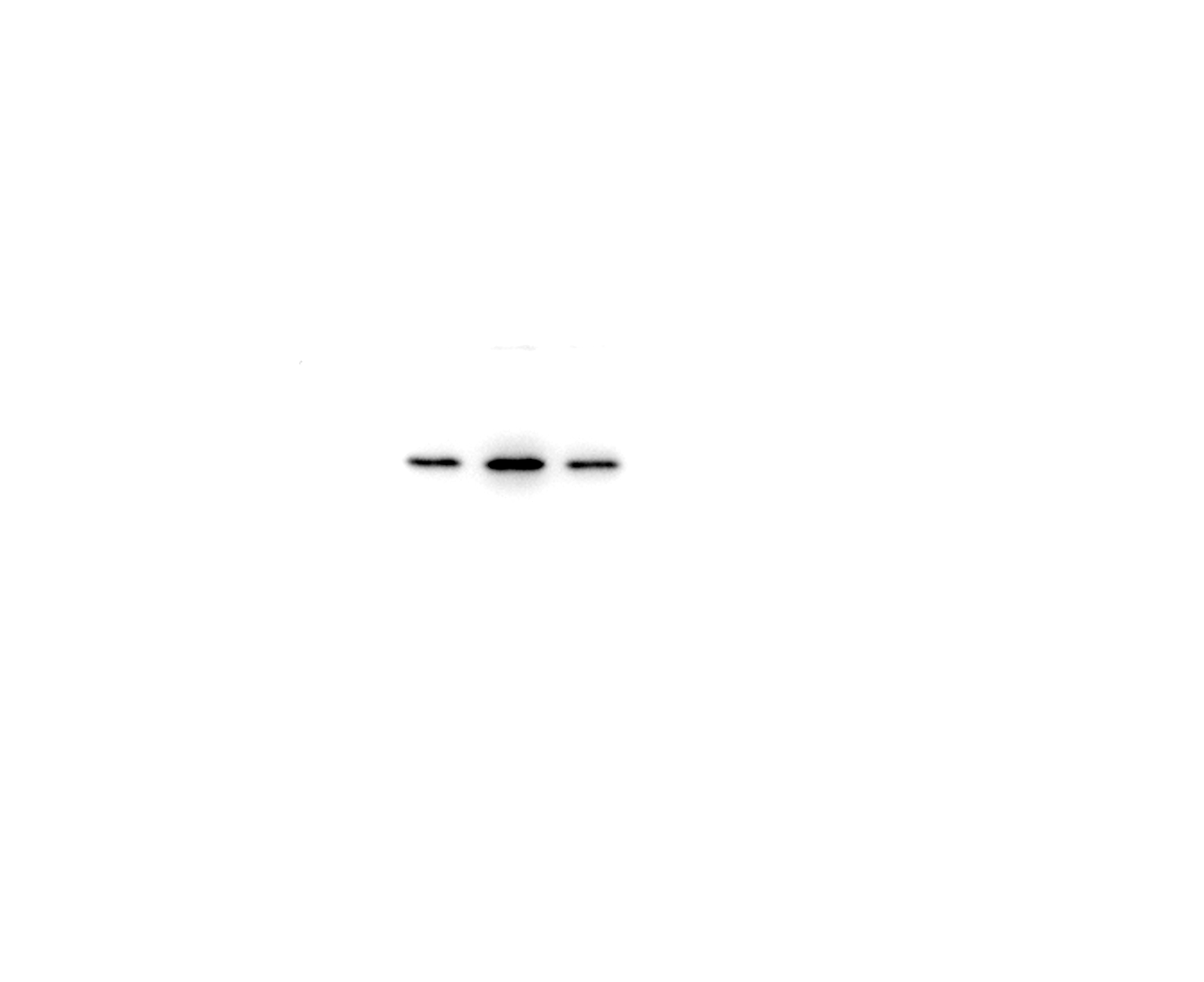

Supplement: Supplemental Information 4 [file peerj-12-17874-s004.zip › fig 2F/BAX (2).tif]

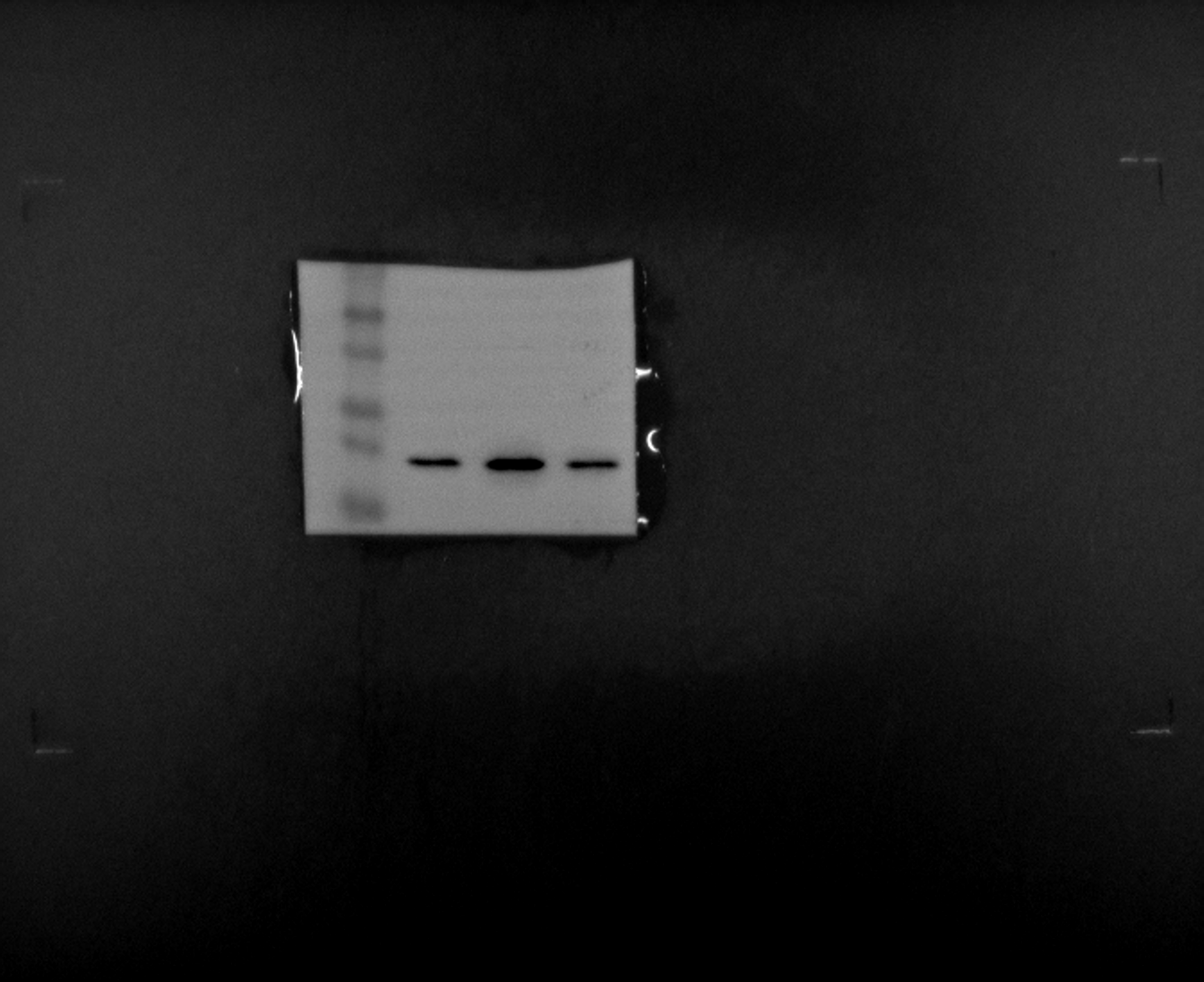

Supplement: Supplemental Information 4 [file peerj-12-17874-s004.zip › fig 2F/BAX (3).tif]

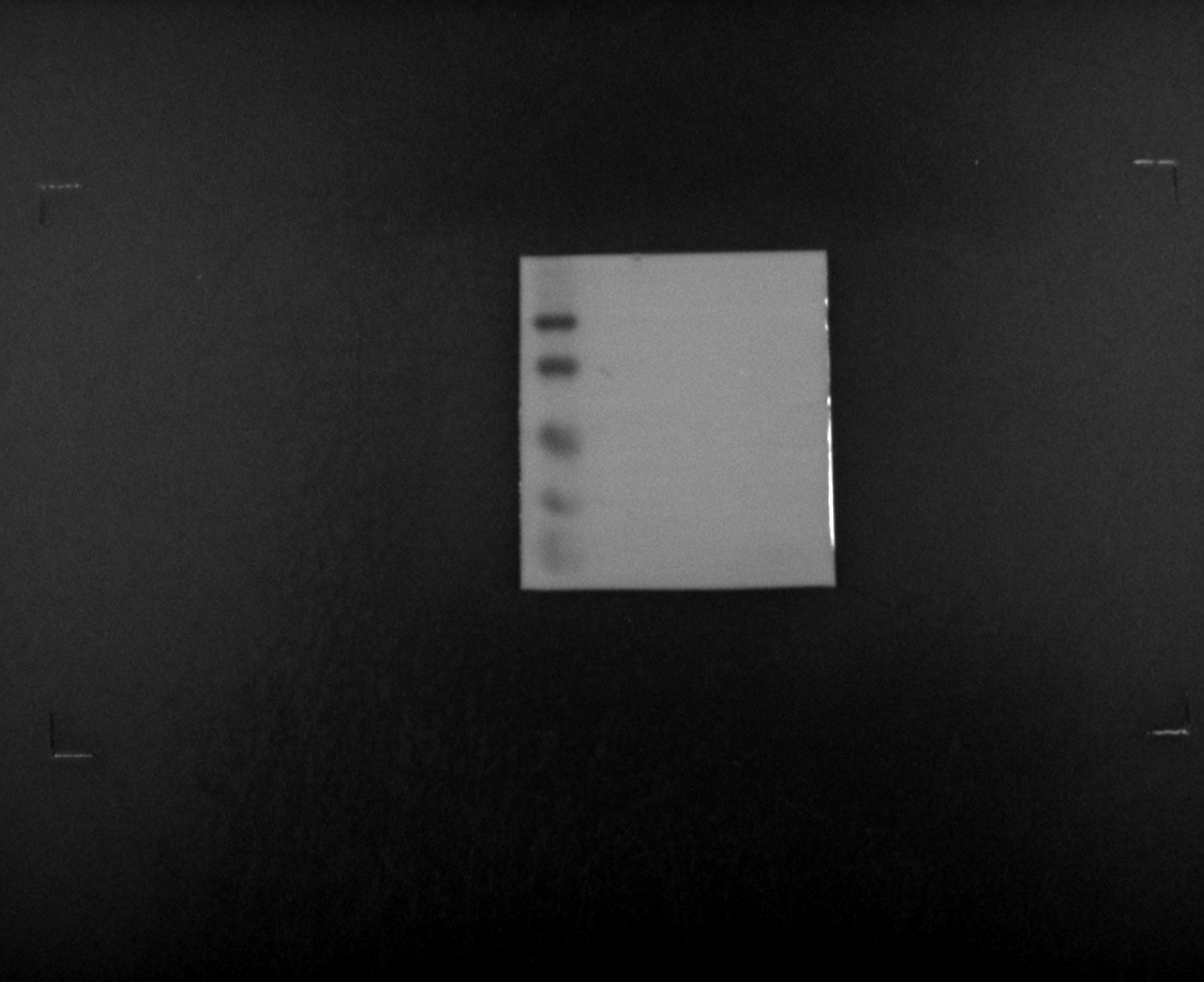

Supplement: Supplemental Information 4 [file peerj-12-17874-s004.zip › fig 2F/bax-2 (1).tif]

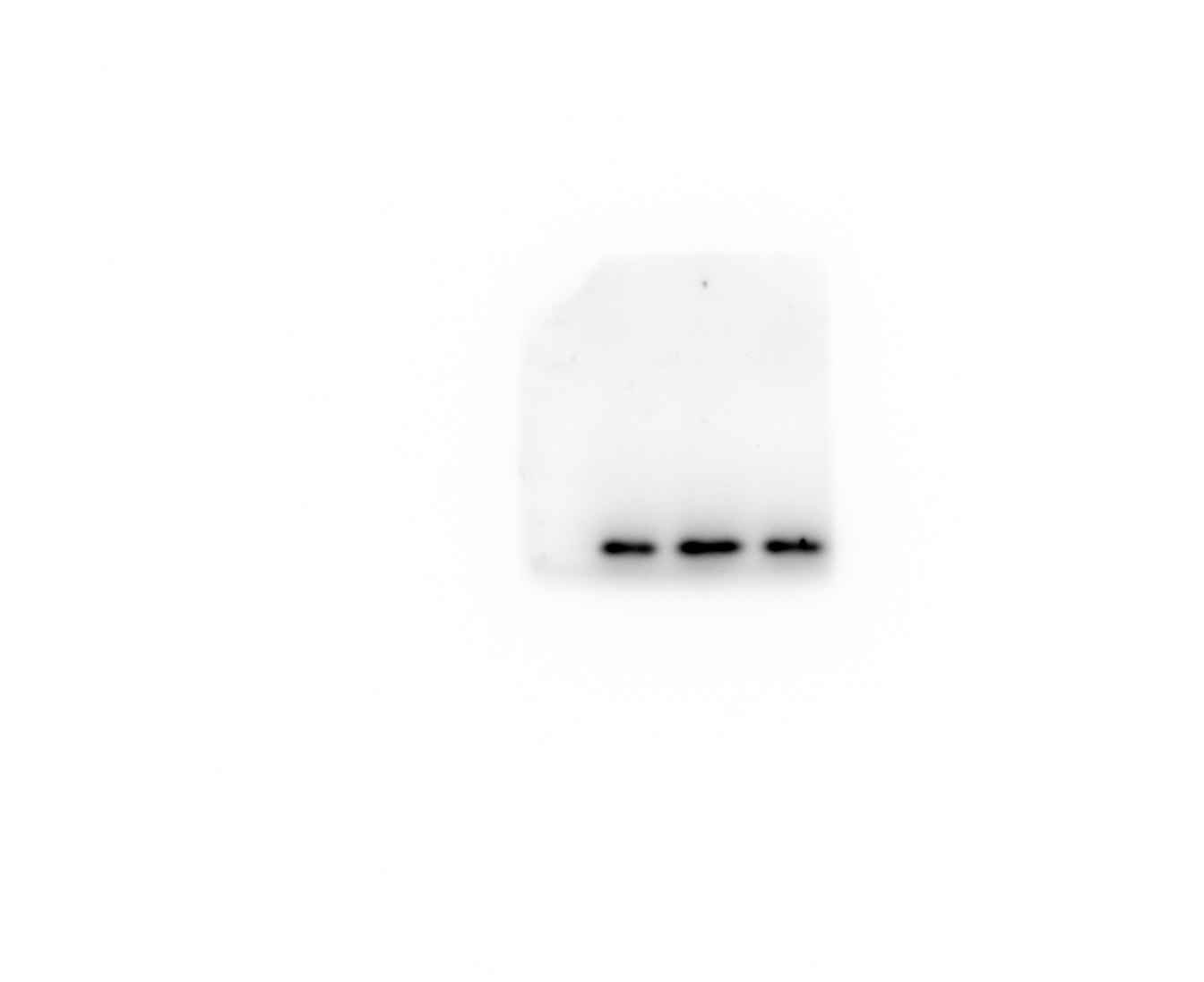

Supplement: Supplemental Information 4 [file peerj-12-17874-s004.zip › fig 2F/bax-2 (2).tif]

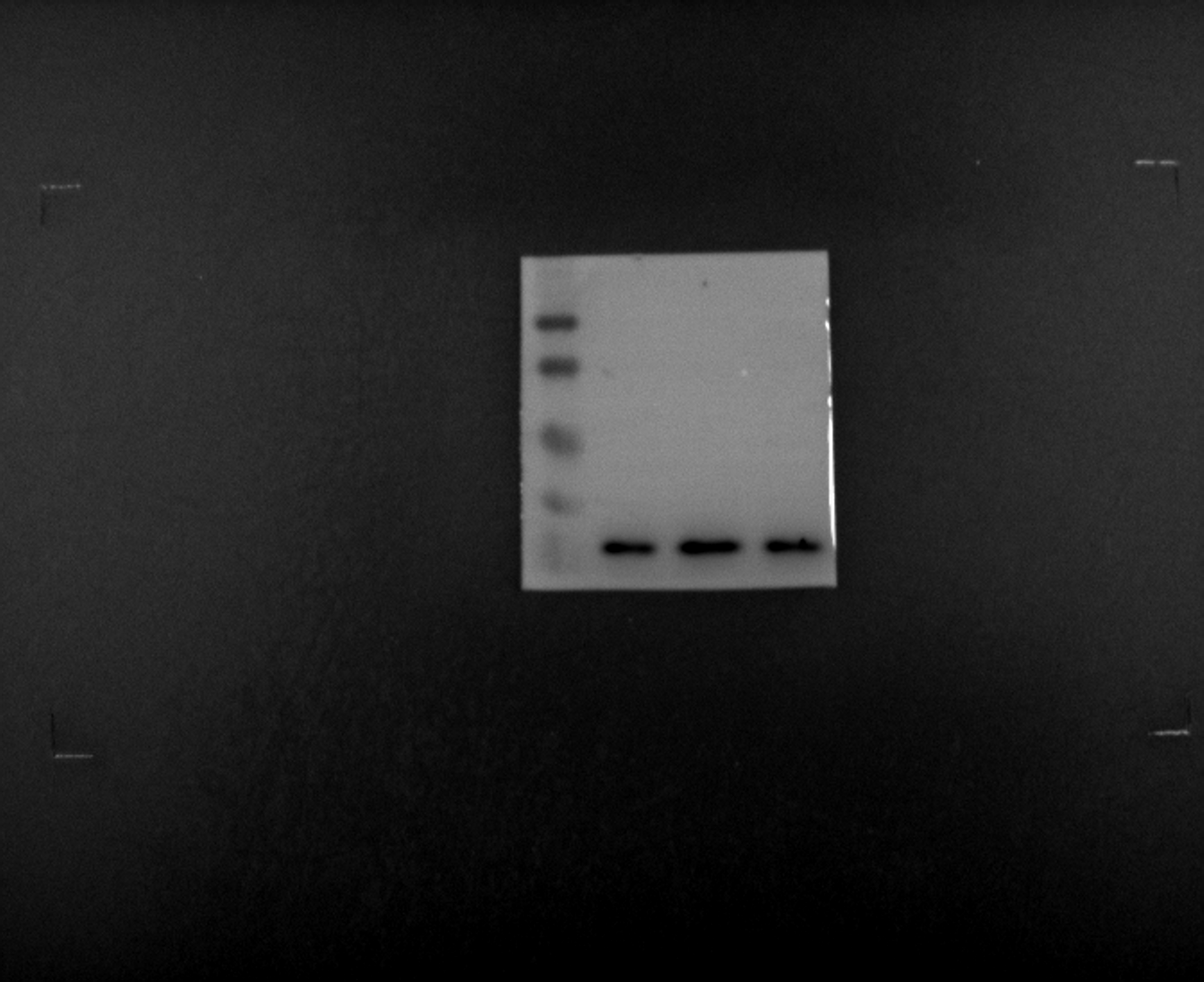

Supplement: Supplemental Information 4 [file peerj-12-17874-s004.zip › fig 2F/bax-2 (3).tif]

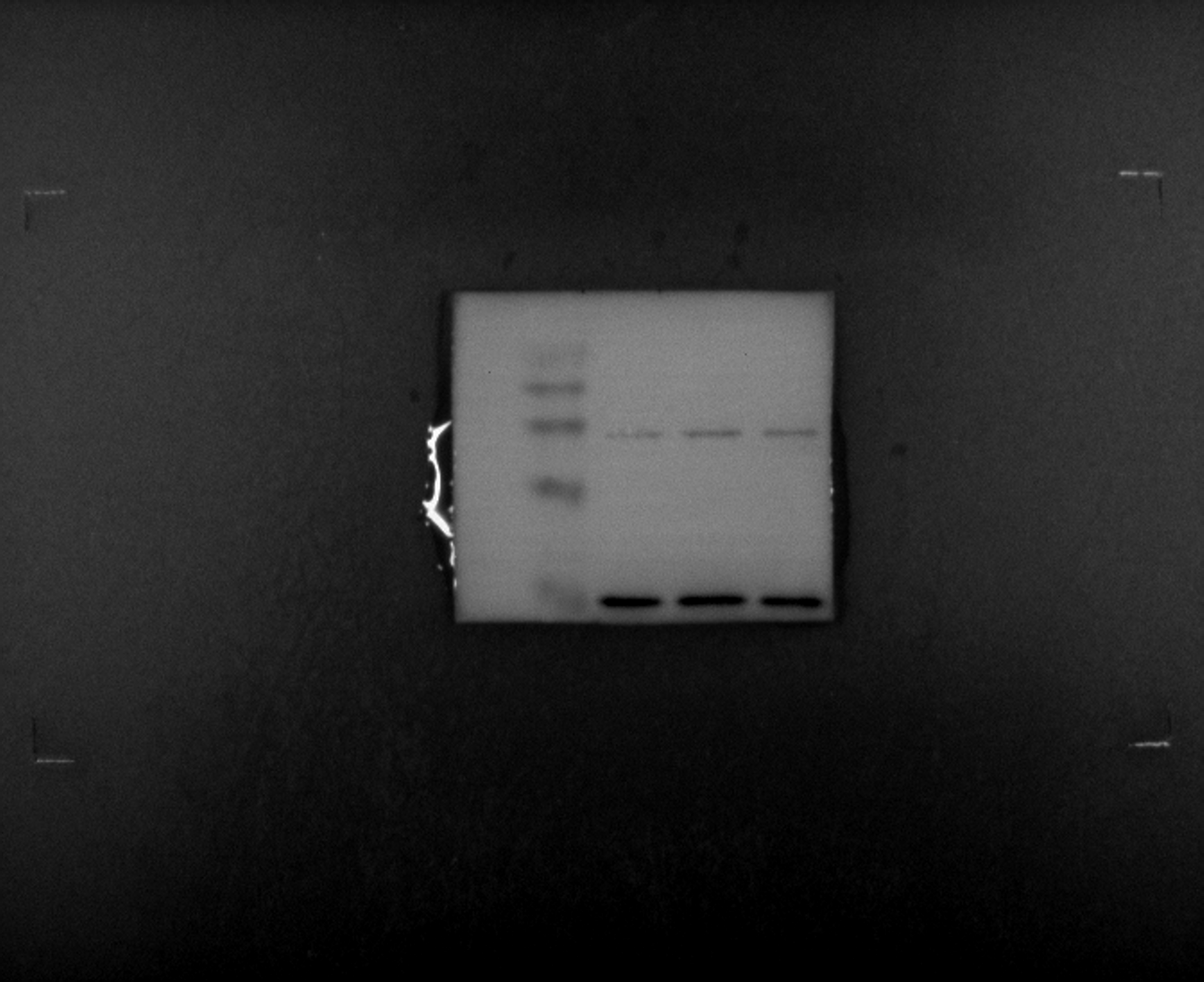

Supplement: Supplemental Information 4 [file peerj-12-17874-s004.zip › fig 2F/bax-3 (1).tif]

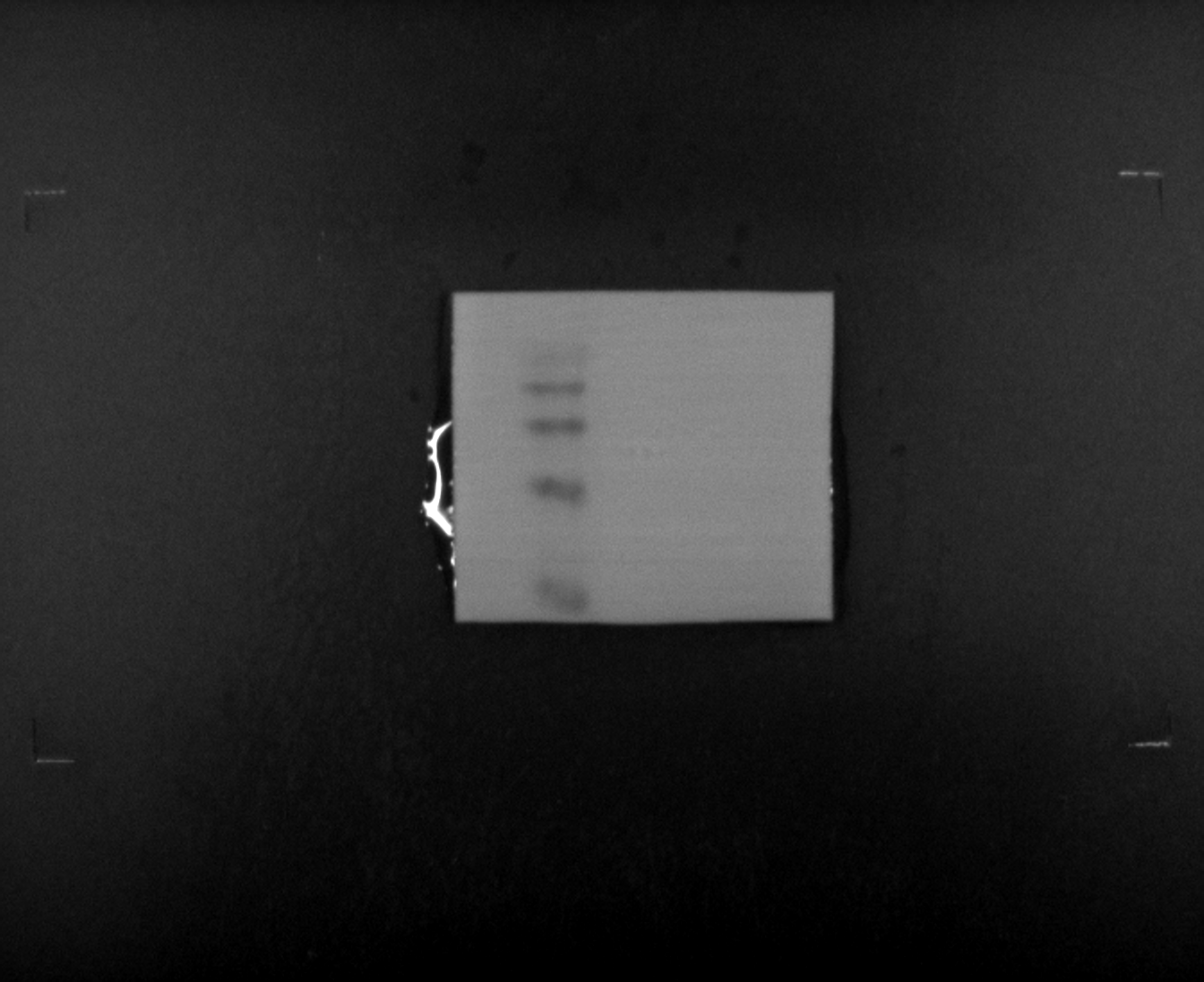

Supplement: Supplemental Information 4 [file peerj-12-17874-s004.zip › fig 2F/bax-3 (2).tif]

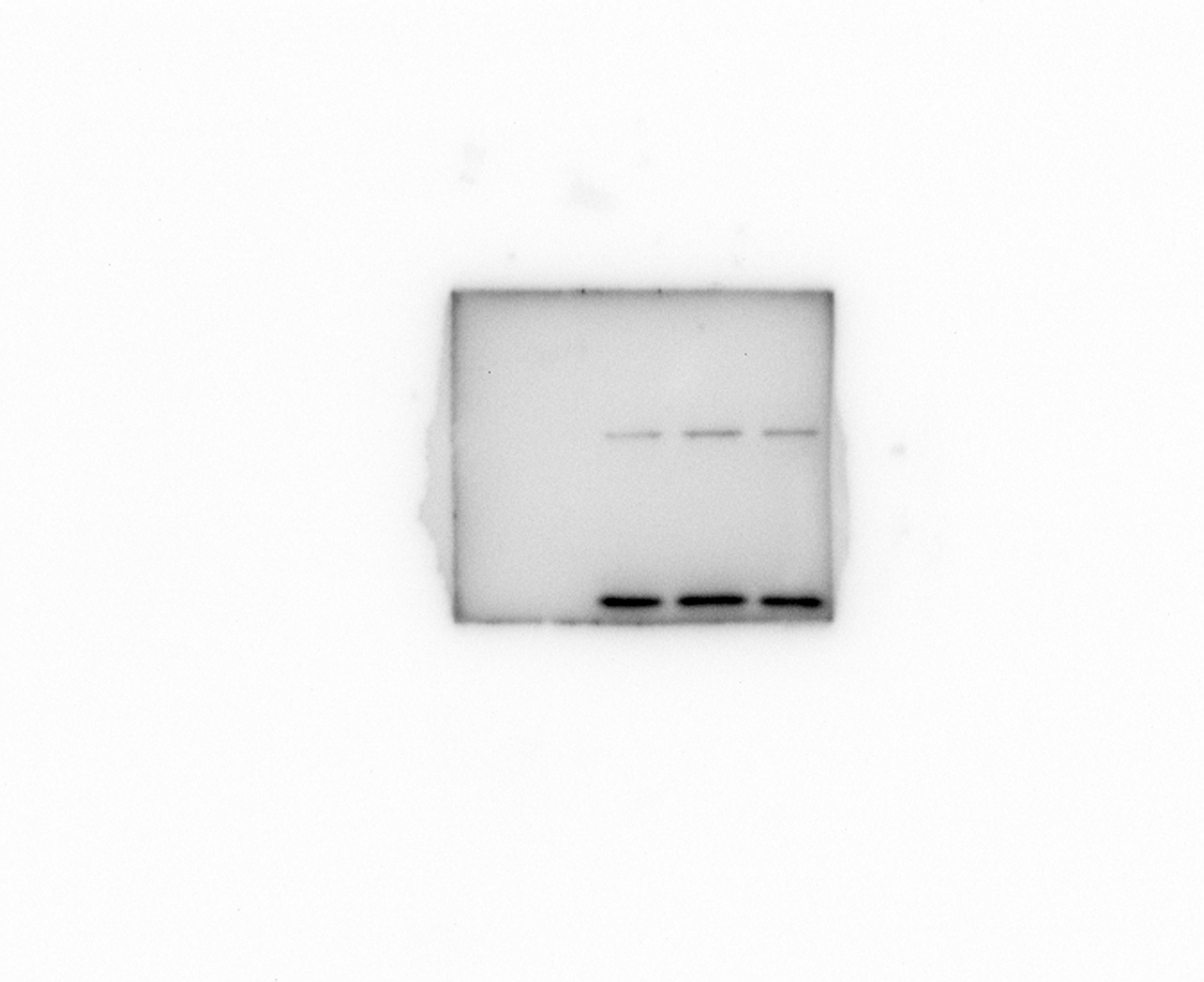

Supplement: Supplemental Information 4 [file peerj-12-17874-s004.zip › fig 2F/bax-3 (3).tif]

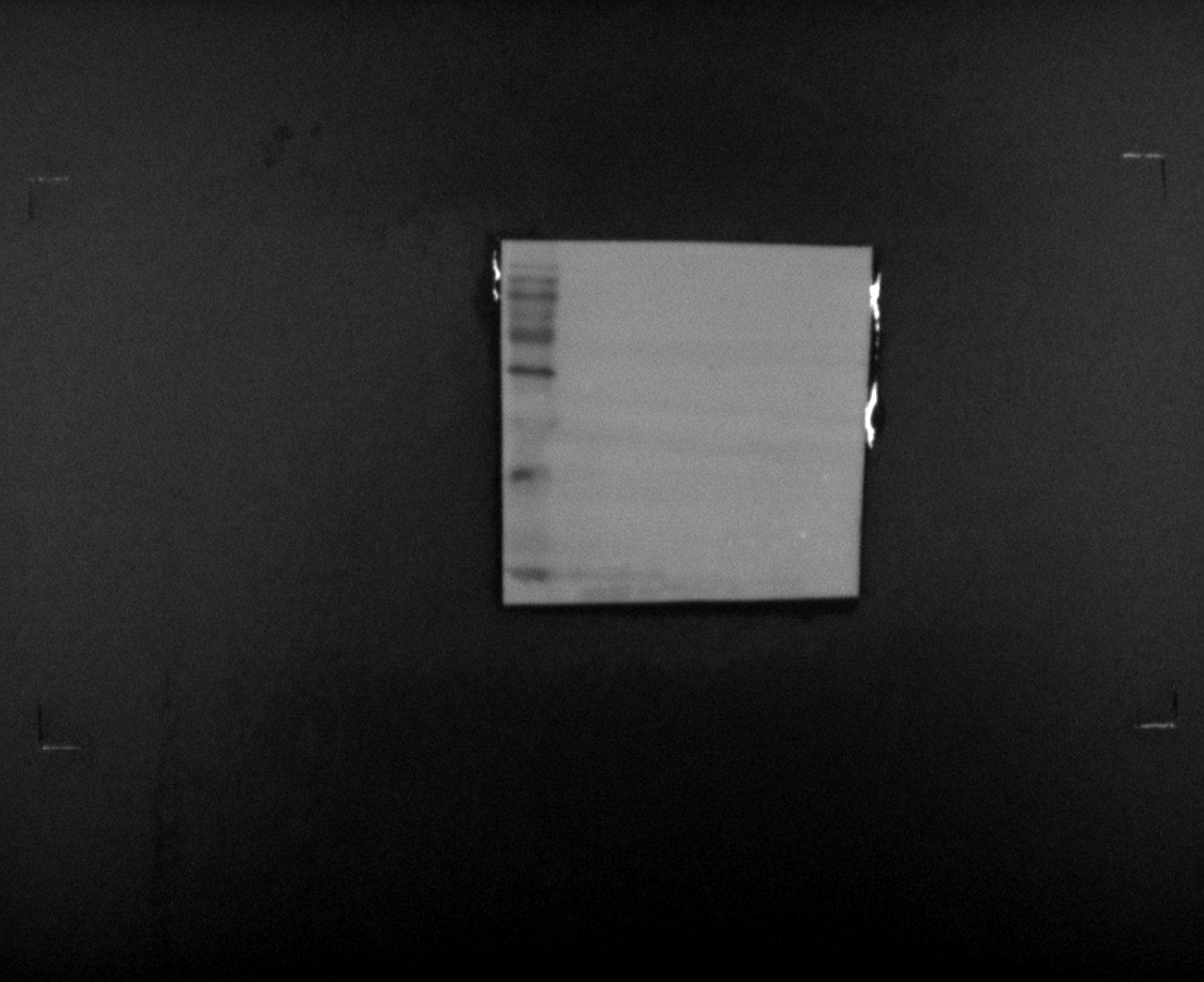

Supplement: Supplemental Information 4 [file peerj-12-17874-s004.zip › fig 2F/bcl2 (1).tif]

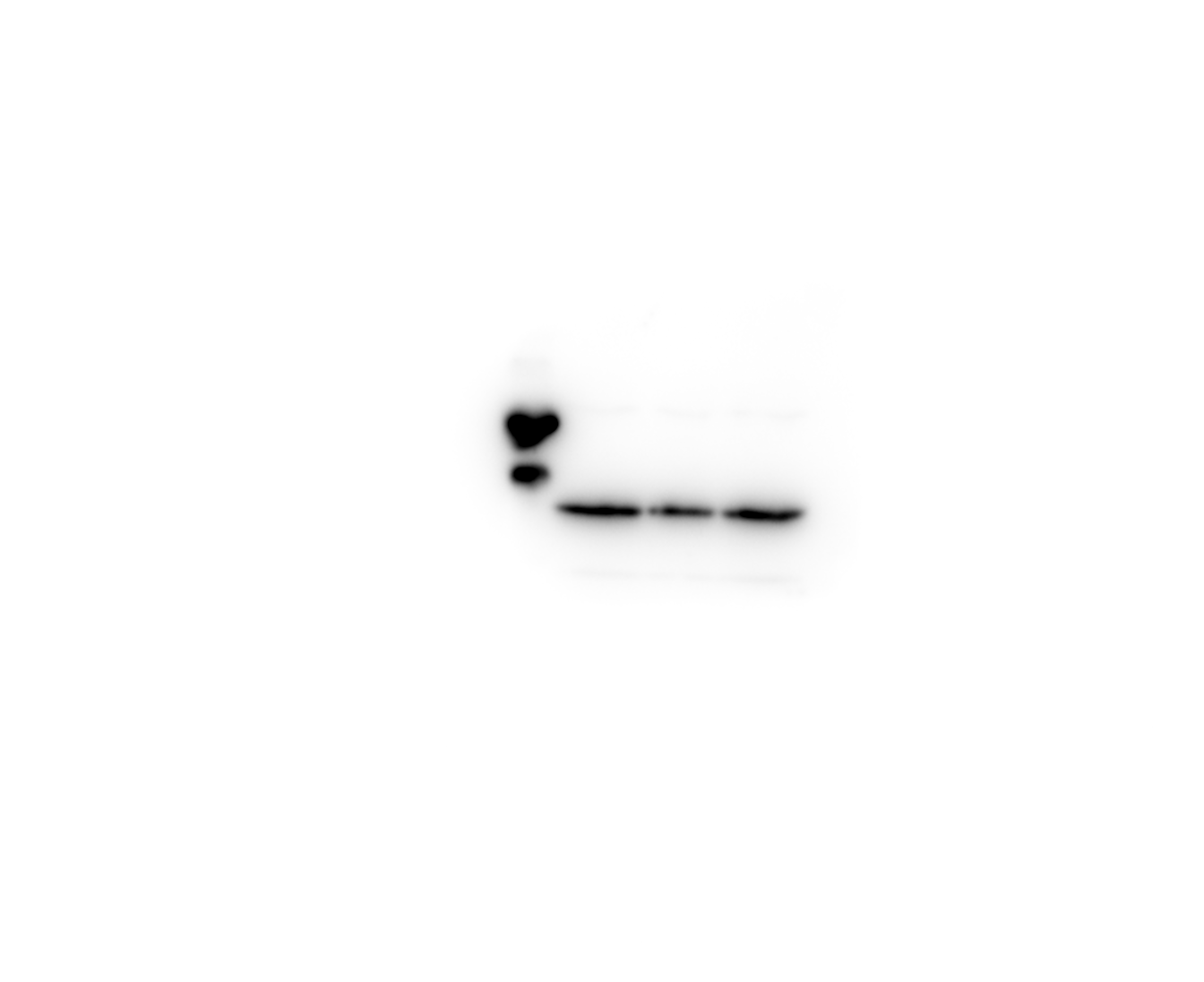

Supplement: Supplemental Information 4 [file peerj-12-17874-s004.zip › fig 2F/bcl2 (2).tif]

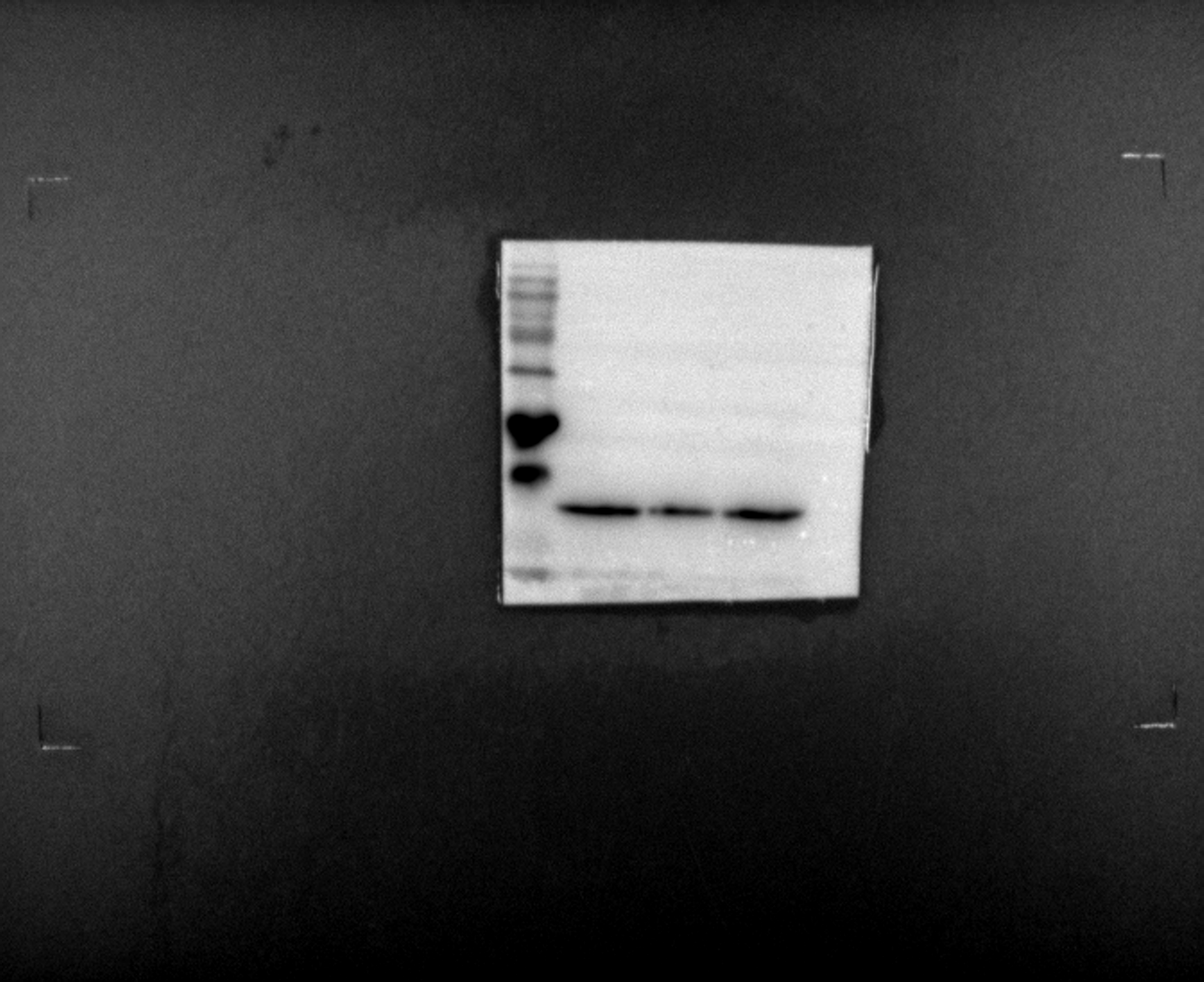

Supplement: Supplemental Information 4 [file peerj-12-17874-s004.zip › fig 2F/bcl2 (3).tif]

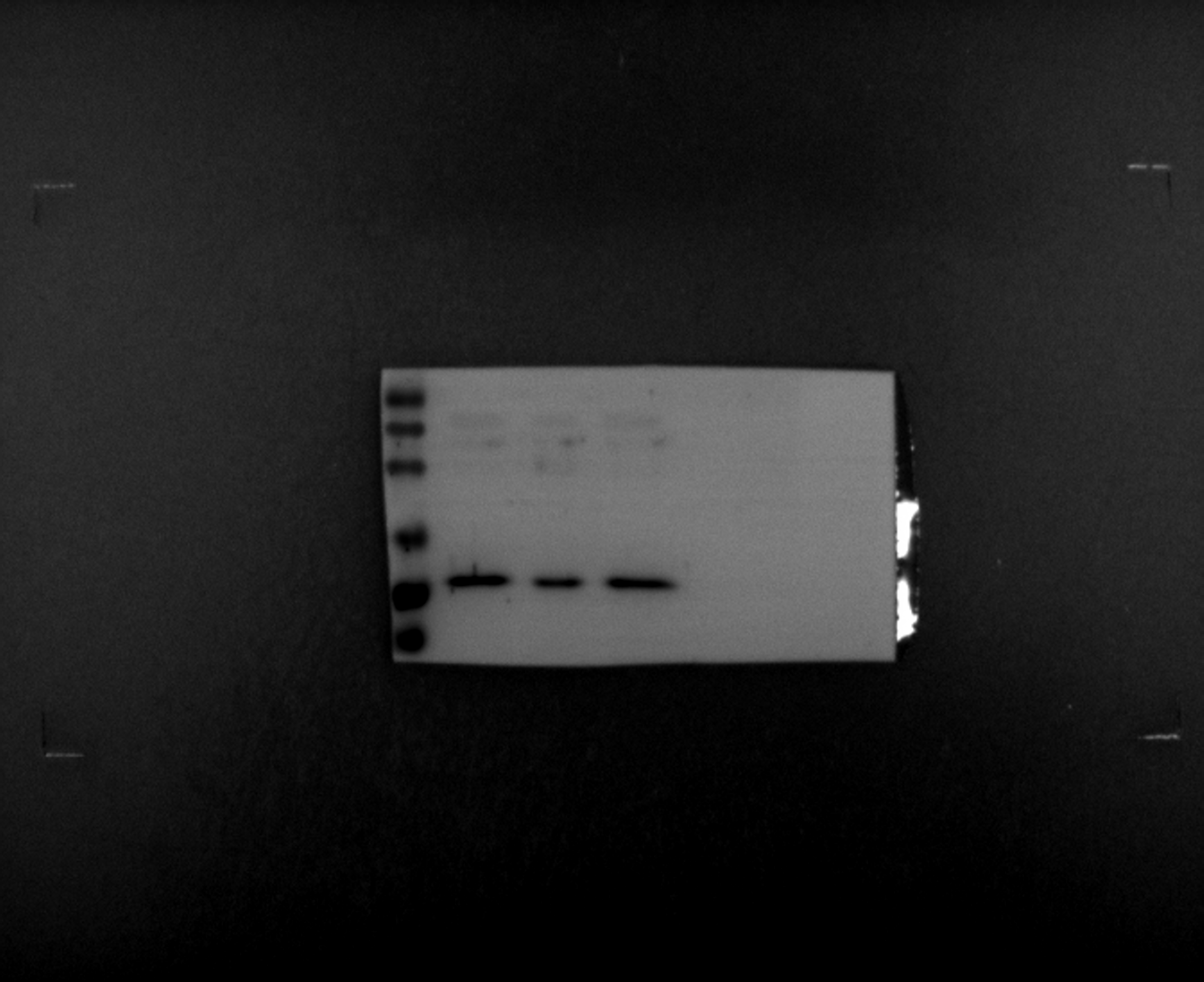

Supplement: Supplemental Information 4 [file peerj-12-17874-s004.zip › fig 2F/bcl2-2 (1).tif]

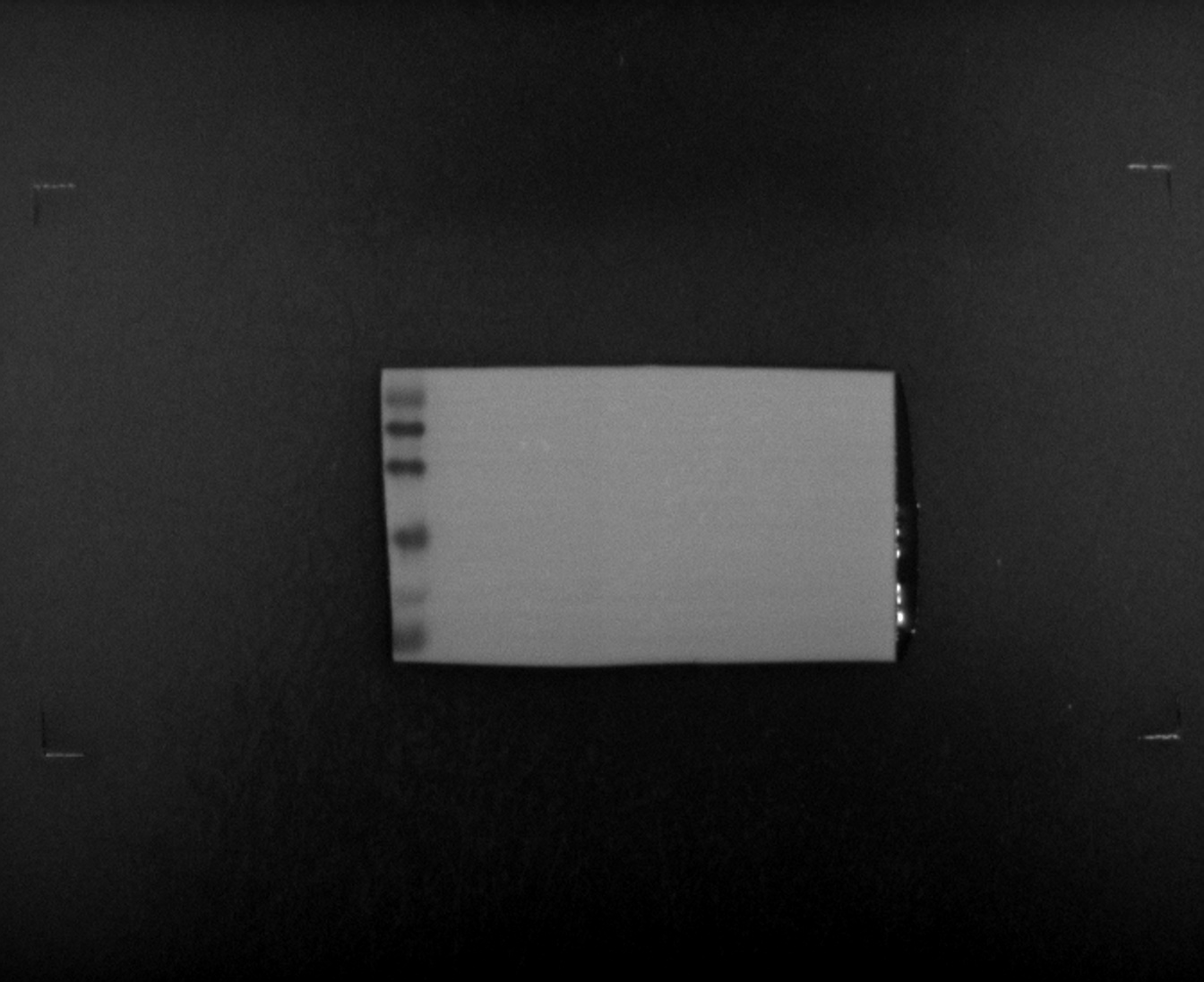

Supplement: Supplemental Information 4 [file peerj-12-17874-s004.zip › fig 2F/bcl2-2 (2).tif]

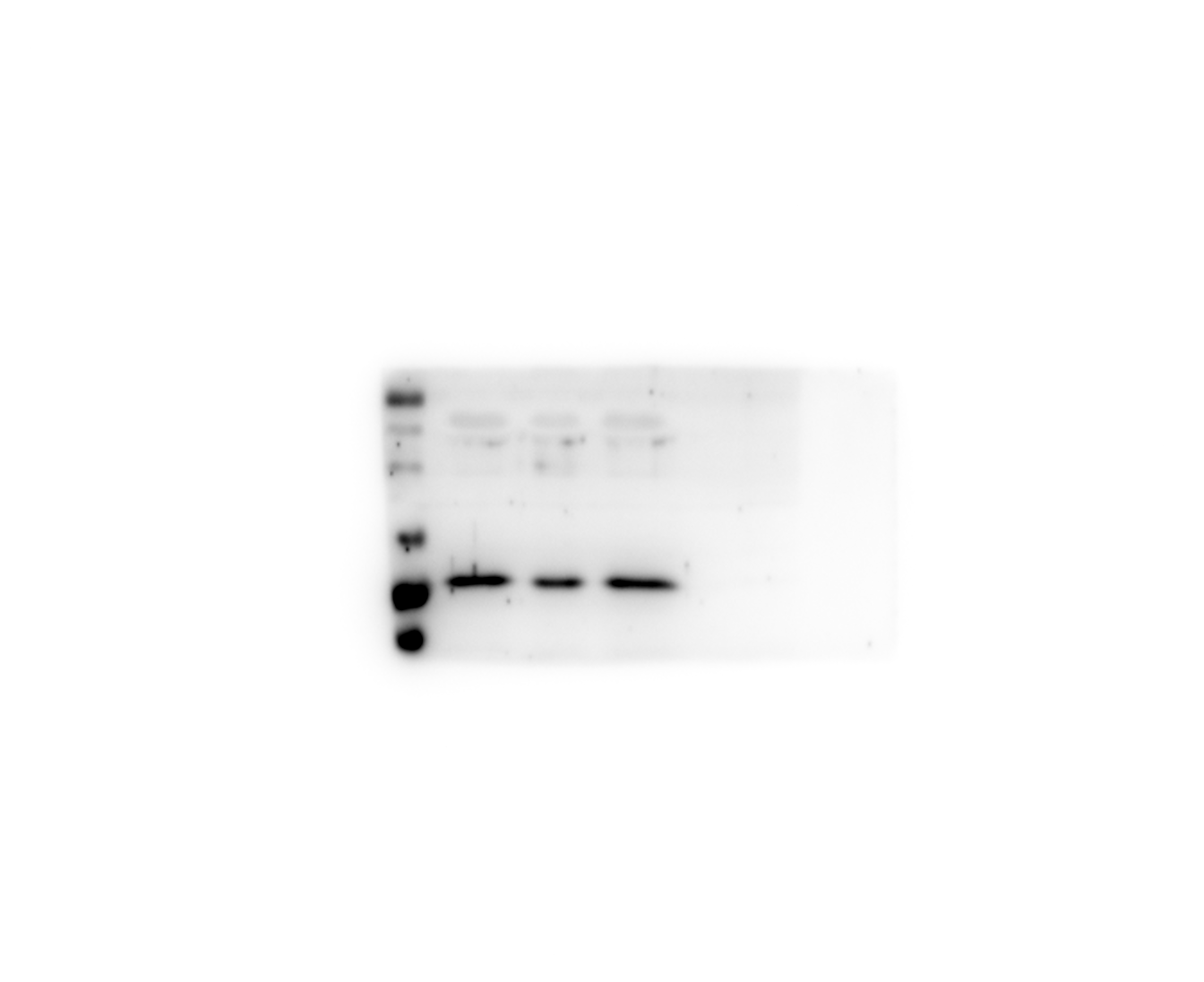

Supplement: Supplemental Information 4 [file peerj-12-17874-s004.zip › fig 2F/bcl2-2 (3).tif]

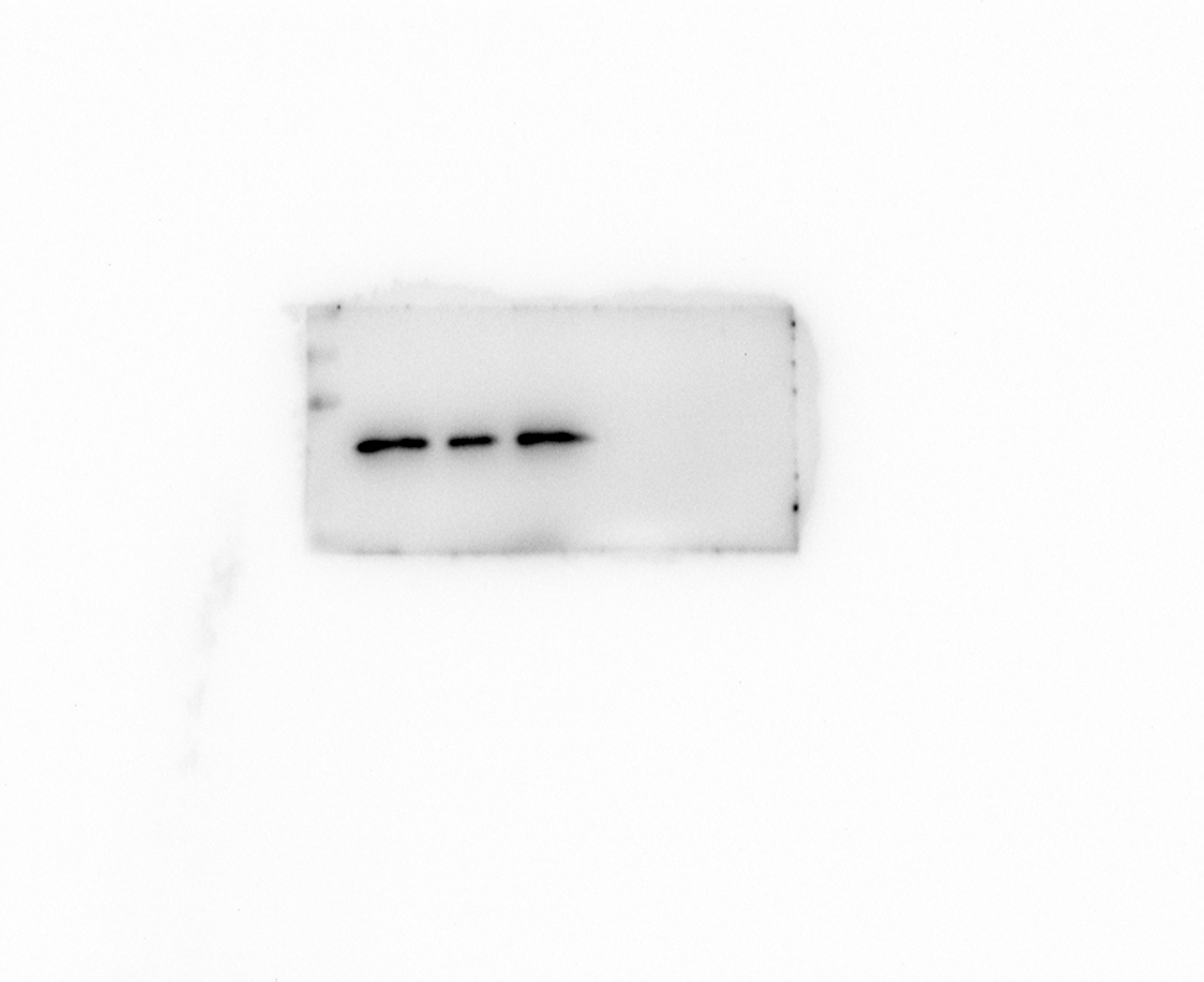

Supplement: Supplemental Information 4 [file peerj-12-17874-s004.zip › fig 2F/bcl2-3 (1).tif]

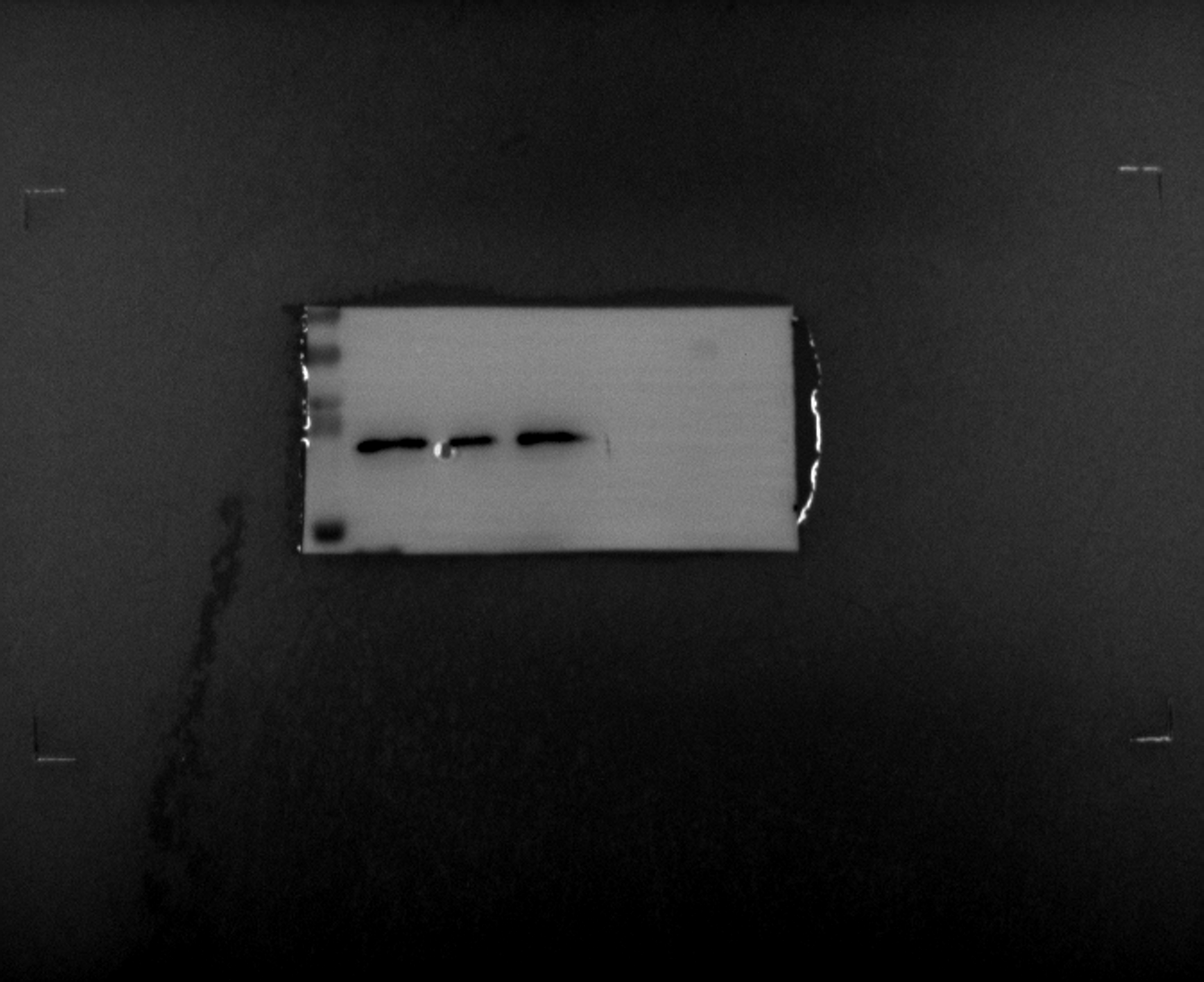

Supplement: Supplemental Information 4 [file peerj-12-17874-s004.zip › fig 2F/bcl2-3 (2).tif]

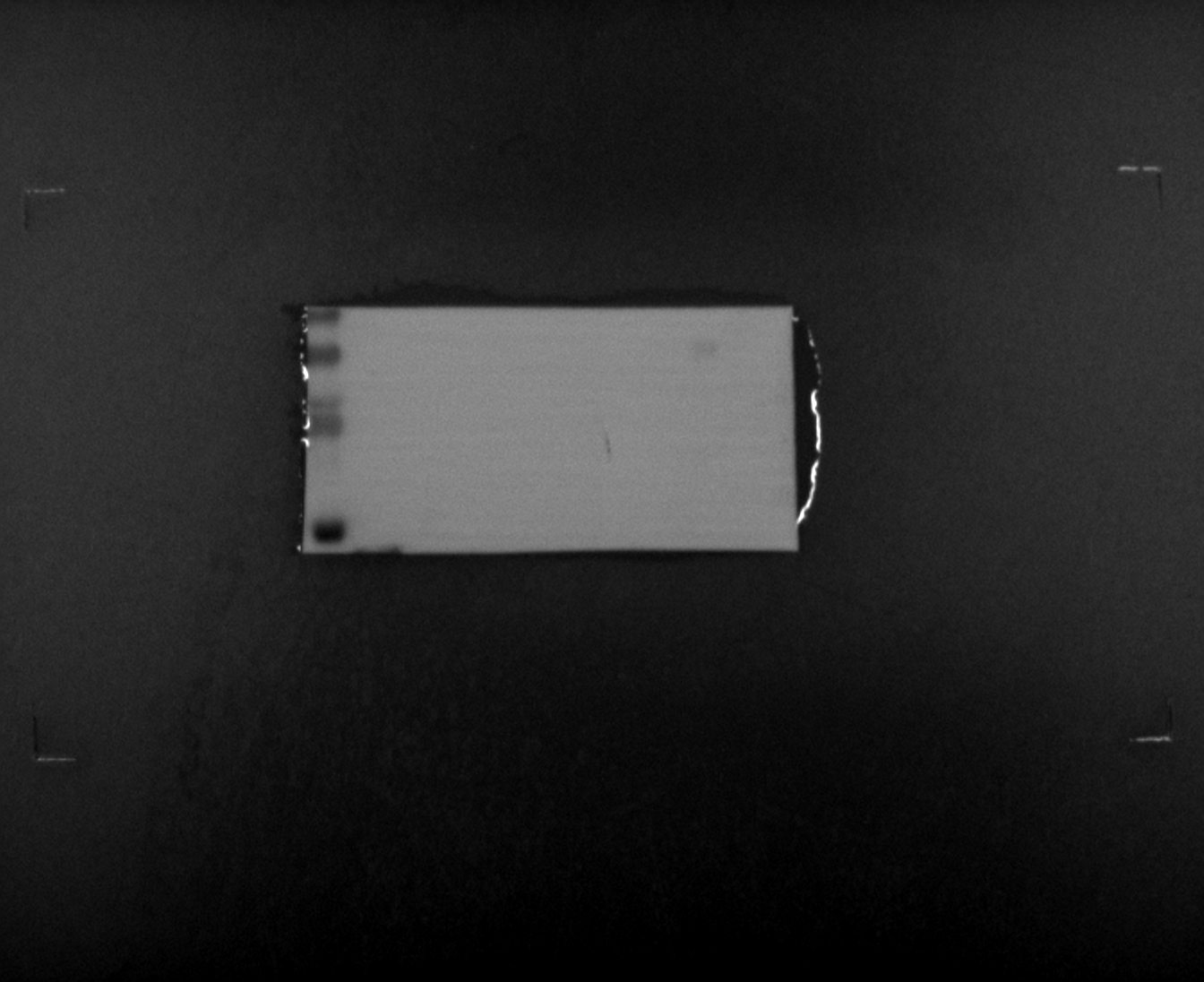

Supplement: Supplemental Information 4 [file peerj-12-17874-s004.zip › fig 2F/bcl2-3 (3).tif]

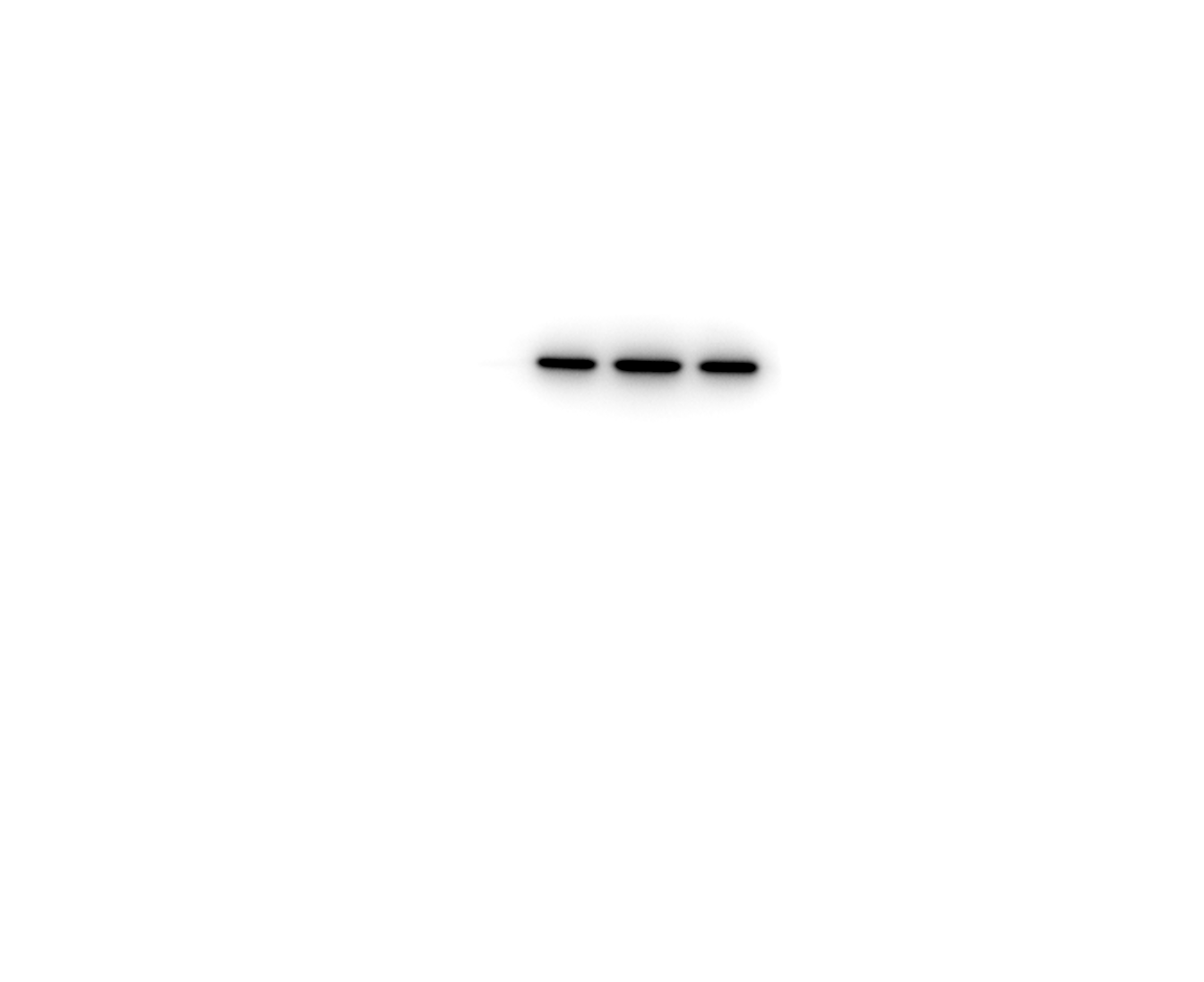

Supplement: Supplemental Information 4 [file peerj-12-17874-s004.zip › fig 2F/Caspase 1-2 (1).tif]
