# Supplementary figures and images for: miR-605-3p may affect caerulein-induced ductal cell injury and pyroptosis in acute pancreatitis by targeting the DUOX2/NLRP3/NF-κB pathway (part 2 of 3)
Source: PeerJ. 2024 Aug 30;12:e17874. doi: 10.7717/peerj.17874 (PMC11368084; doi:10.7717/peerj.17874)

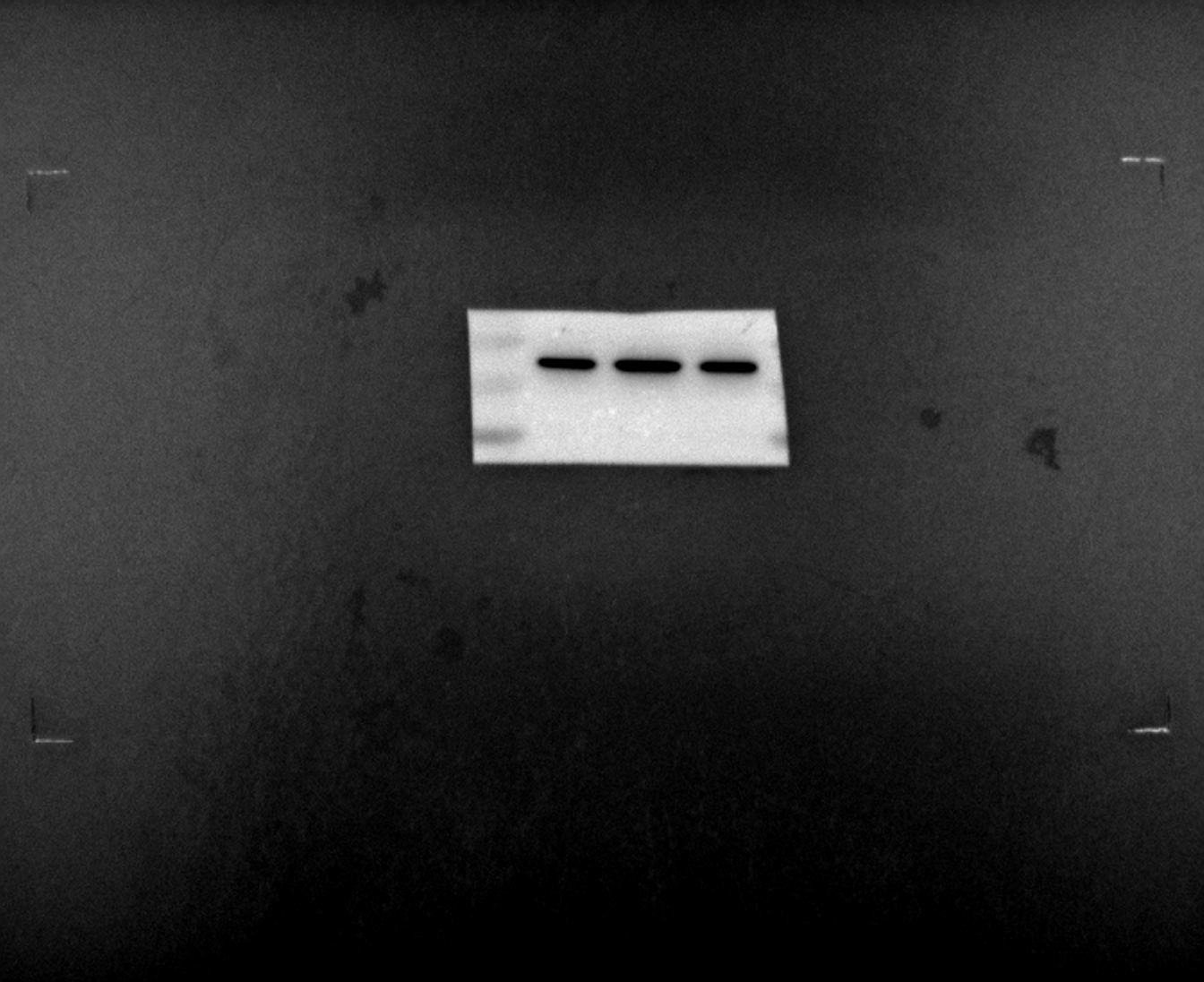

Supplement: Supplemental Information 4 [file peerj-12-17874-s004.zip › fig 2F/Caspase 1-2 (2).tif]

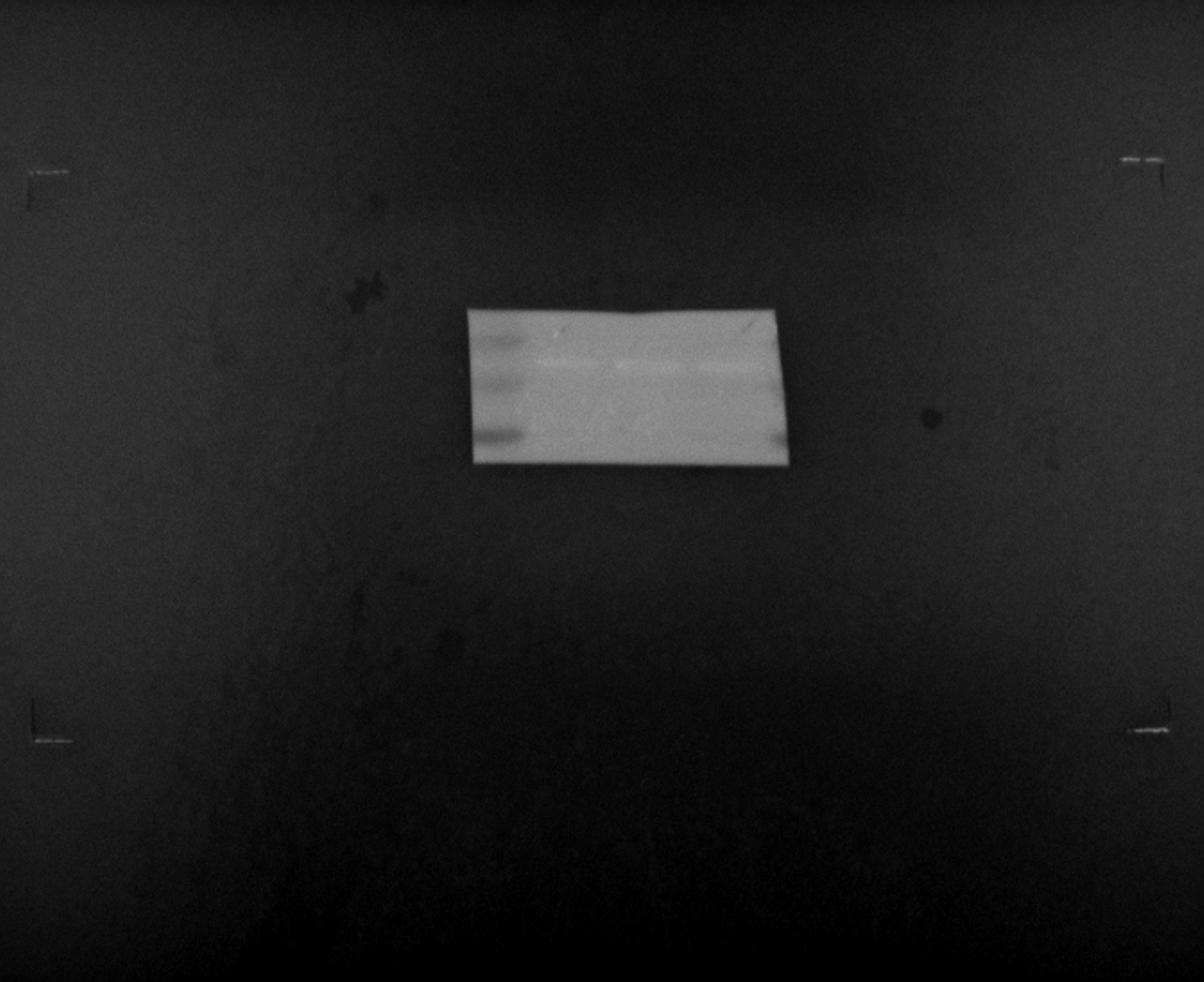

Supplement: Supplemental Information 4 [file peerj-12-17874-s004.zip › fig 2F/Caspase 1-2 (3).tif]

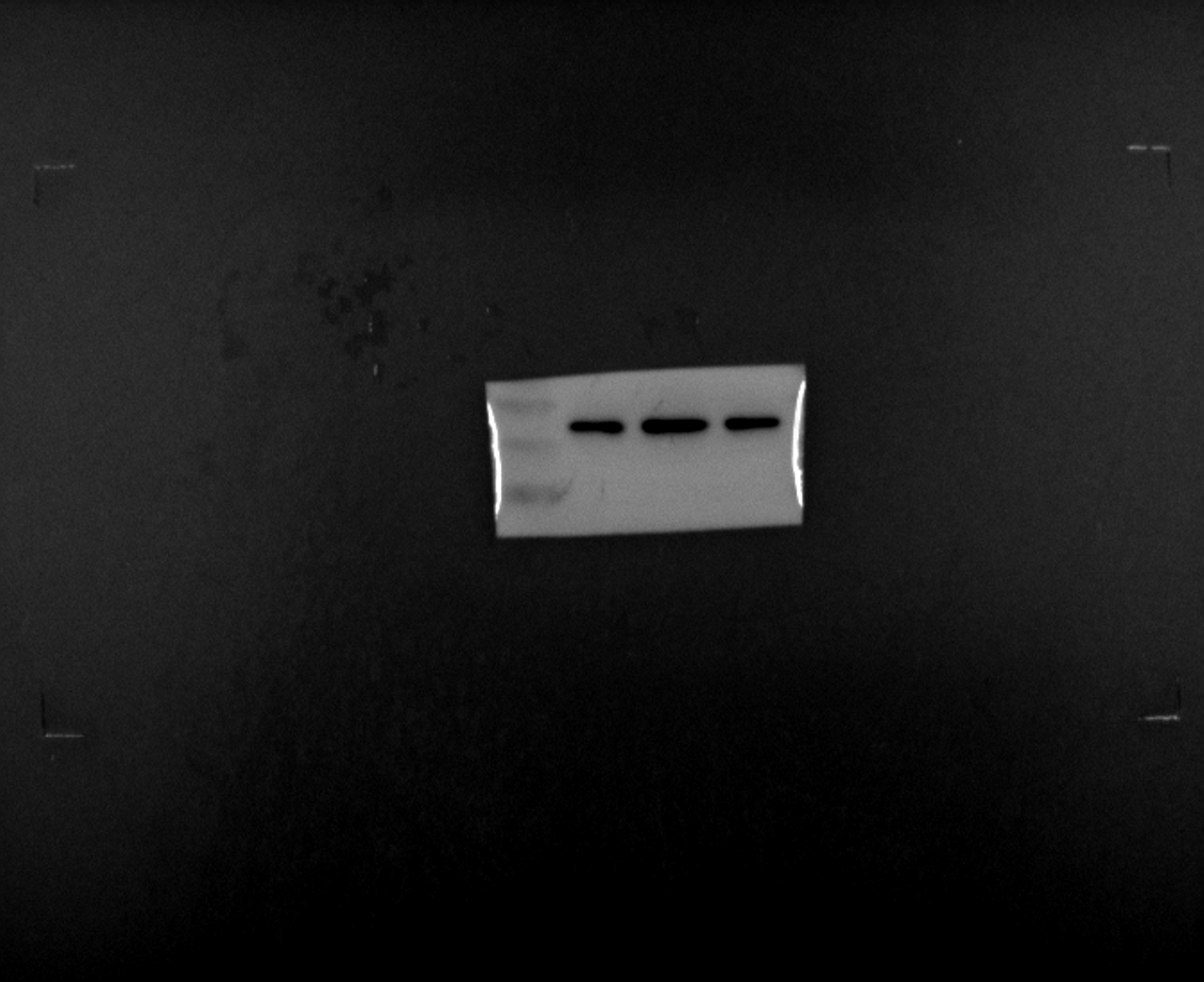

Supplement: Supplemental Information 4 [file peerj-12-17874-s004.zip › fig 2F/caspase 1-3 (1).tif]

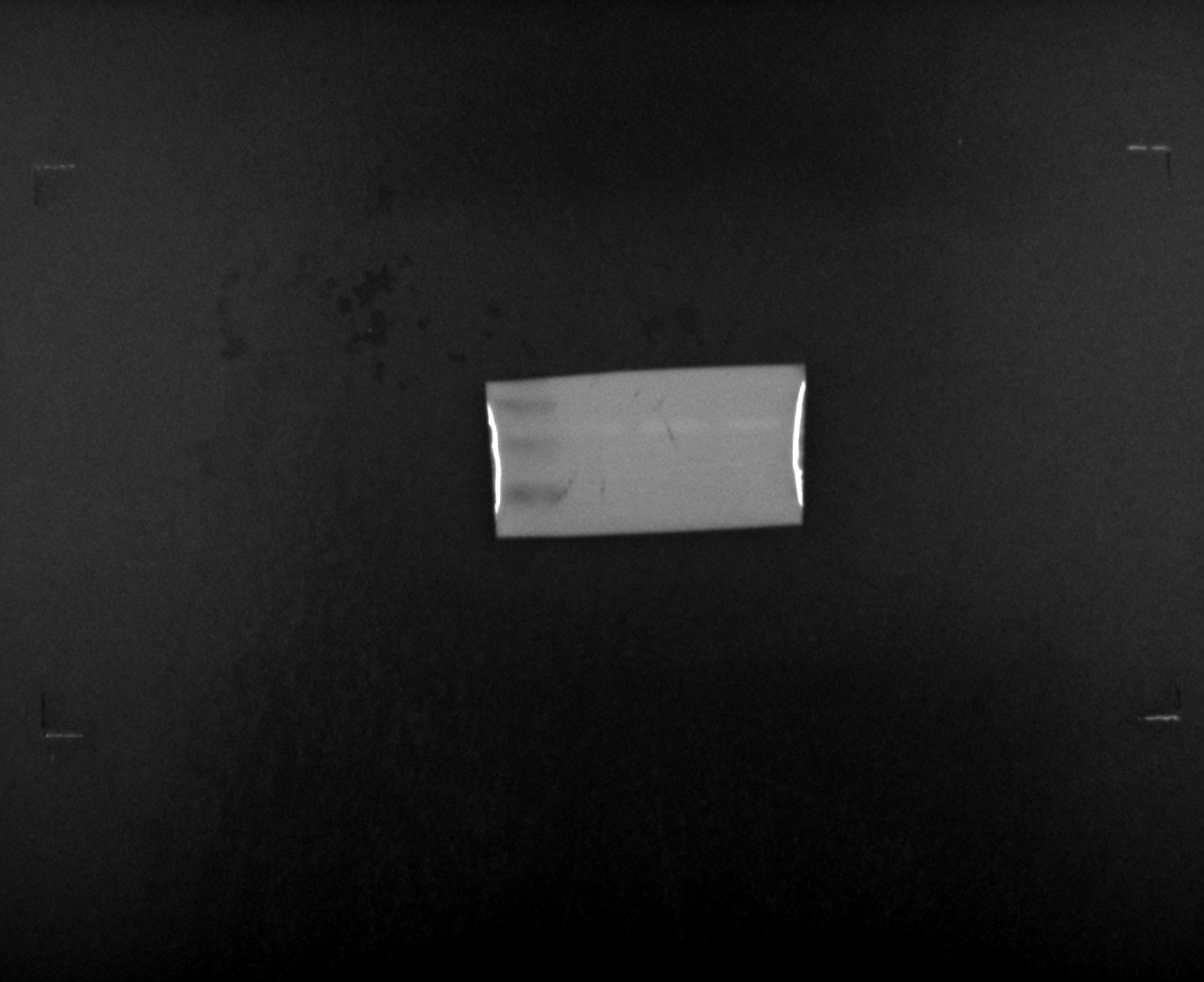

Supplement: Supplemental Information 4 [file peerj-12-17874-s004.zip › fig 2F/caspase 1-3 (2).tif]

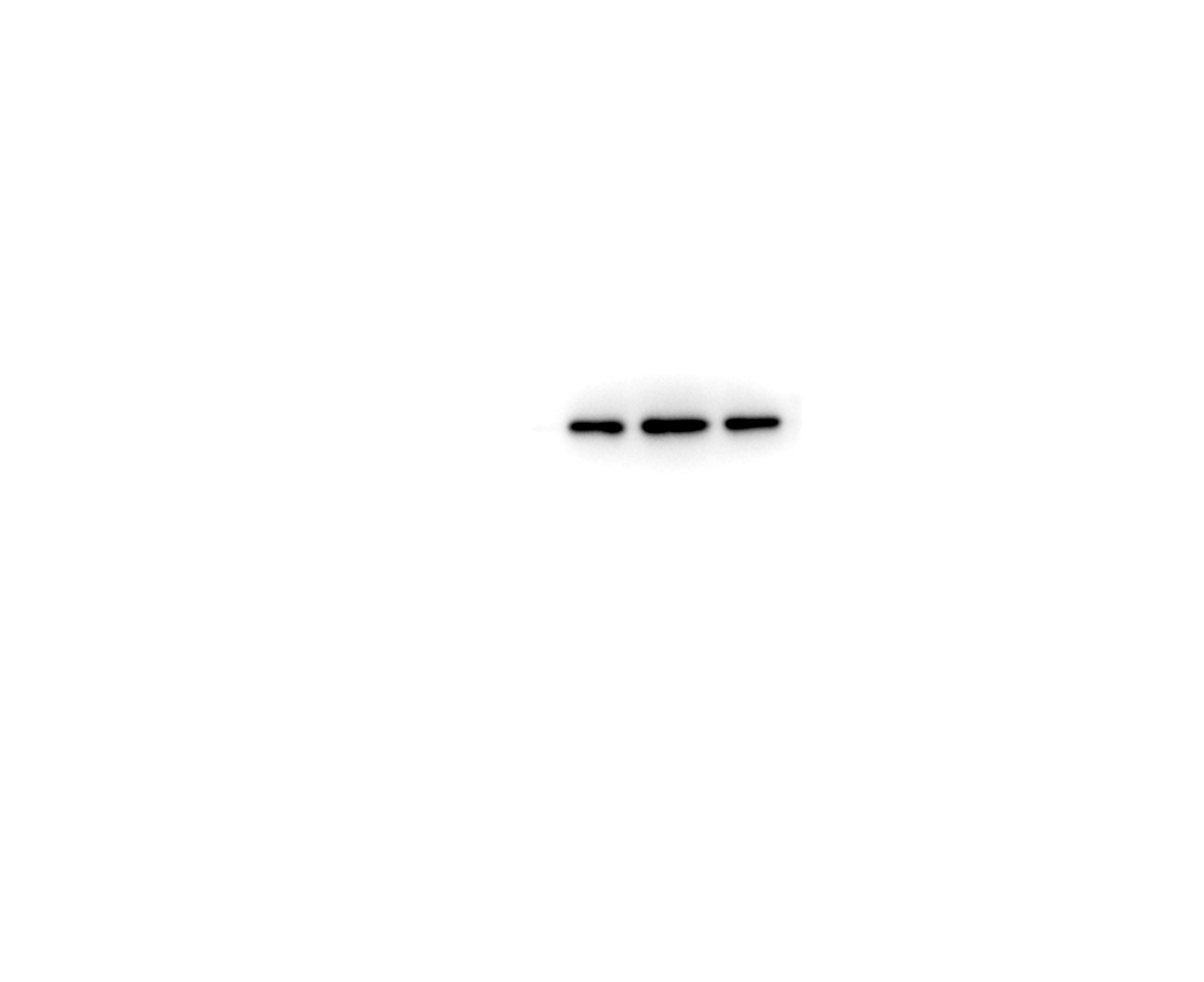

Supplement: Supplemental Information 4 [file peerj-12-17874-s004.zip › fig 2F/caspase 1-3 (3).tif]

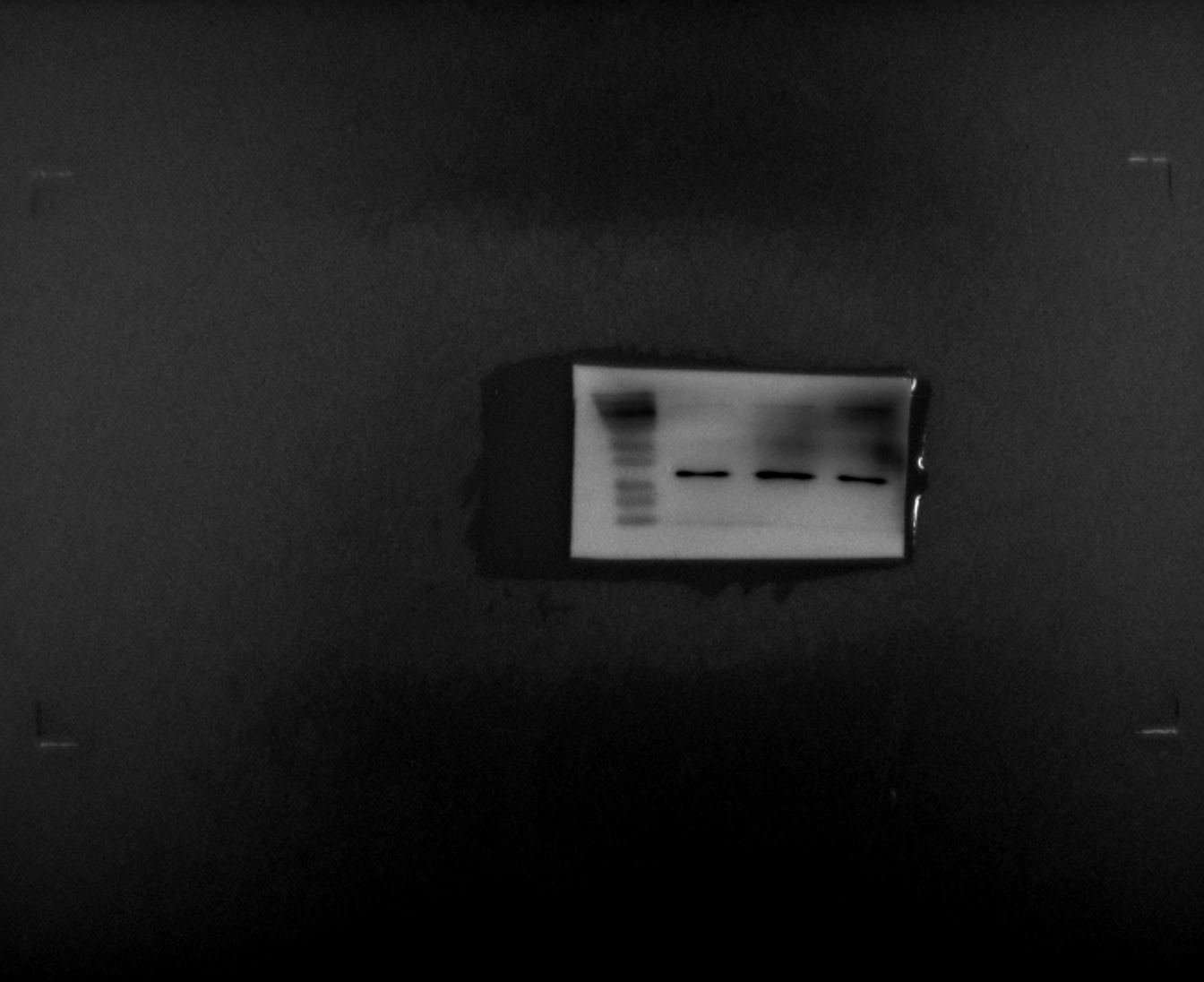

Supplement: Supplemental Information 4 [file peerj-12-17874-s004.zip › fig 2F/CL-CASAPSE1 (1).tif]

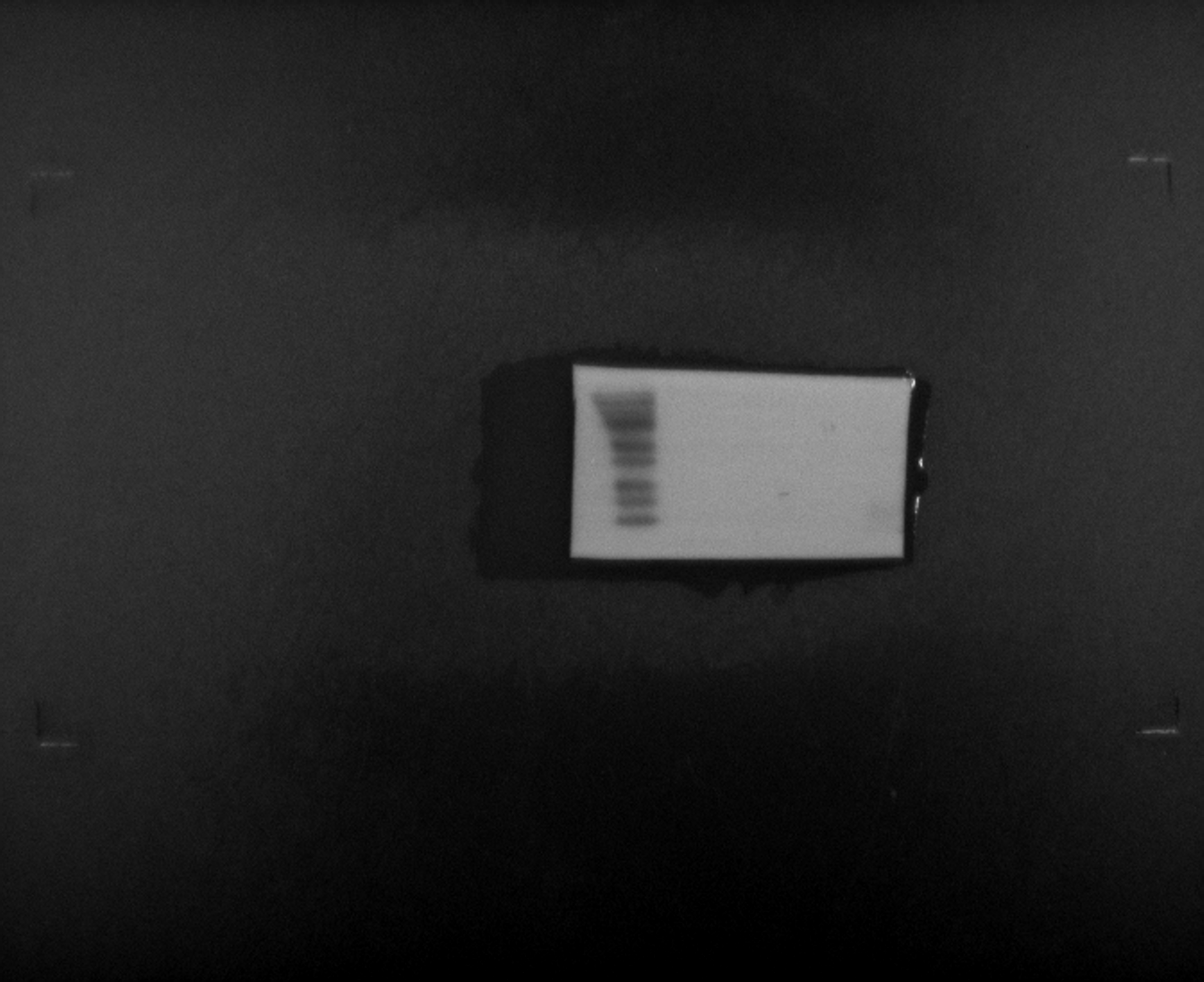

Supplement: Supplemental Information 4 [file peerj-12-17874-s004.zip › fig 2F/CL-CASAPSE1 (2).tif]

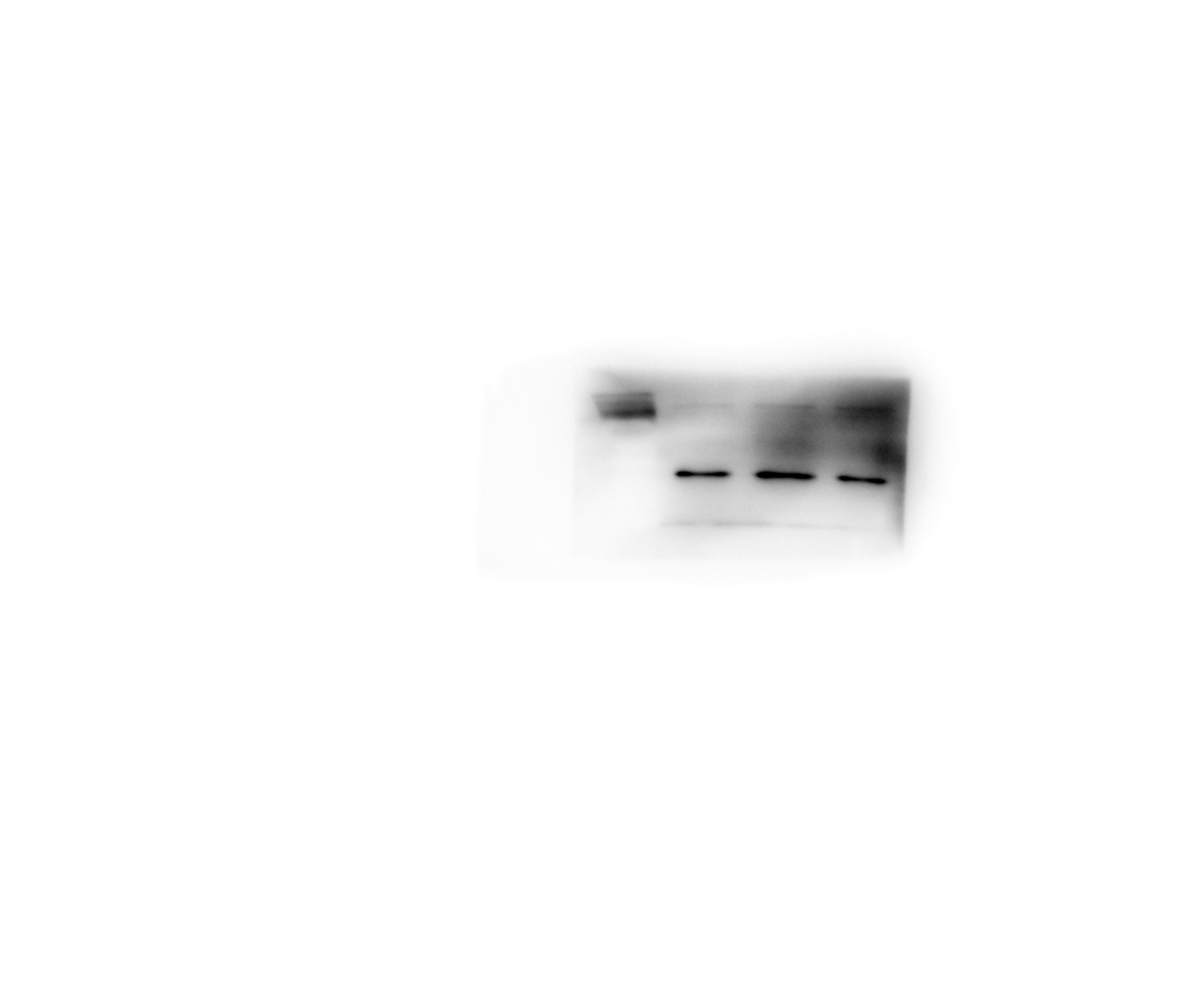

Supplement: Supplemental Information 4 [file peerj-12-17874-s004.zip › fig 2F/CL-CASAPSE1 (3).tif]

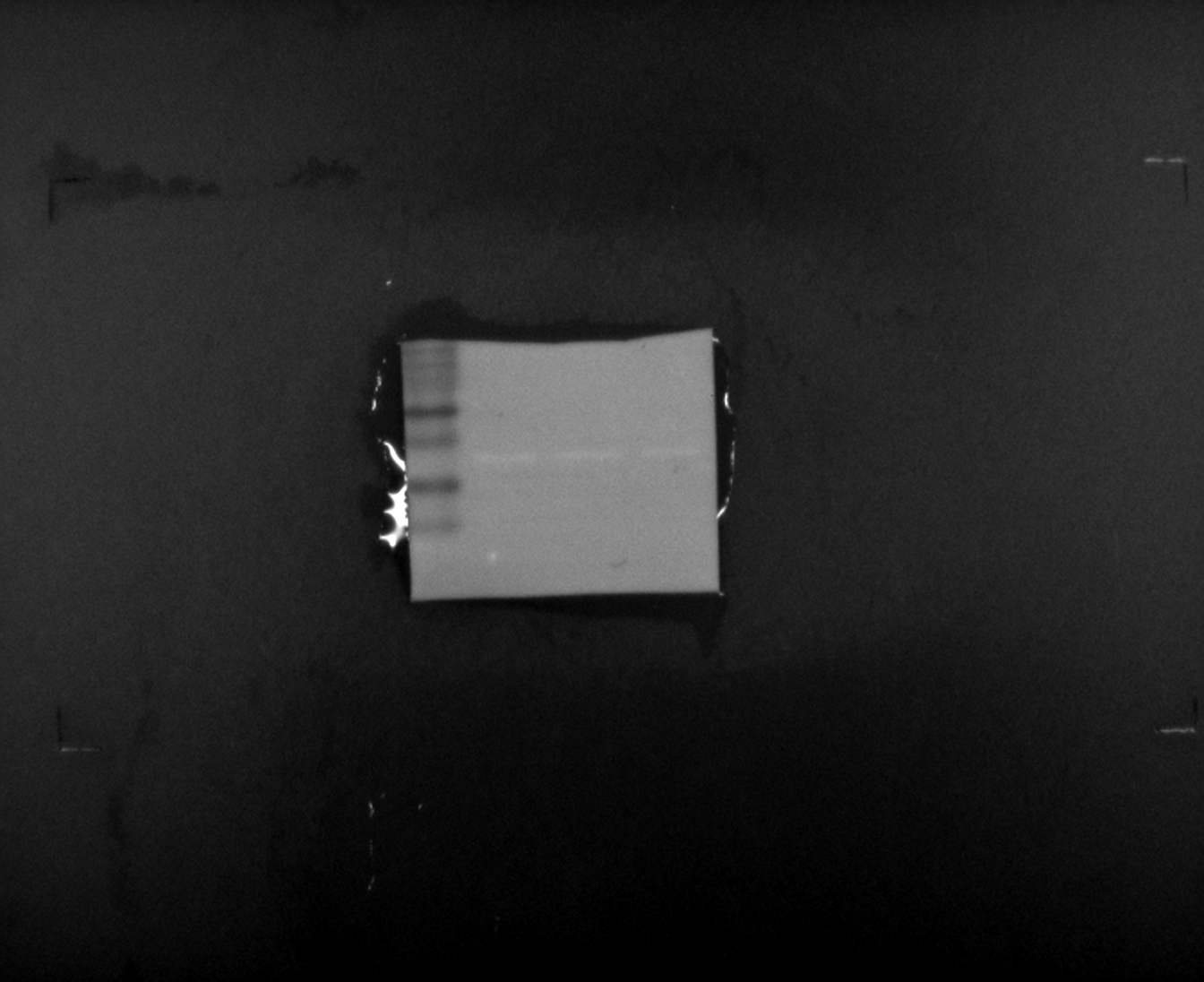

Supplement: Supplemental Information 4 [file peerj-12-17874-s004.zip › fig 2F/GAPDH (1).tif]

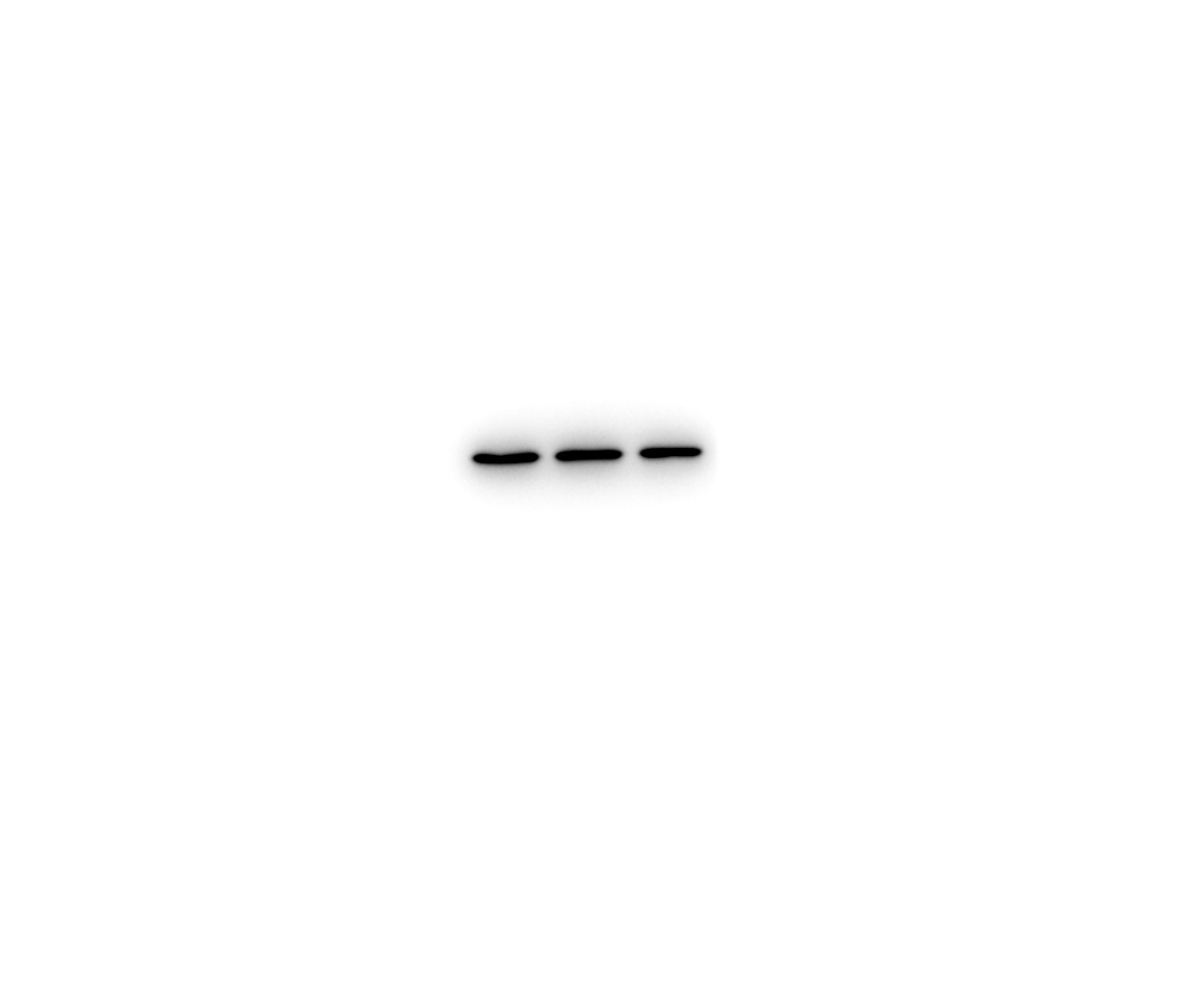

Supplement: Supplemental Information 4 [file peerj-12-17874-s004.zip › fig 2F/GAPDH (2).tif]

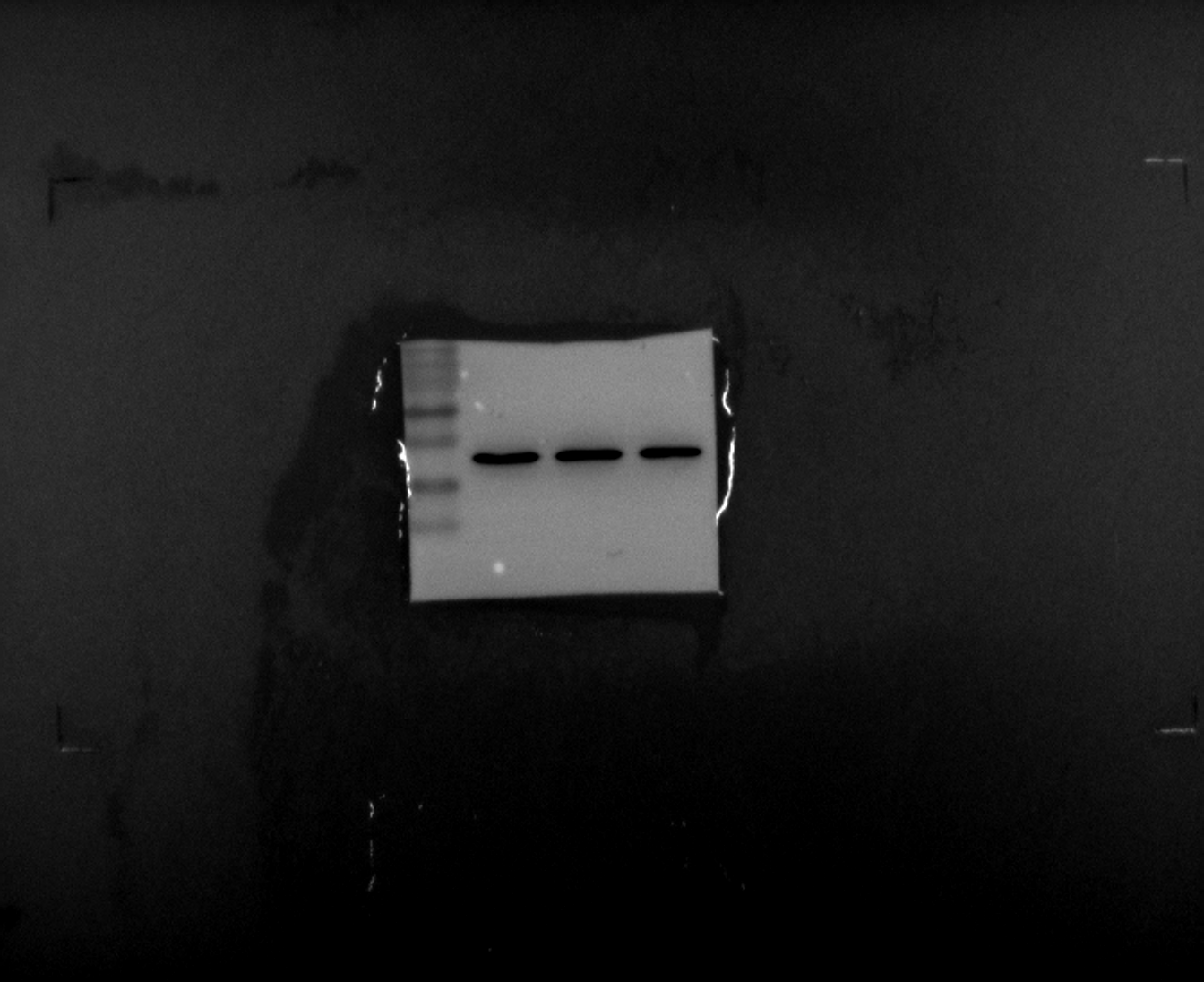

Supplement: Supplemental Information 4 [file peerj-12-17874-s004.zip › fig 2F/GAPDH (3).tif]

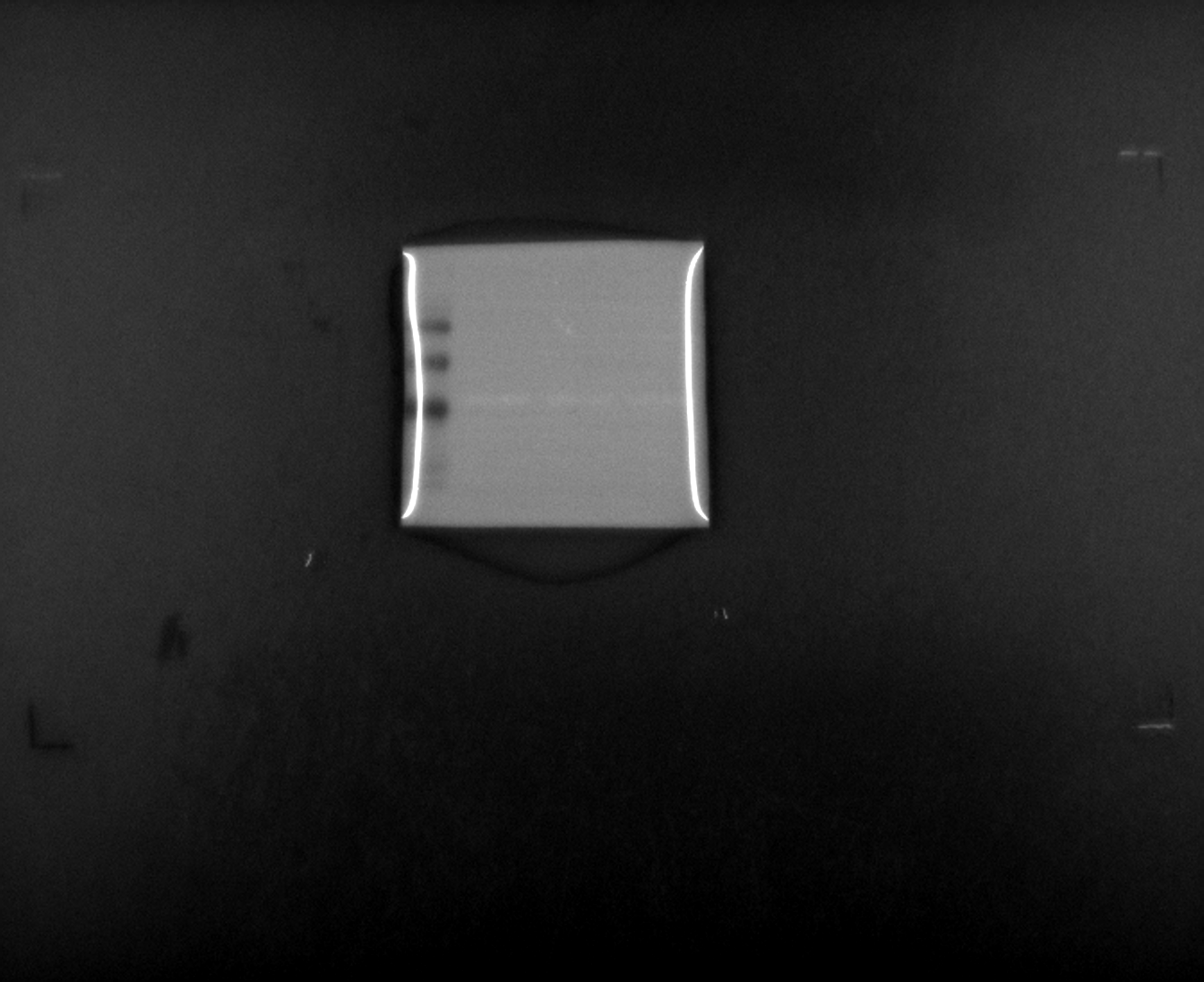

Supplement: Supplemental Information 4 [file peerj-12-17874-s004.zip › fig 2F/GAPDH-1 (1).tif]

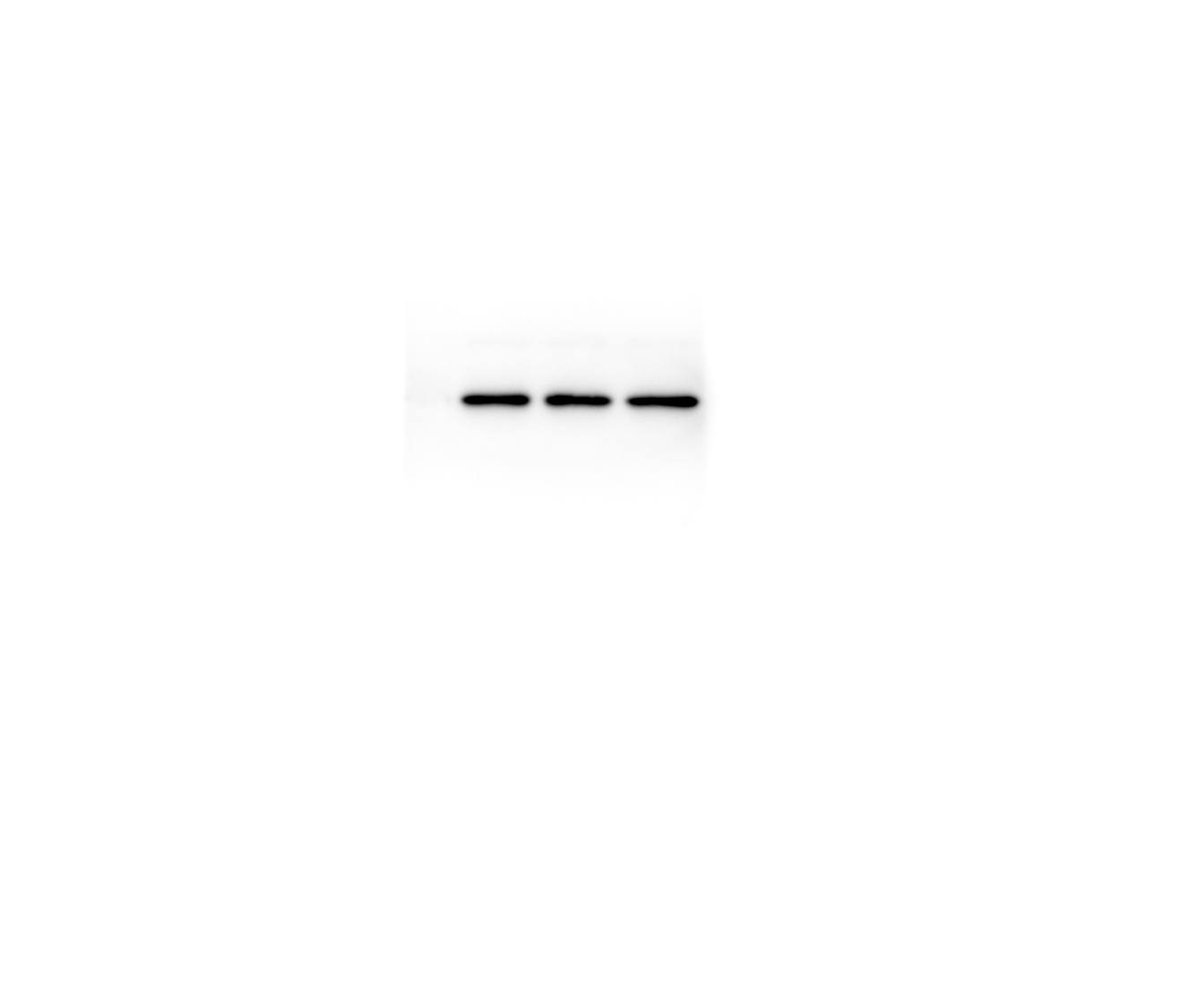

Supplement: Supplemental Information 4 [file peerj-12-17874-s004.zip › fig 2F/GAPDH-1 (2).tif]

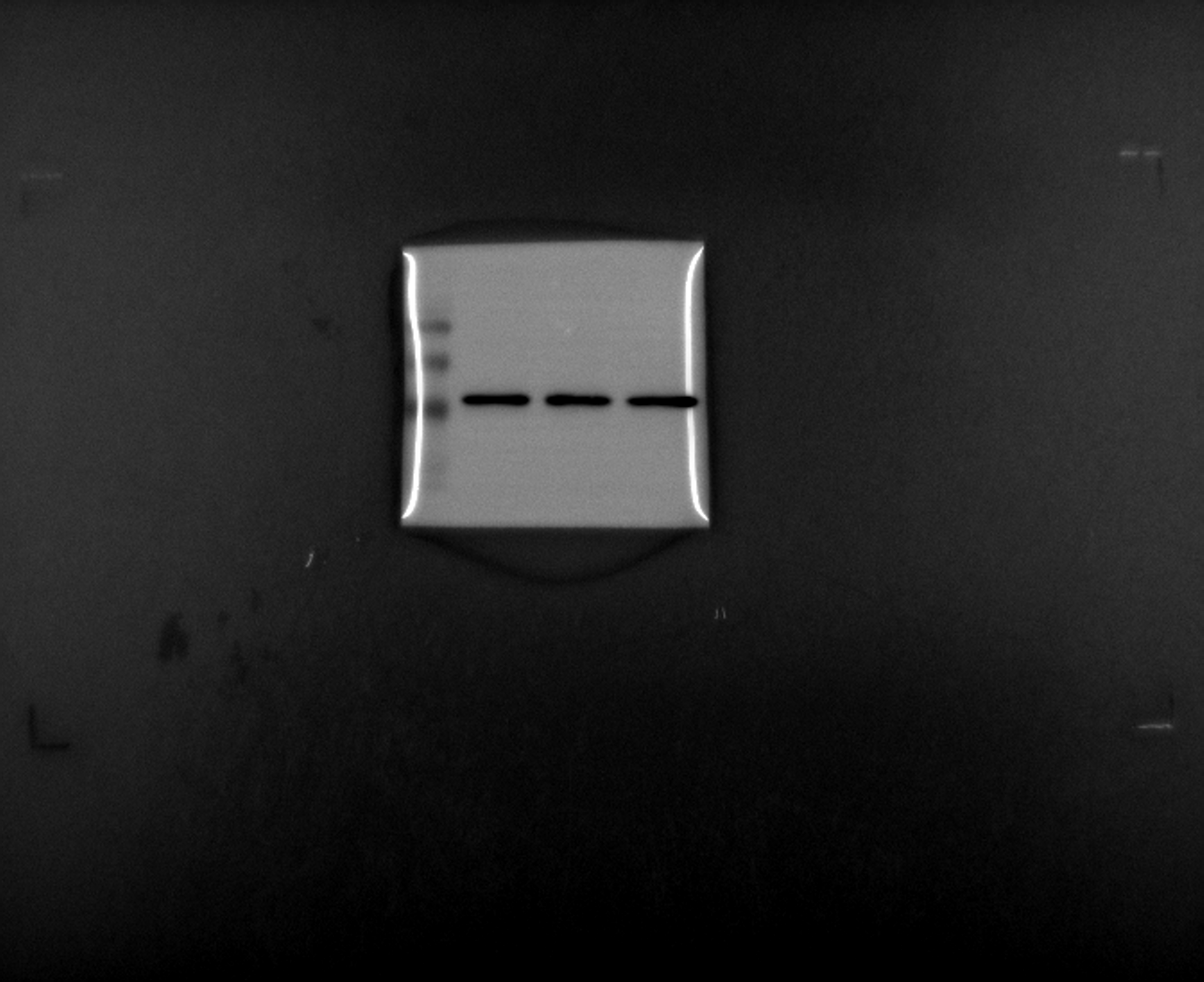

Supplement: Supplemental Information 4 [file peerj-12-17874-s004.zip › fig 2F/GAPDH-1 (3).tif]

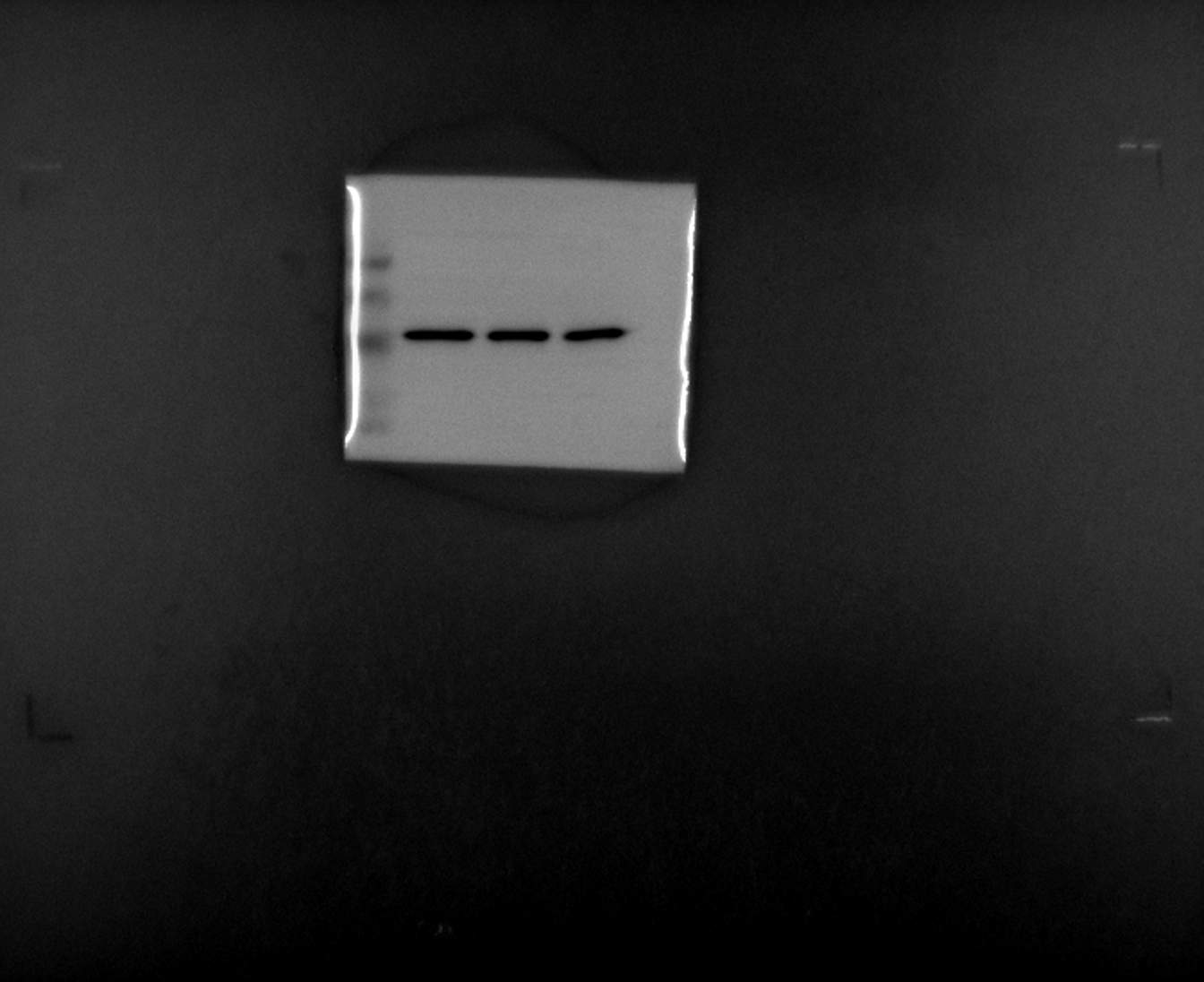

Supplement: Supplemental Information 4 [file peerj-12-17874-s004.zip › fig 2F/GAPDH-2 (1).tif]

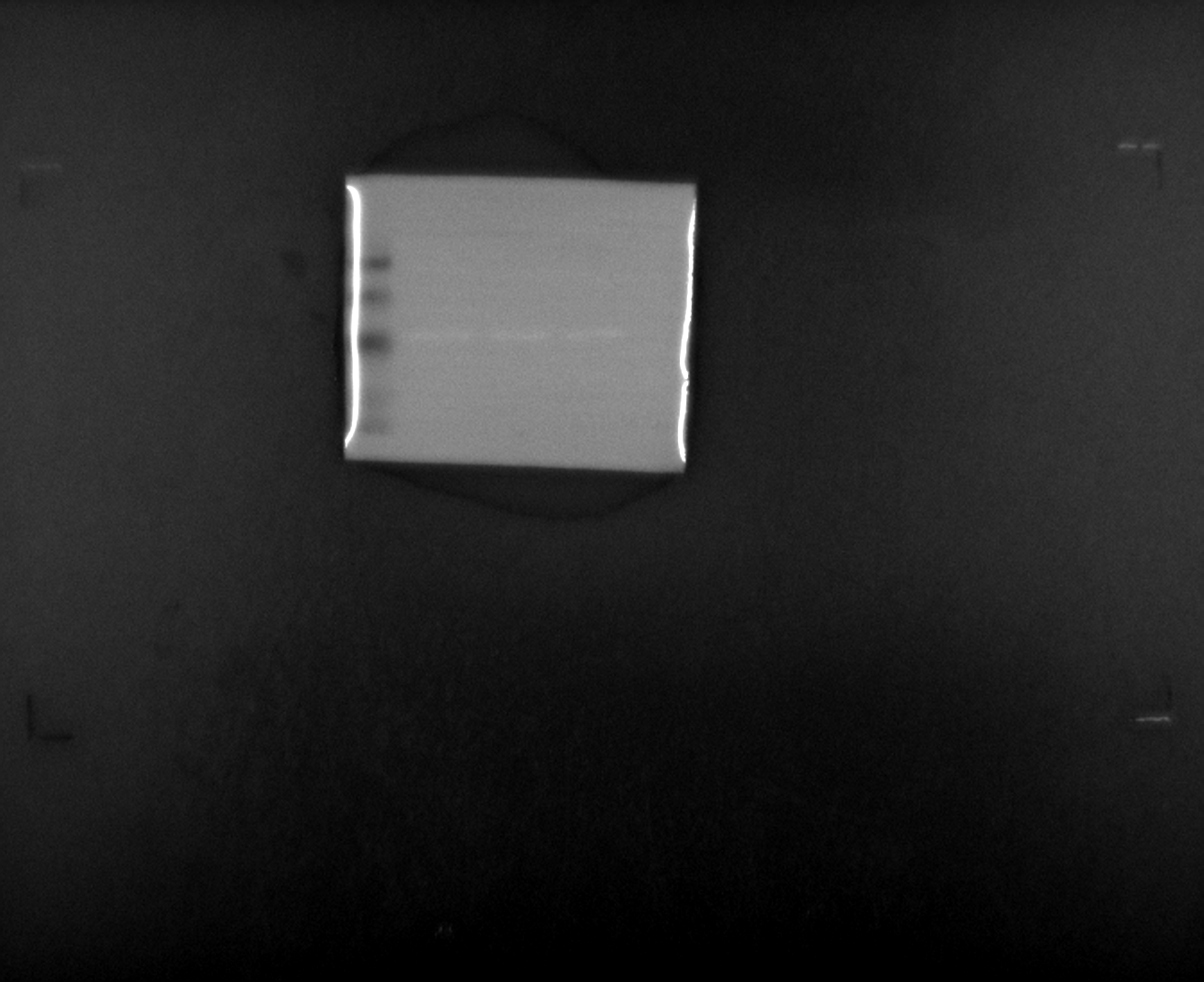

Supplement: Supplemental Information 4 [file peerj-12-17874-s004.zip › fig 2F/GAPDH-2 (2).tif]

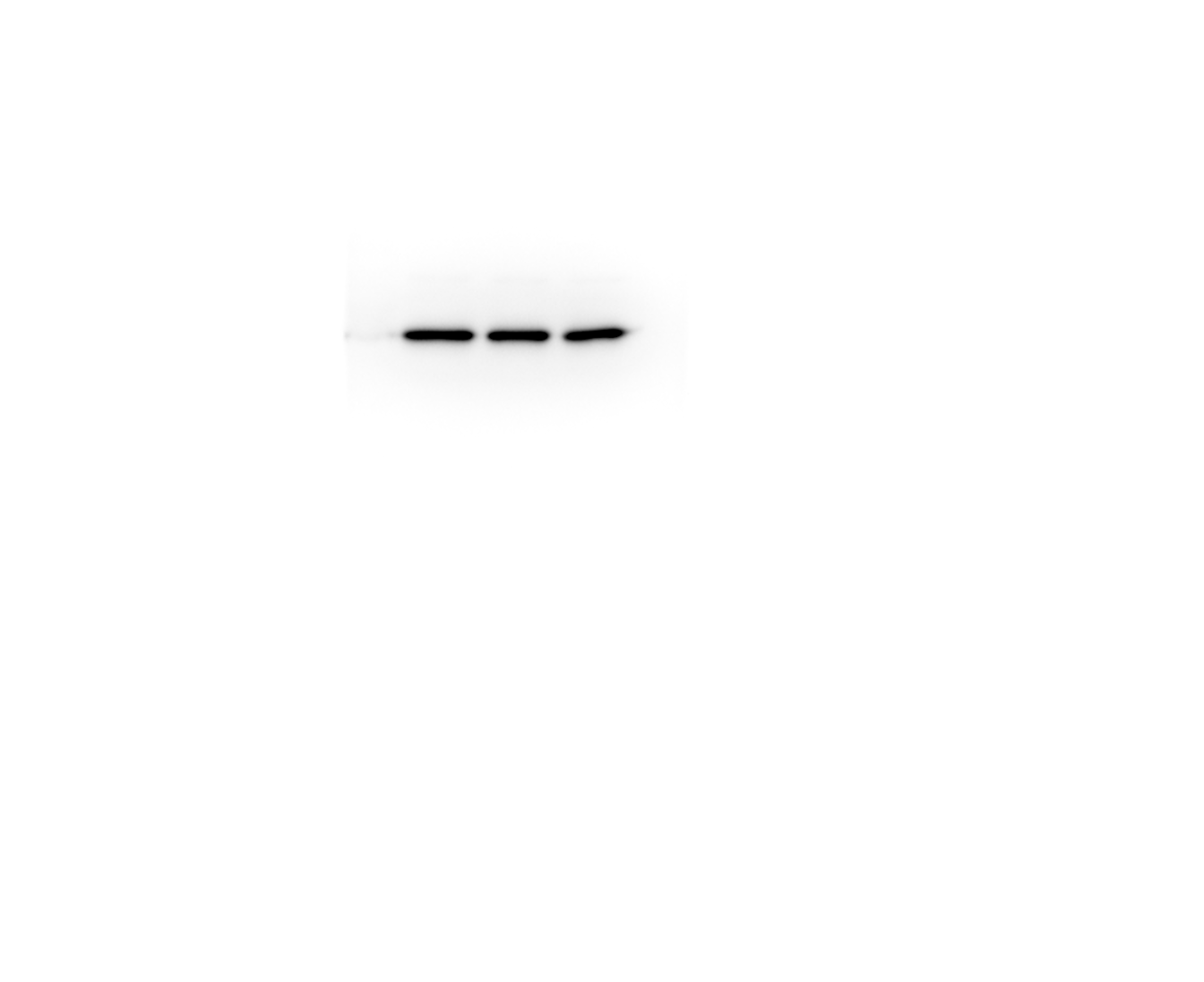

Supplement: Supplemental Information 4 [file peerj-12-17874-s004.zip › fig 2F/GAPDH-2 (3).tif]

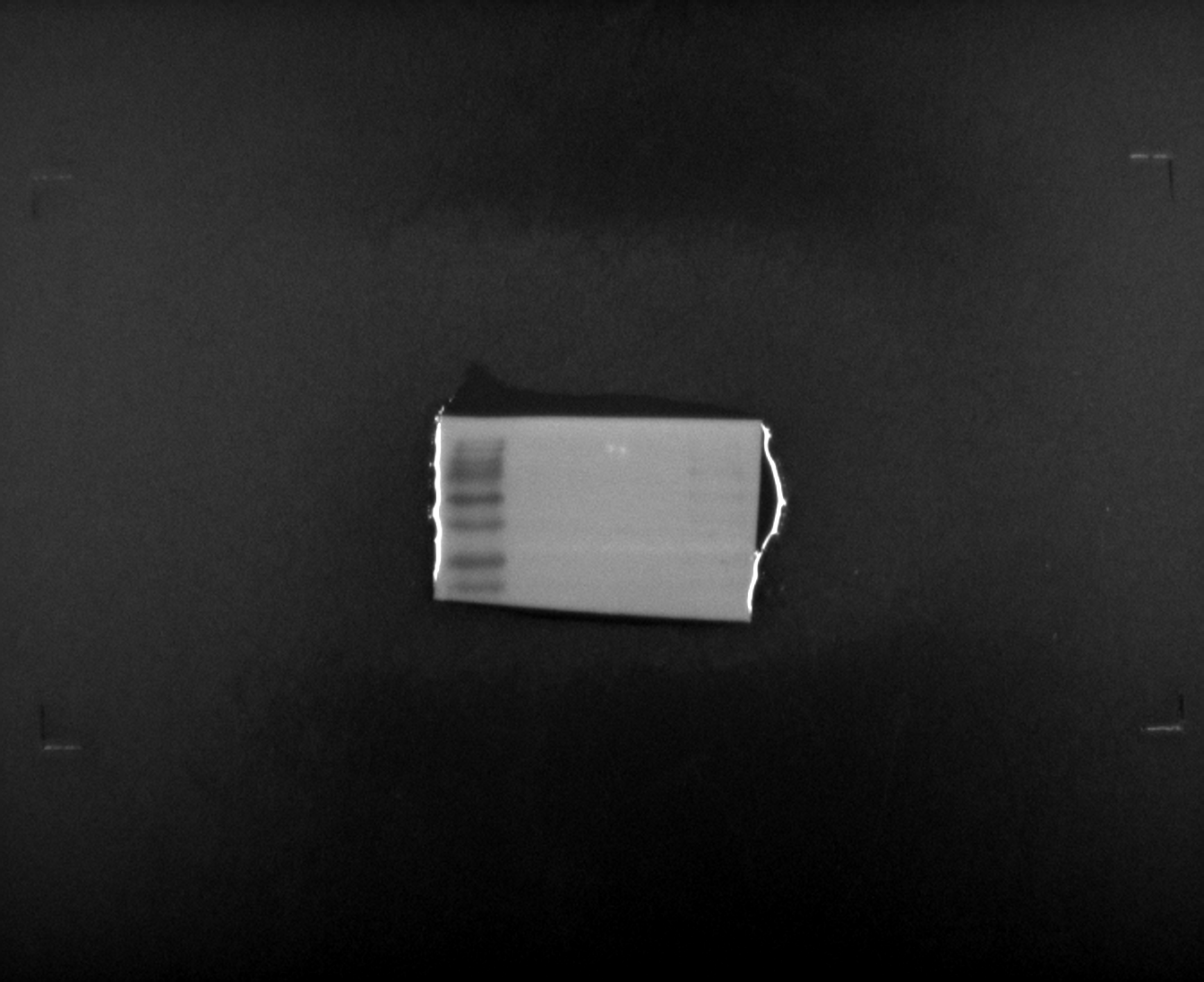

Supplement: Supplemental Information 4 [file peerj-12-17874-s004.zip › fig 2F/NRLP3 (1).tif]

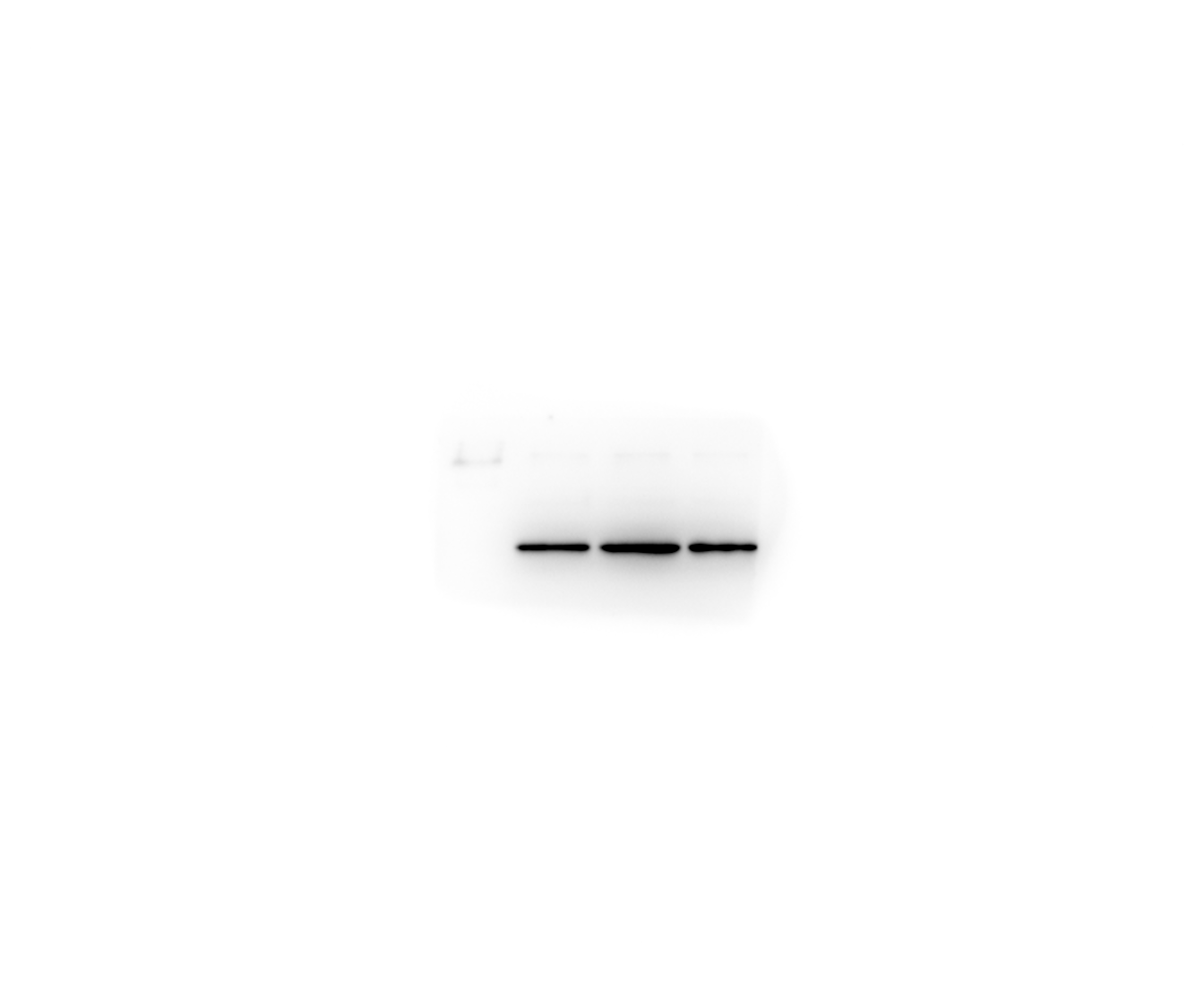

Supplement: Supplemental Information 4 [file peerj-12-17874-s004.zip › fig 2F/NRLP3 (2).tif]

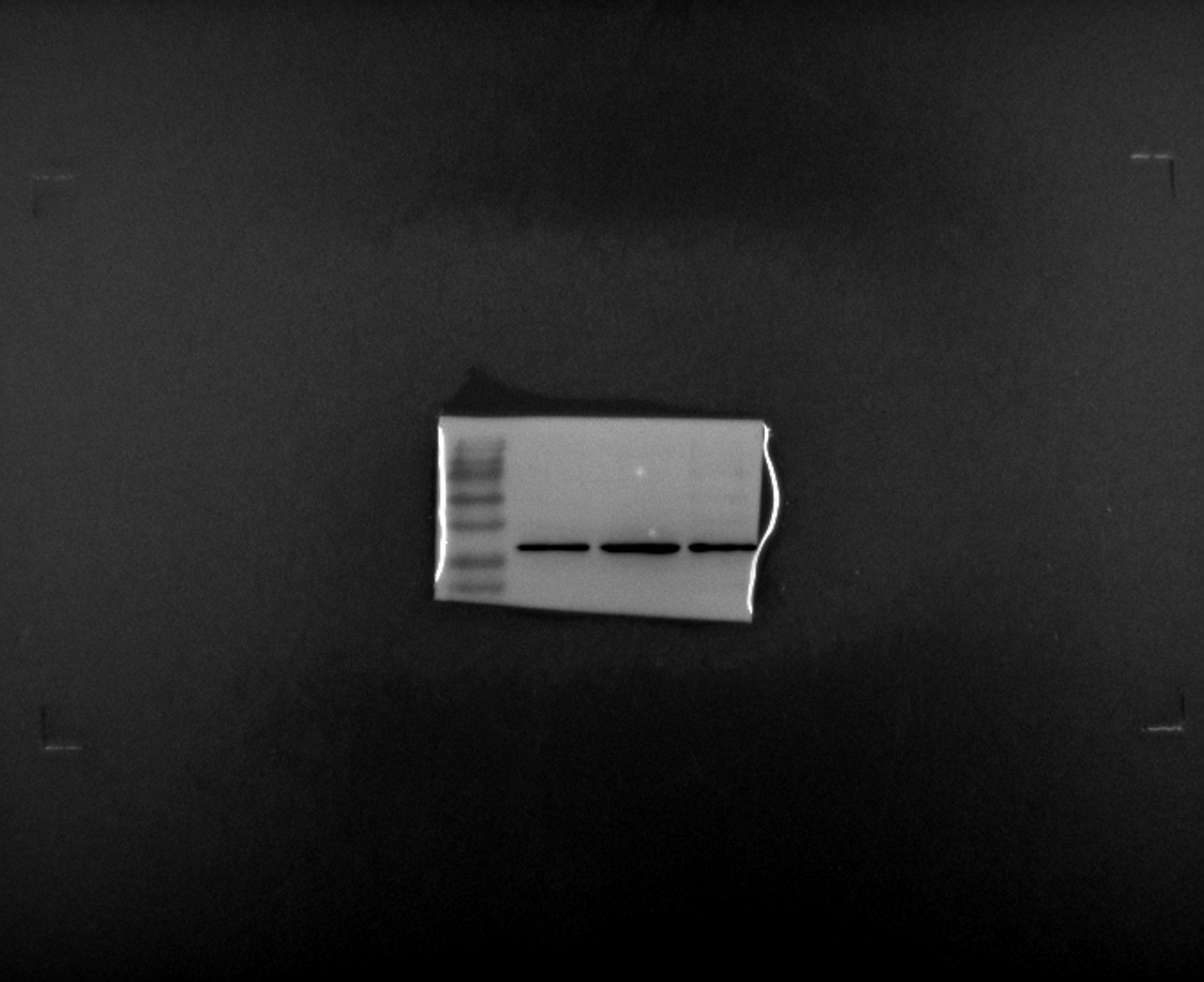

Supplement: Supplemental Information 4 [file peerj-12-17874-s004.zip › fig 2F/NRLP3 (3).tif]

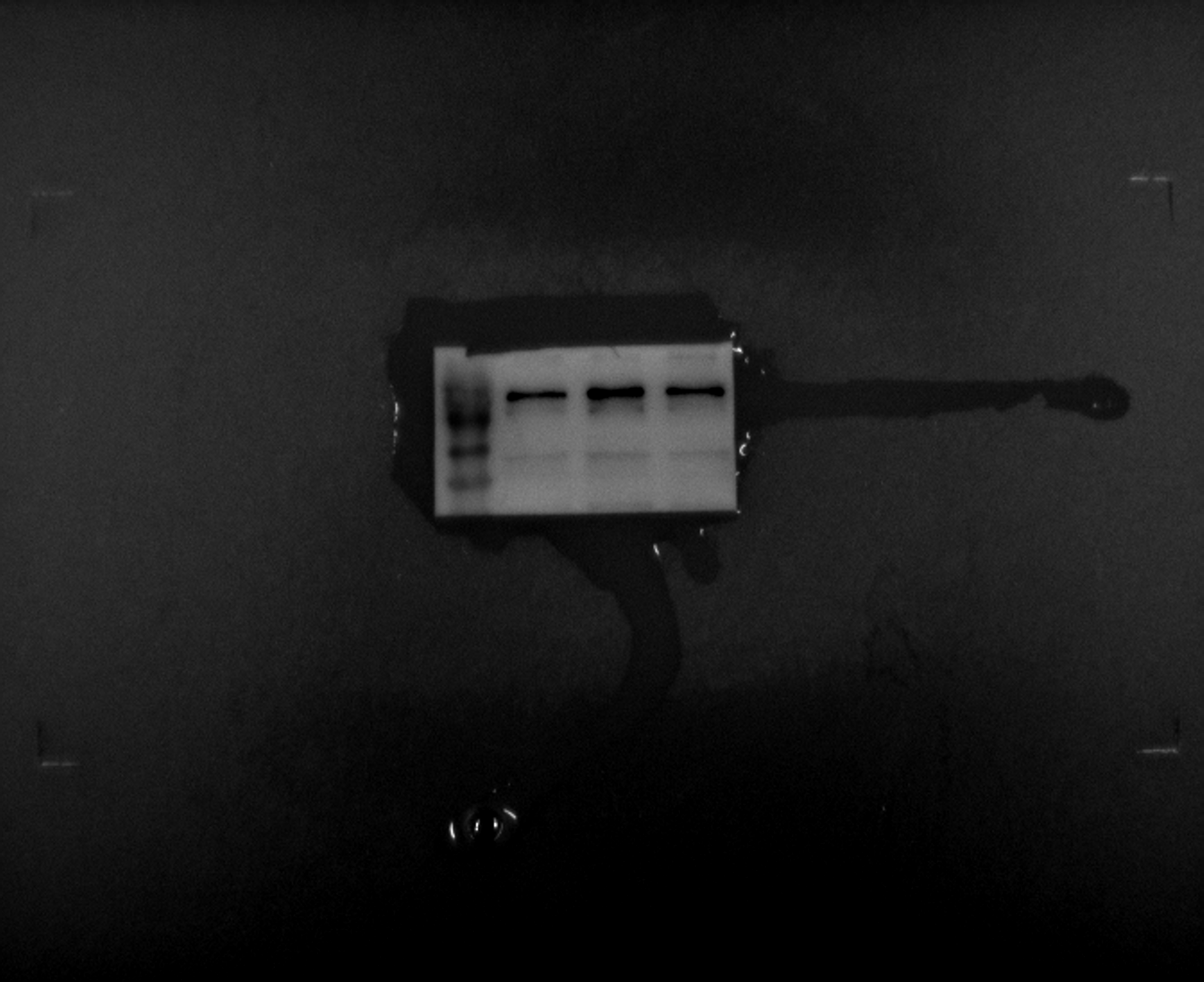

Supplement: Supplemental Information 4 [file peerj-12-17874-s004.zip › fig 2F/NRLP3-2 (1).tif]

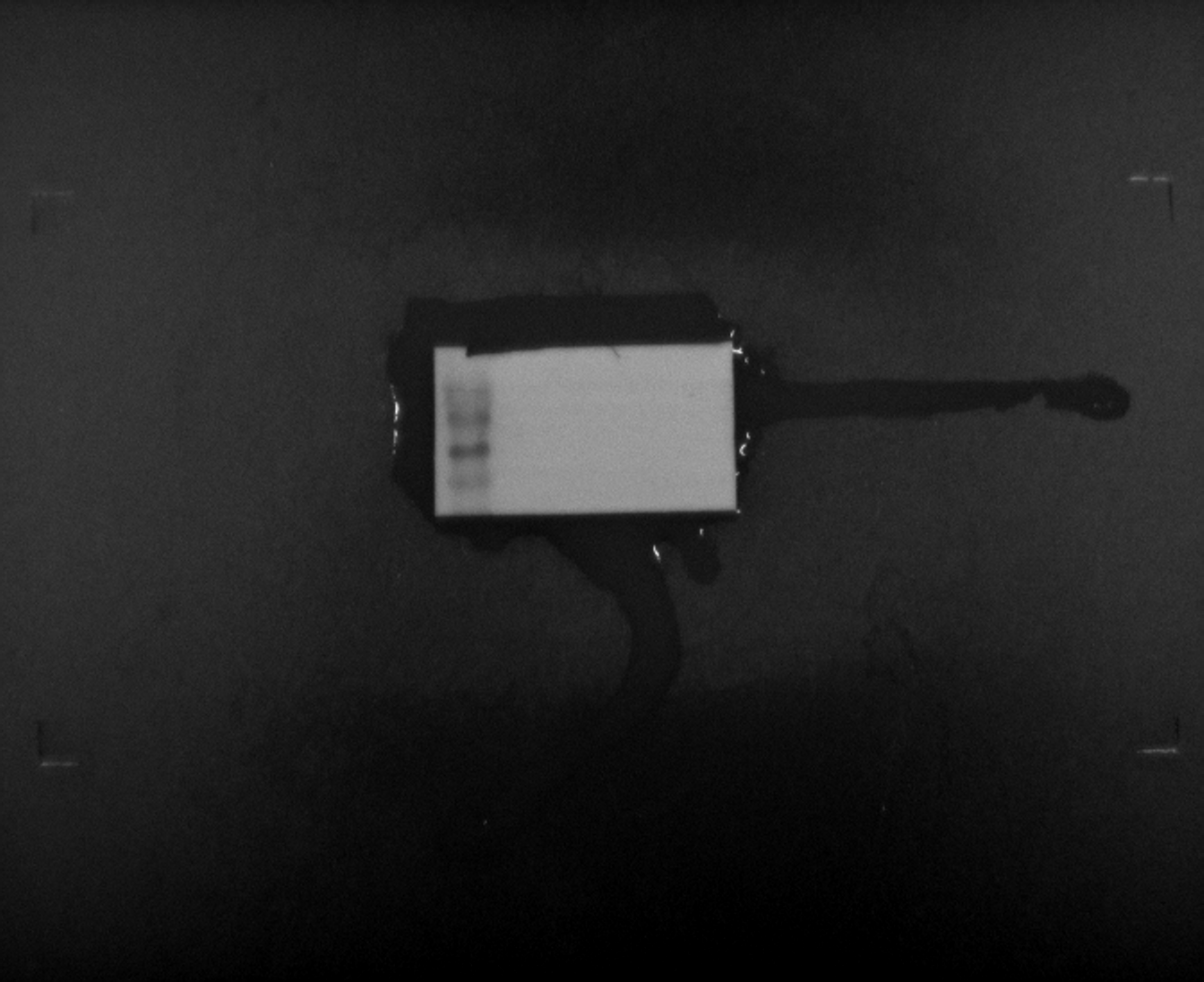

Supplement: Supplemental Information 4 [file peerj-12-17874-s004.zip › fig 2F/NRLP3-2 (2).tif]

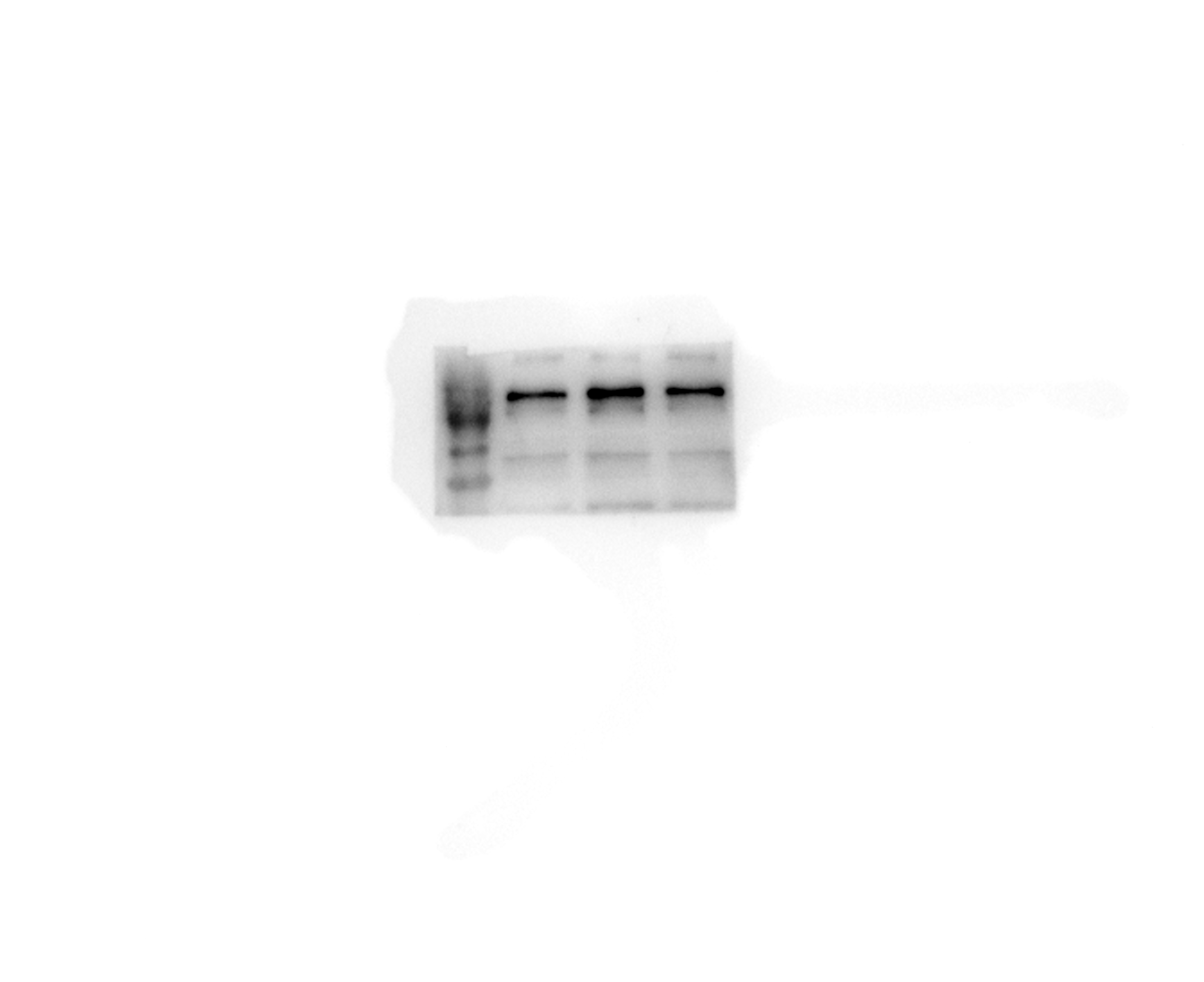

Supplement: Supplemental Information 4 [file peerj-12-17874-s004.zip › fig 2F/NRLP3-2 (3).tif]

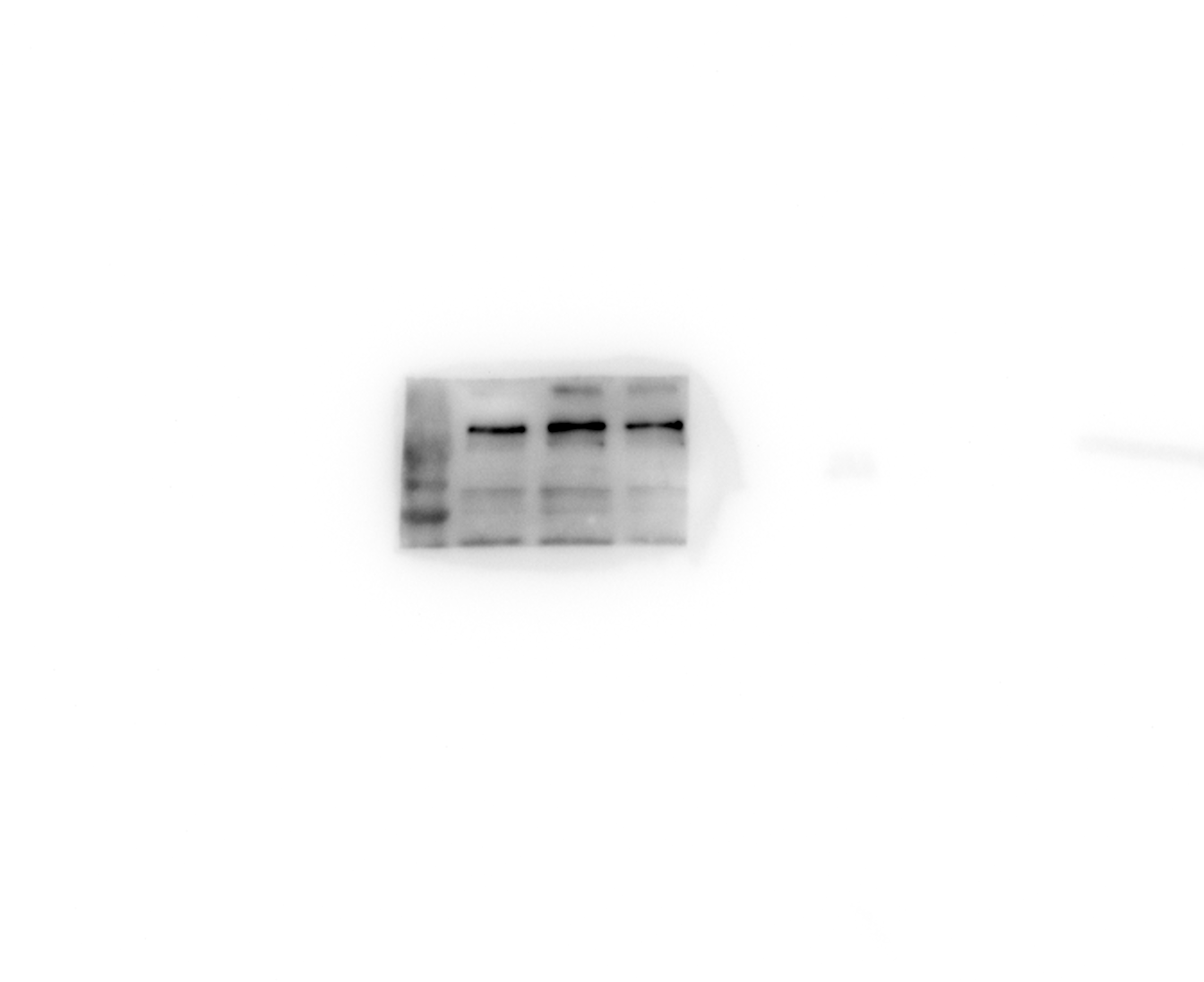

Supplement: Supplemental Information 4 [file peerj-12-17874-s004.zip › fig 2F/NRLP3-3 (1).tif]

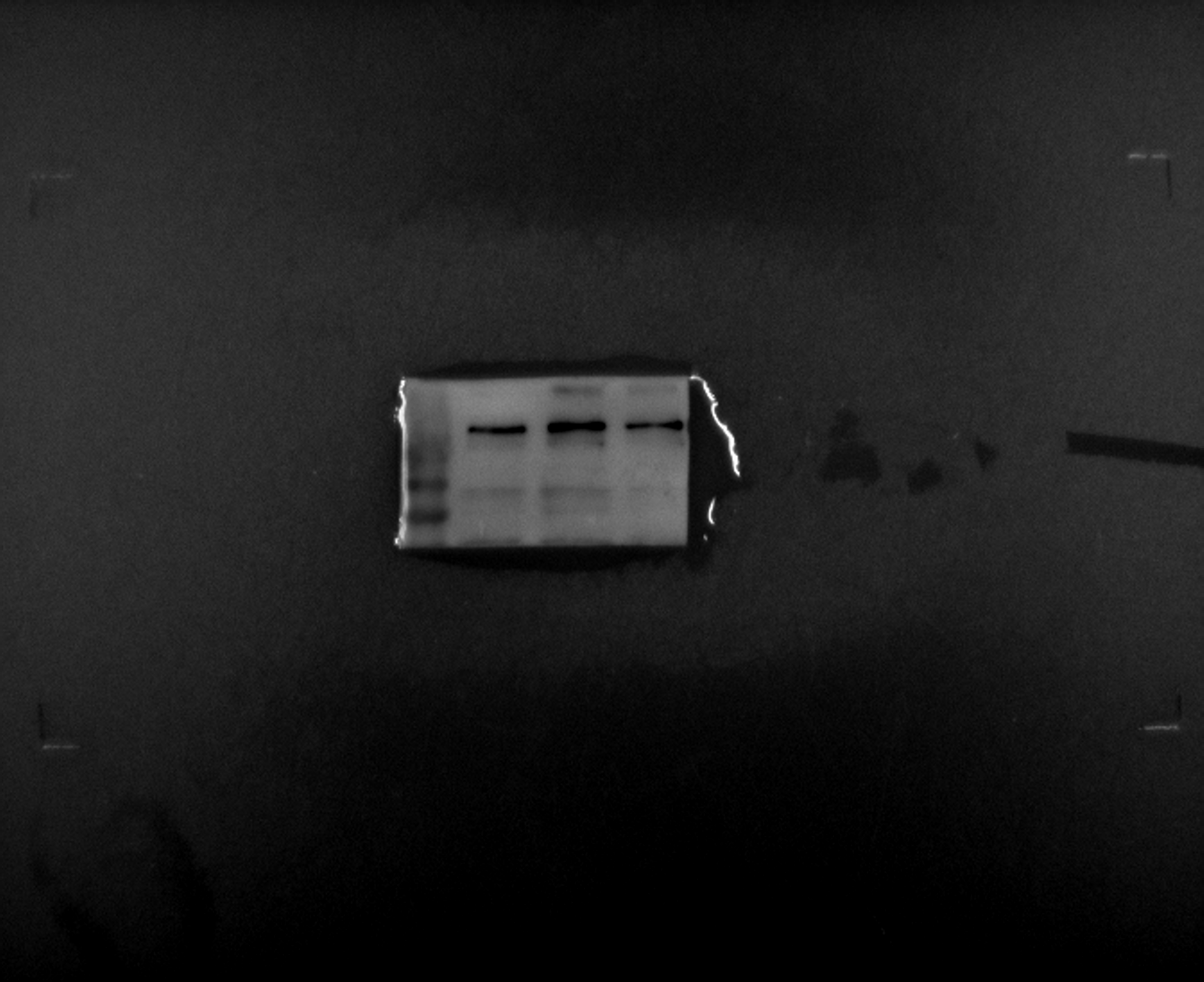

Supplement: Supplemental Information 4 [file peerj-12-17874-s004.zip › fig 2F/NRLP3-3 (2).tif]

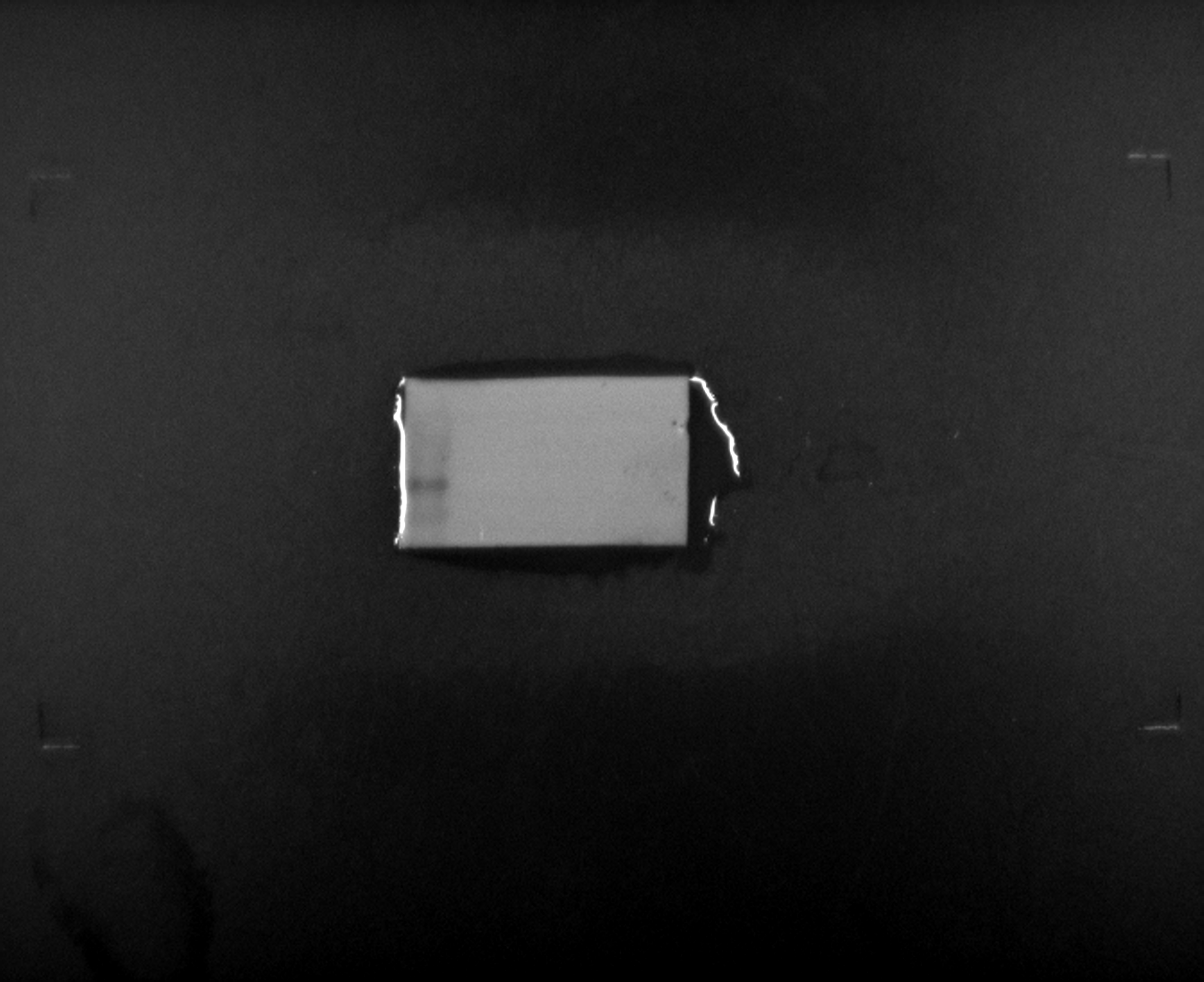

Supplement: Supplemental Information 4 [file peerj-12-17874-s004.zip › fig 2F/NRLP3-3 (3).tif]

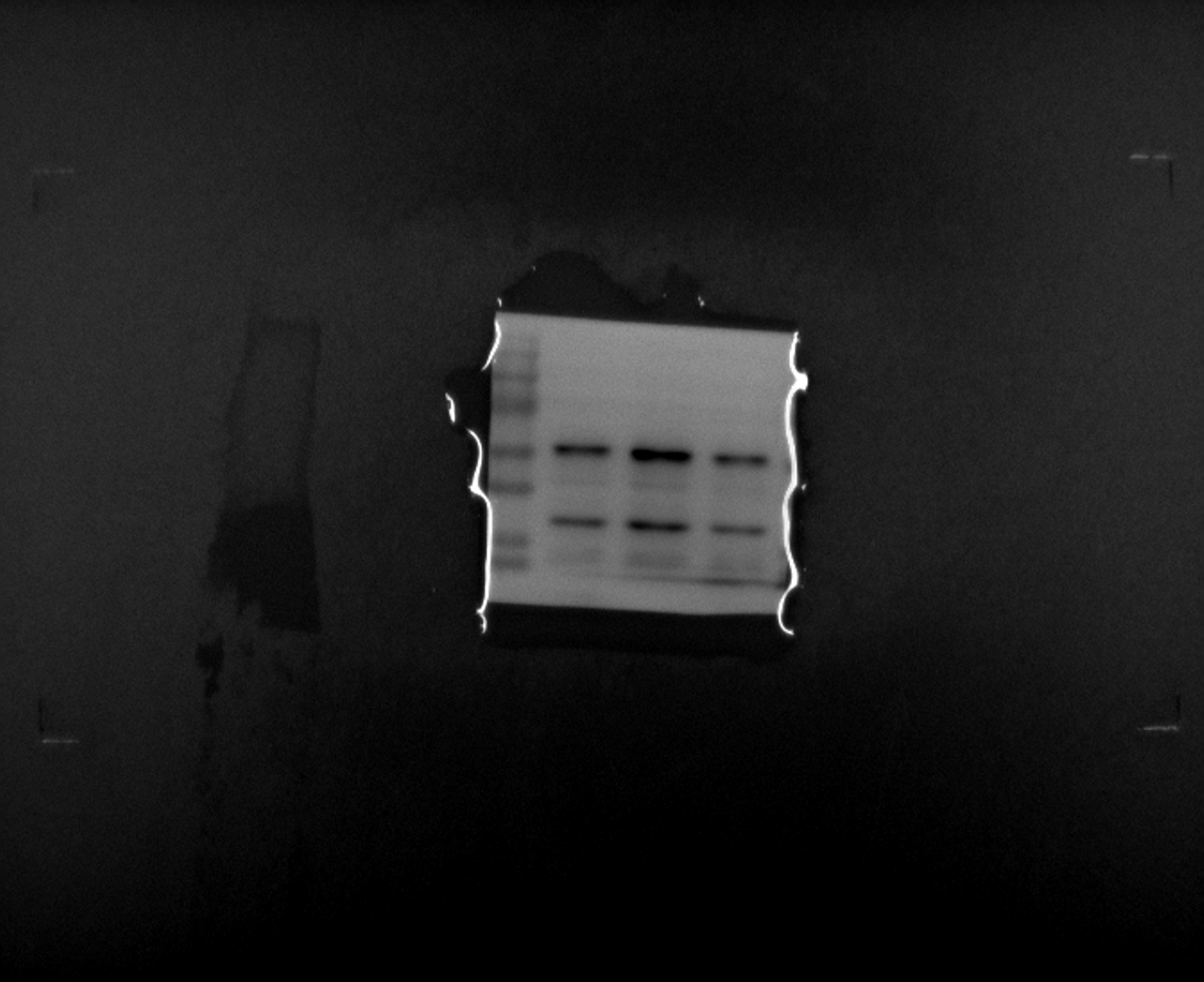

Supplement: Supplemental Information 4 [file peerj-12-17874-s004.zip › fig 2F/P-P65 (1).tif]

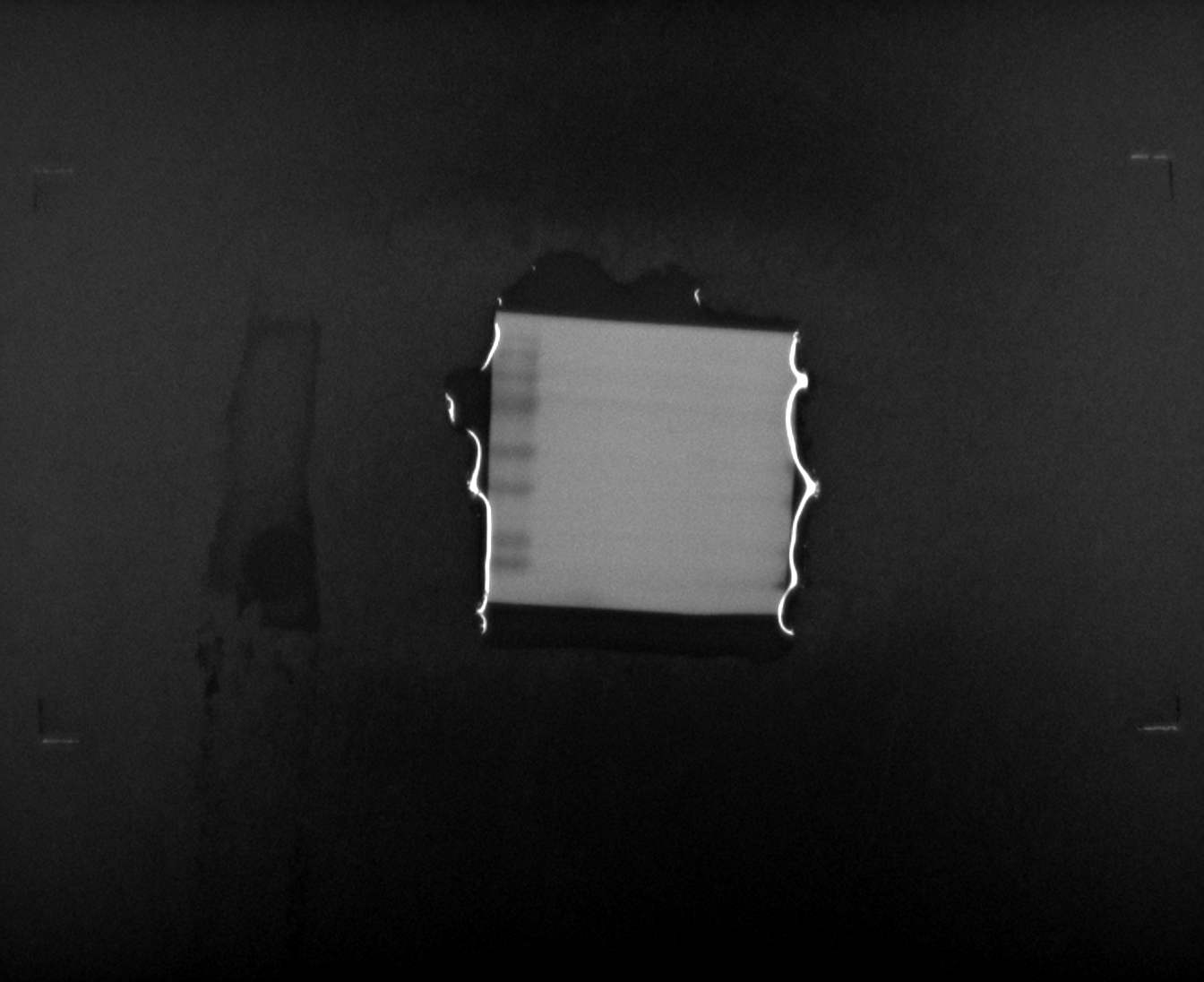

Supplement: Supplemental Information 4 [file peerj-12-17874-s004.zip › fig 2F/P-P65 (2).tif]

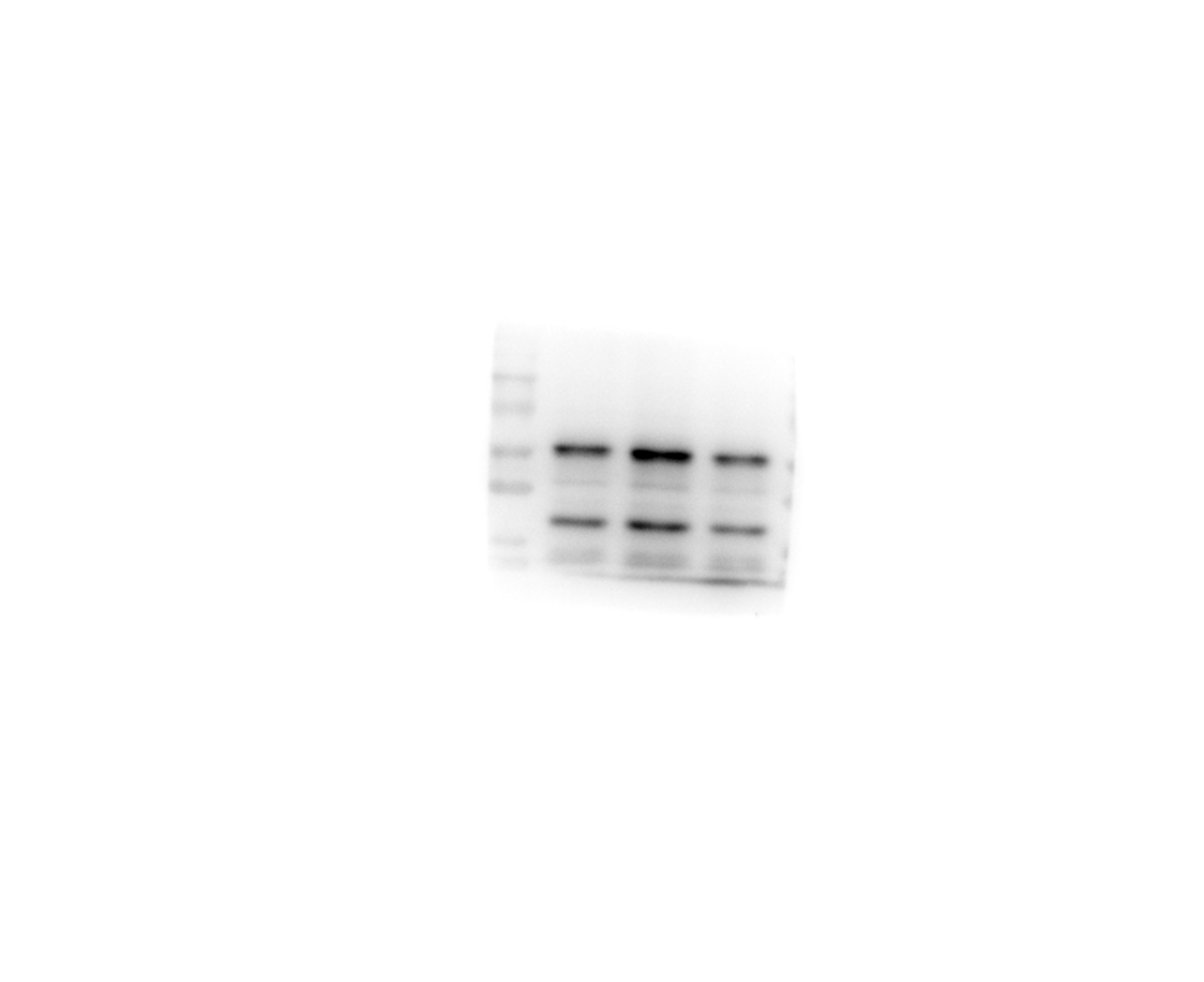

Supplement: Supplemental Information 4 [file peerj-12-17874-s004.zip › fig 2F/P-P65 (3).tif]

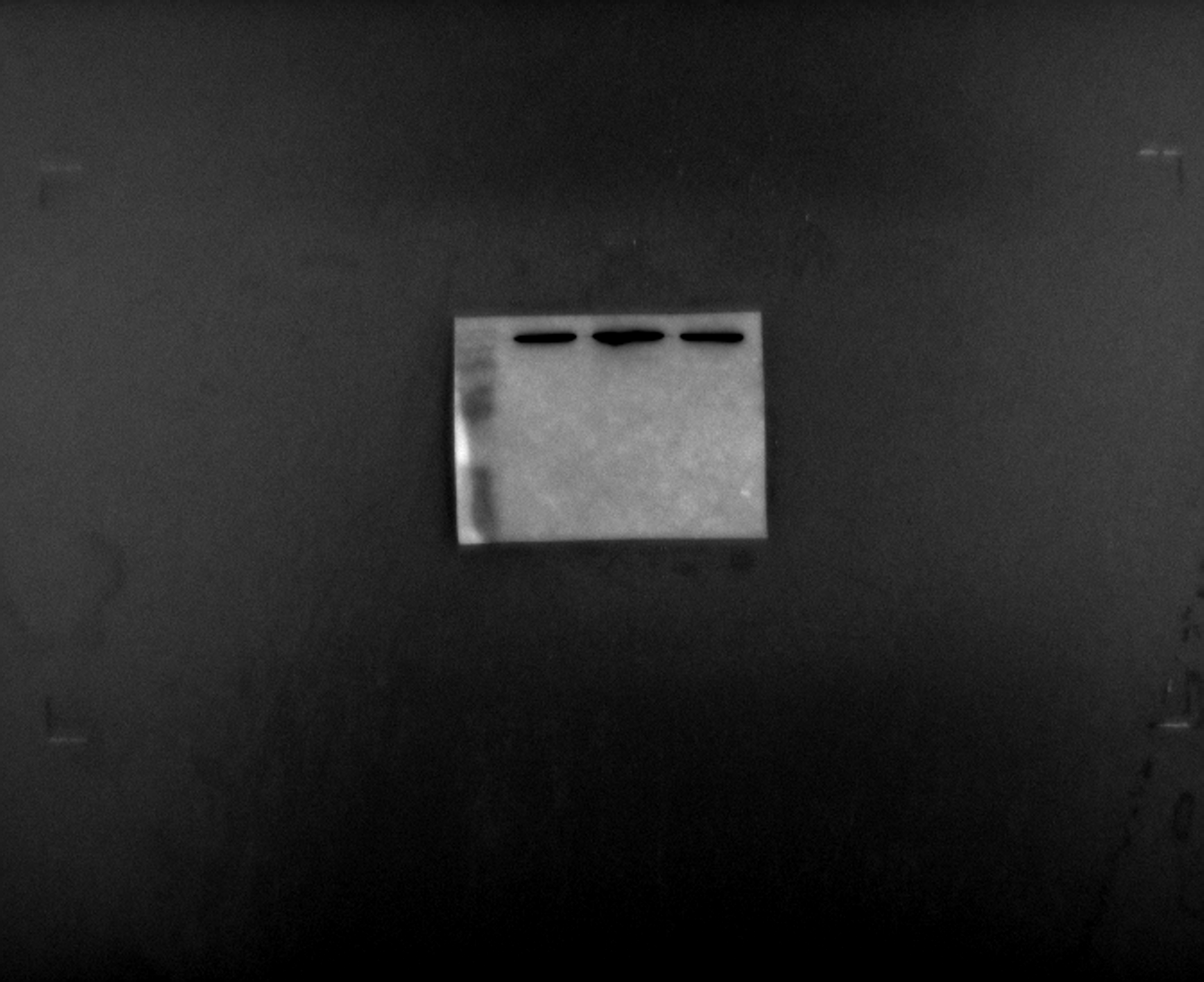

Supplement: Supplemental Information 4 [file peerj-12-17874-s004.zip › fig 2F/p-p65-2 (1).tif]

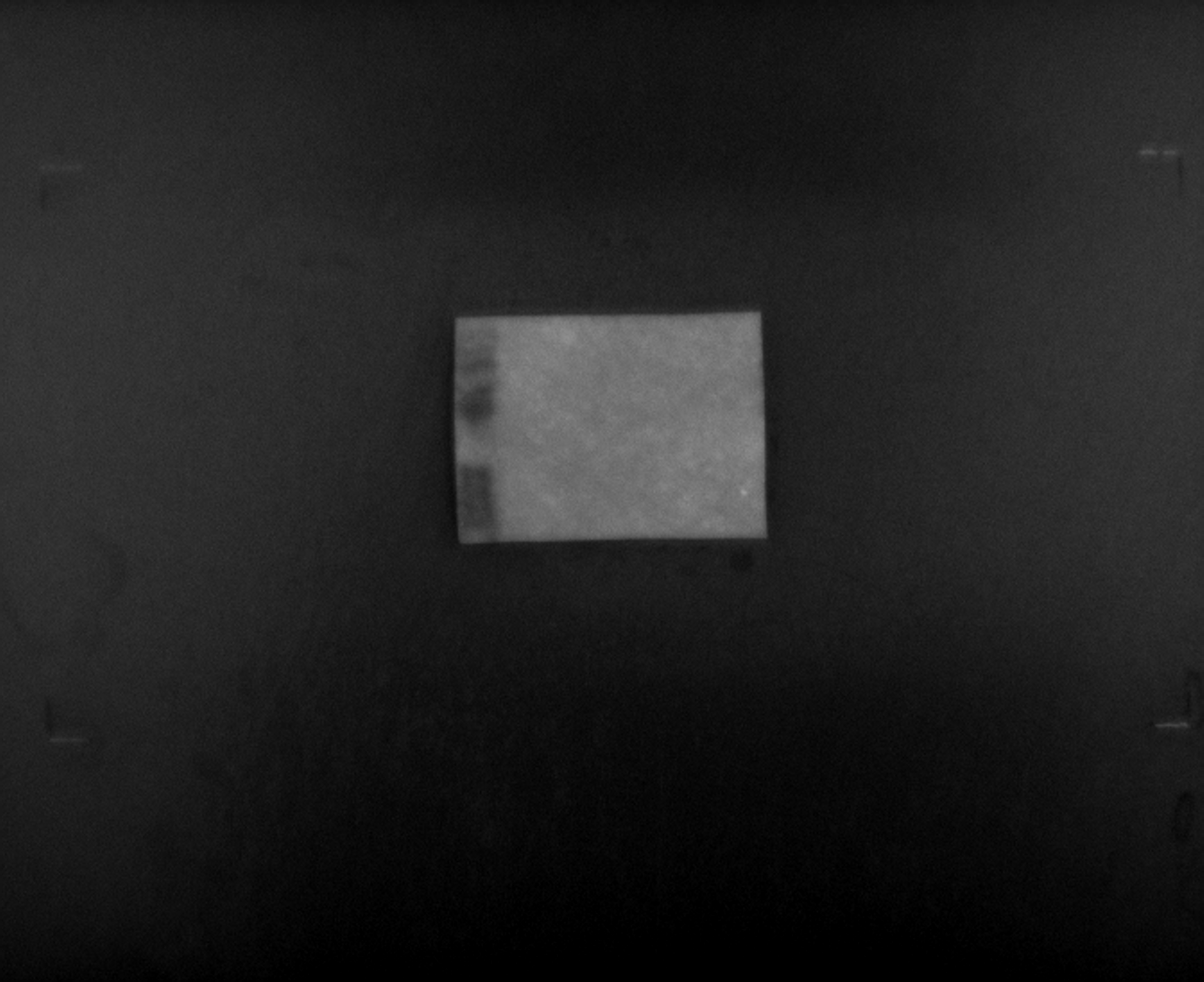

Supplement: Supplemental Information 4 [file peerj-12-17874-s004.zip › fig 2F/p-p65-2 (2).tif]

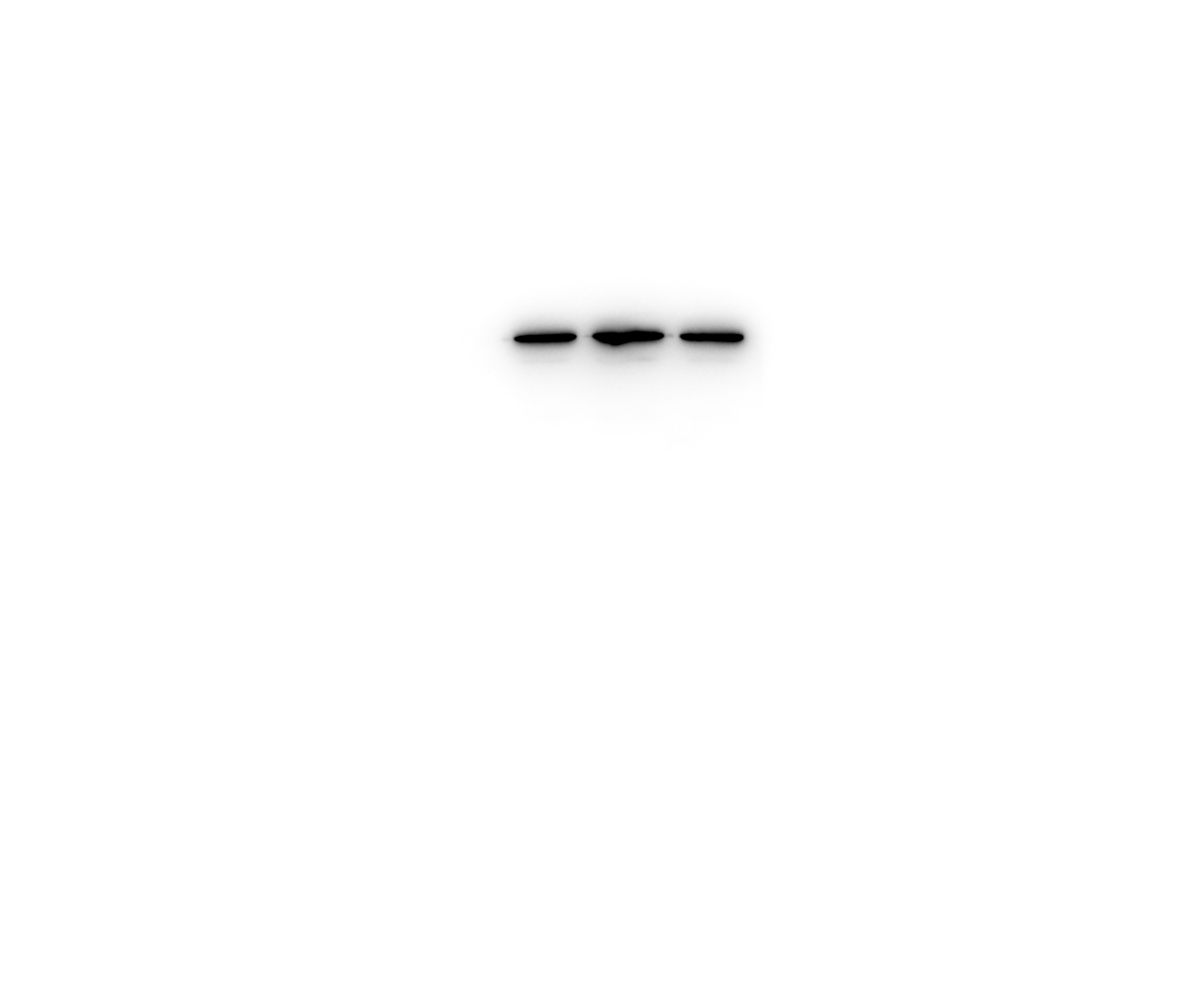

Supplement: Supplemental Information 4 [file peerj-12-17874-s004.zip › fig 2F/p-p65-2 (3).tif]

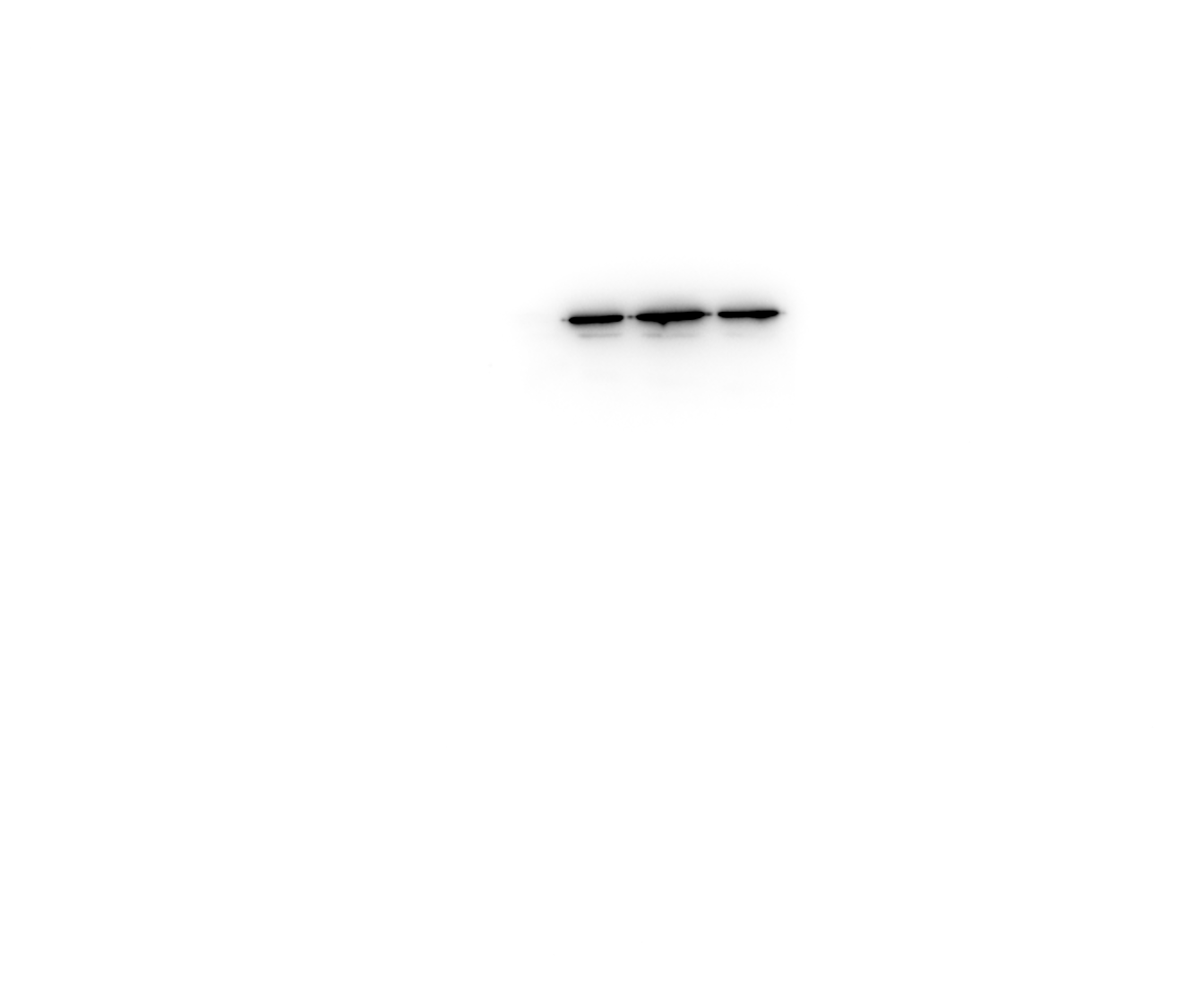

Supplement: Supplemental Information 4 [file peerj-12-17874-s004.zip › fig 2F/p-p65-3 (1).tif]

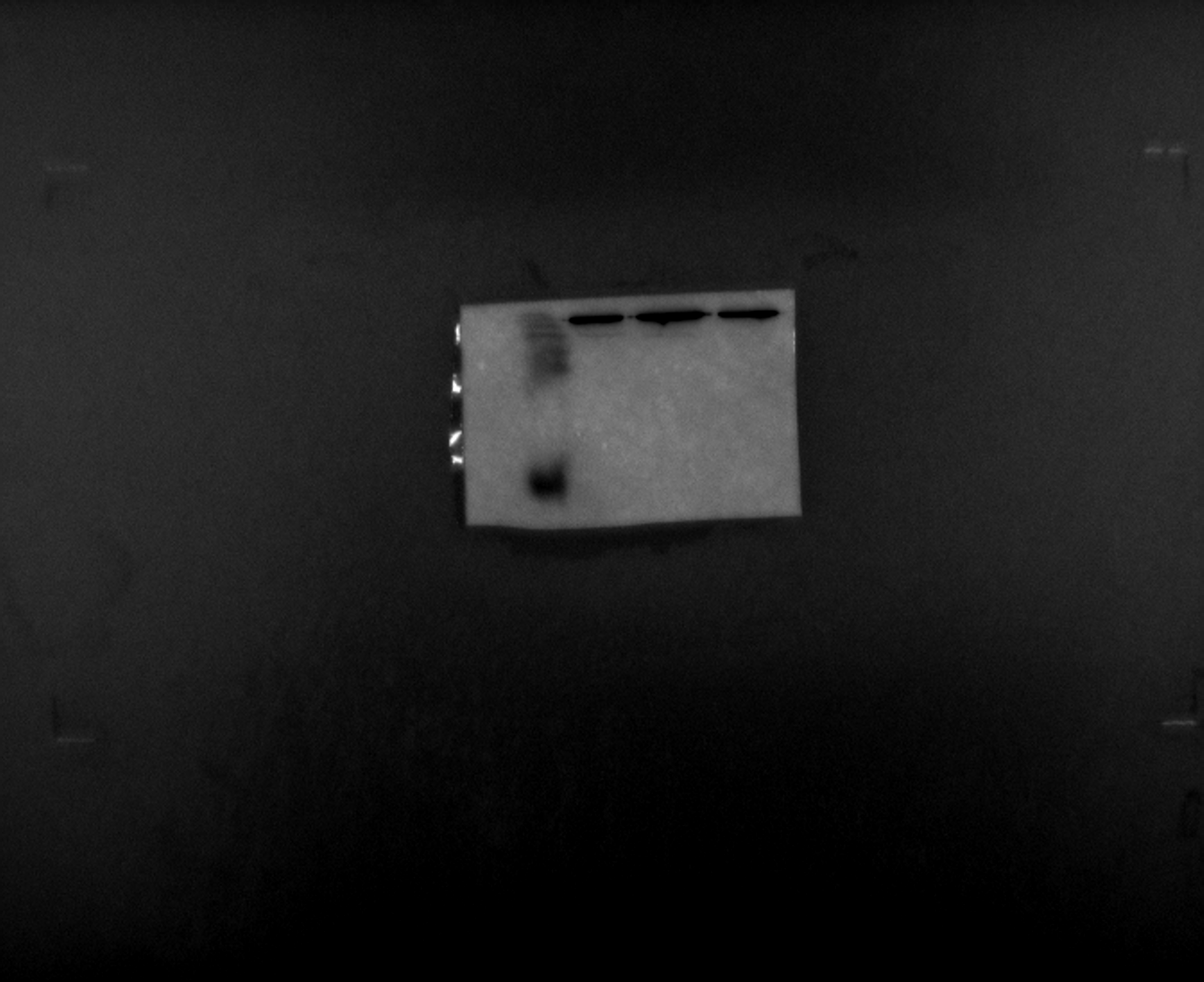

Supplement: Supplemental Information 4 [file peerj-12-17874-s004.zip › fig 2F/p-p65-3 (2).tif]

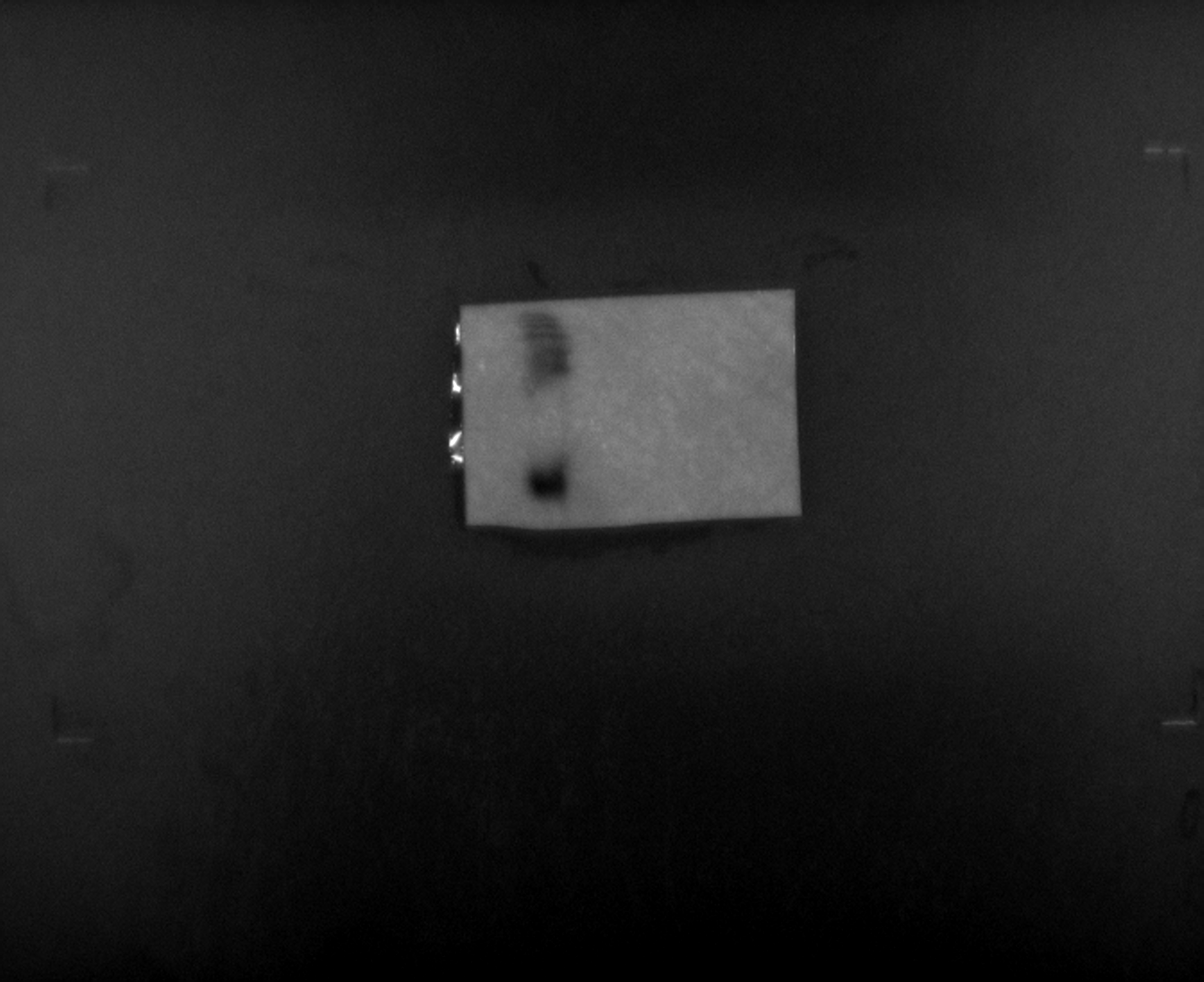

Supplement: Supplemental Information 4 [file peerj-12-17874-s004.zip › fig 2F/p-p65-3 (3).tif]

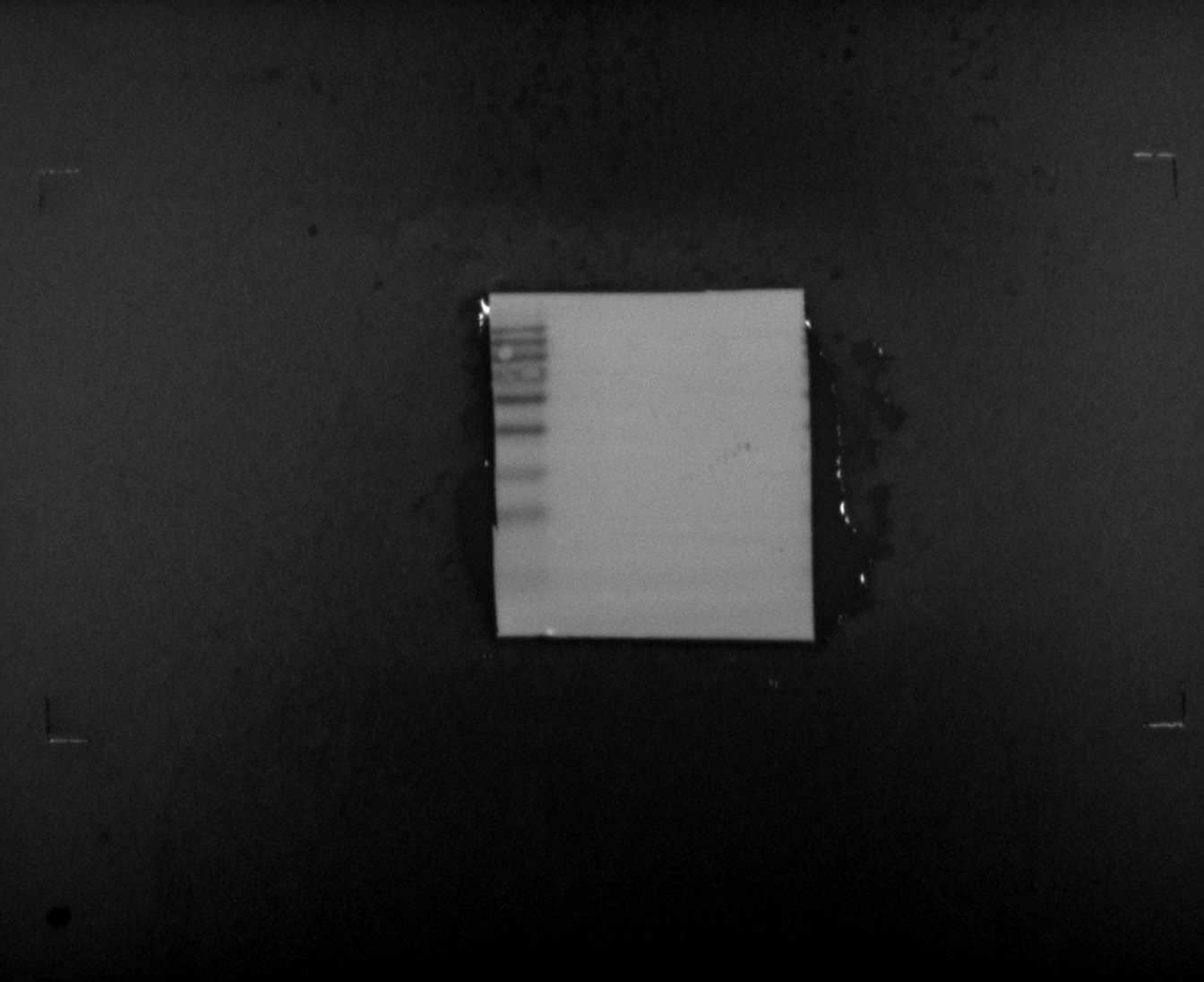

Supplement: Supplemental Information 5 [file peerj-12-17874-s005.zip › fig 3G/duox2 (1).tif]

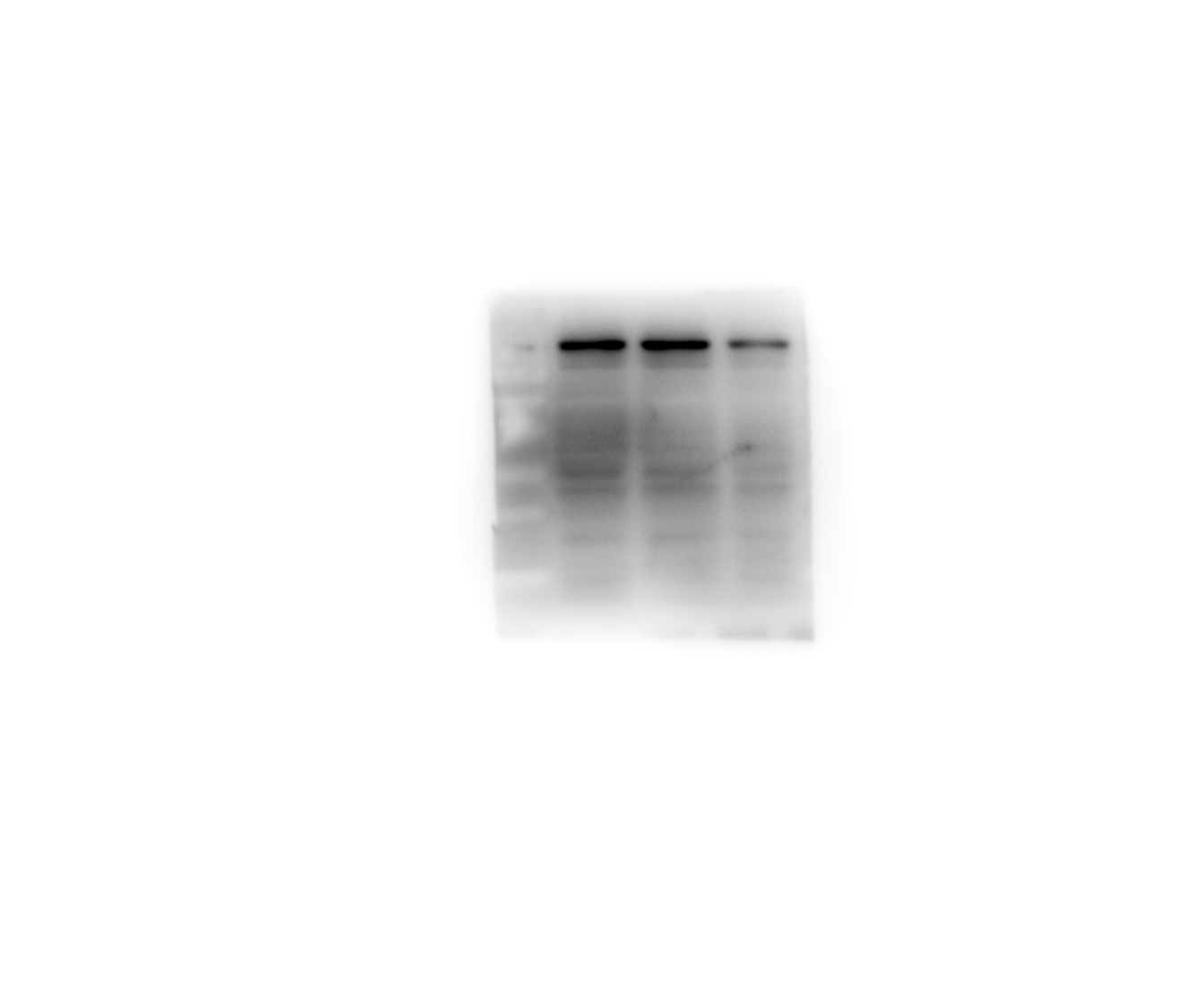

Supplement: Supplemental Information 5 [file peerj-12-17874-s005.zip › fig 3G/duox2 (2).tif]

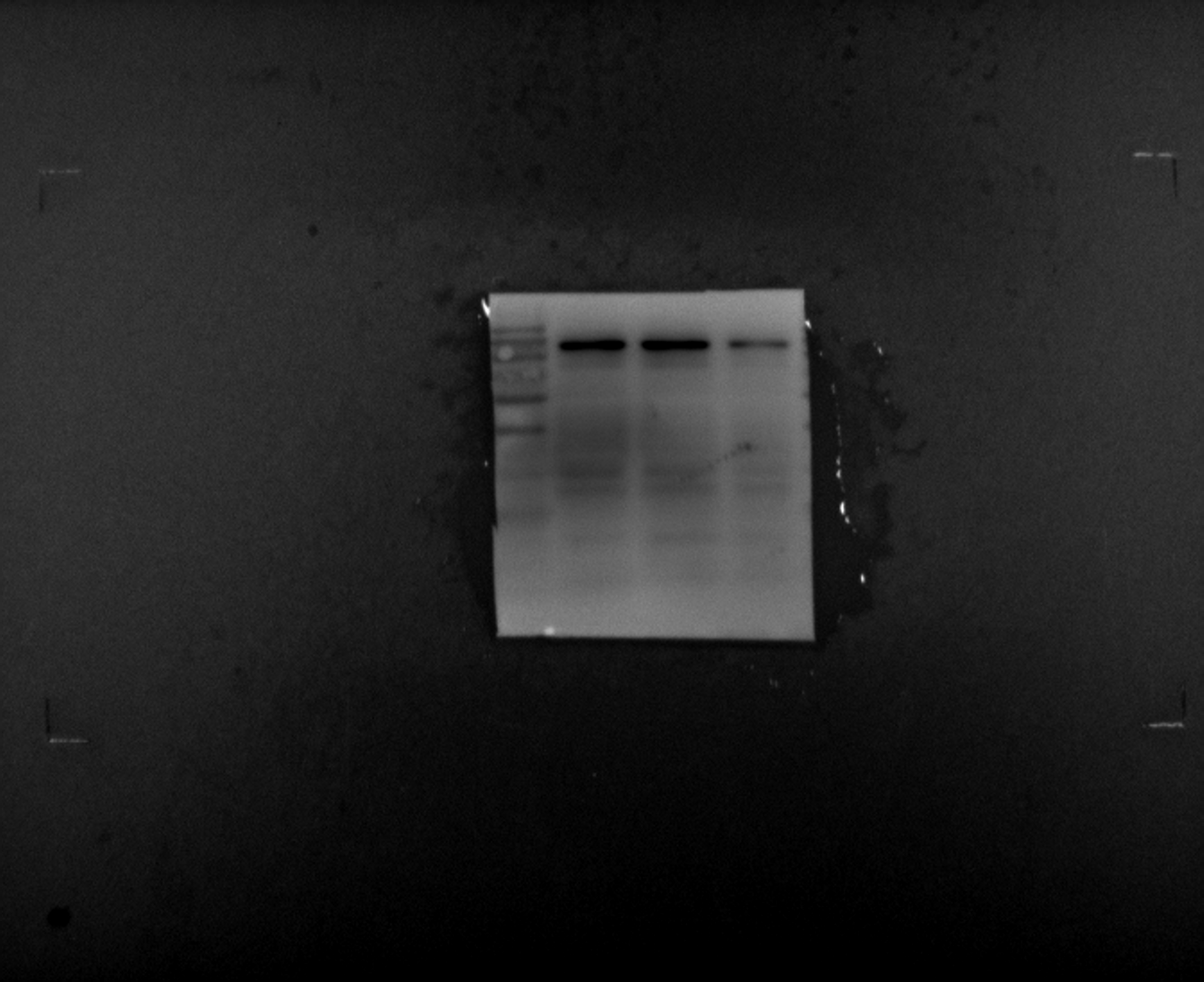

Supplement: Supplemental Information 5 [file peerj-12-17874-s005.zip › fig 3G/duox2 (3).tif]

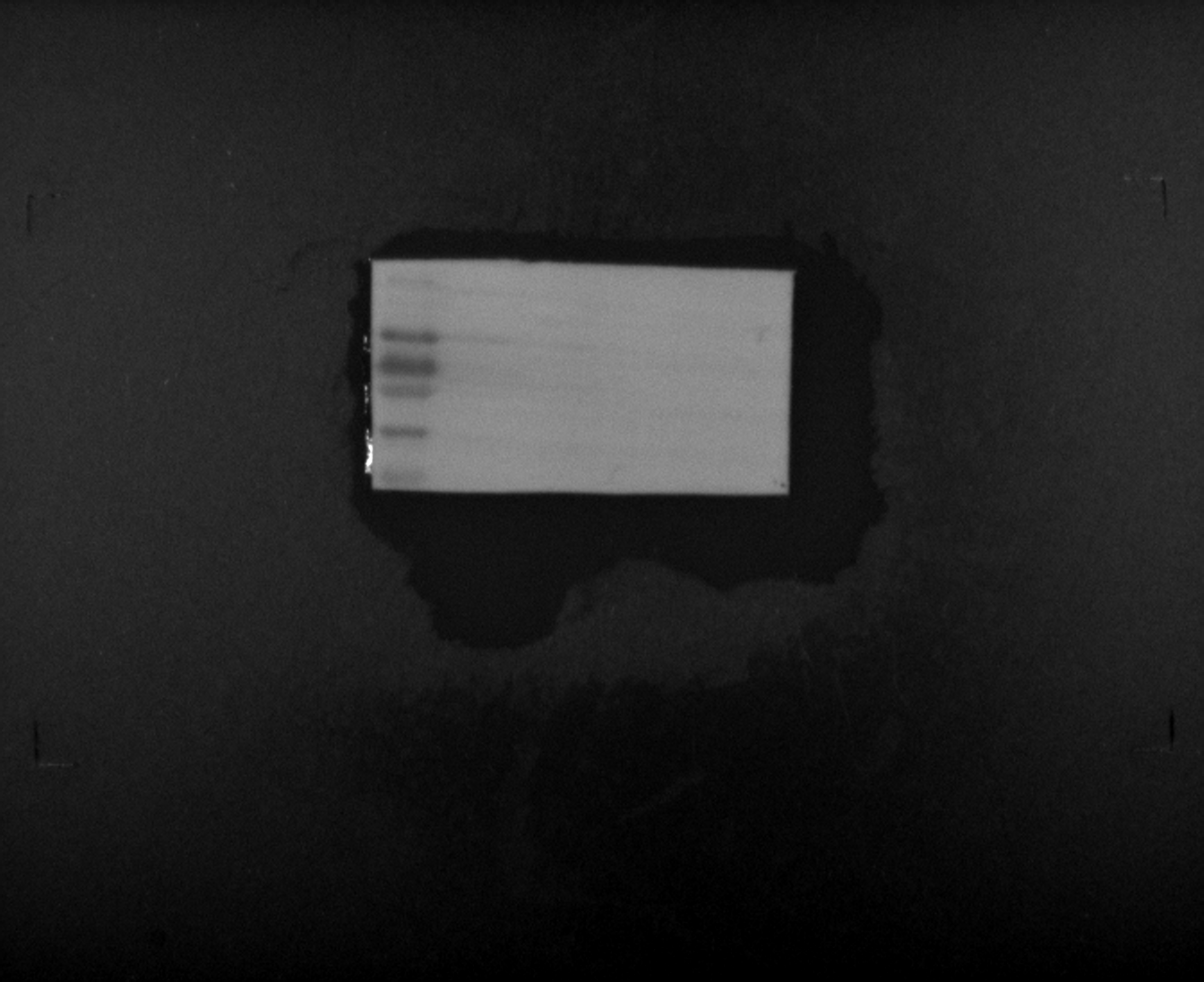

Supplement: Supplemental Information 5 [file peerj-12-17874-s005.zip › fig 3G/DUOX2-2 (1).tif]

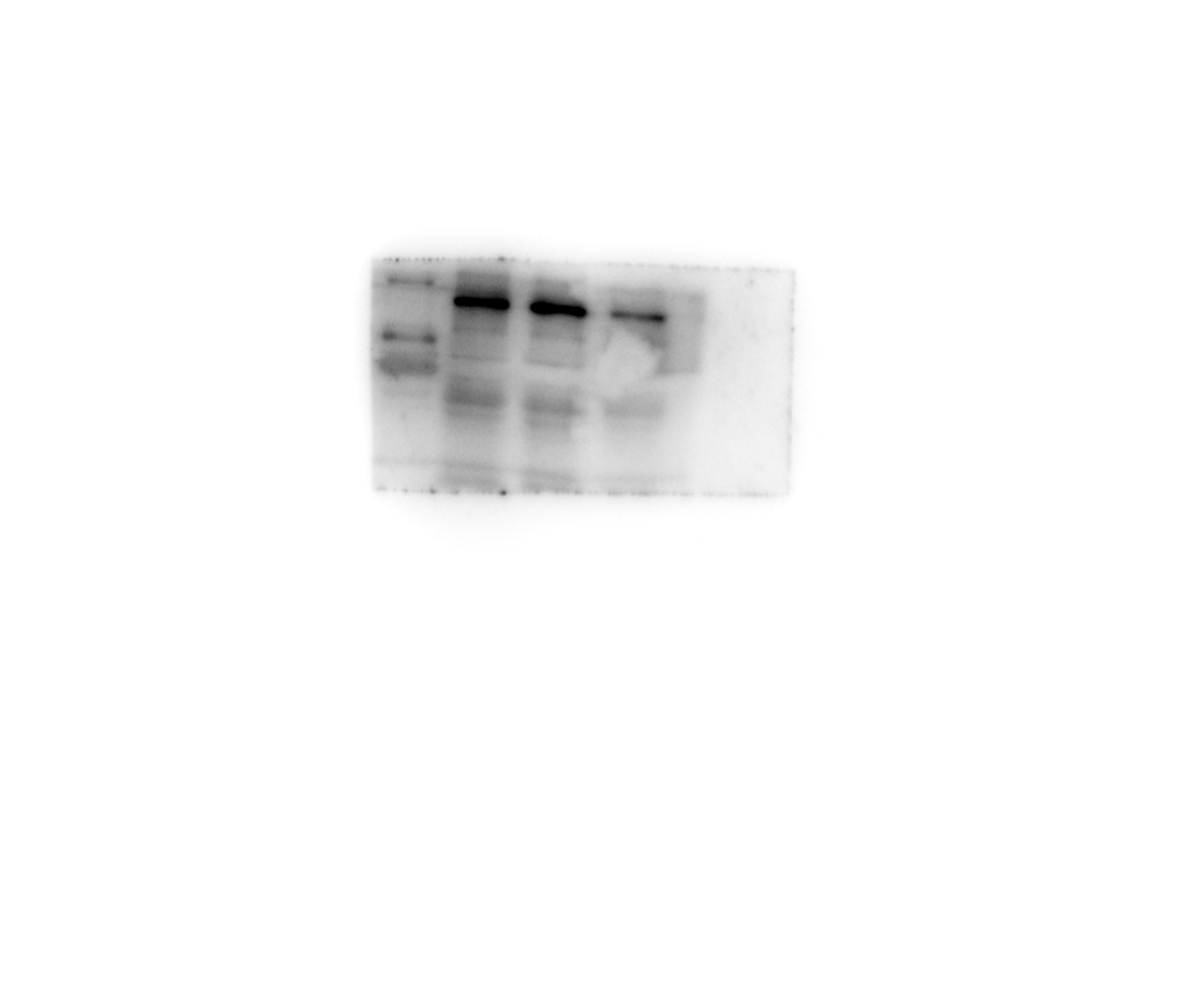

Supplement: Supplemental Information 5 [file peerj-12-17874-s005.zip › fig 3G/DUOX2-2 (2).tif]

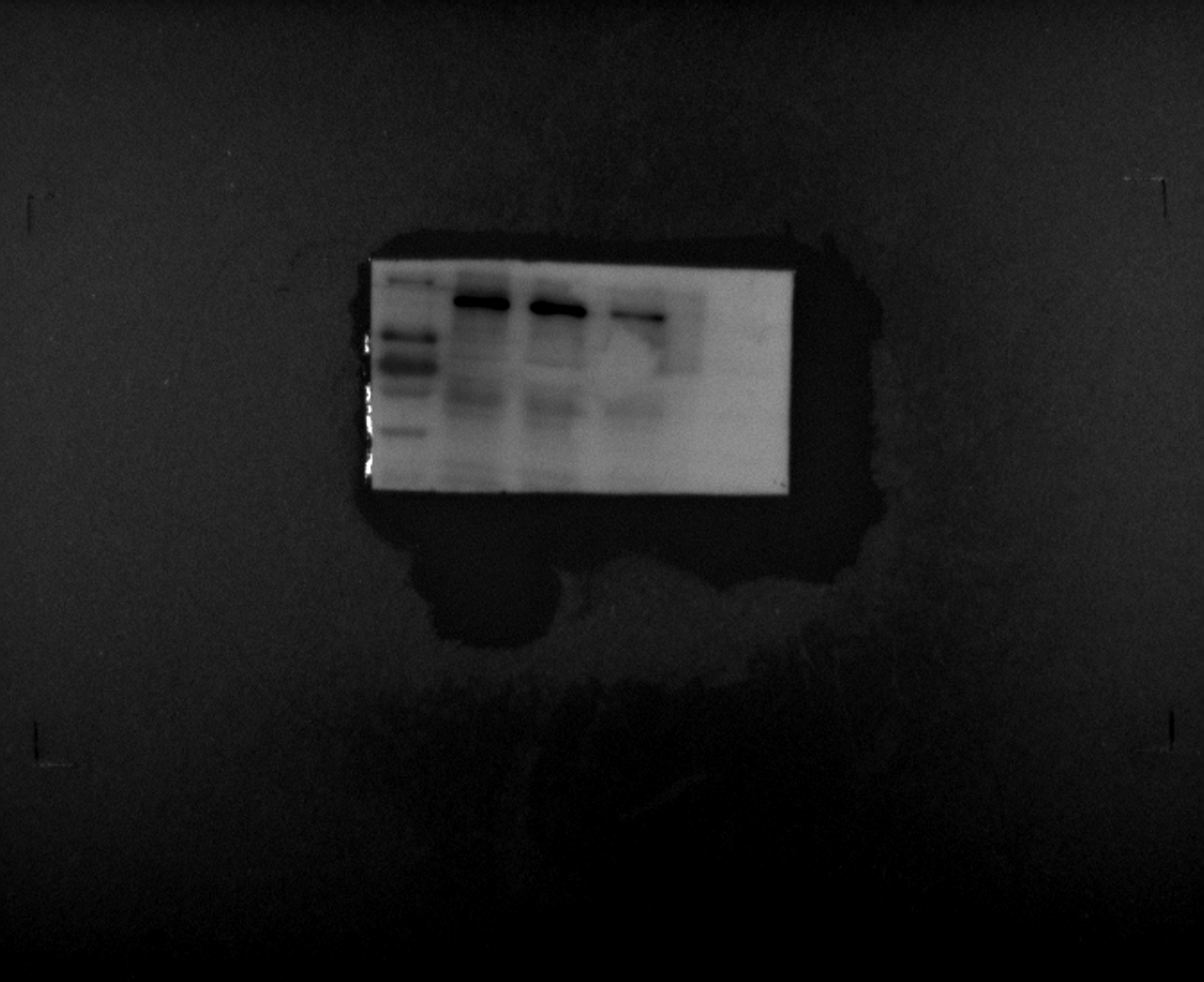

Supplement: Supplemental Information 5 [file peerj-12-17874-s005.zip › fig 3G/DUOX2-2 (3).tif]

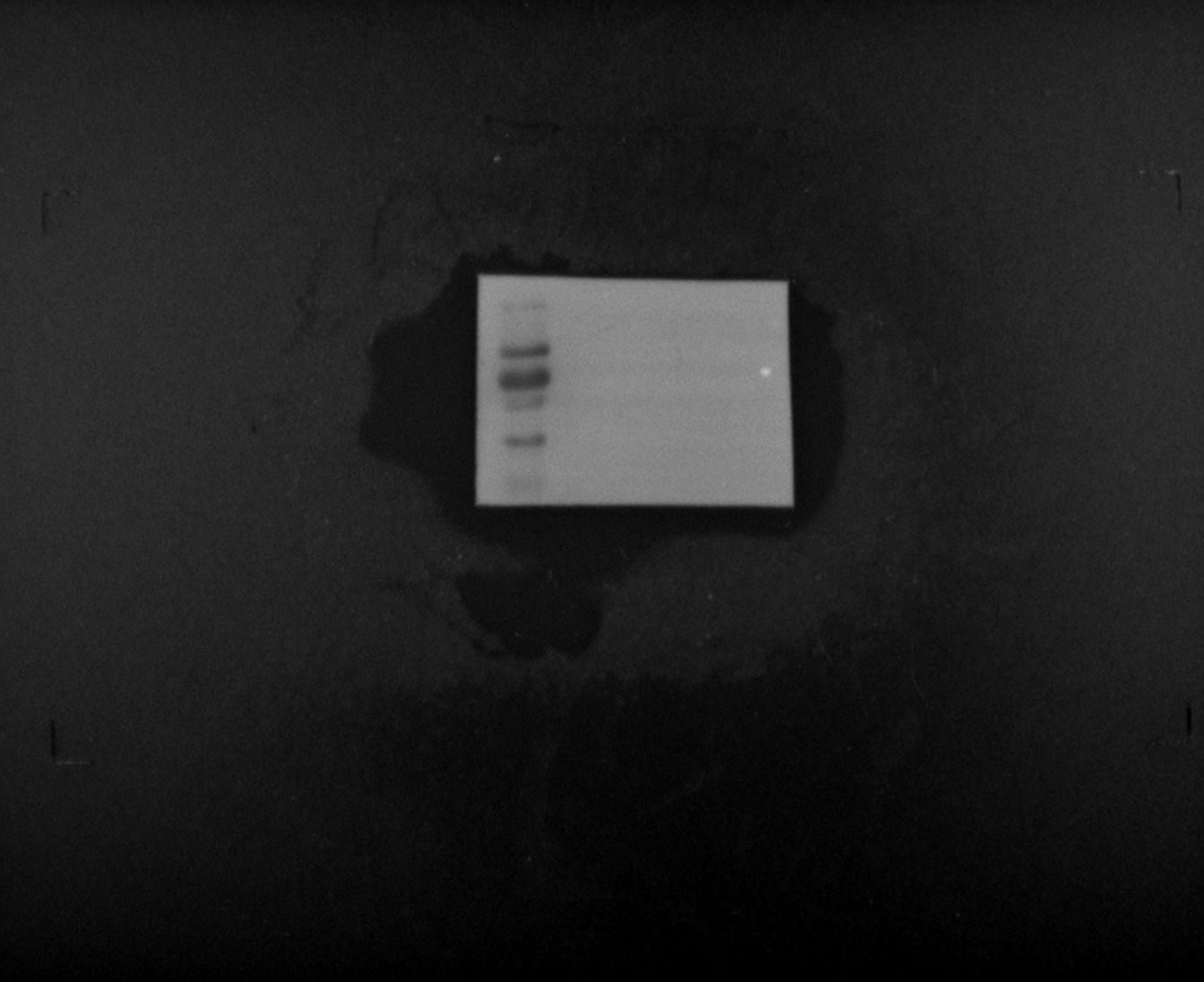

Supplement: Supplemental Information 5 [file peerj-12-17874-s005.zip › fig 3G/DUOX2-3 (1).tif]

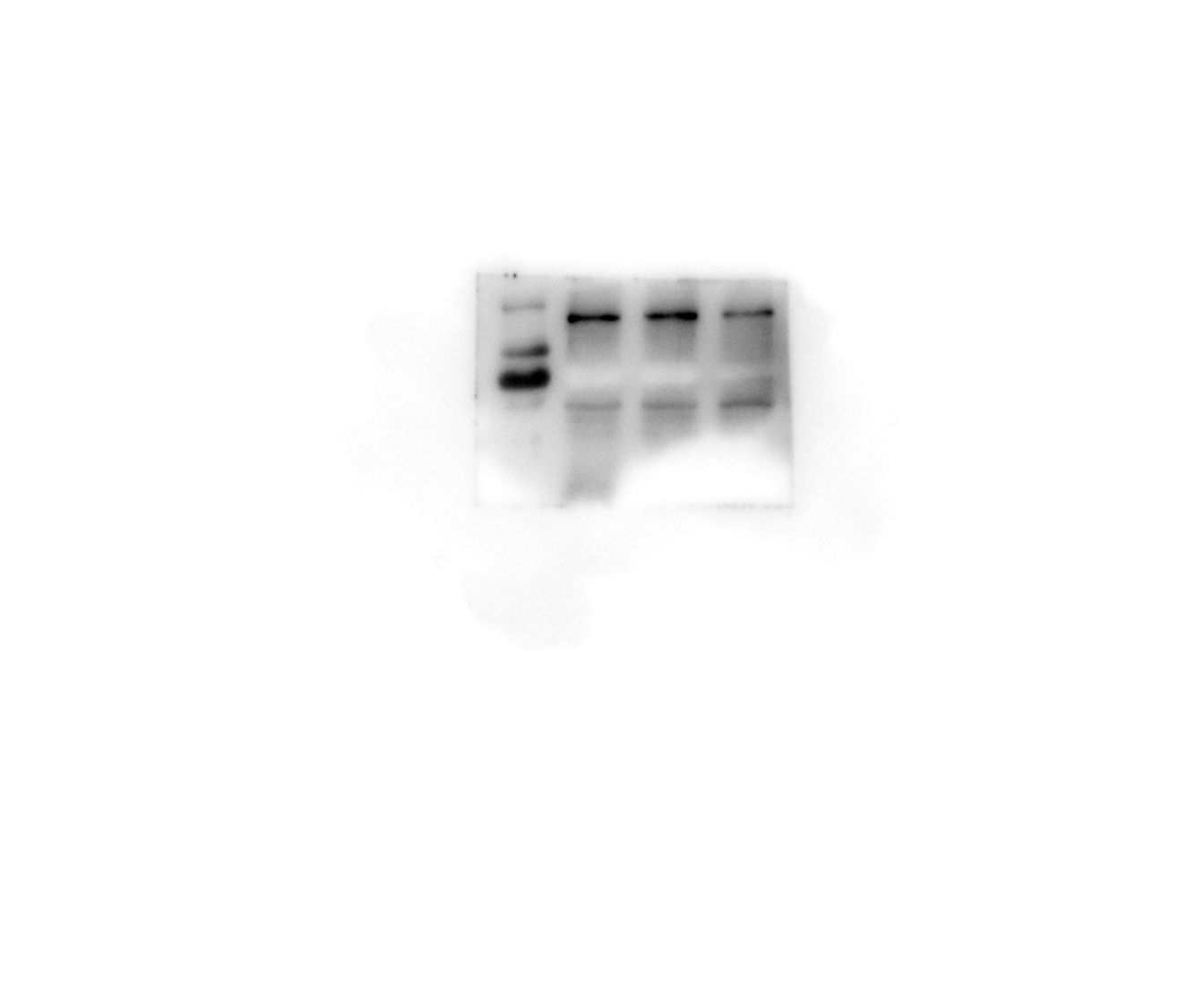

Supplement: Supplemental Information 5 [file peerj-12-17874-s005.zip › fig 3G/DUOX2-3 (2).tif]

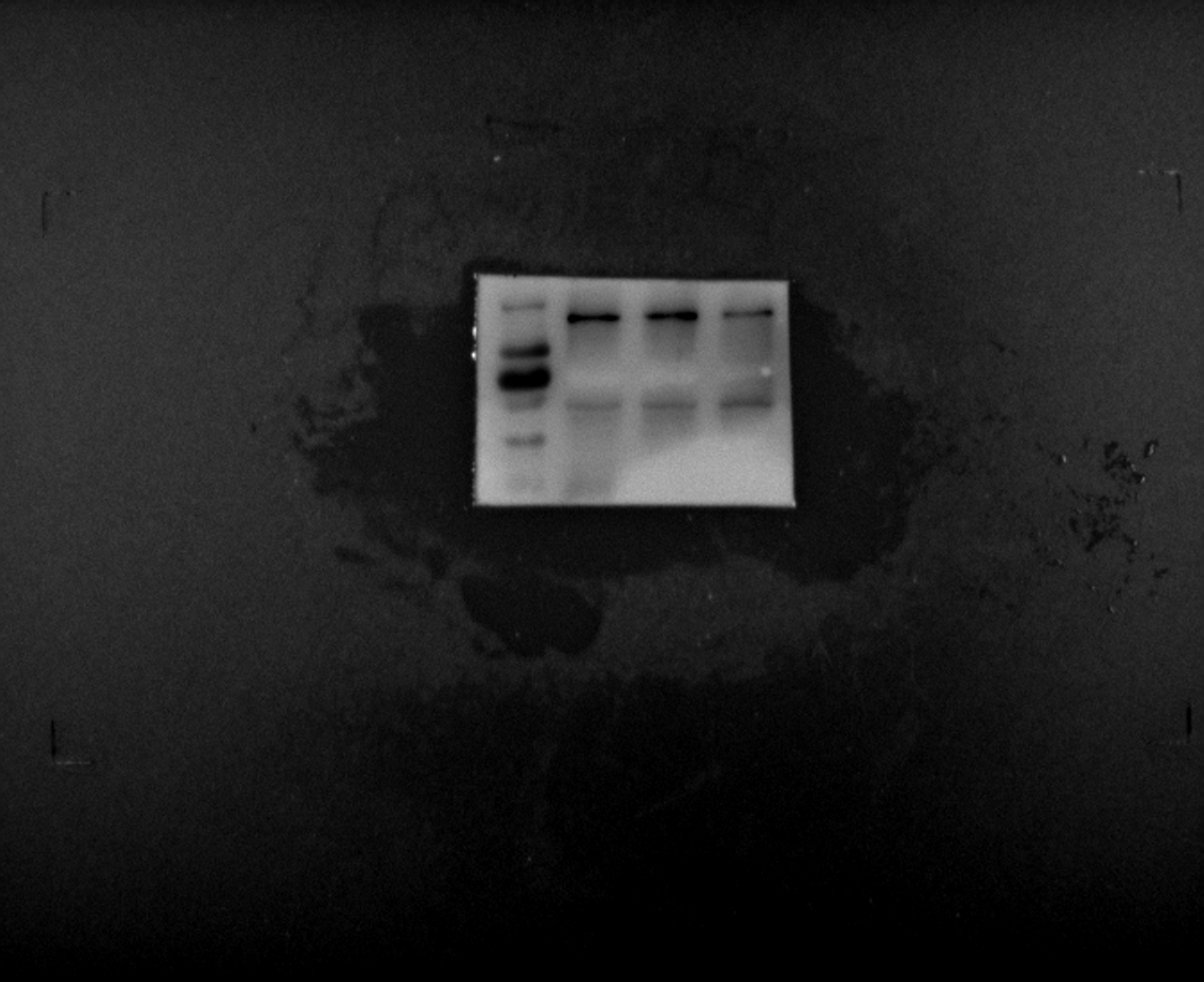

Supplement: Supplemental Information 5 [file peerj-12-17874-s005.zip › fig 3G/DUOX2-3 (3).tif]

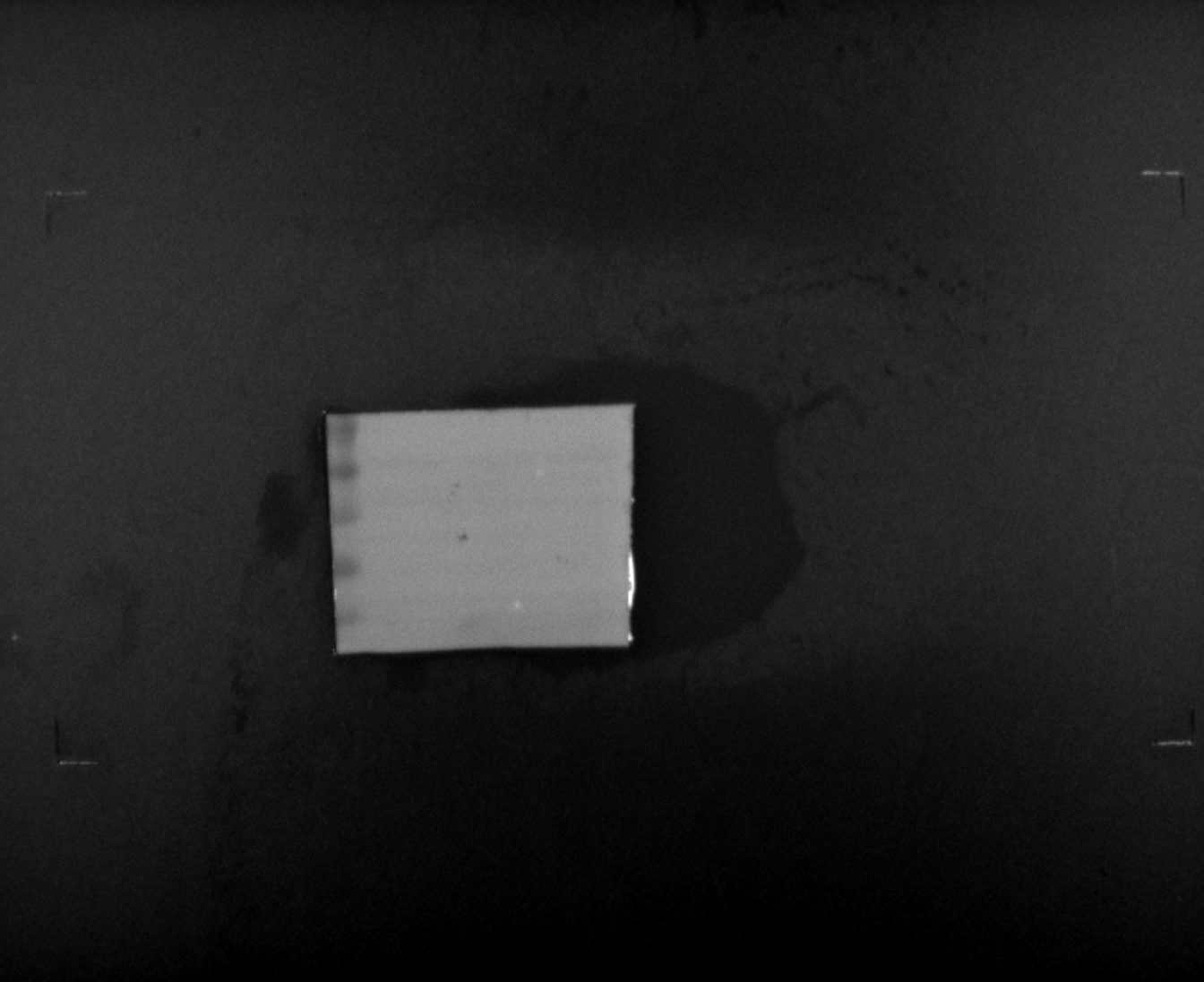

Supplement: Supplemental Information 5 [file peerj-12-17874-s005.zip › fig 3G/gapdh (1).tif]

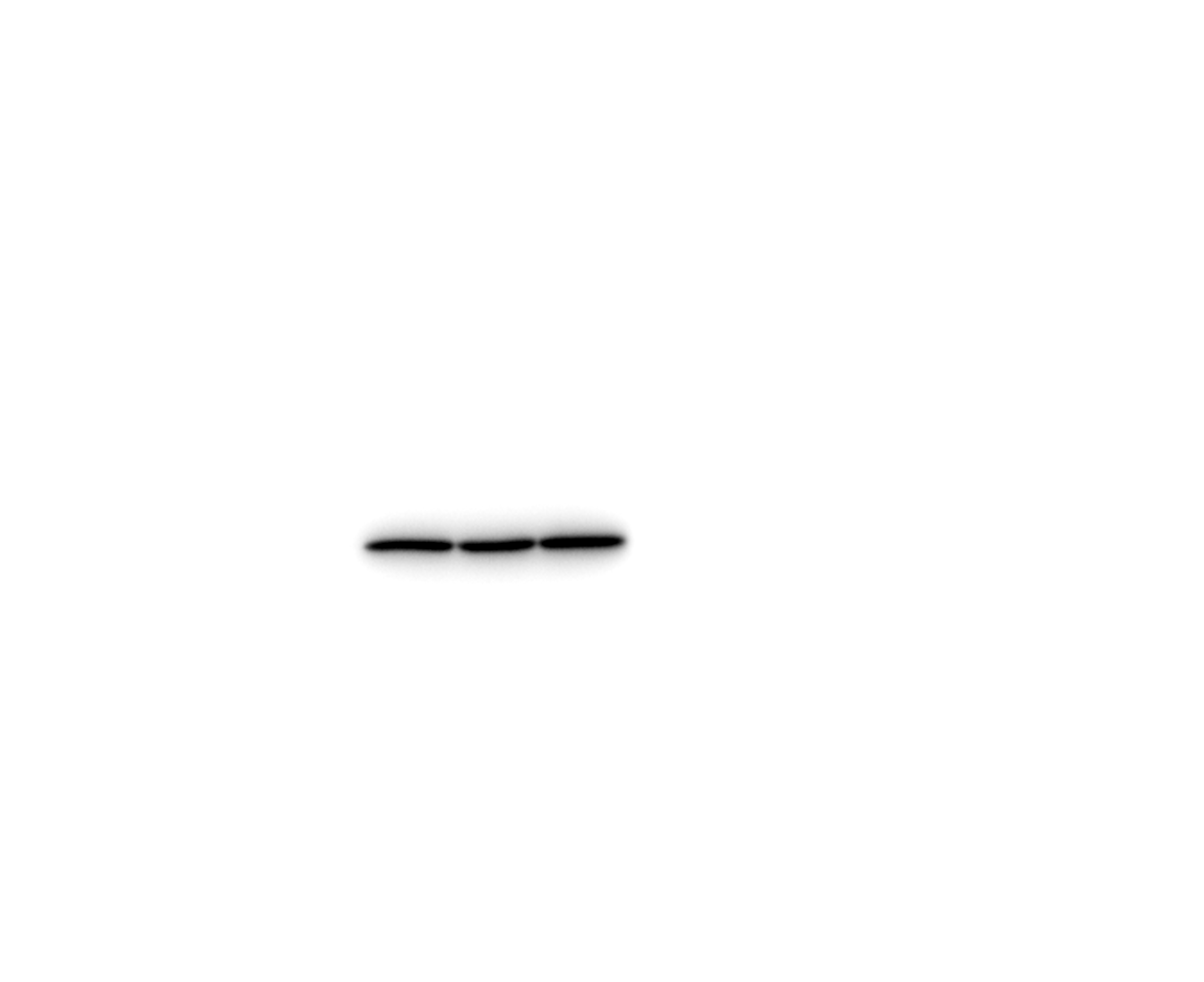

Supplement: Supplemental Information 5 [file peerj-12-17874-s005.zip › fig 3G/gapdh (2).tif]

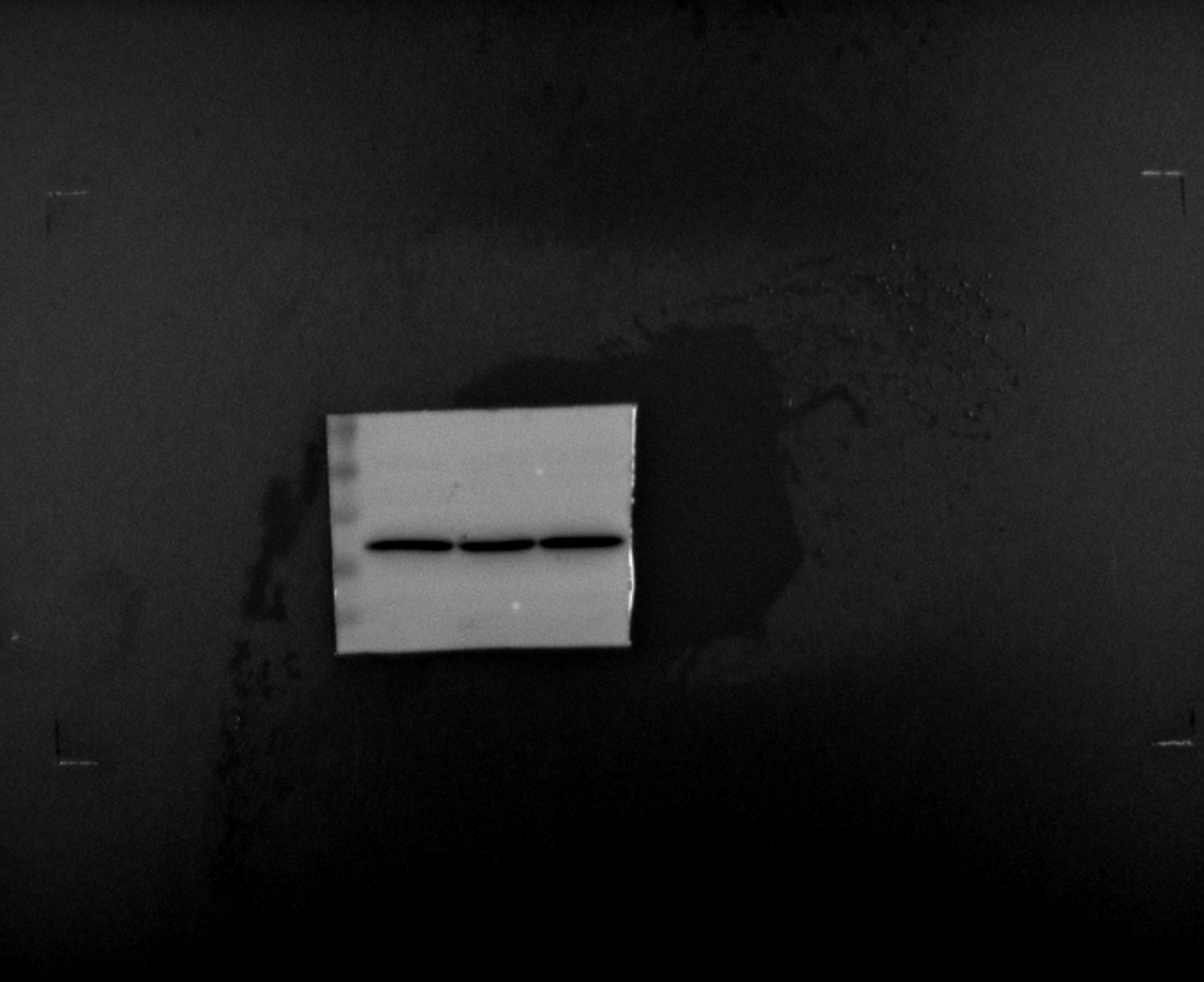

Supplement: Supplemental Information 5 [file peerj-12-17874-s005.zip › fig 3G/gapdh (3).tif]

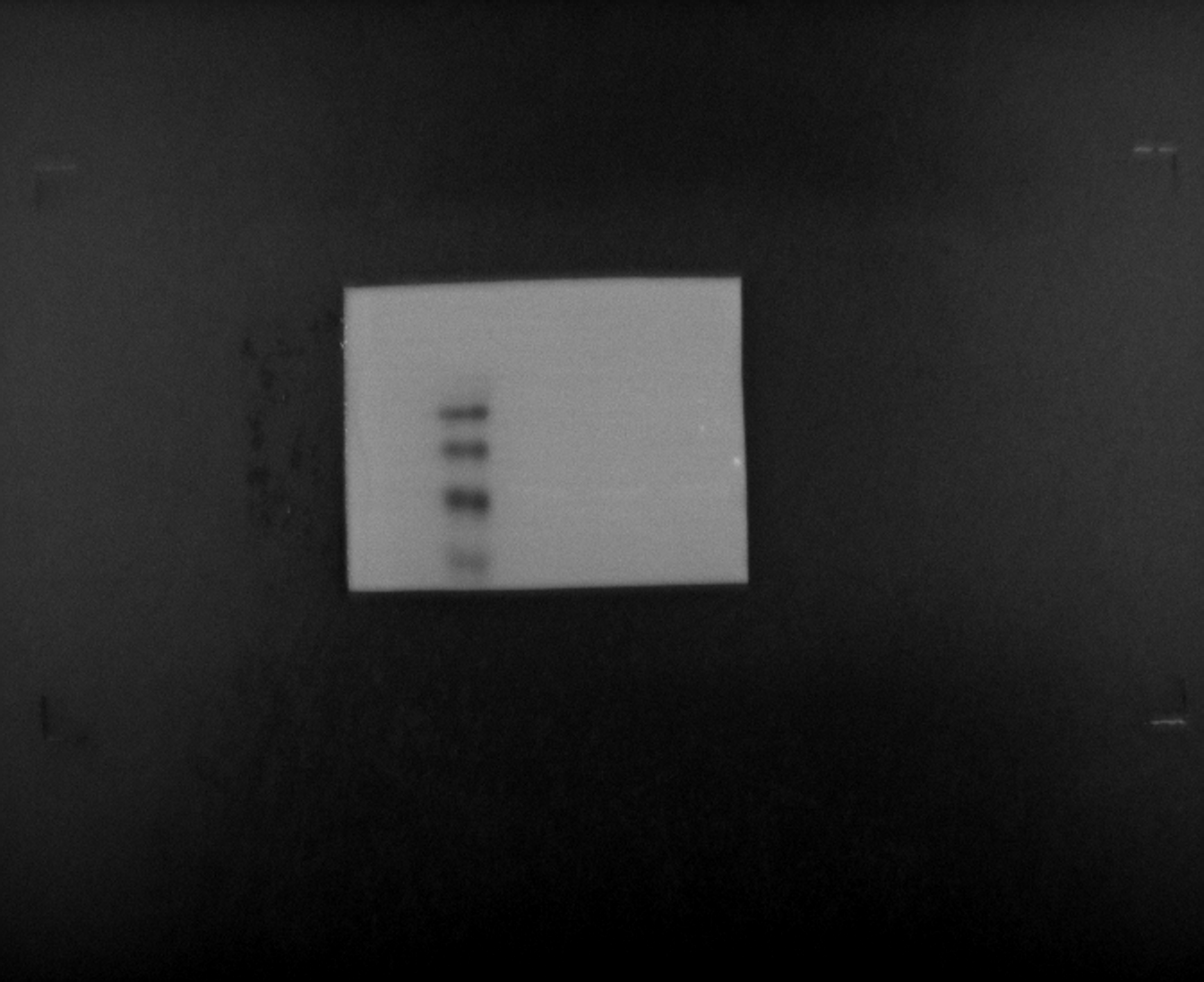

Supplement: Supplemental Information 5 [file peerj-12-17874-s005.zip › fig 3G/gapdh-2 (1).tif]

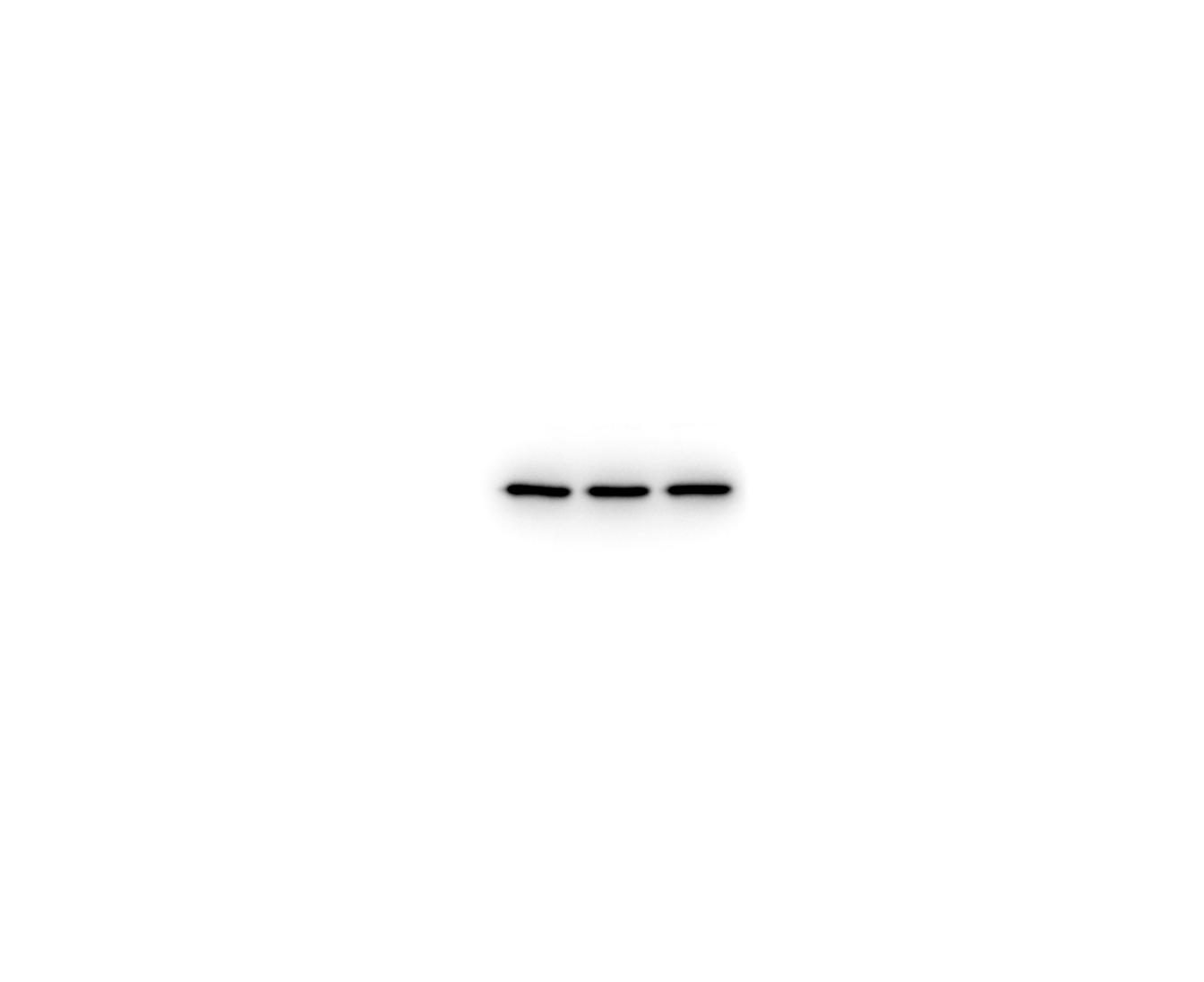

Supplement: Supplemental Information 5 [file peerj-12-17874-s005.zip › fig 3G/gapdh-2 (2).tif]

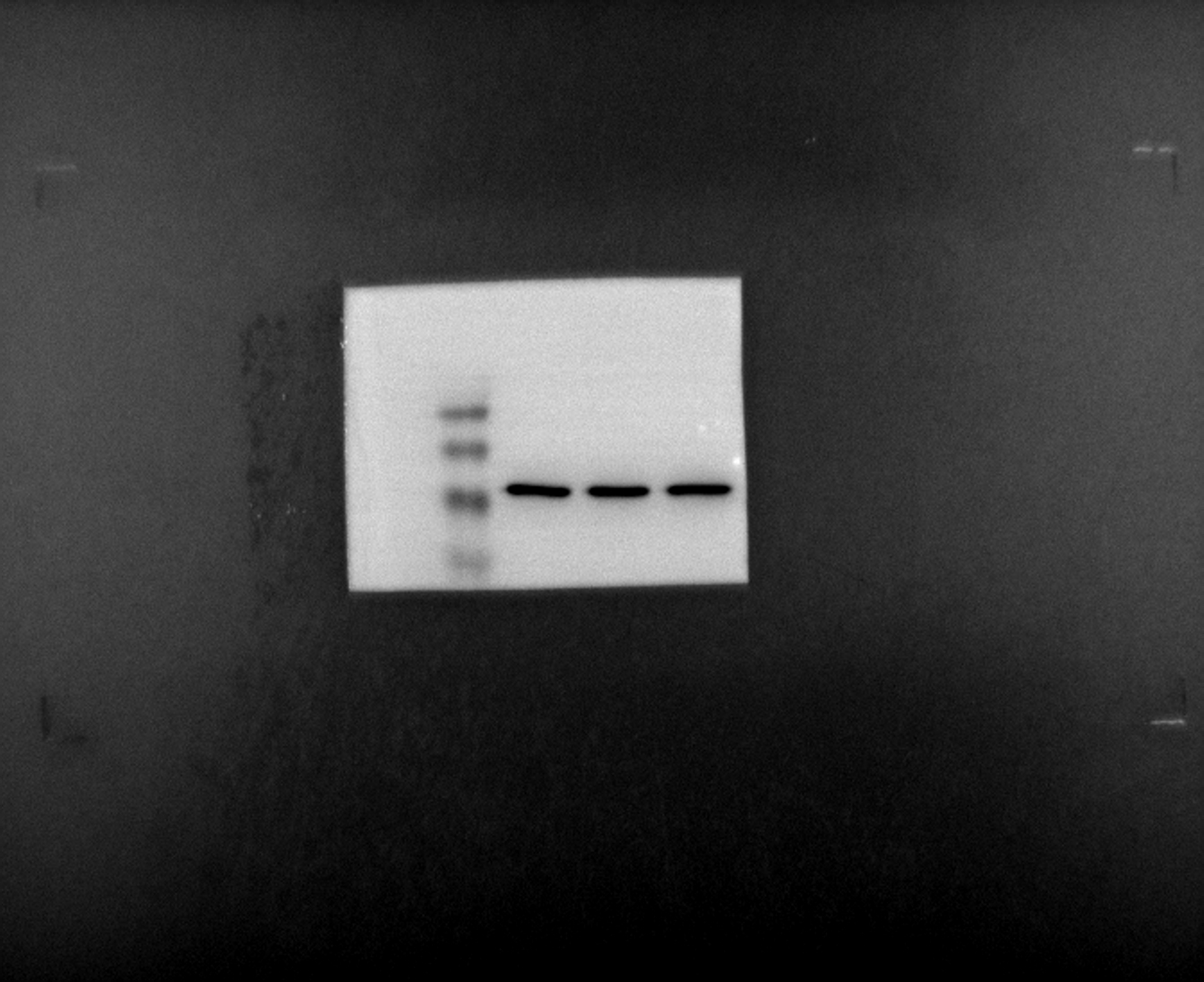

Supplement: Supplemental Information 5 [file peerj-12-17874-s005.zip › fig 3G/gapdh-2 (3).tif]

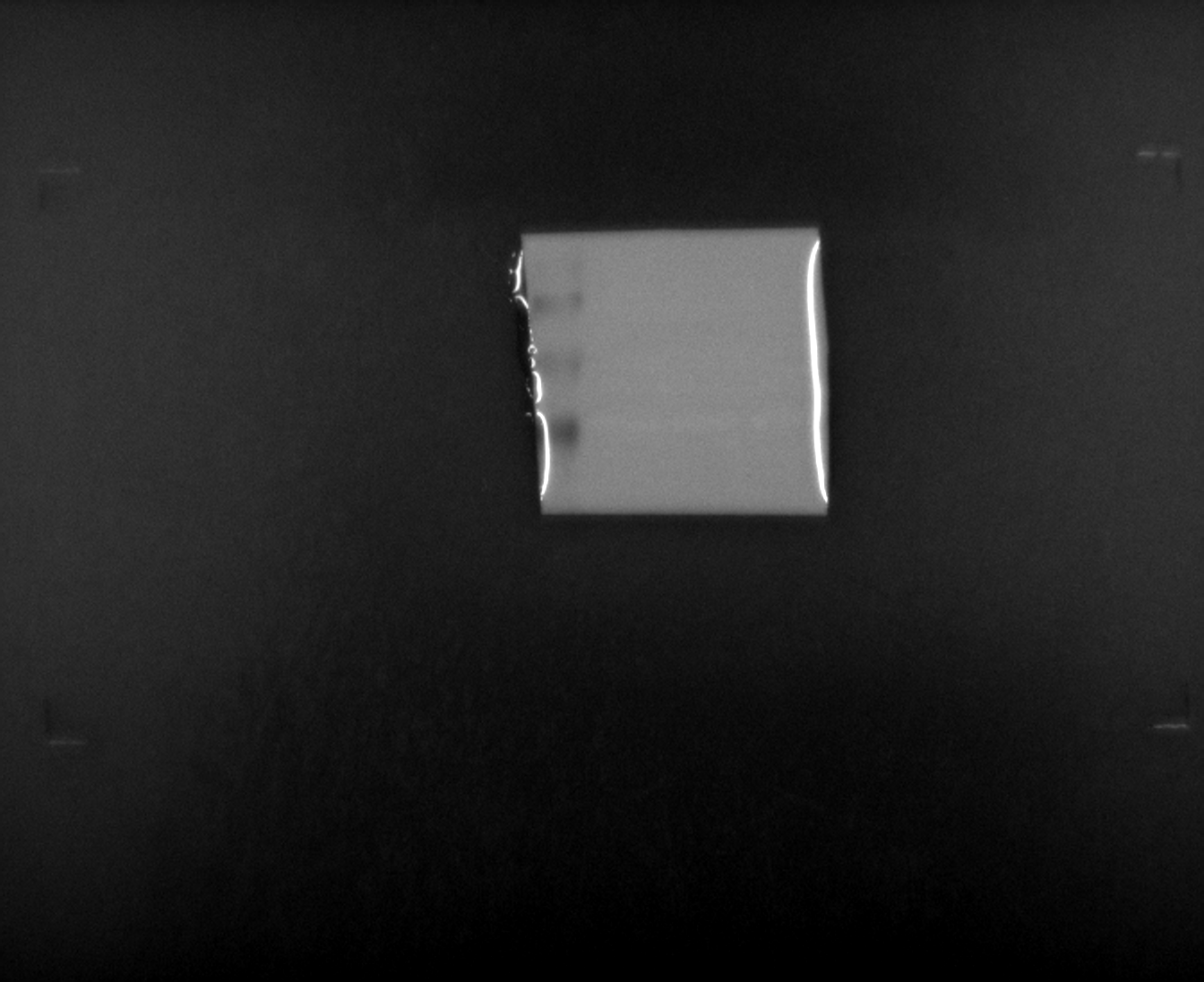

Supplement: Supplemental Information 5 [file peerj-12-17874-s005.zip › fig 3G/GAPDH-3 (1).tif]

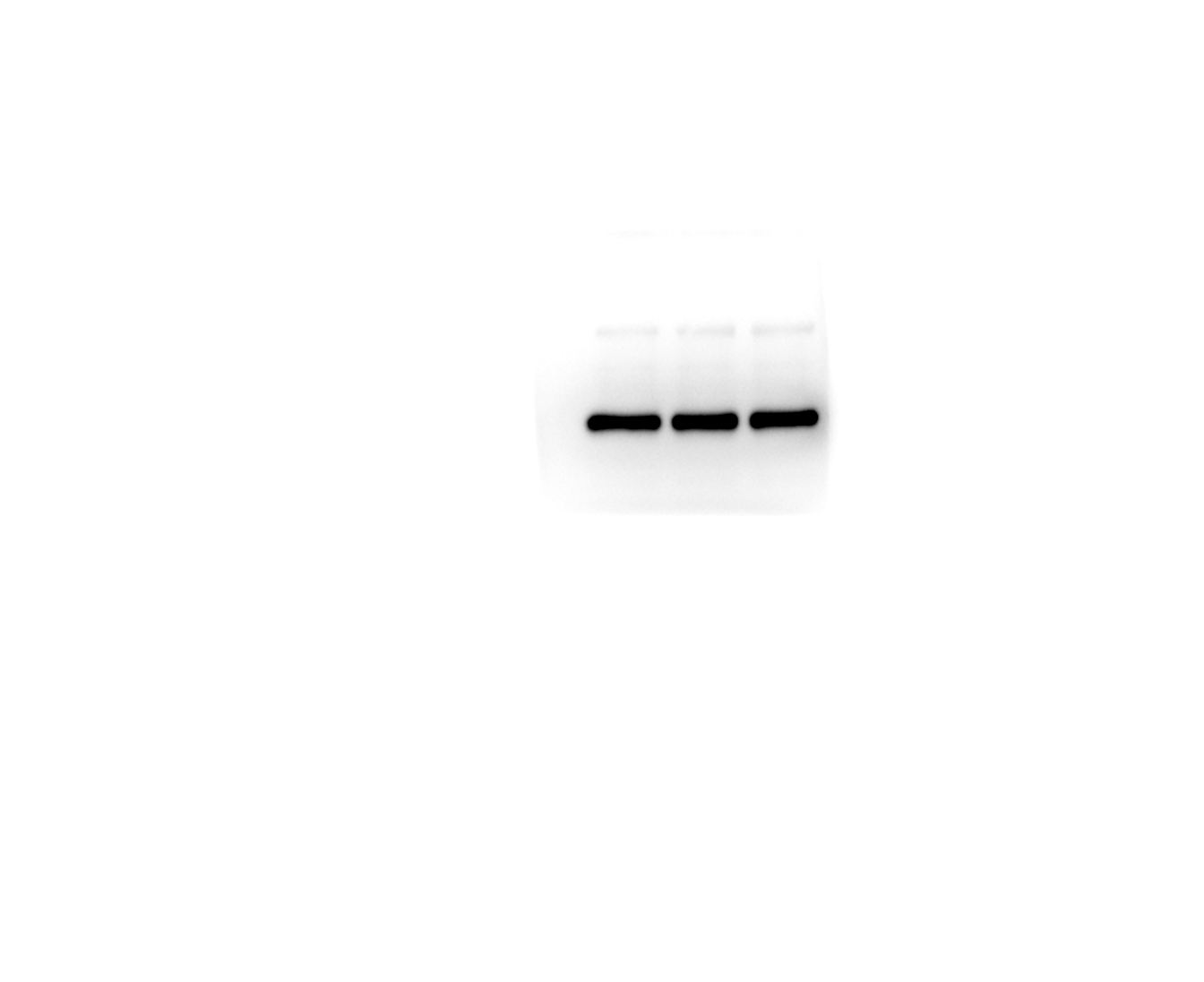

Supplement: Supplemental Information 5 [file peerj-12-17874-s005.zip › fig 3G/GAPDH-3 (2).tif]

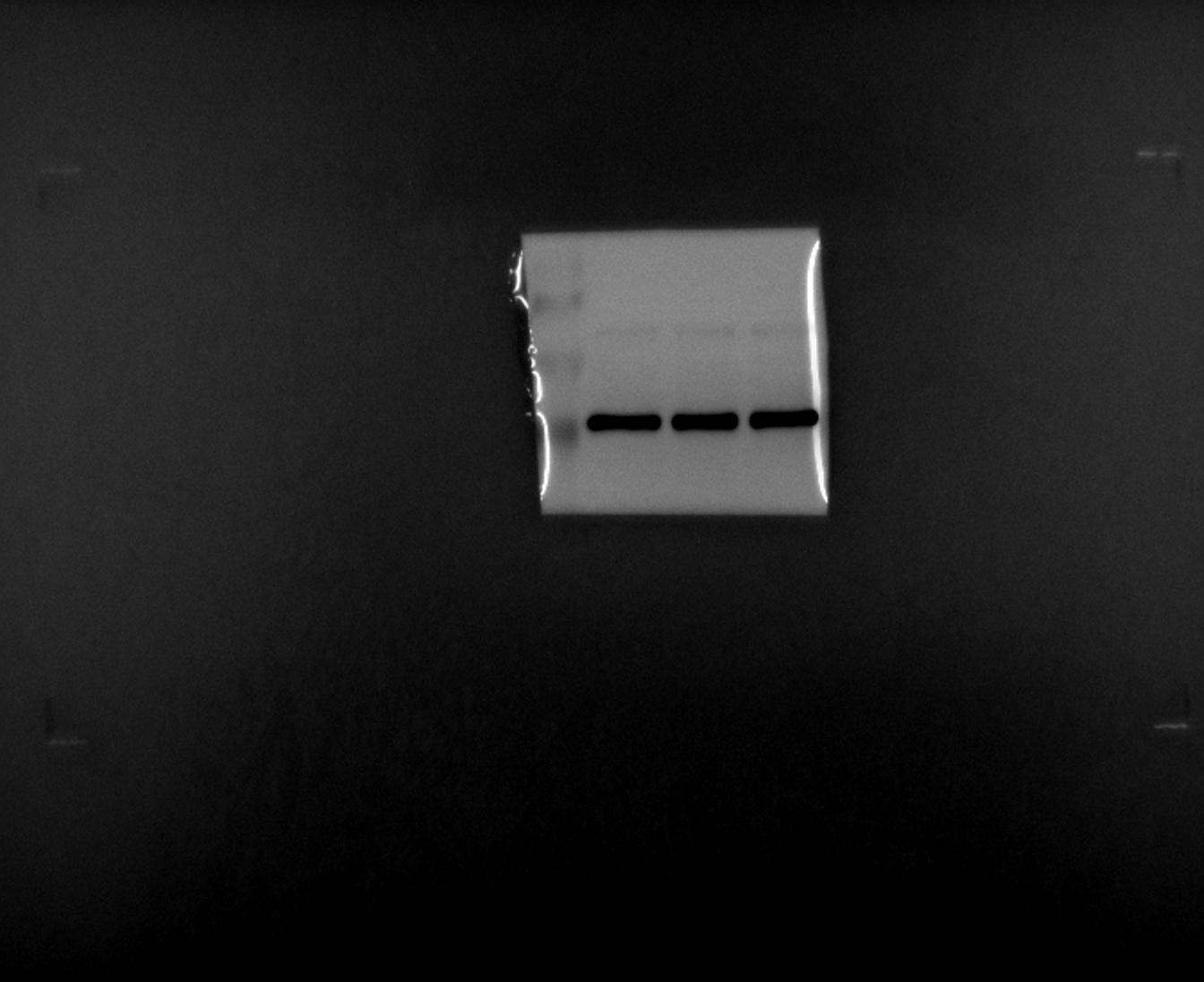

Supplement: Supplemental Information 5 [file peerj-12-17874-s005.zip › fig 3G/GAPDH-3 (3).tif]

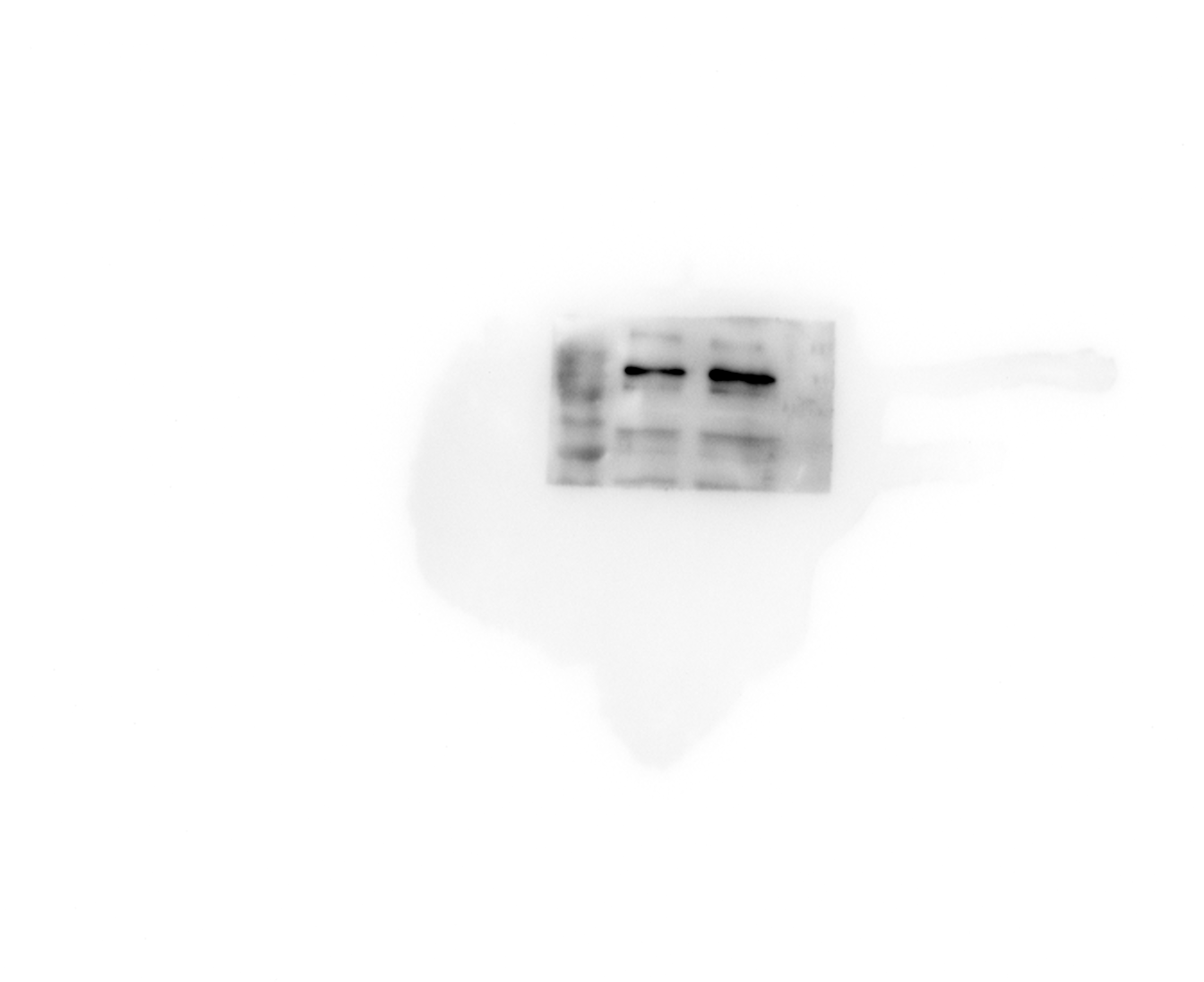

Supplement: Supplemental Information 6 [file peerj-12-17874-s006.zip › fig 4B/DUOX2 (1).tif]

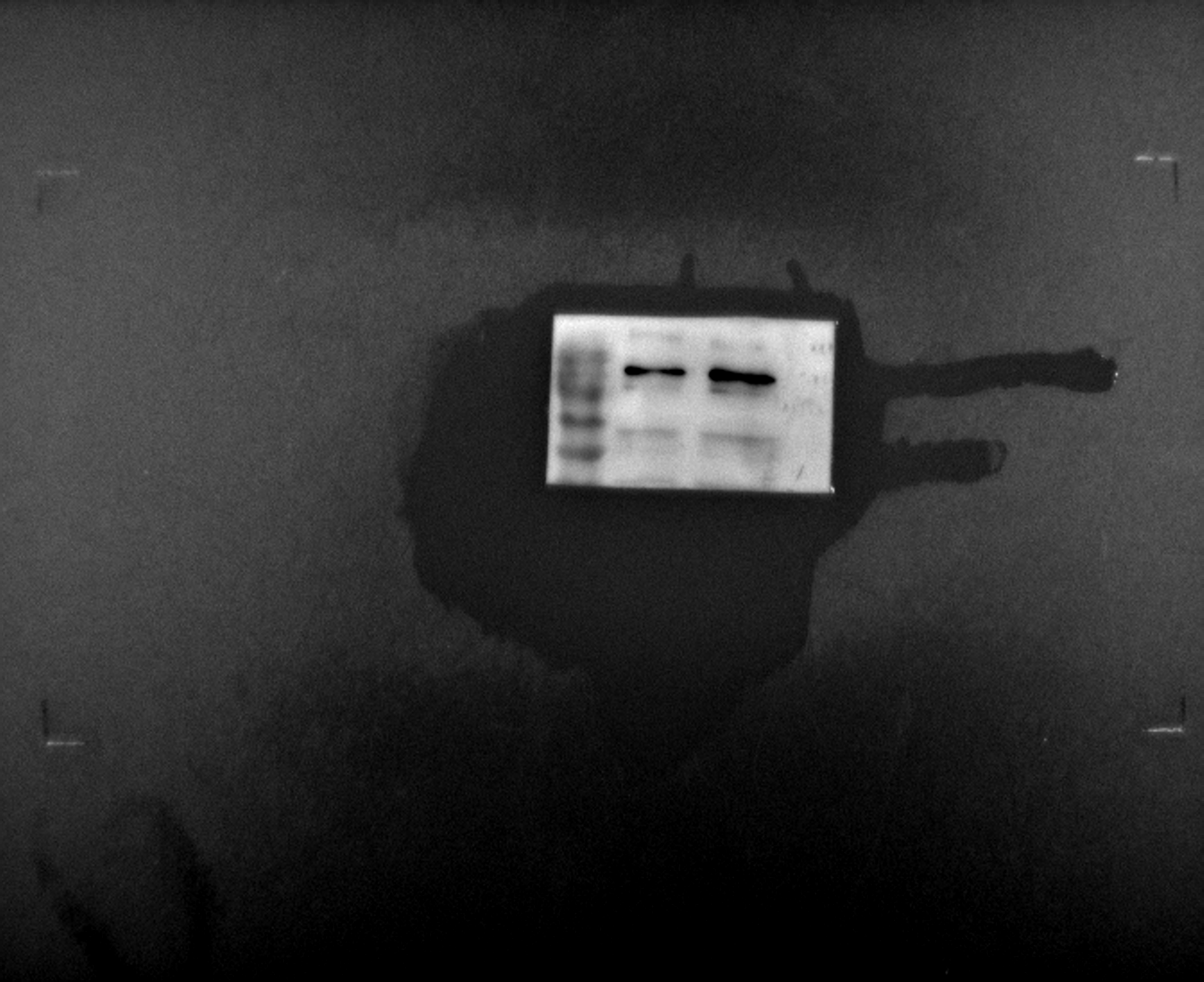

Supplement: Supplemental Information 6 [file peerj-12-17874-s006.zip › fig 4B/DUOX2 (2).tif]

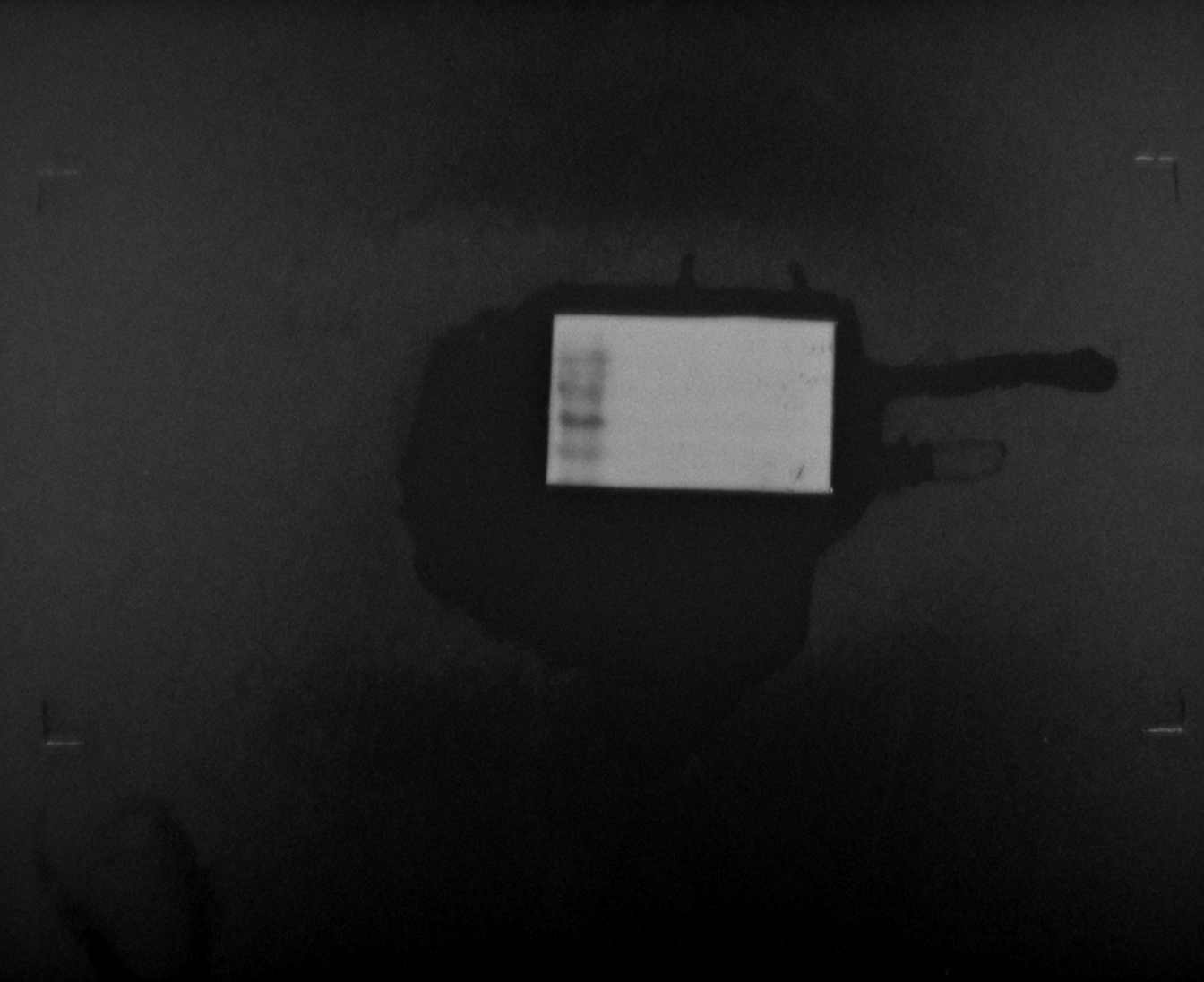

Supplement: Supplemental Information 6 [file peerj-12-17874-s006.zip › fig 4B/DUOX2 (3).tif]

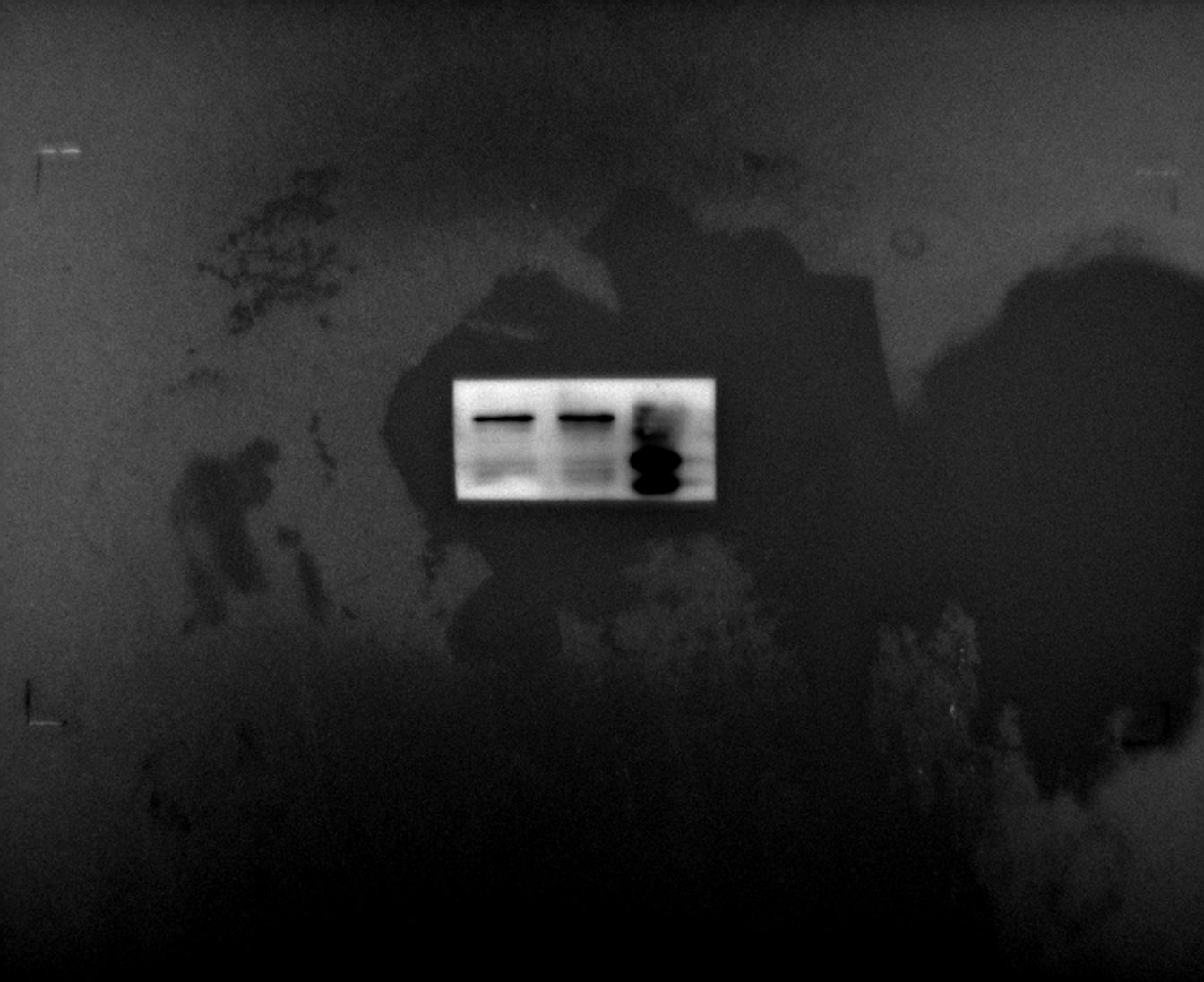

Supplement: Supplemental Information 6 [file peerj-12-17874-s006.zip › fig 4B/duox2-2 (1).tif]

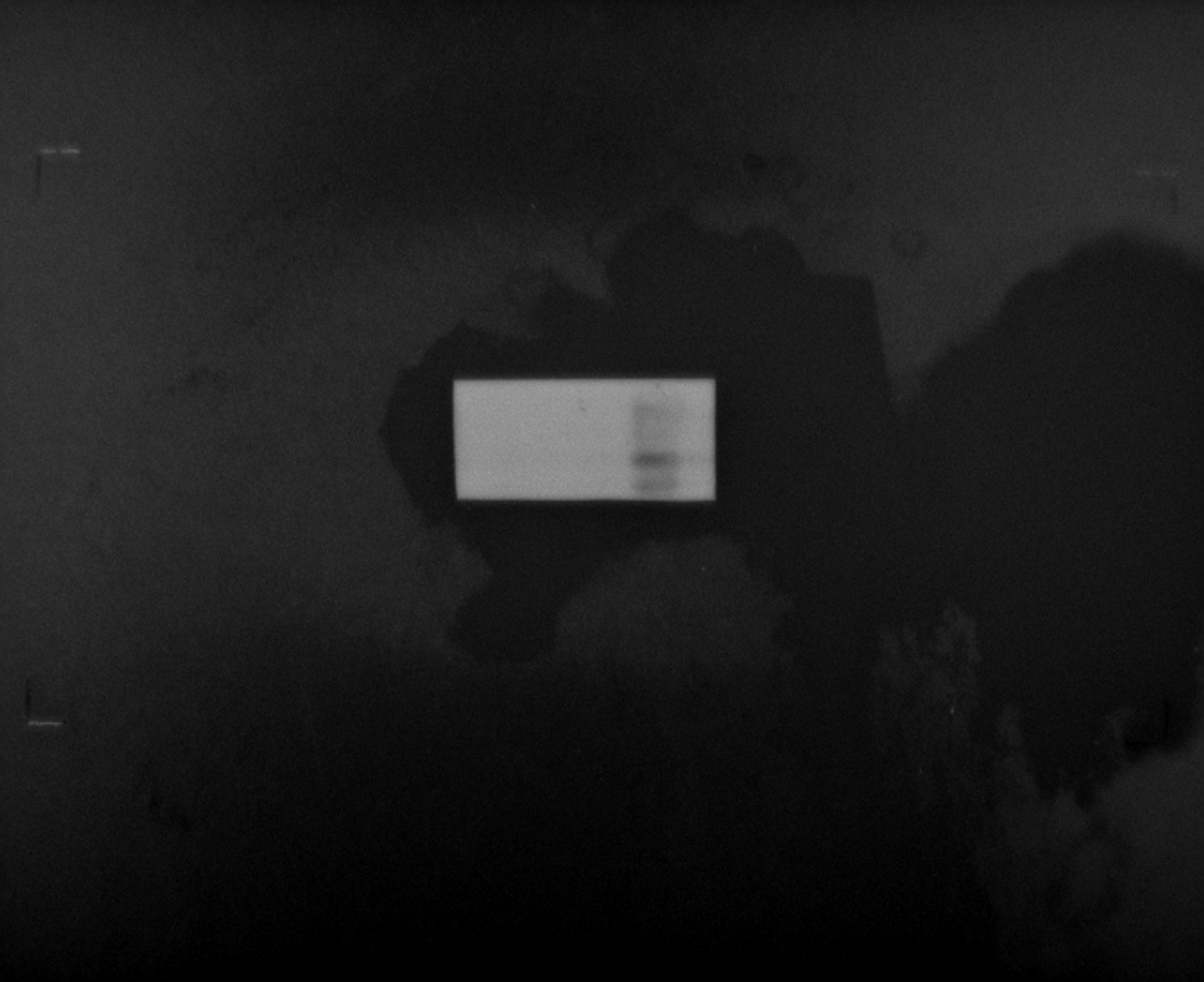

Supplement: Supplemental Information 6 [file peerj-12-17874-s006.zip › fig 4B/duox2-2 (2).tif]

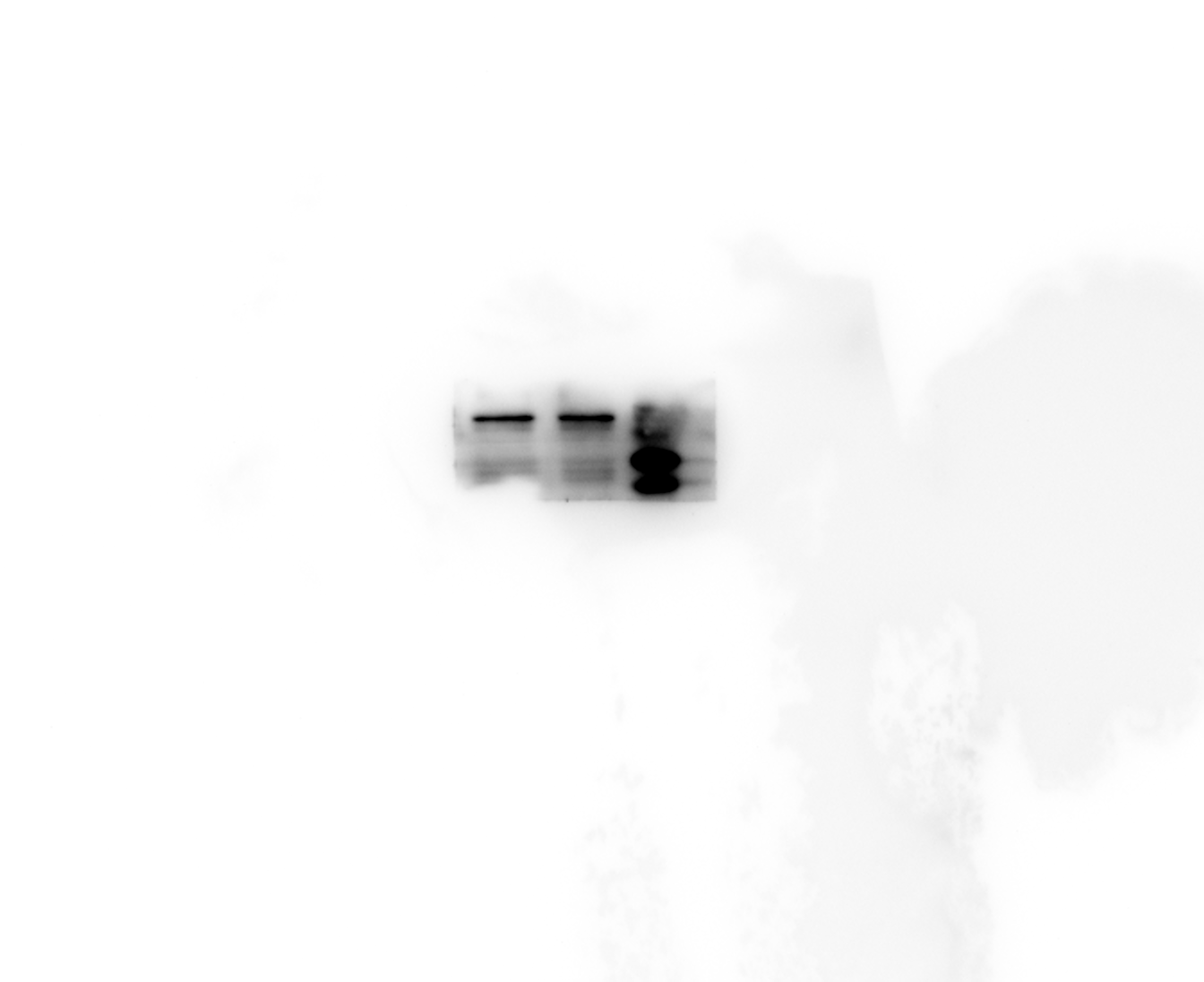

Supplement: Supplemental Information 6 [file peerj-12-17874-s006.zip › fig 4B/duox2-2 (3).tif]

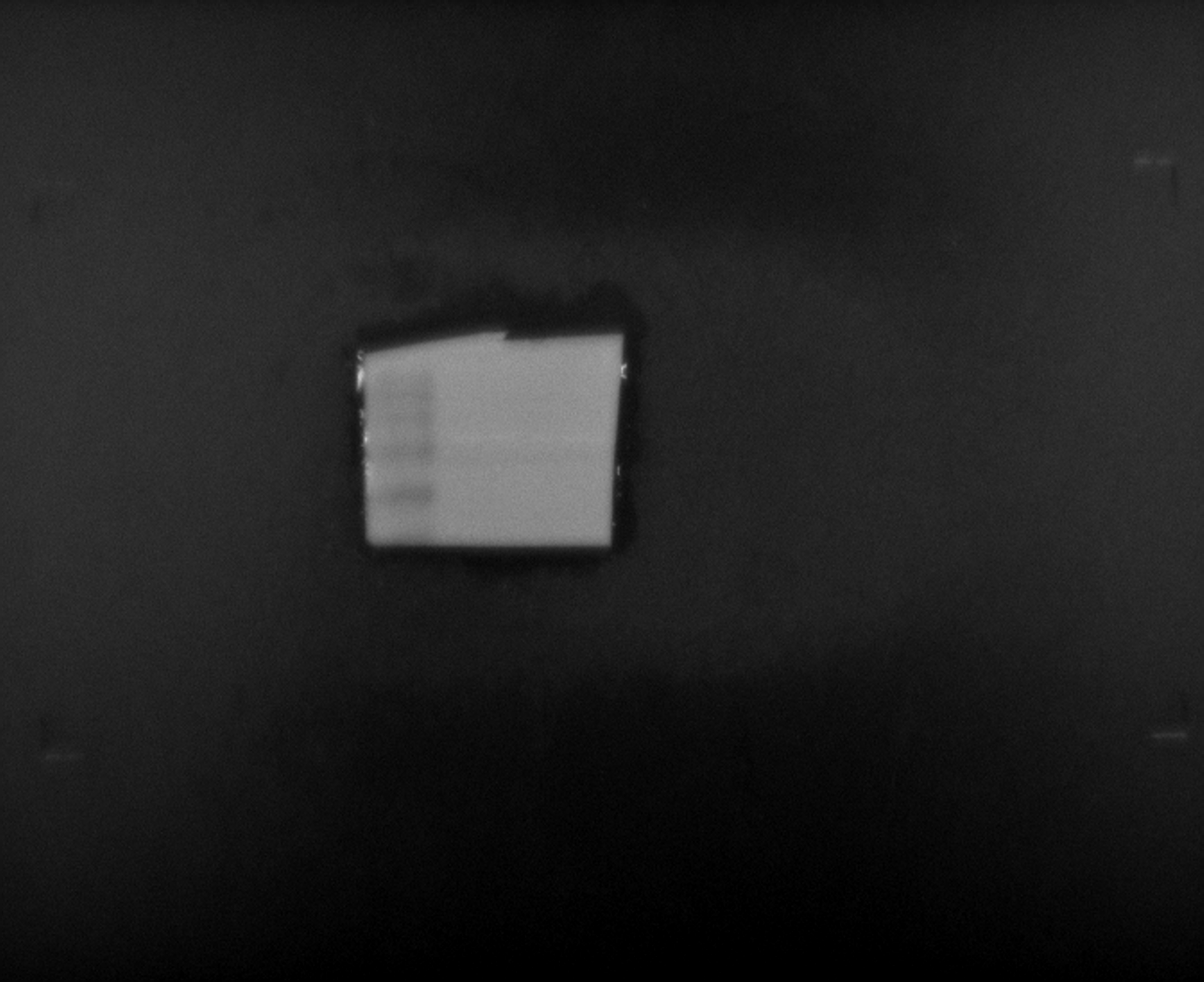

Supplement: Supplemental Information 6 [file peerj-12-17874-s006.zip › fig 4B/DUOX2-3 (1).tif]

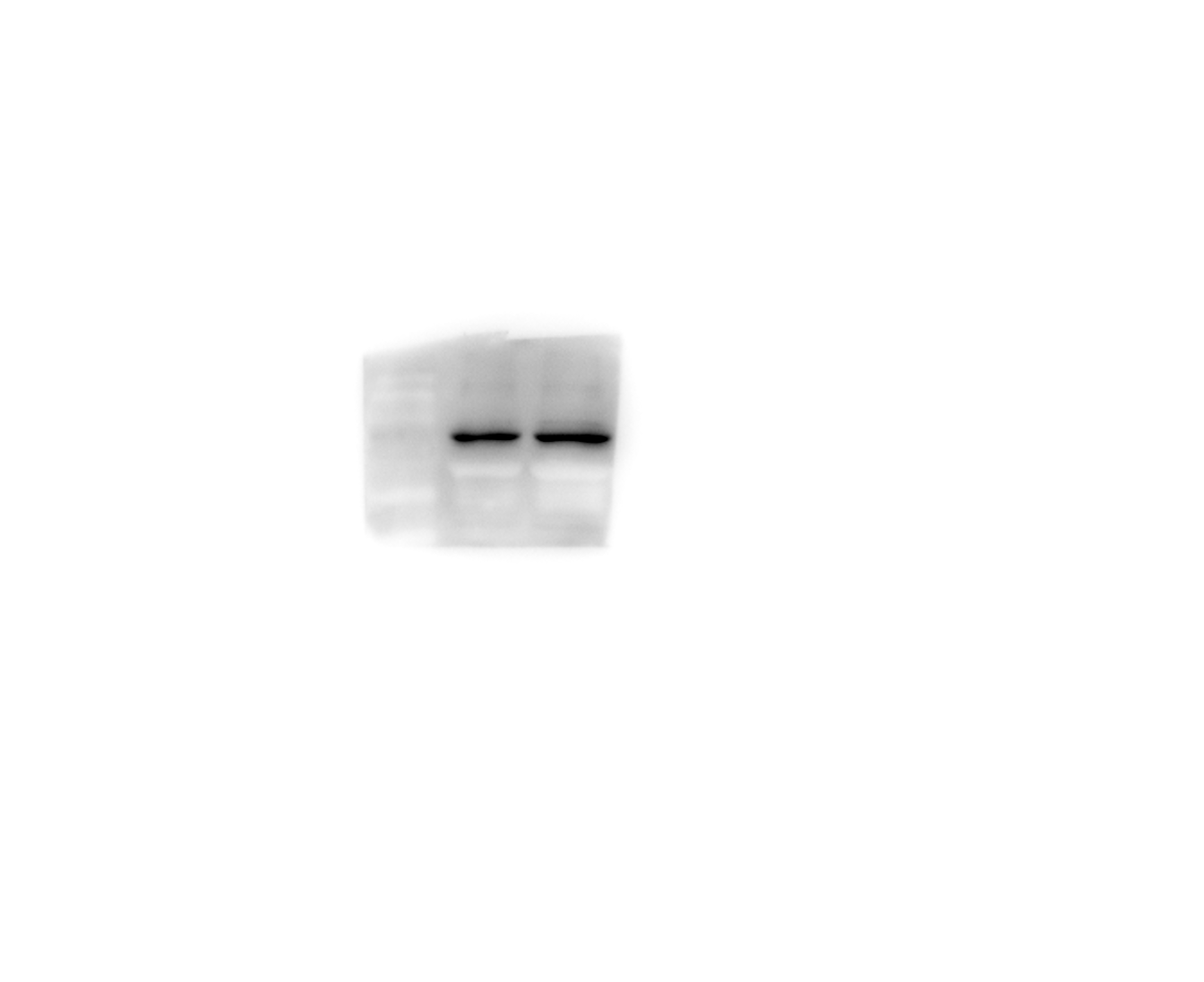

Supplement: Supplemental Information 6 [file peerj-12-17874-s006.zip › fig 4B/DUOX2-3 (2).tif]

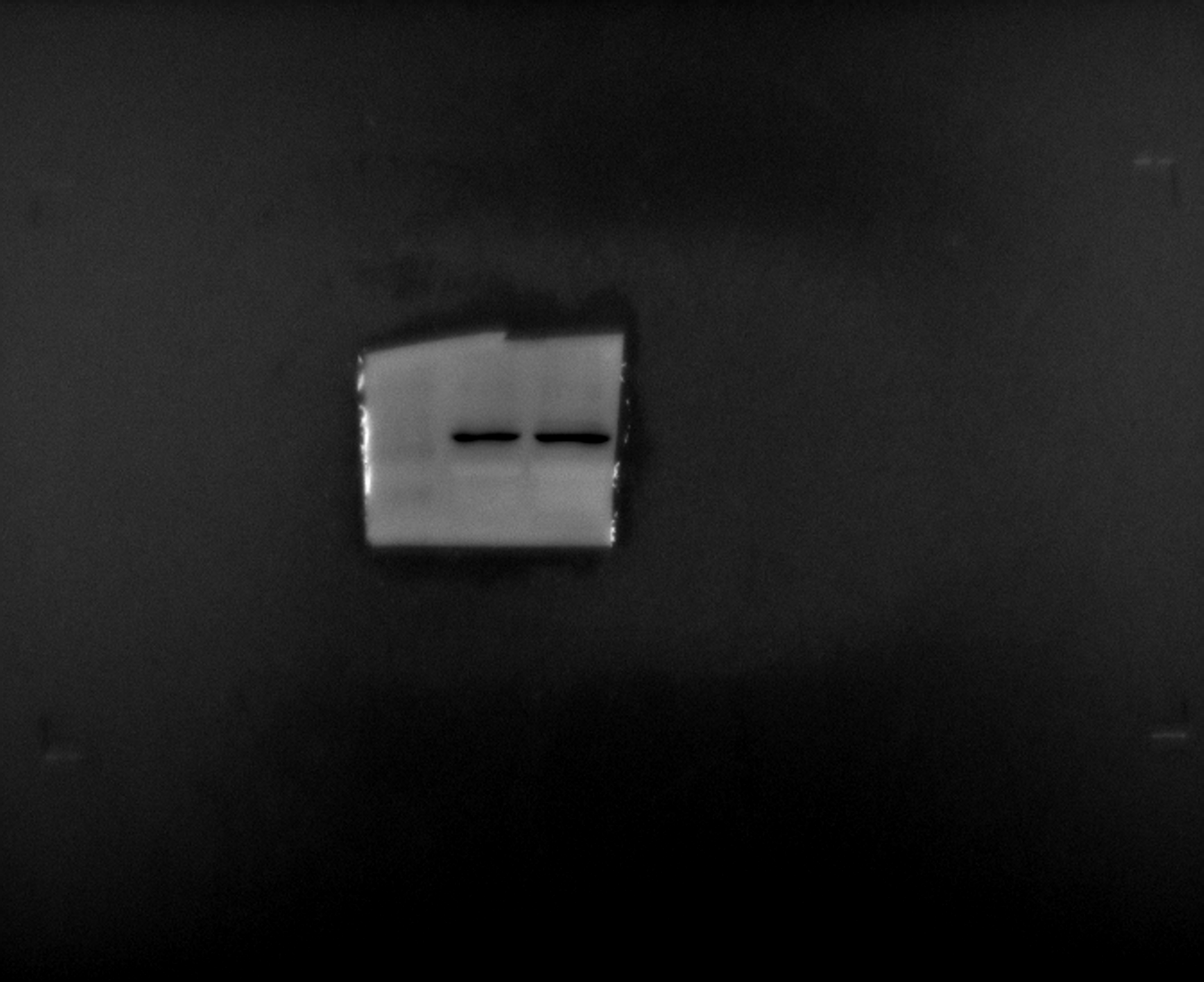

Supplement: Supplemental Information 6 [file peerj-12-17874-s006.zip › fig 4B/DUOX2-3 (3).tif]

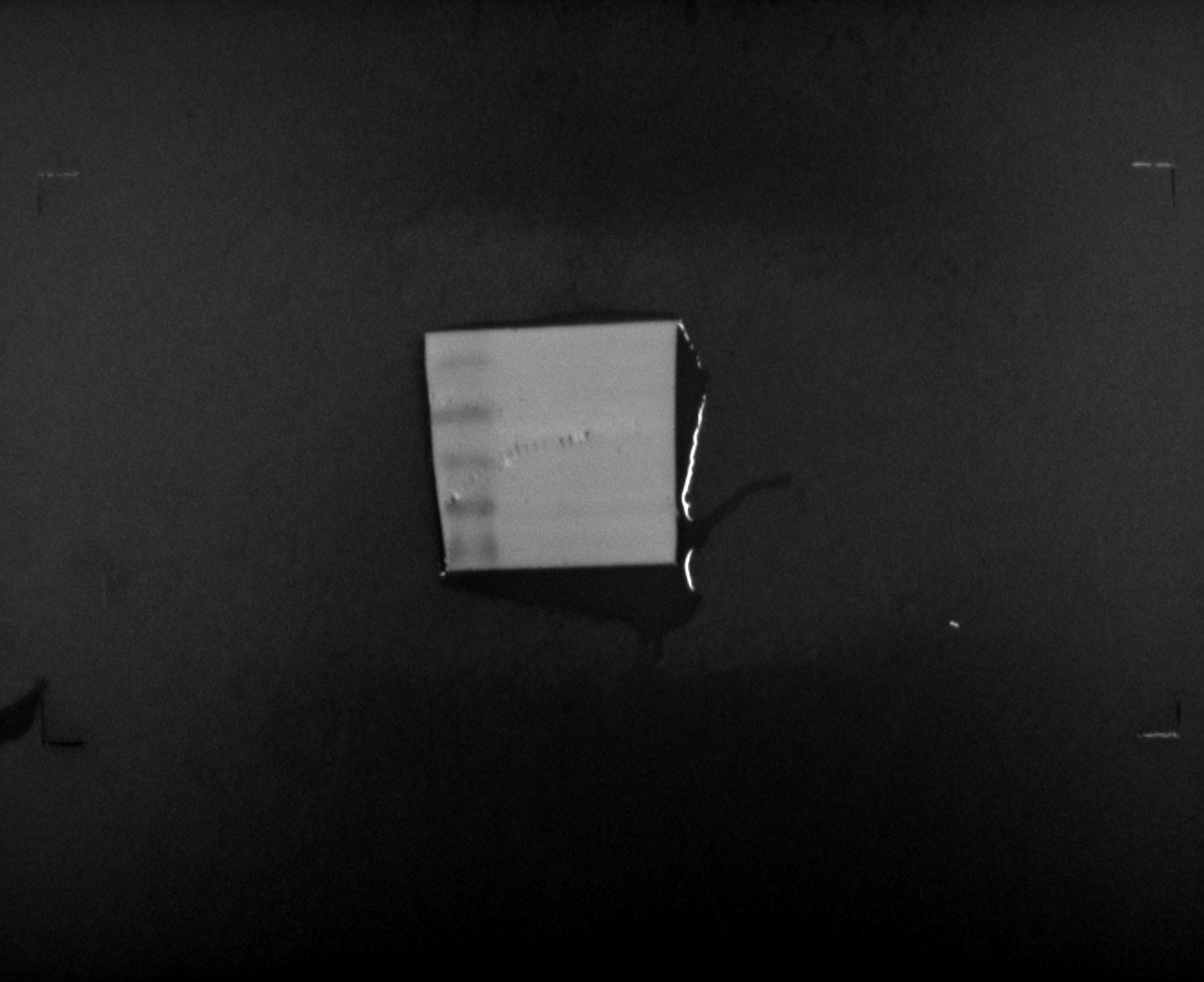

Supplement: Supplemental Information 6 [file peerj-12-17874-s006.zip › fig 4B/GAPDH (4).tif]

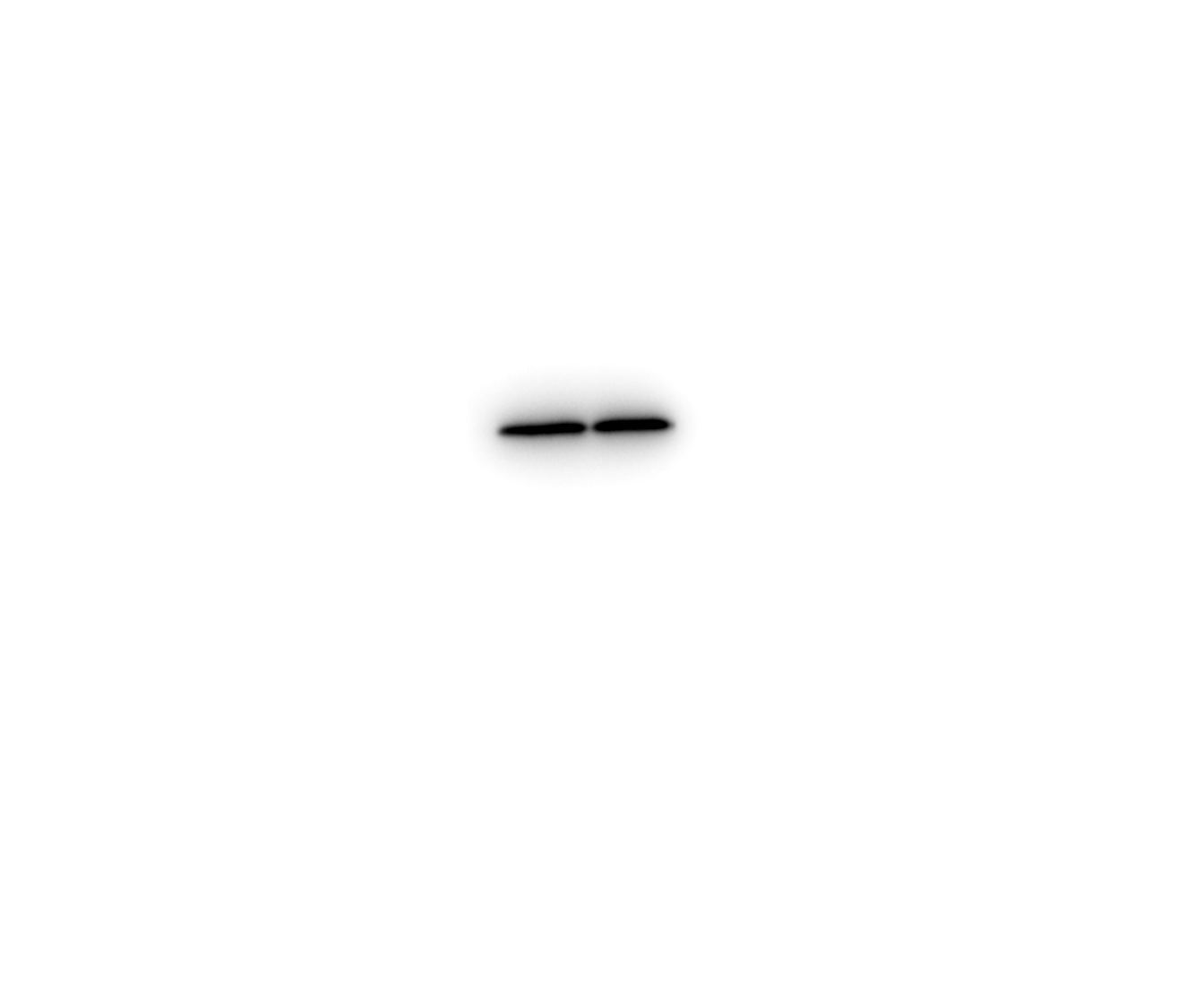

Supplement: Supplemental Information 6 [file peerj-12-17874-s006.zip › fig 4B/GAPDH (5).tif]

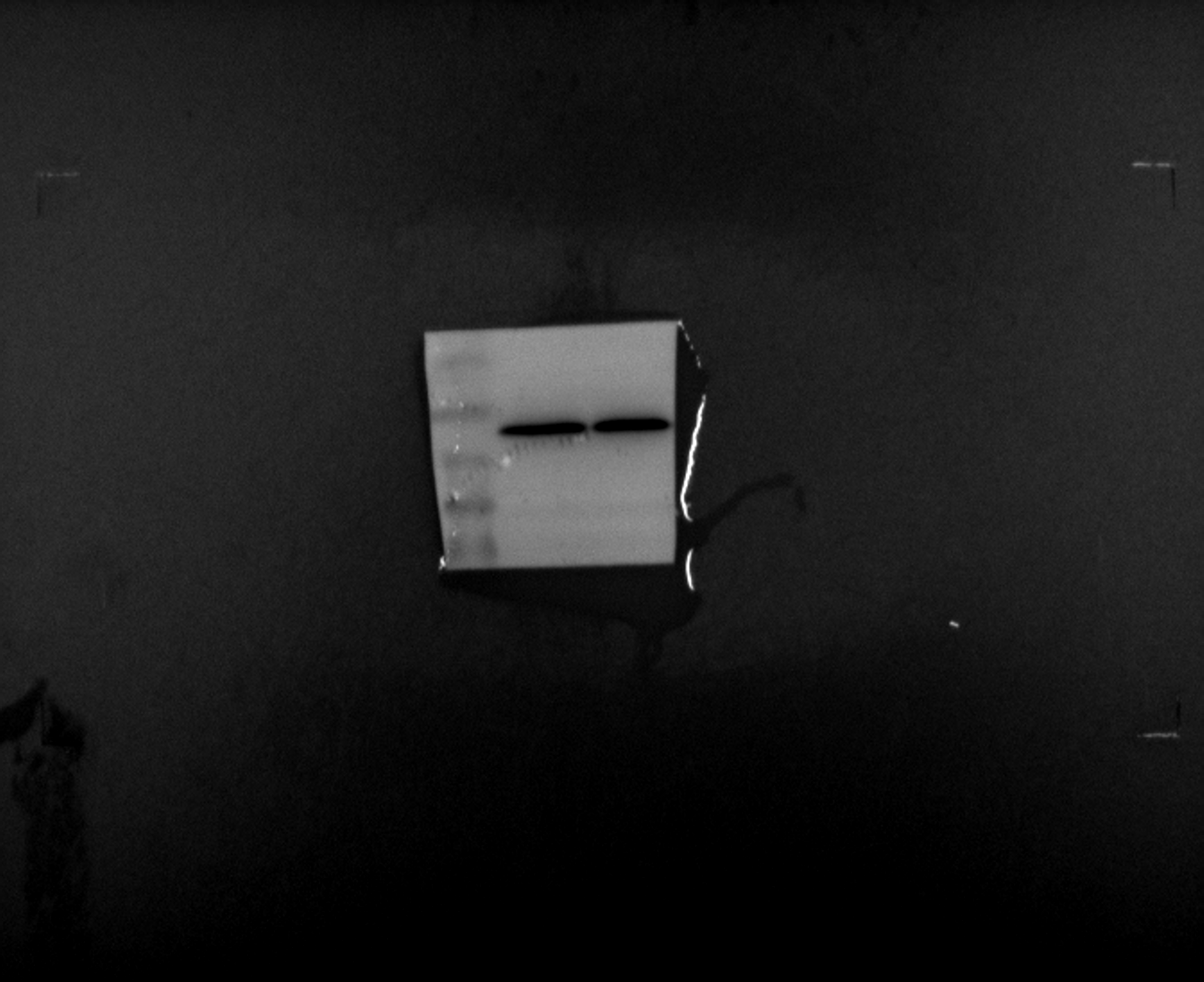

Supplement: Supplemental Information 6 [file peerj-12-17874-s006.zip › fig 4B/GAPDH (6).tif]

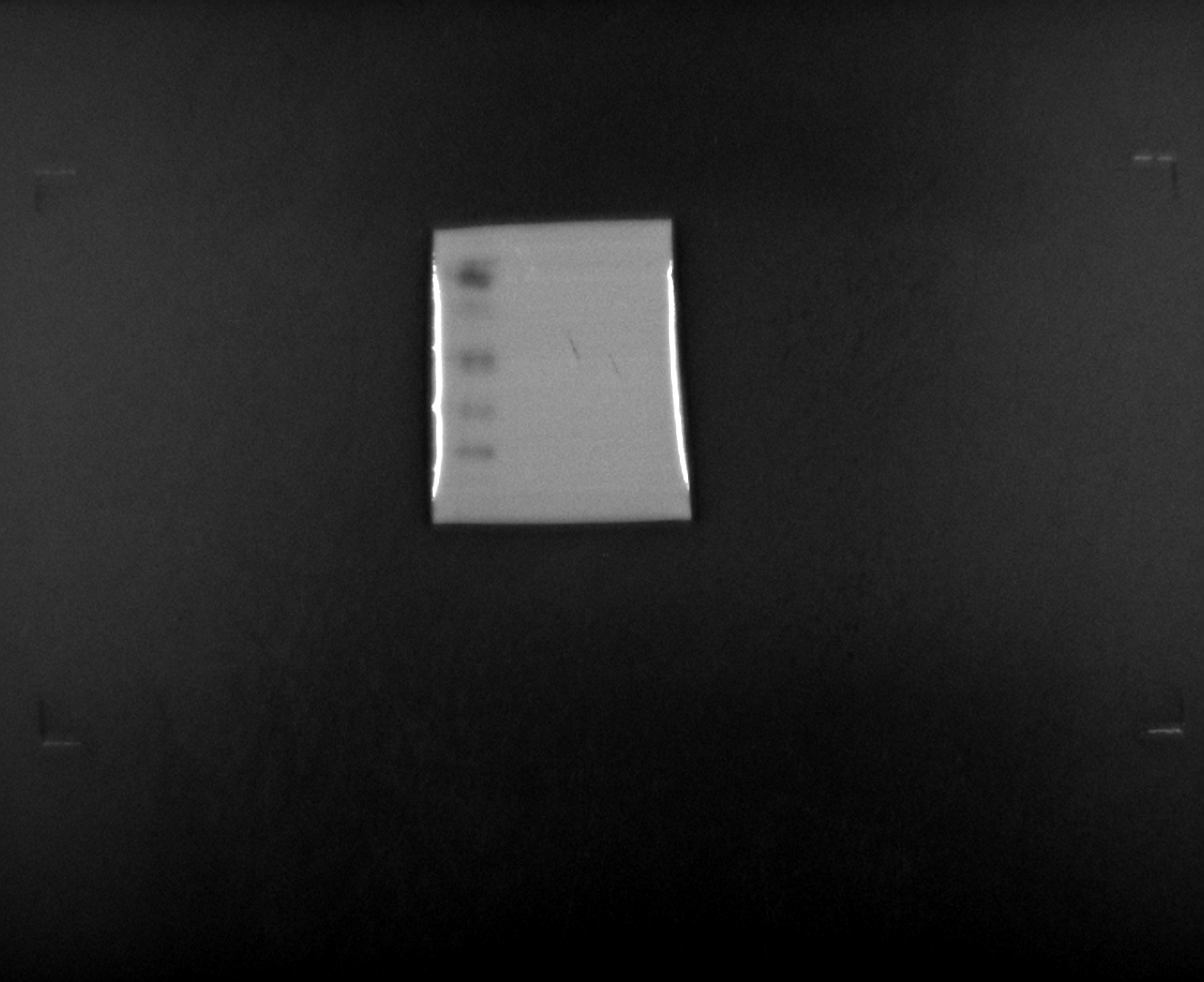

Supplement: Supplemental Information 6 [file peerj-12-17874-s006.zip › fig 4B/GAPDH-2 (4).tif]

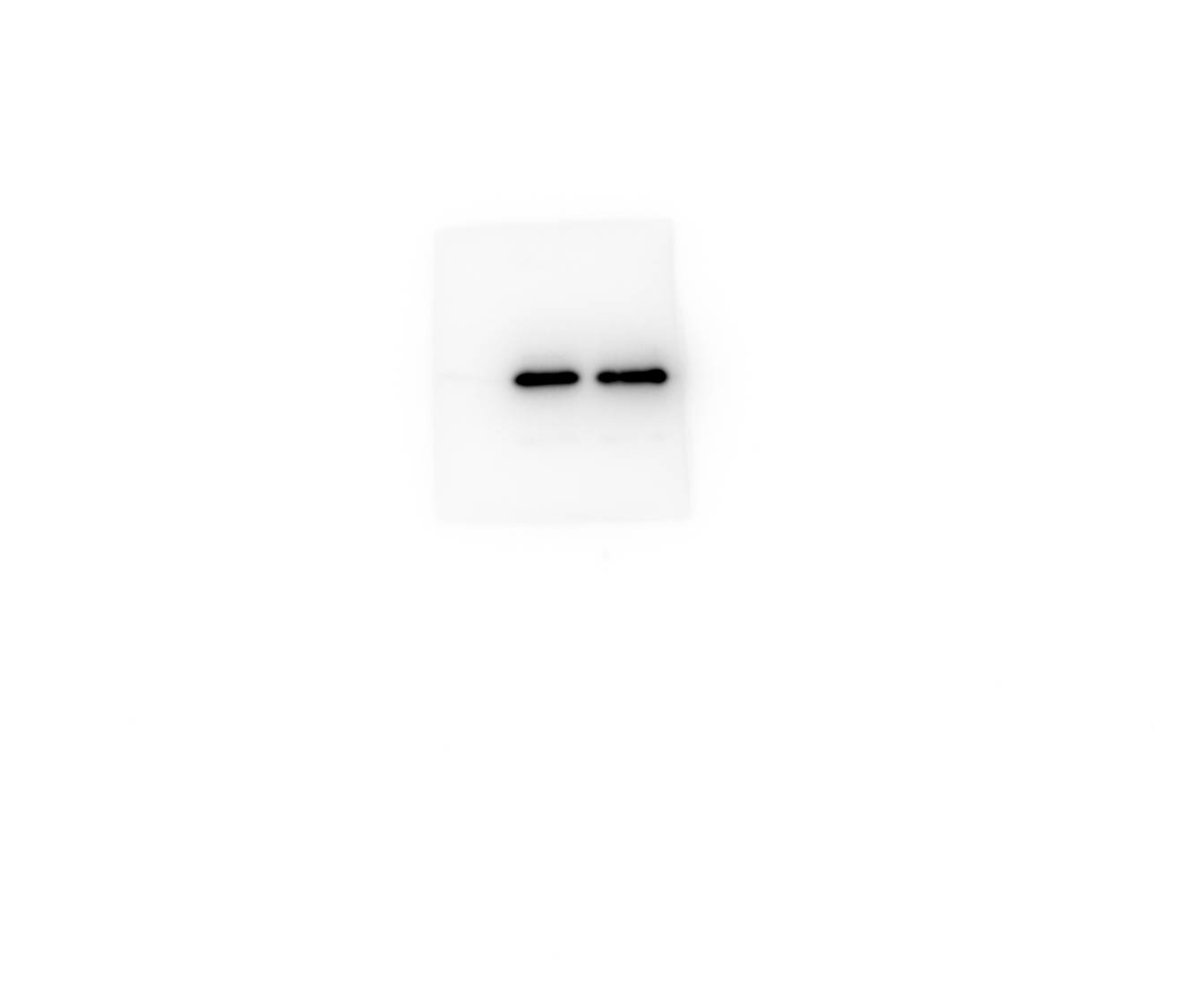

Supplement: Supplemental Information 6 [file peerj-12-17874-s006.zip › fig 4B/GAPDH-2 (5).tif]

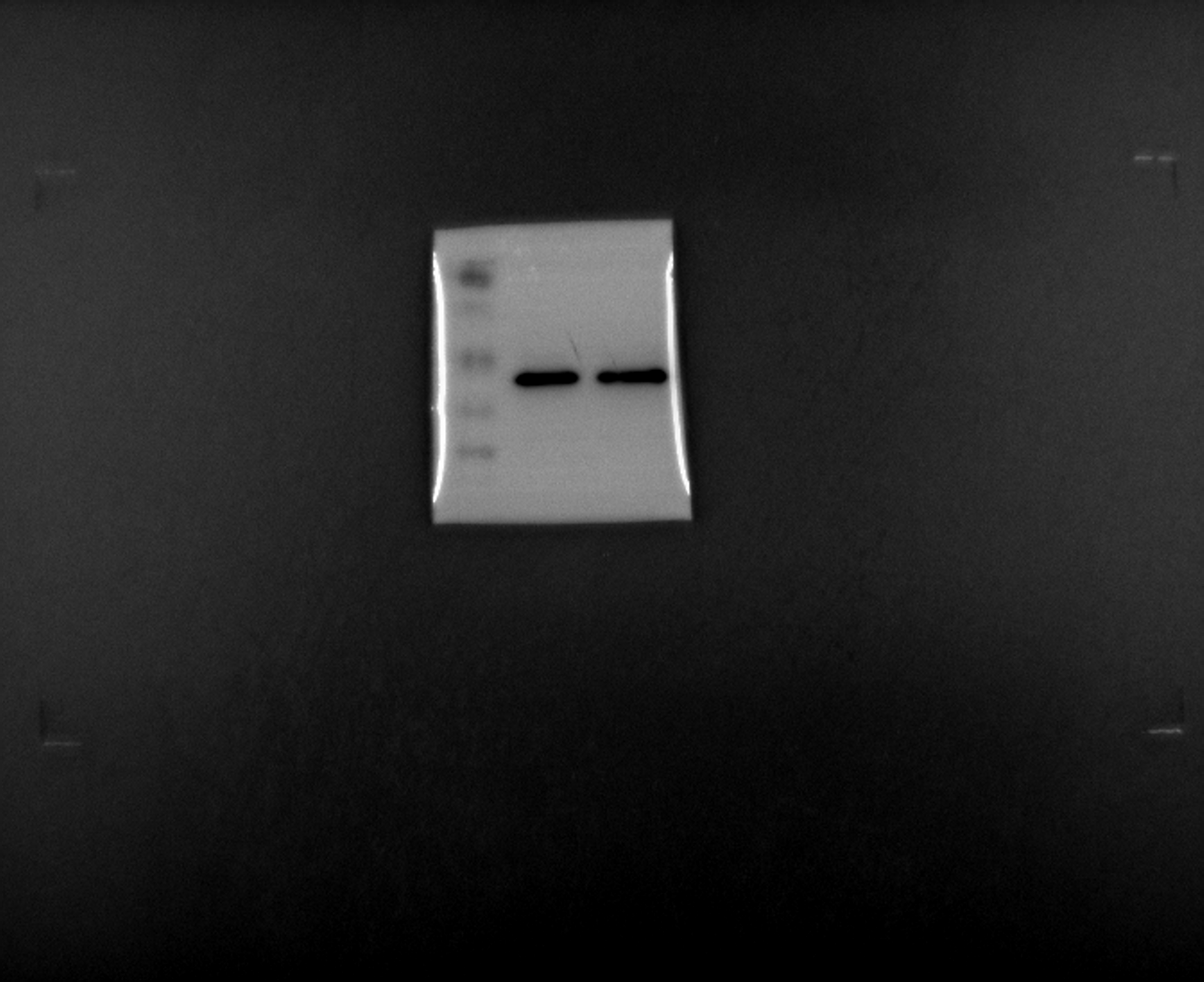

Supplement: Supplemental Information 6 [file peerj-12-17874-s006.zip › fig 4B/GAPDH-2 (6).tif]

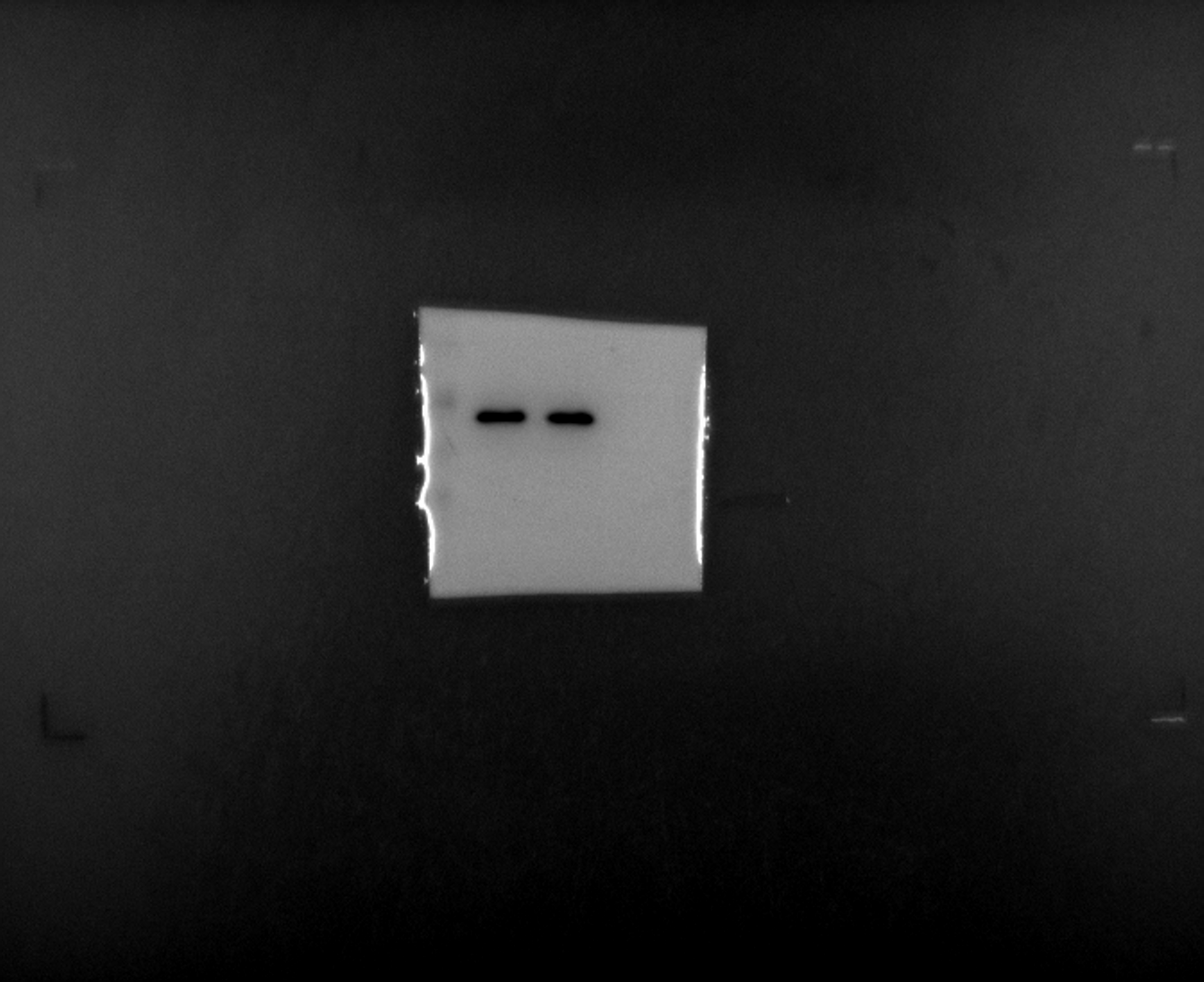

Supplement: Supplemental Information 6 [file peerj-12-17874-s006.zip › fig 4B/GAPDH-3 (4).tif]

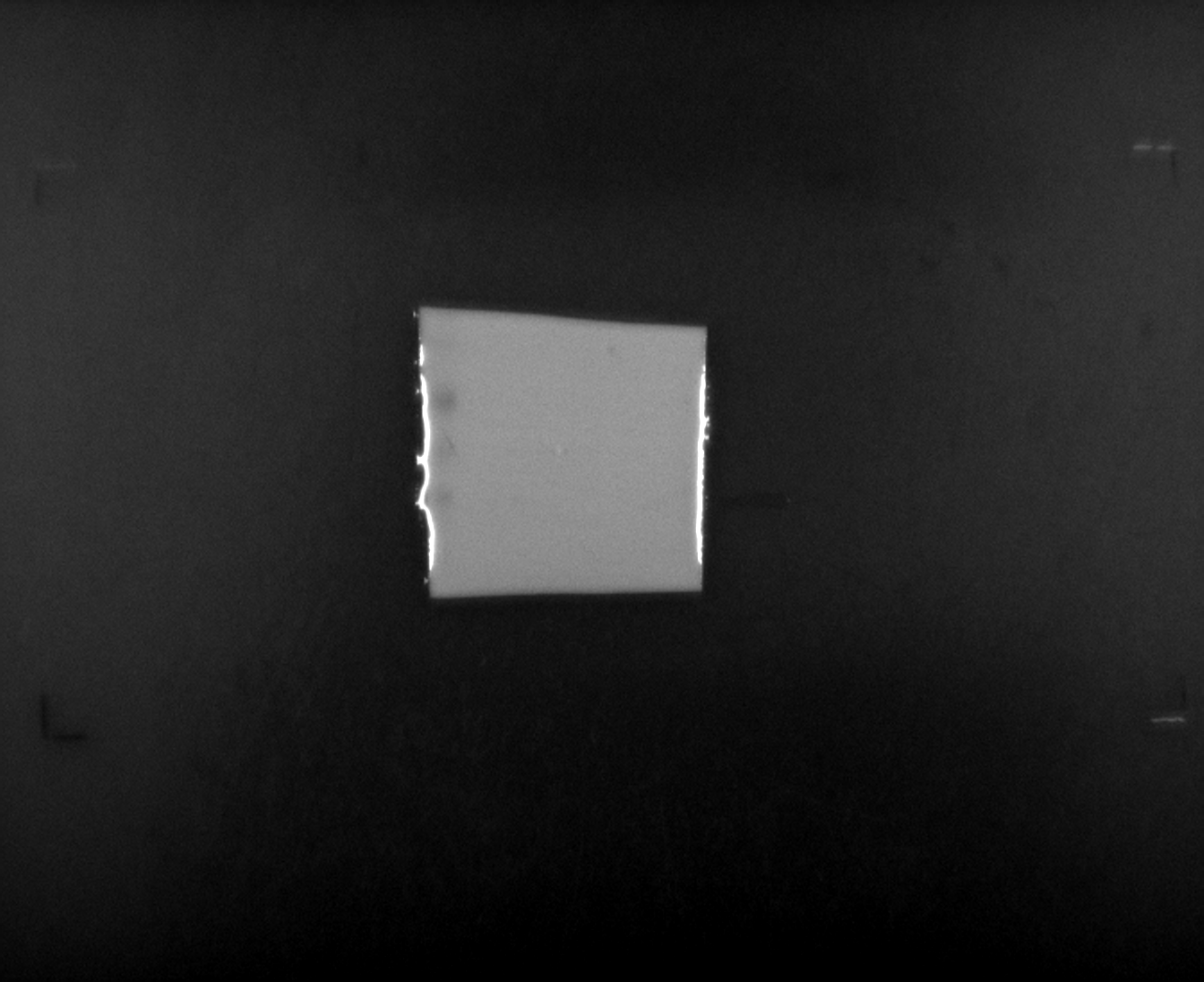

Supplement: Supplemental Information 6 [file peerj-12-17874-s006.zip › fig 4B/GAPDH-3 (5).tif]

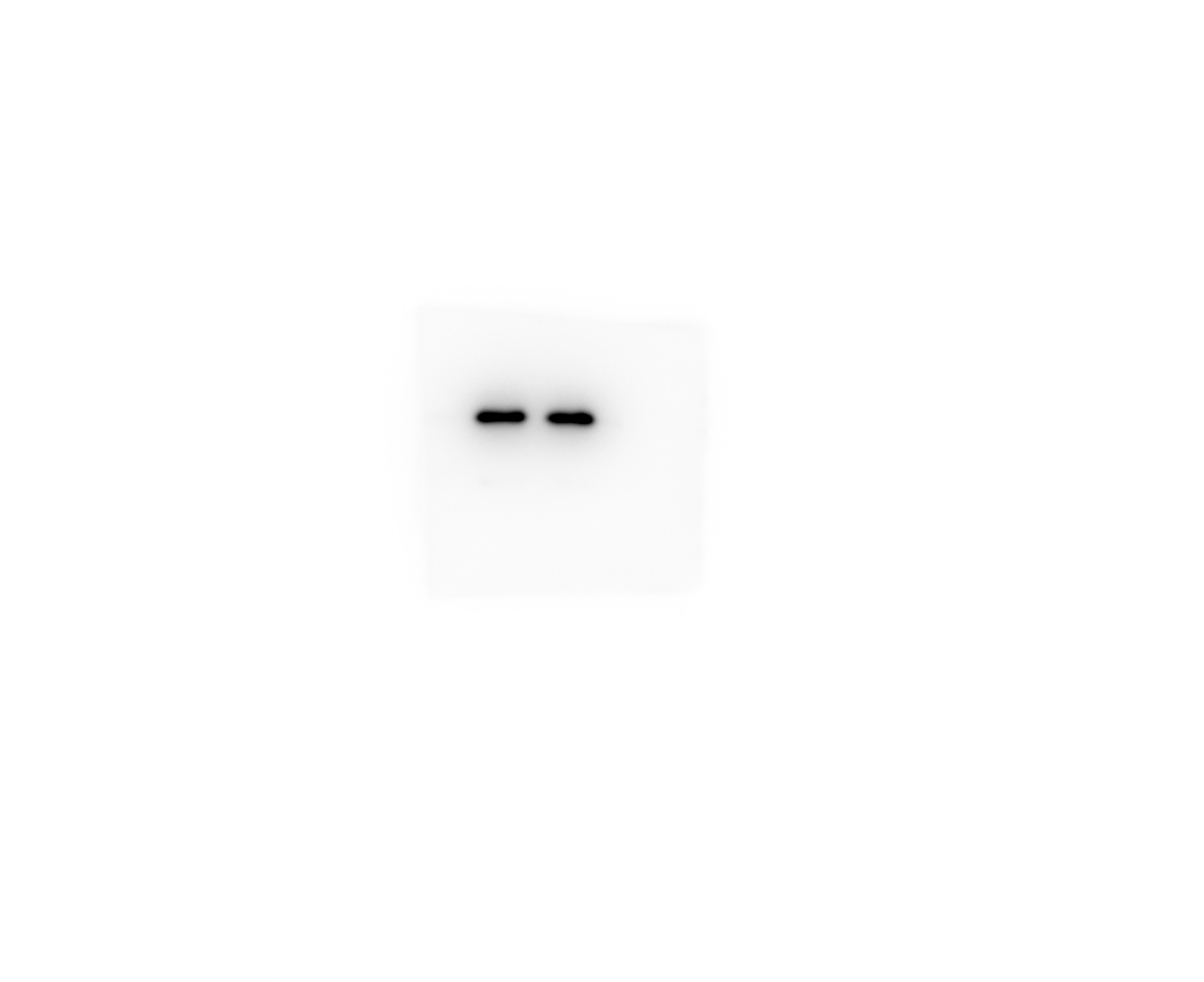

Supplement: Supplemental Information 6 [file peerj-12-17874-s006.zip › fig 4B/GAPDH-3 (6).tif]

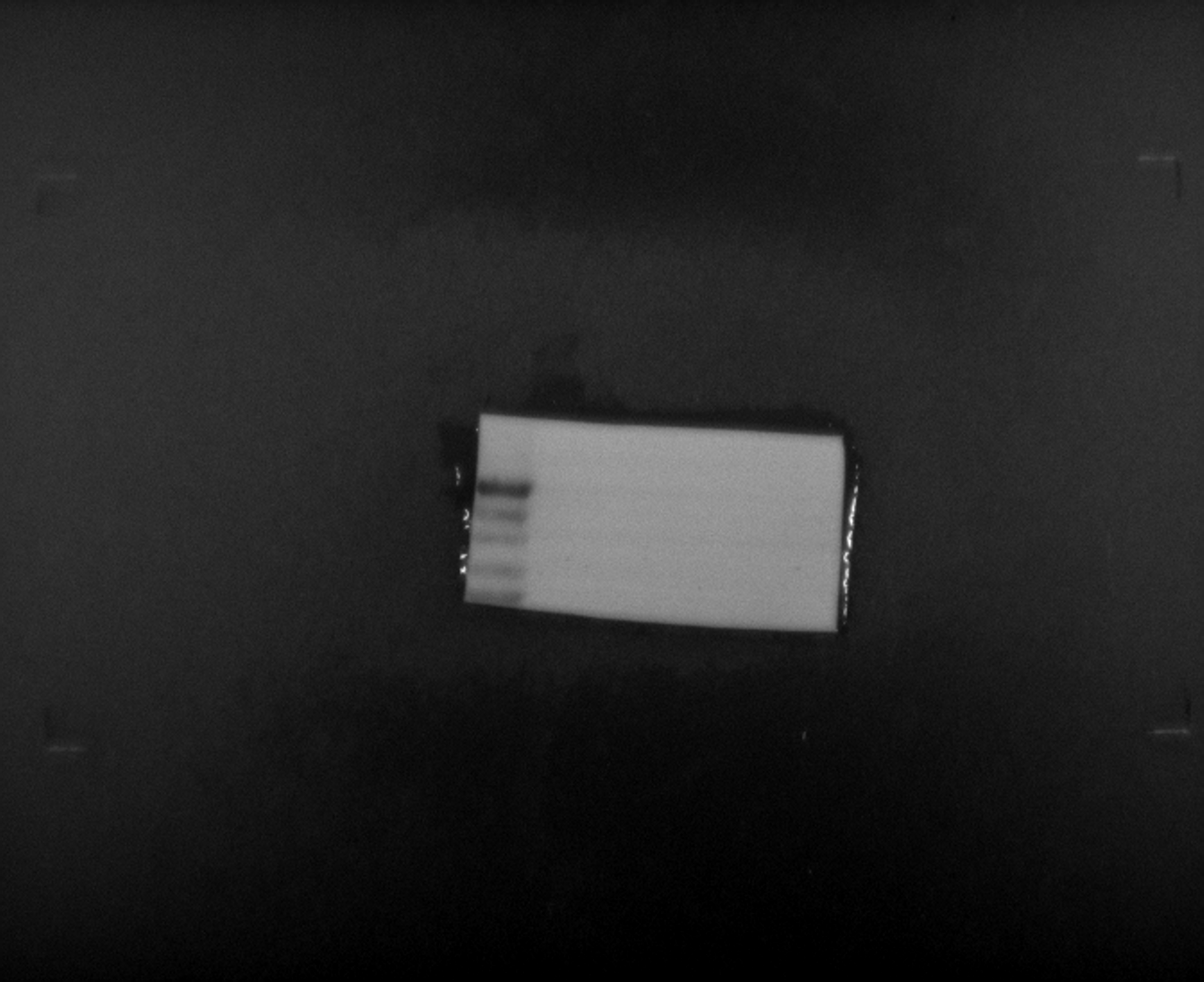

Supplement: Supplemental Information 7 [file peerj-12-17874-s007.zip › fig 4G/BAX (1).tif]

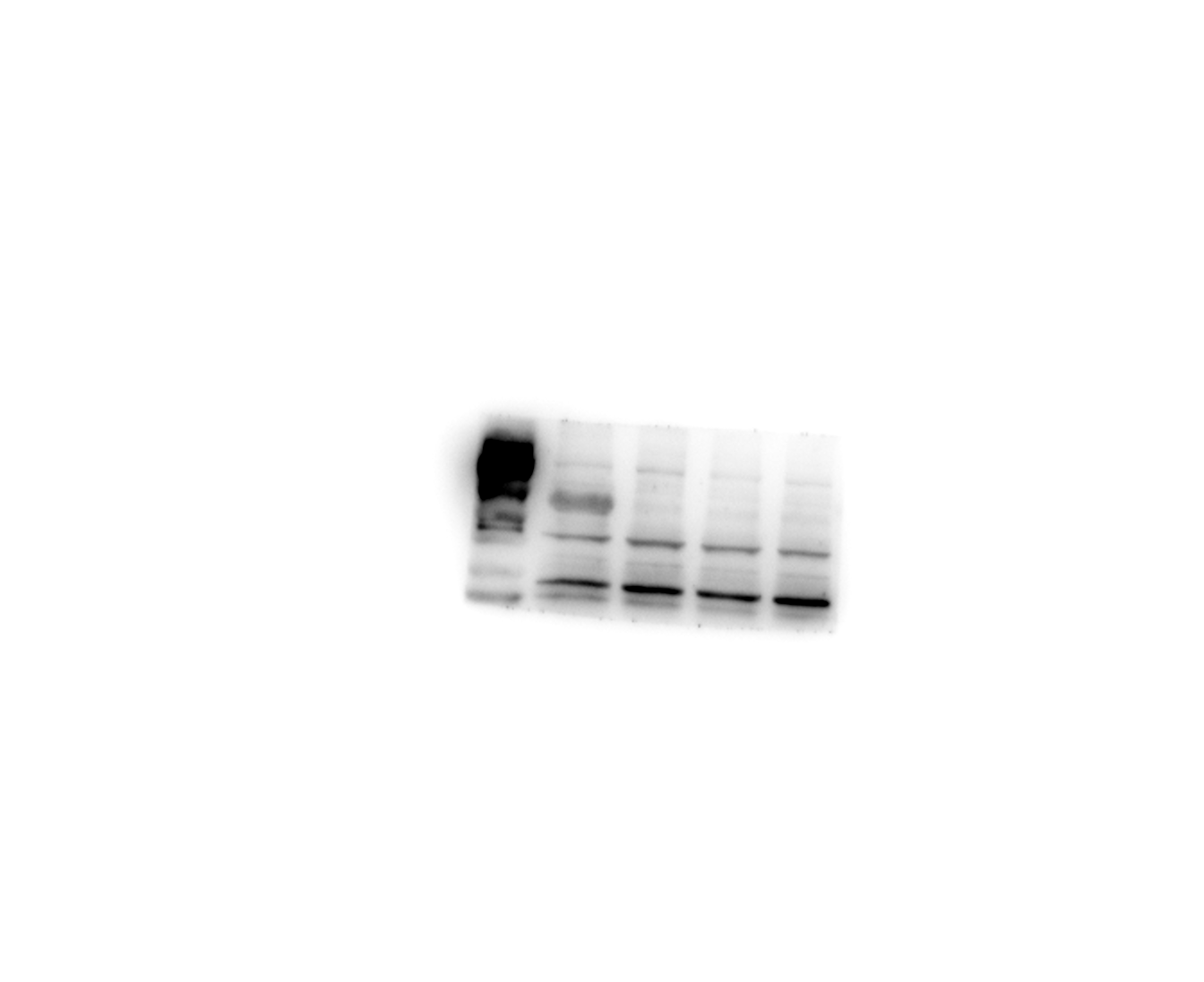

Supplement: Supplemental Information 7 [file peerj-12-17874-s007.zip › fig 4G/BAX (2).tif]

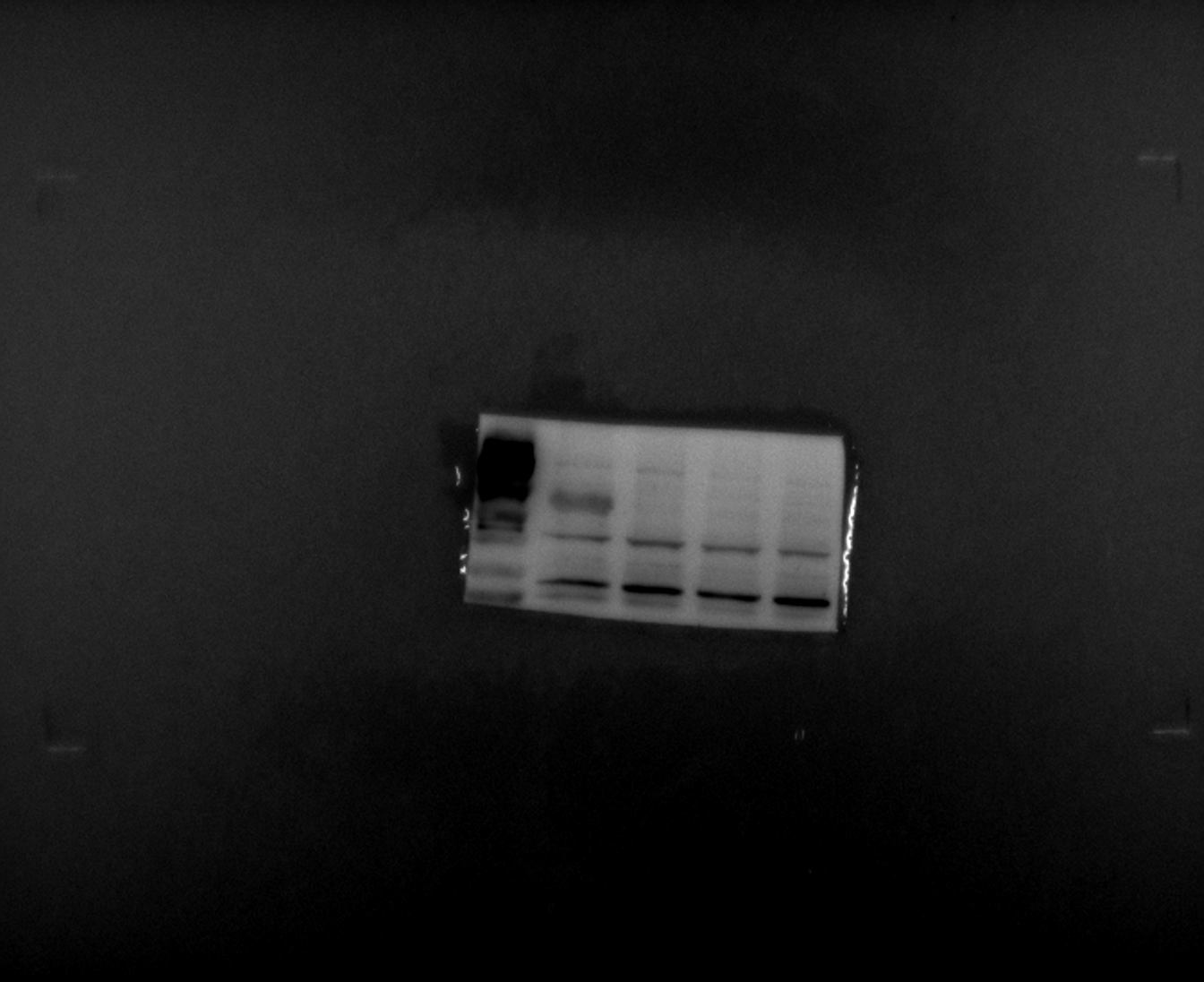

Supplement: Supplemental Information 7 [file peerj-12-17874-s007.zip › fig 4G/BAX (3).tif]

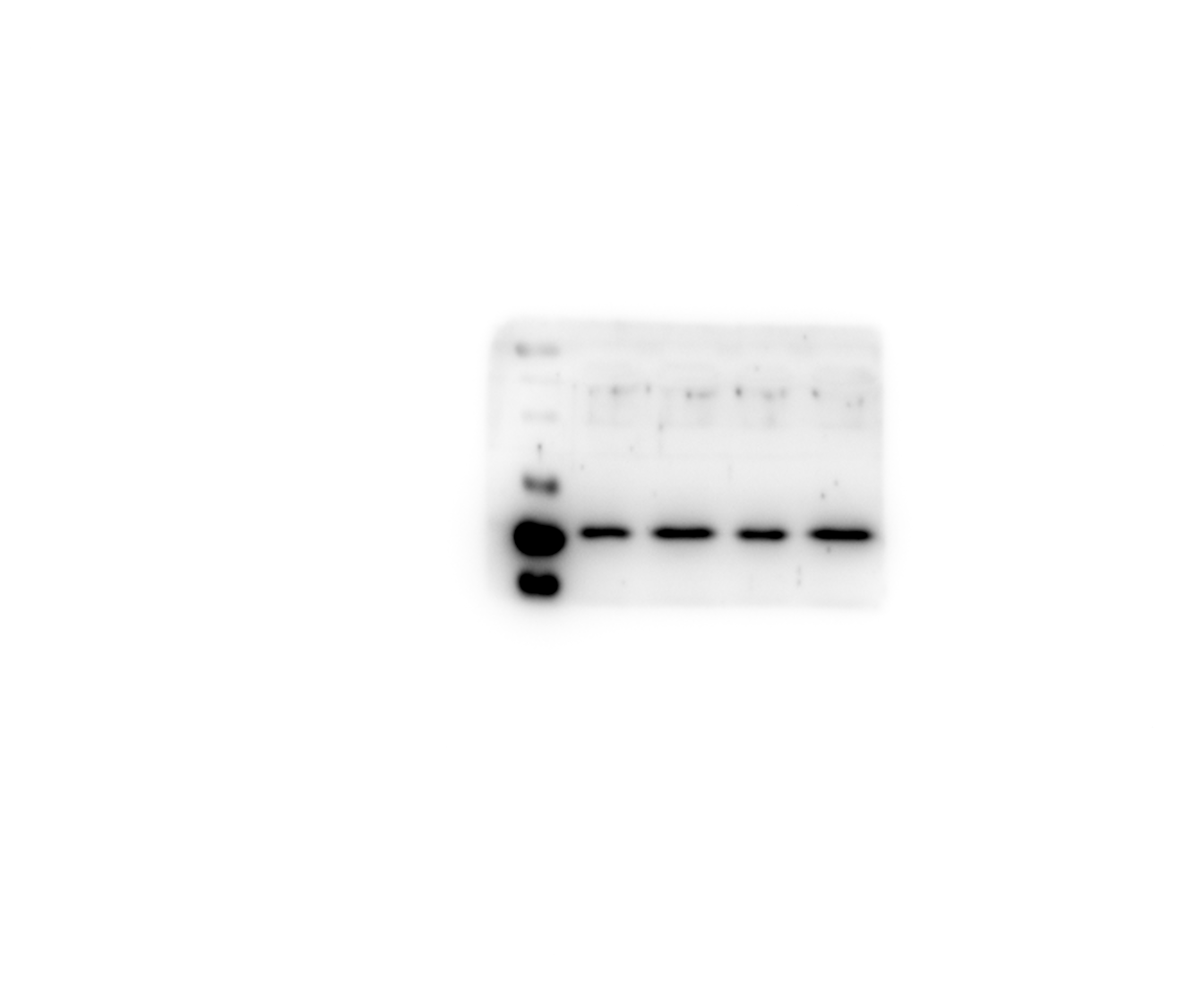

Supplement: Supplemental Information 7 [file peerj-12-17874-s007.zip › fig 4G/bax-2 (1).tif]

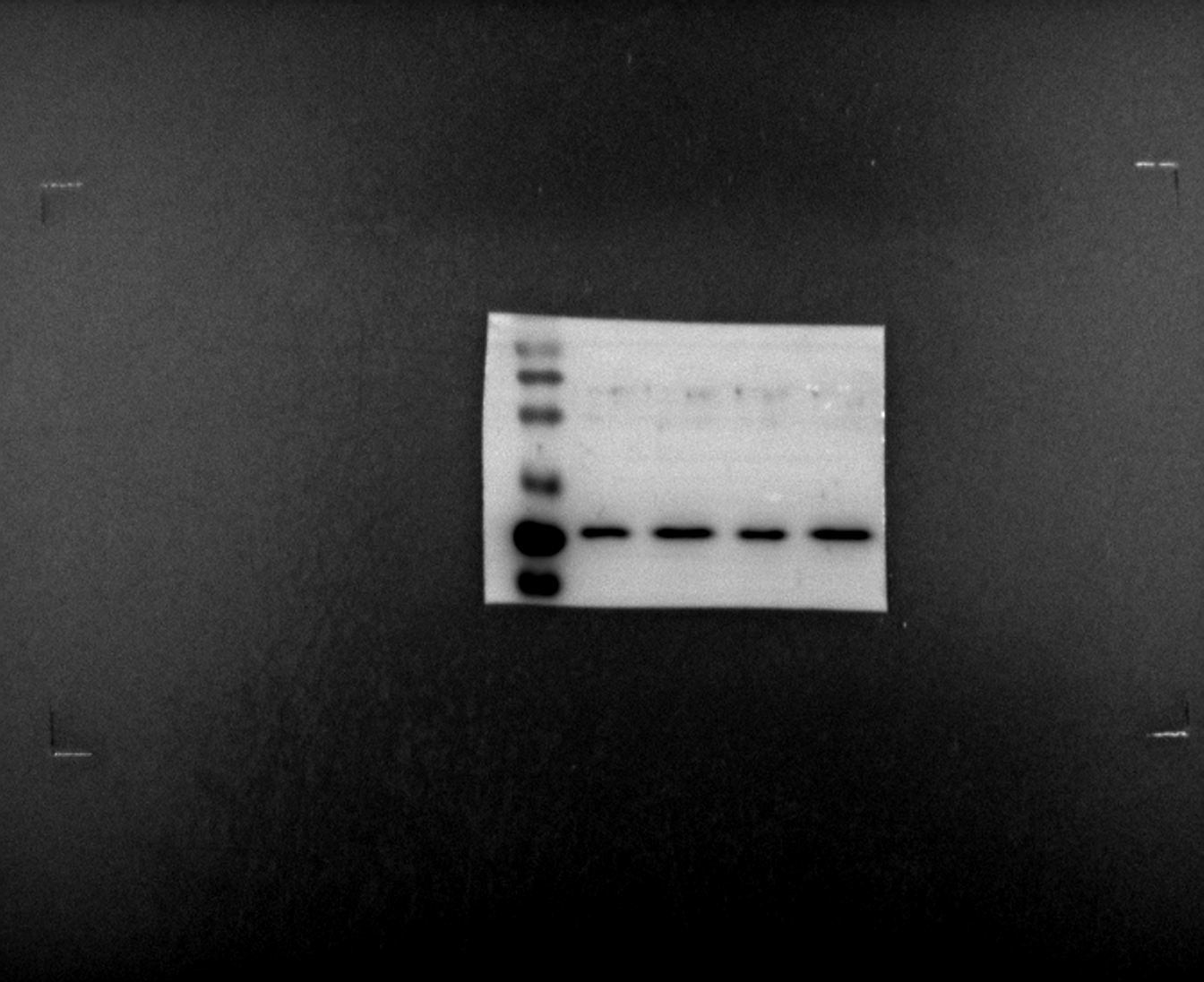

Supplement: Supplemental Information 7 [file peerj-12-17874-s007.zip › fig 4G/bax-2 (2).tif]

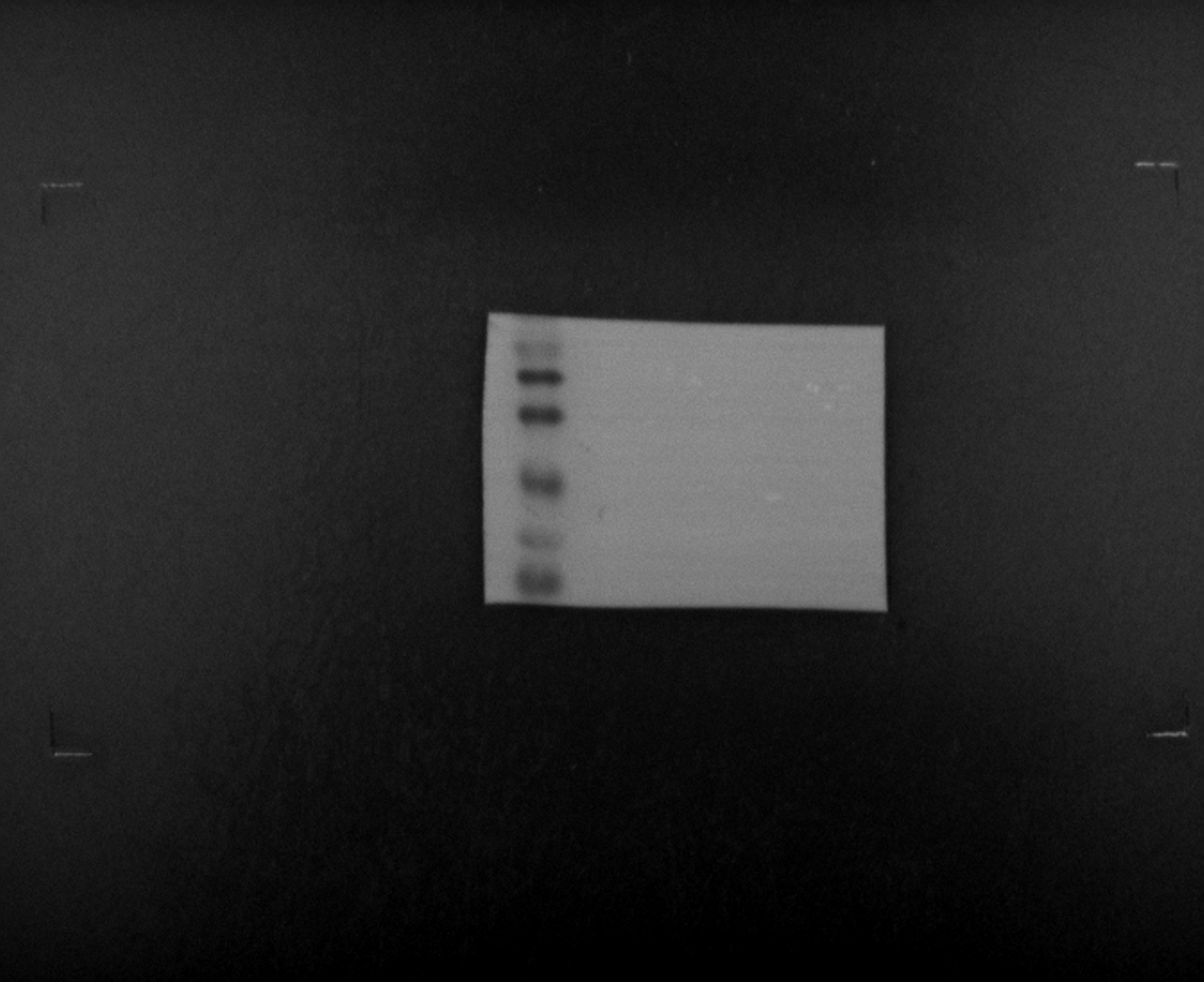

Supplement: Supplemental Information 7 [file peerj-12-17874-s007.zip › fig 4G/bax-2 (3).tif]

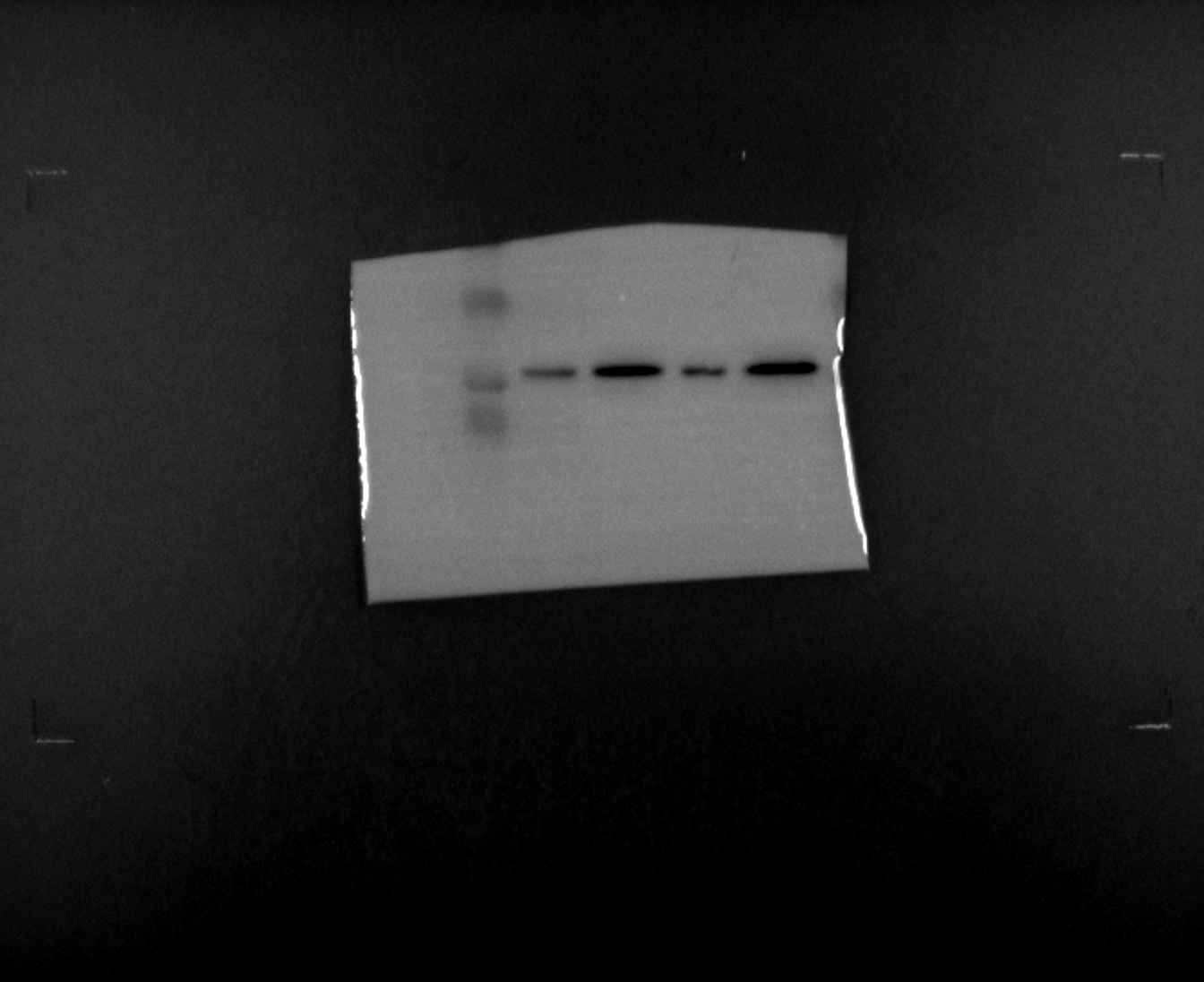

Supplement: Supplemental Information 7 [file peerj-12-17874-s007.zip › fig 4G/bax-3 (1).tif]

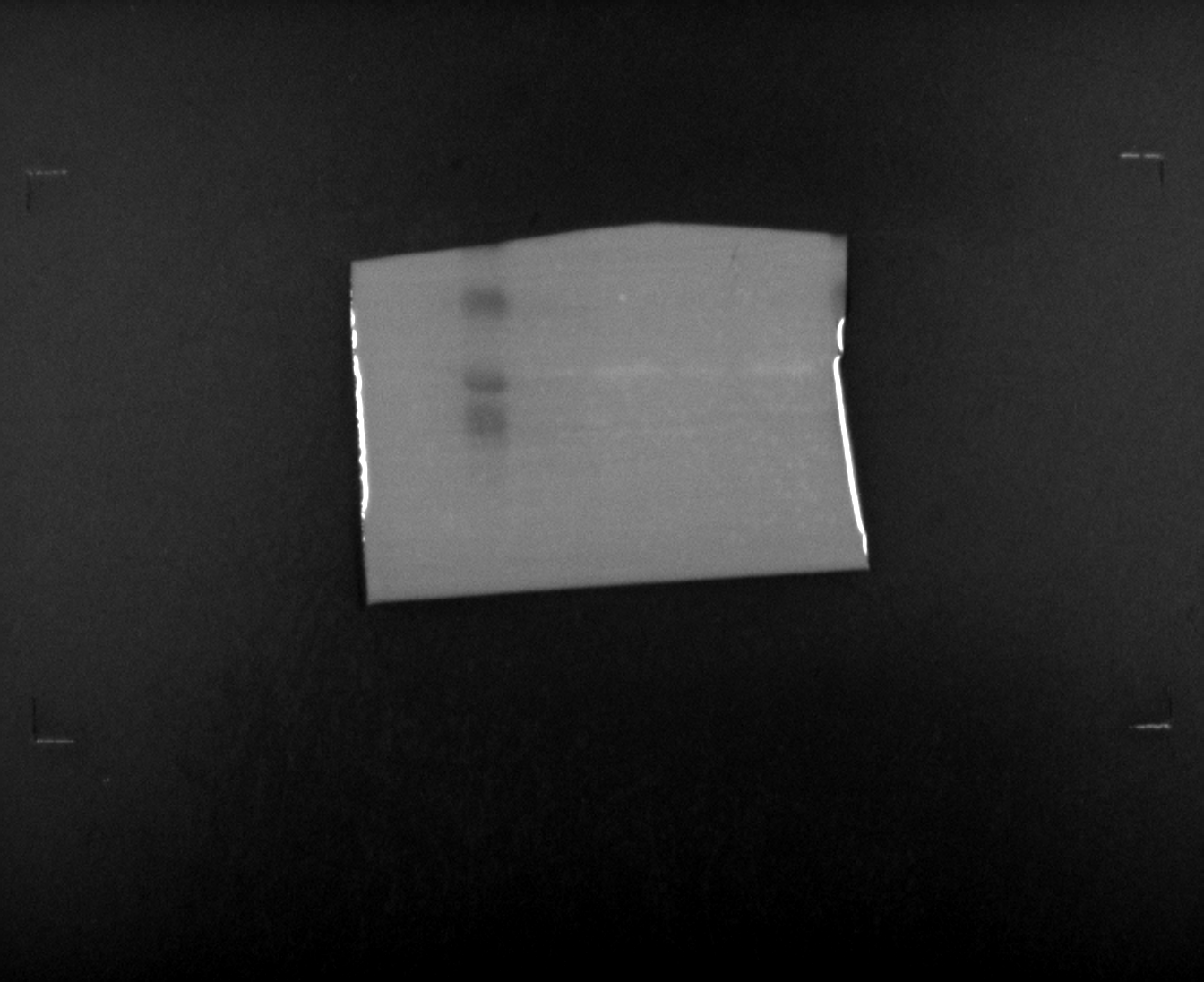

Supplement: Supplemental Information 7 [file peerj-12-17874-s007.zip › fig 4G/bax-3 (2).tif]

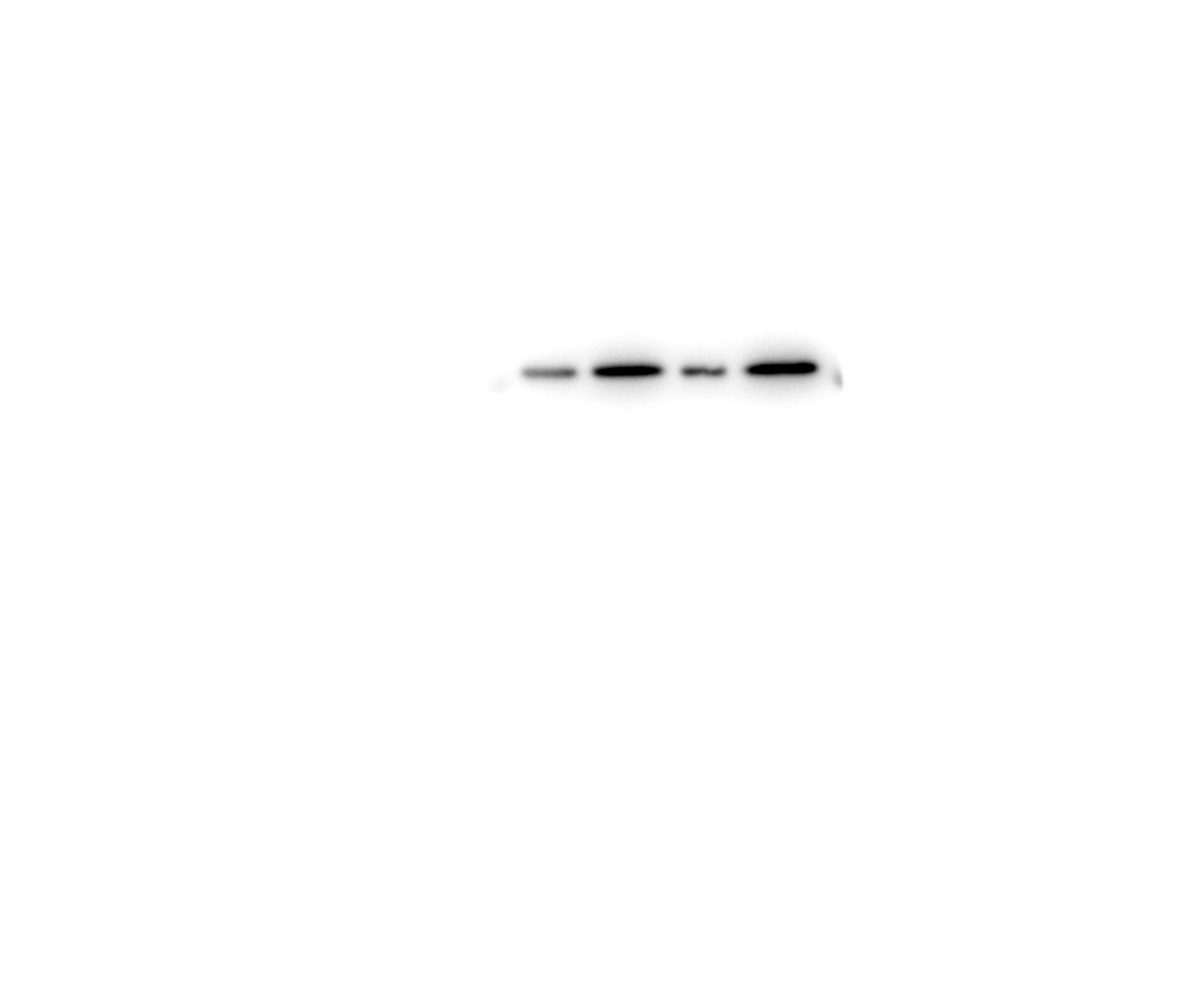

Supplement: Supplemental Information 7 [file peerj-12-17874-s007.zip › fig 4G/bax-3 (3).tif]

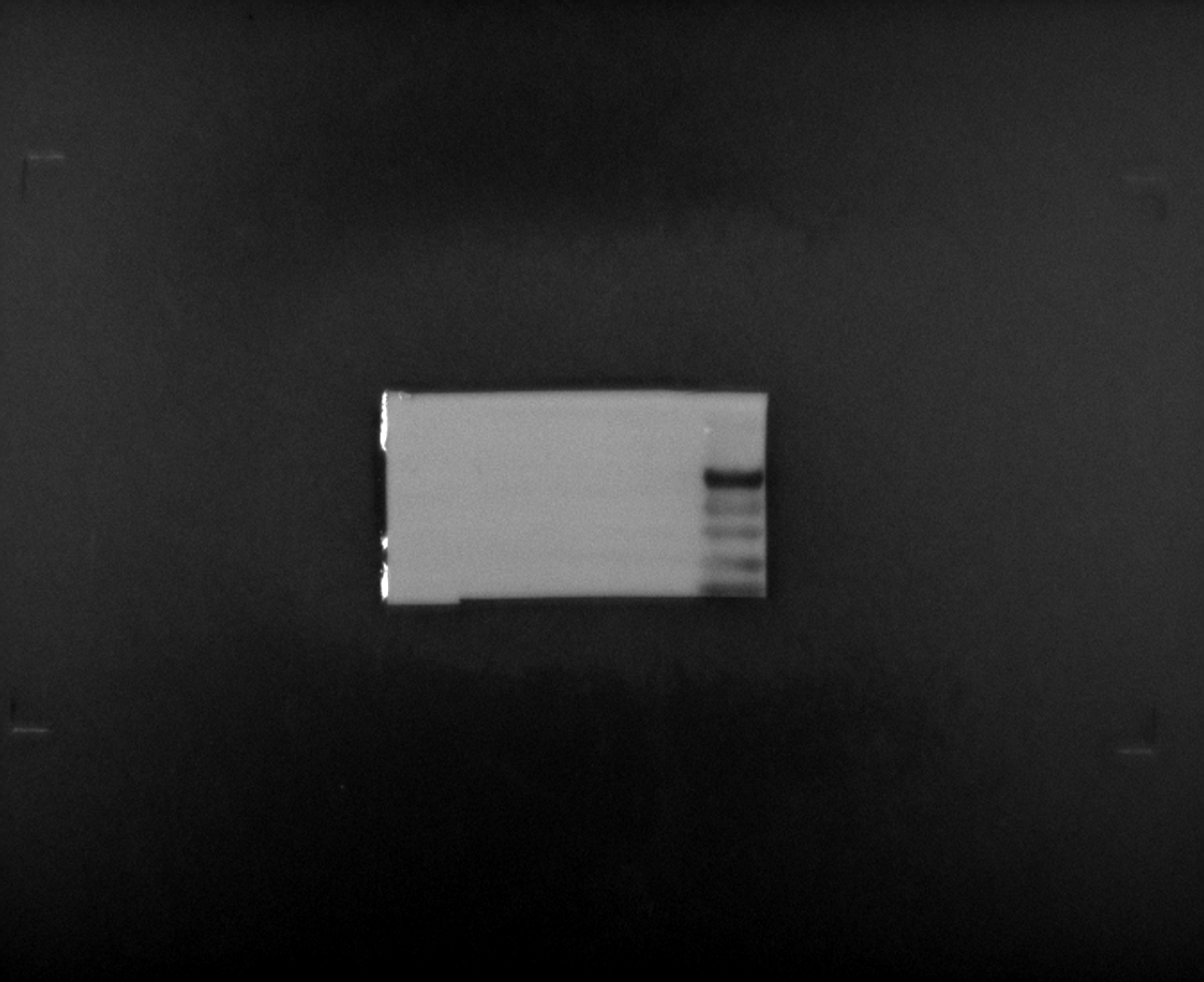

Supplement: Supplemental Information 7 [file peerj-12-17874-s007.zip › fig 4G/BCL2 (1).tif]

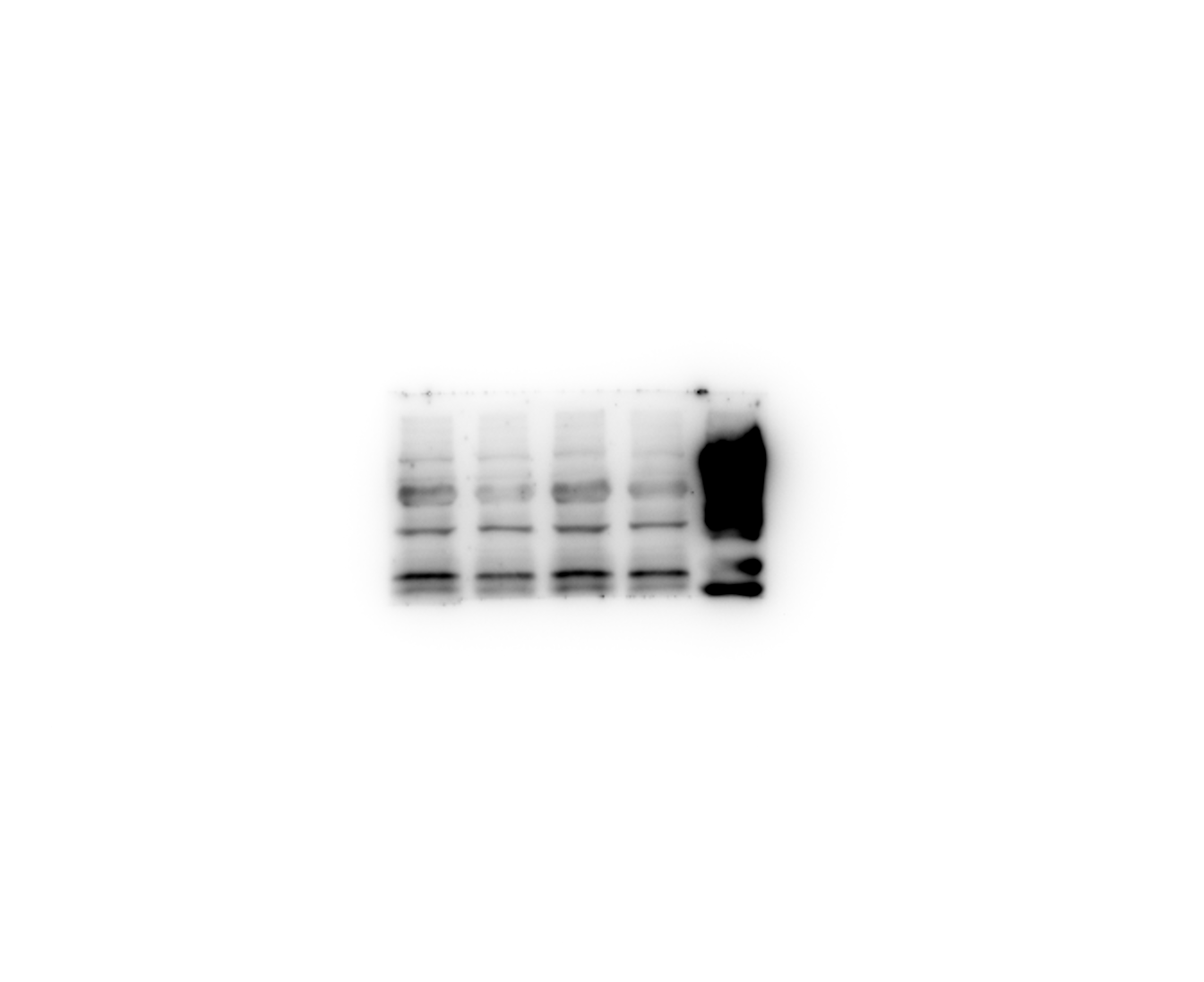

Supplement: Supplemental Information 7 [file peerj-12-17874-s007.zip › fig 4G/BCL2 (2).tif]

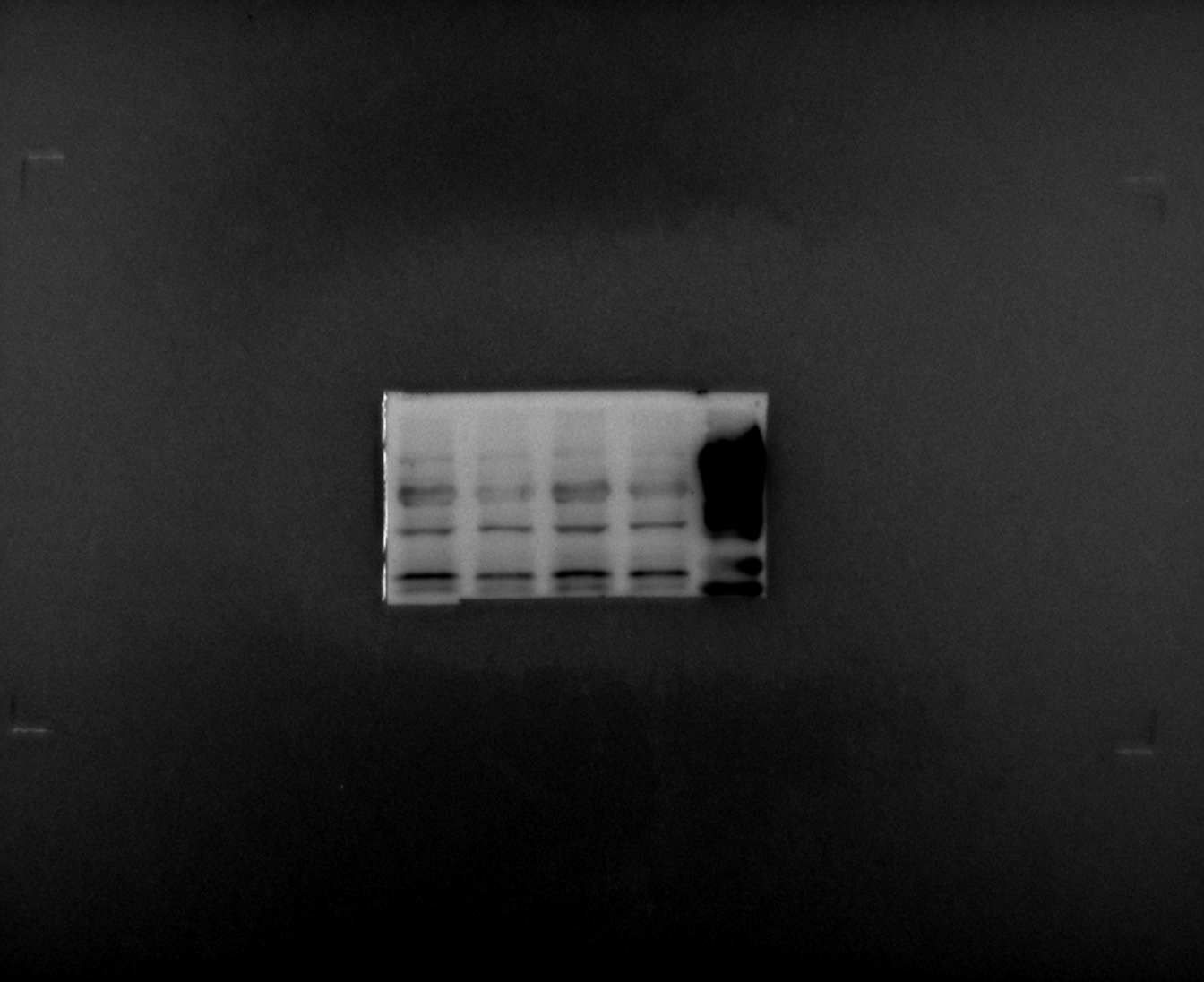

Supplement: Supplemental Information 7 [file peerj-12-17874-s007.zip › fig 4G/BCL2 (3).tif]

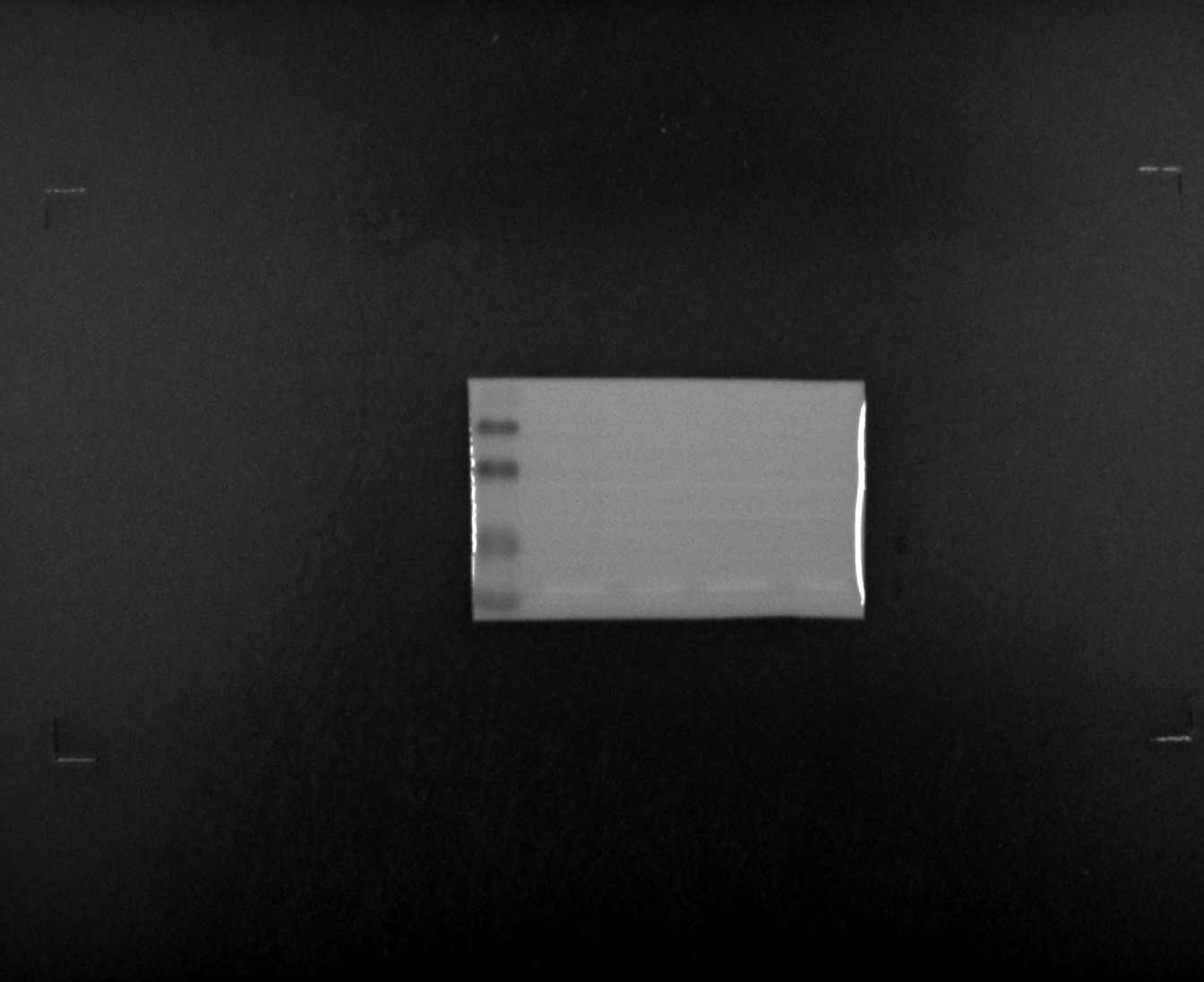

Supplement: Supplemental Information 7 [file peerj-12-17874-s007.zip › fig 4G/bcl2-2 (1).tif]

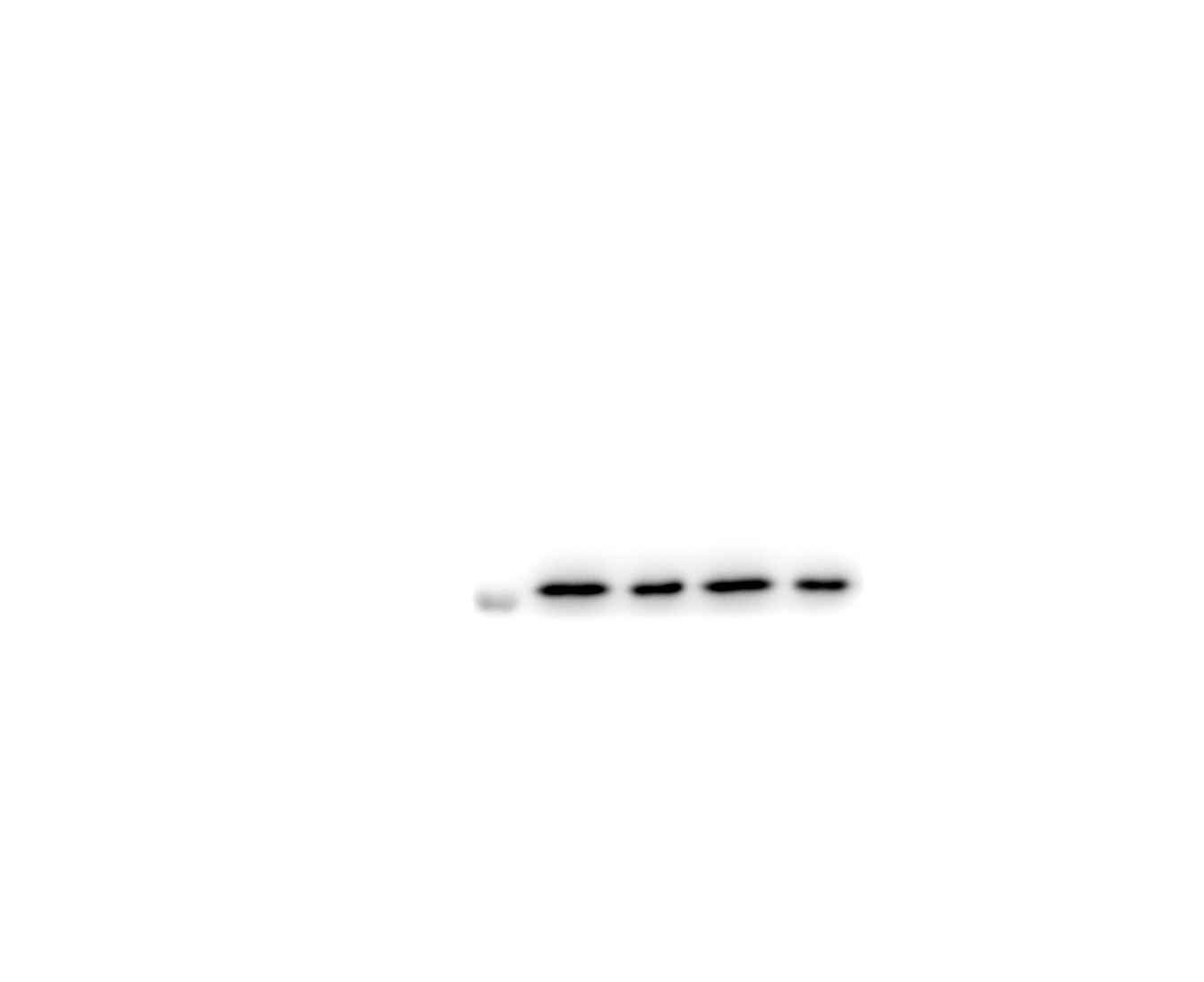

Supplement: Supplemental Information 7 [file peerj-12-17874-s007.zip › fig 4G/bcl2-2 (2).tif]

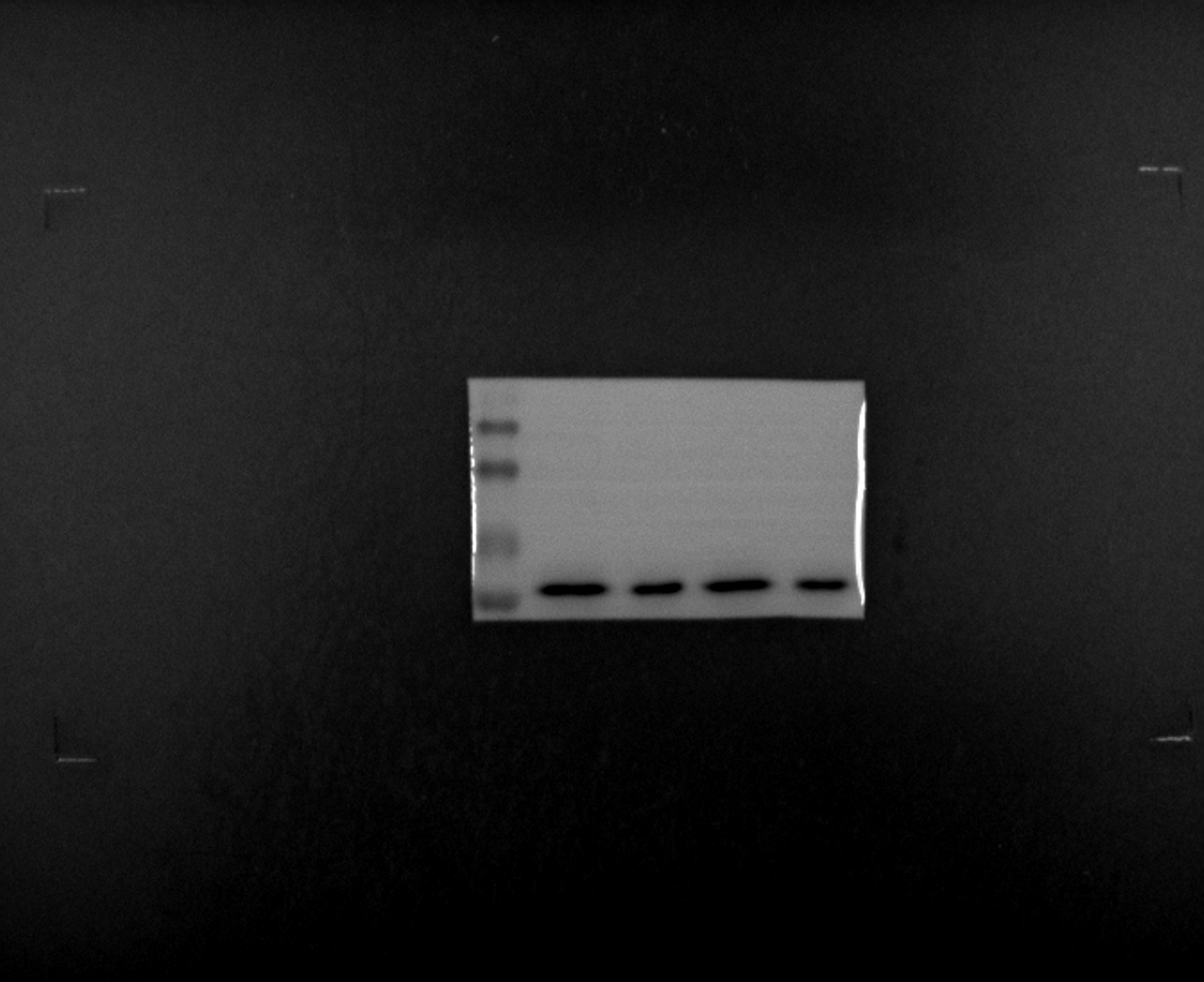

Supplement: Supplemental Information 7 [file peerj-12-17874-s007.zip › fig 4G/bcl2-2 (3).tif]

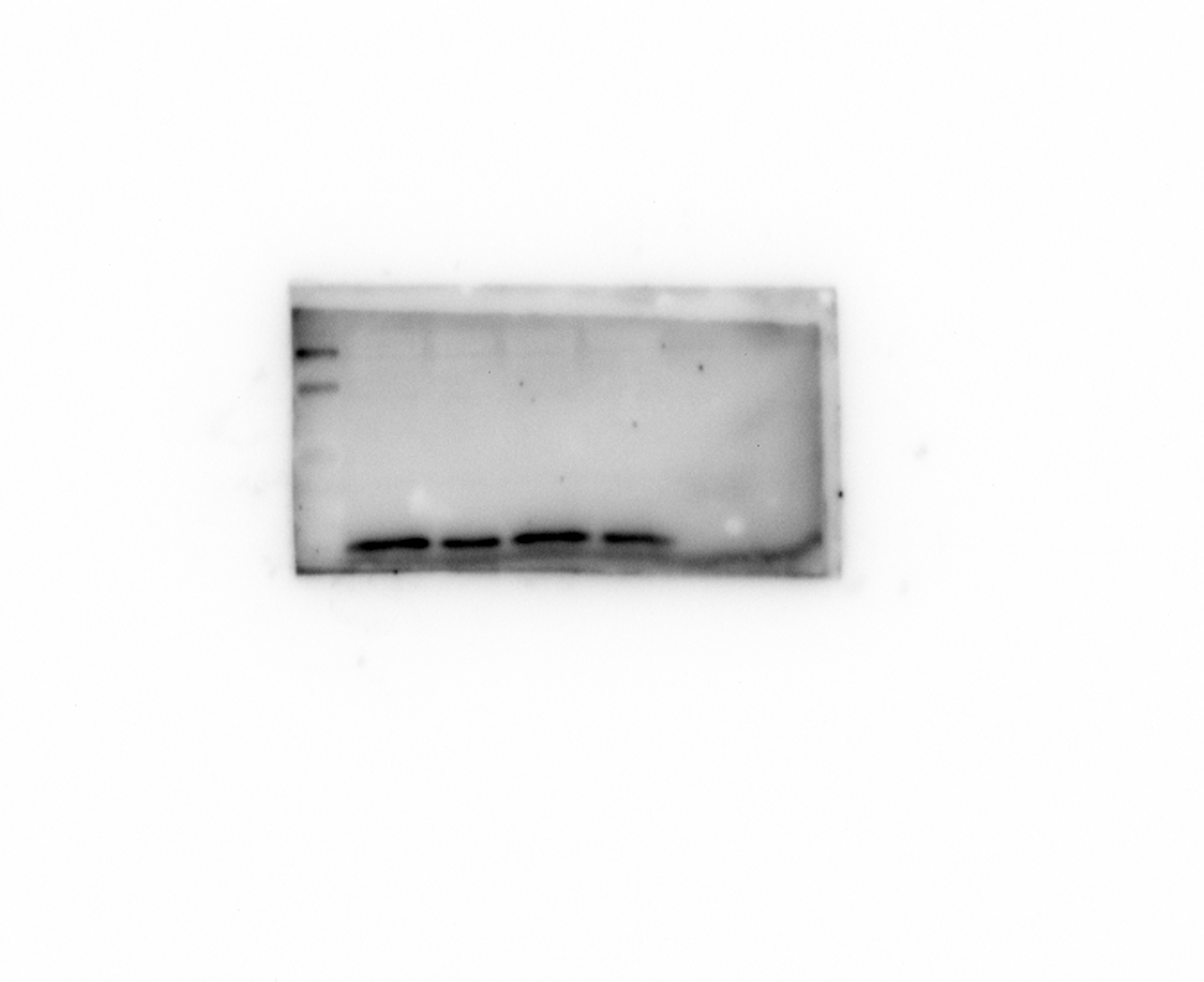

Supplement: Supplemental Information 7 [file peerj-12-17874-s007.zip › fig 4G/bcl2-3 (1).tif]

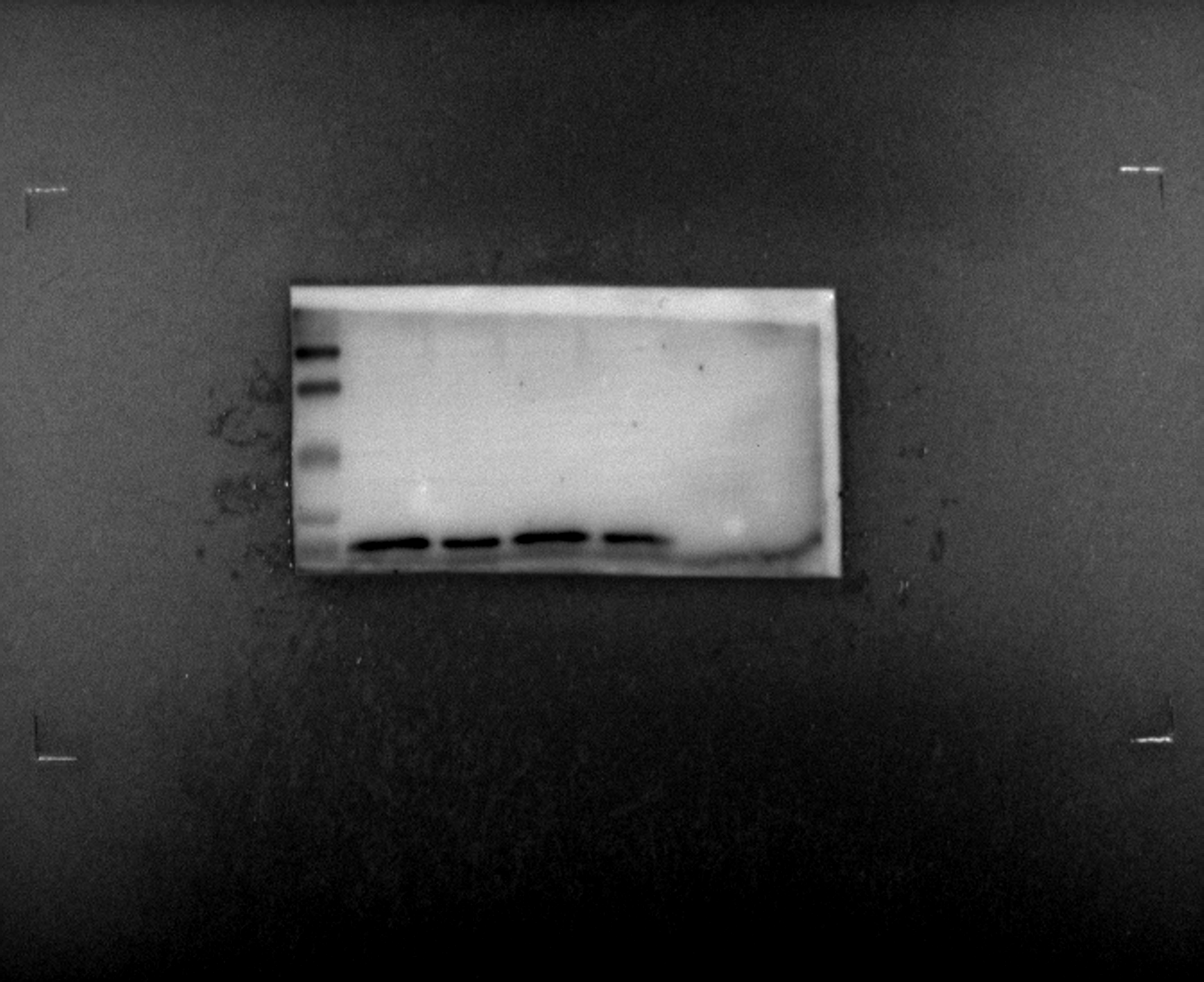

Supplement: Supplemental Information 7 [file peerj-12-17874-s007.zip › fig 4G/bcl2-3 (2).tif]

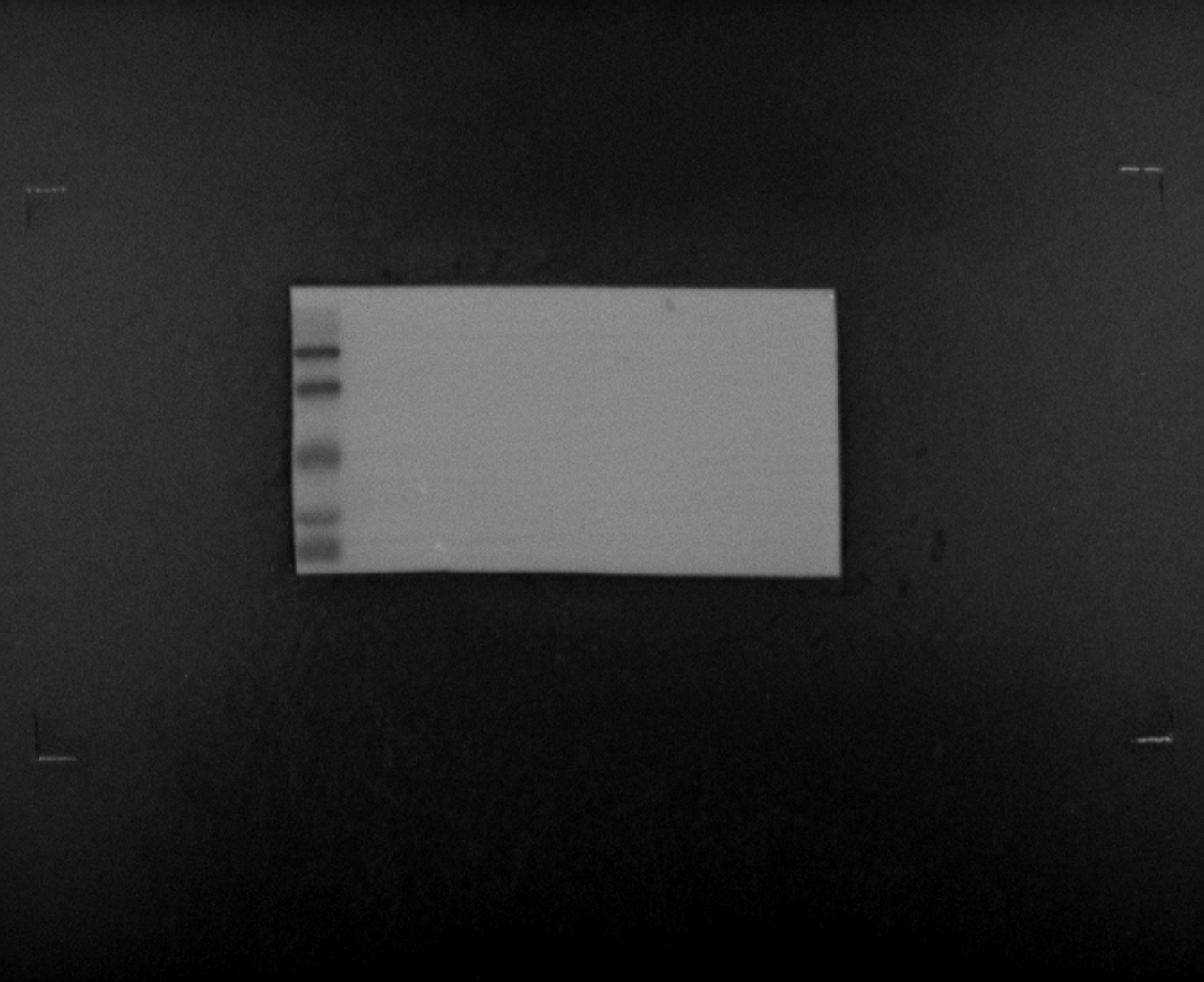

Supplement: Supplemental Information 7 [file peerj-12-17874-s007.zip › fig 4G/bcl2-3 (3).tif]

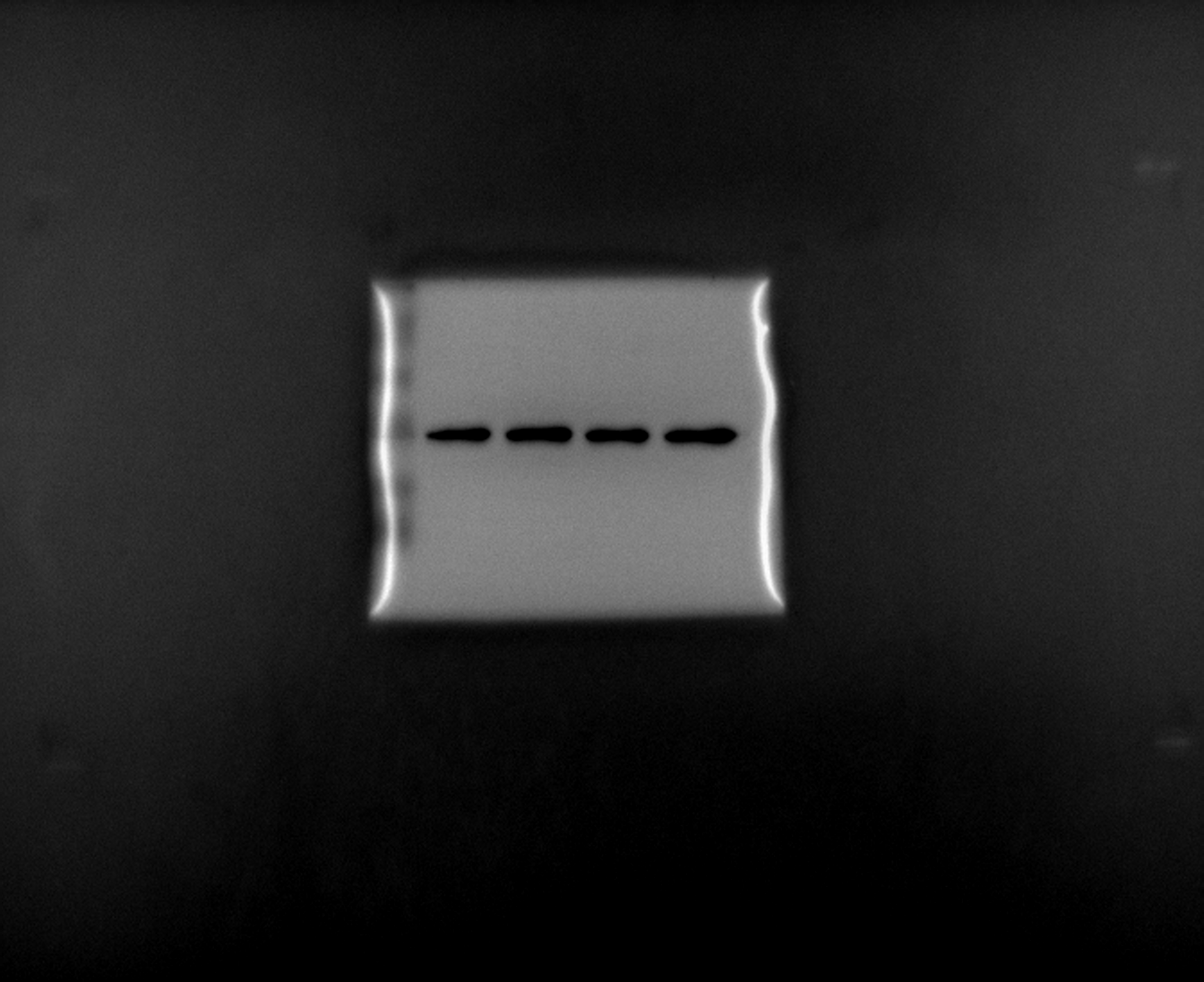

Supplement: Supplemental Information 7 [file peerj-12-17874-s007.zip › fig 4G/caspase1-2 (1).tif]

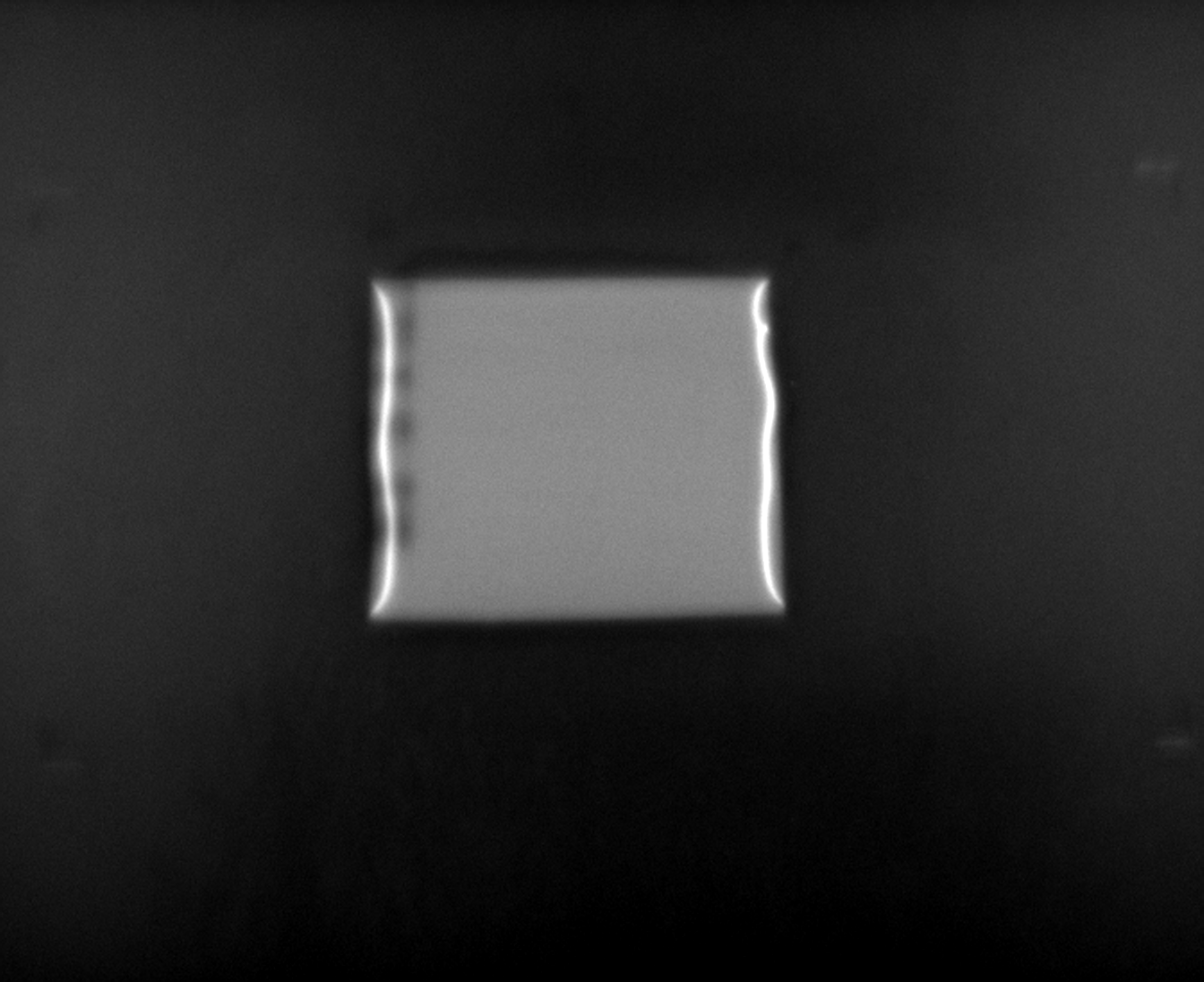

Supplement: Supplemental Information 7 [file peerj-12-17874-s007.zip › fig 4G/caspase1-2 (2).tif]

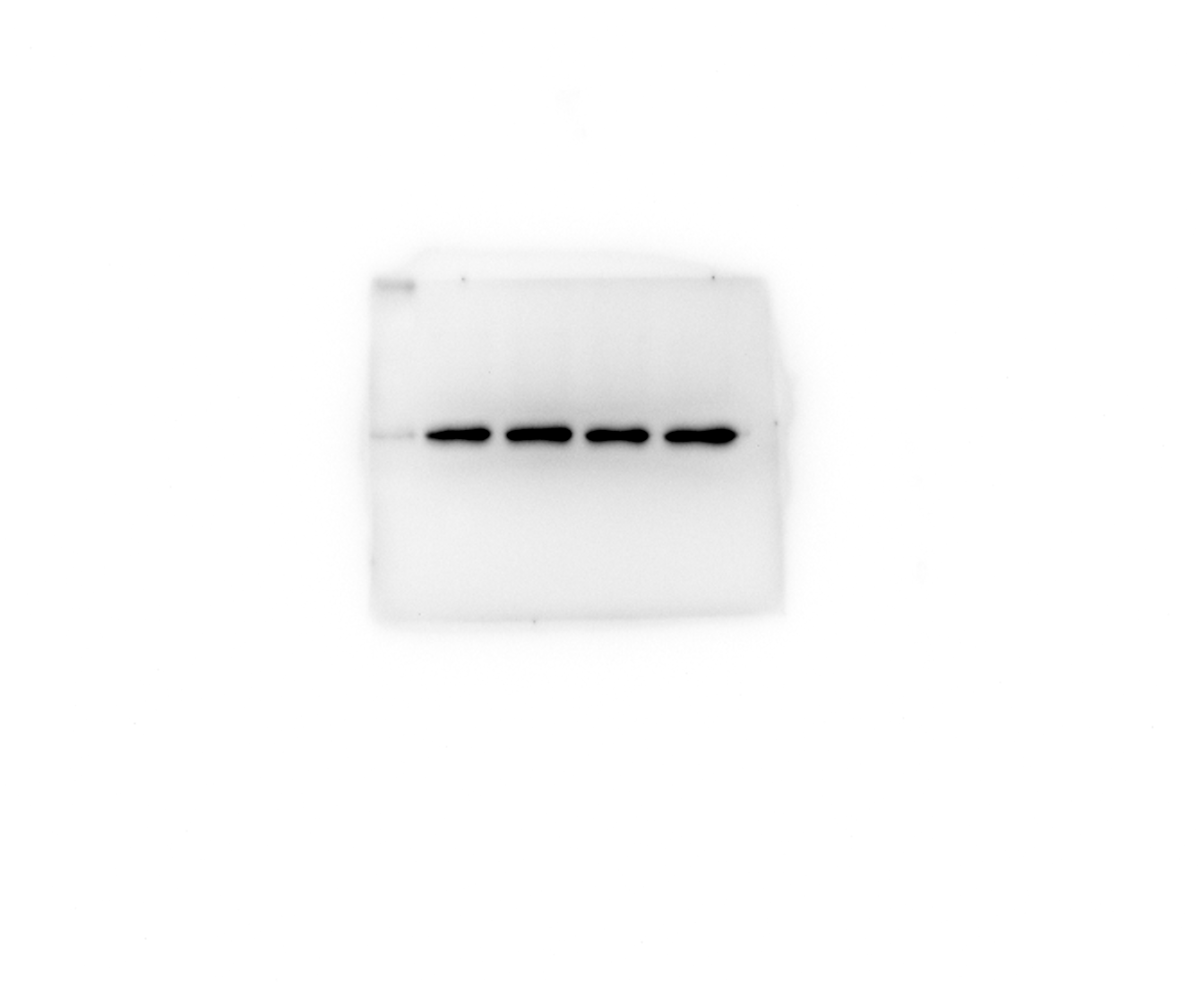

Supplement: Supplemental Information 7 [file peerj-12-17874-s007.zip › fig 4G/caspase1-2 (3).tif]

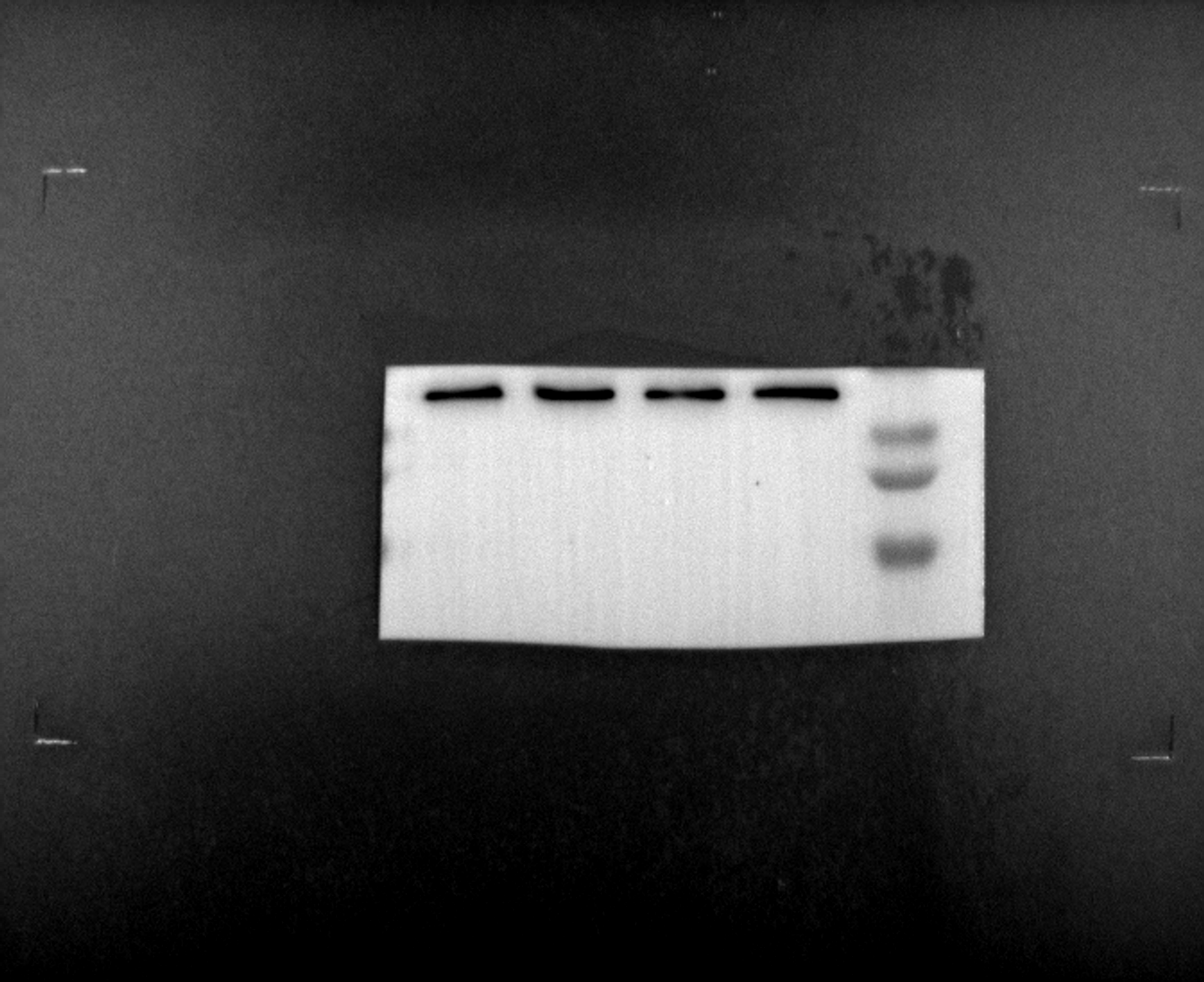

Supplement: Supplemental Information 7 [file peerj-12-17874-s007.zip › fig 4G/caspase1-3 (1).tif]

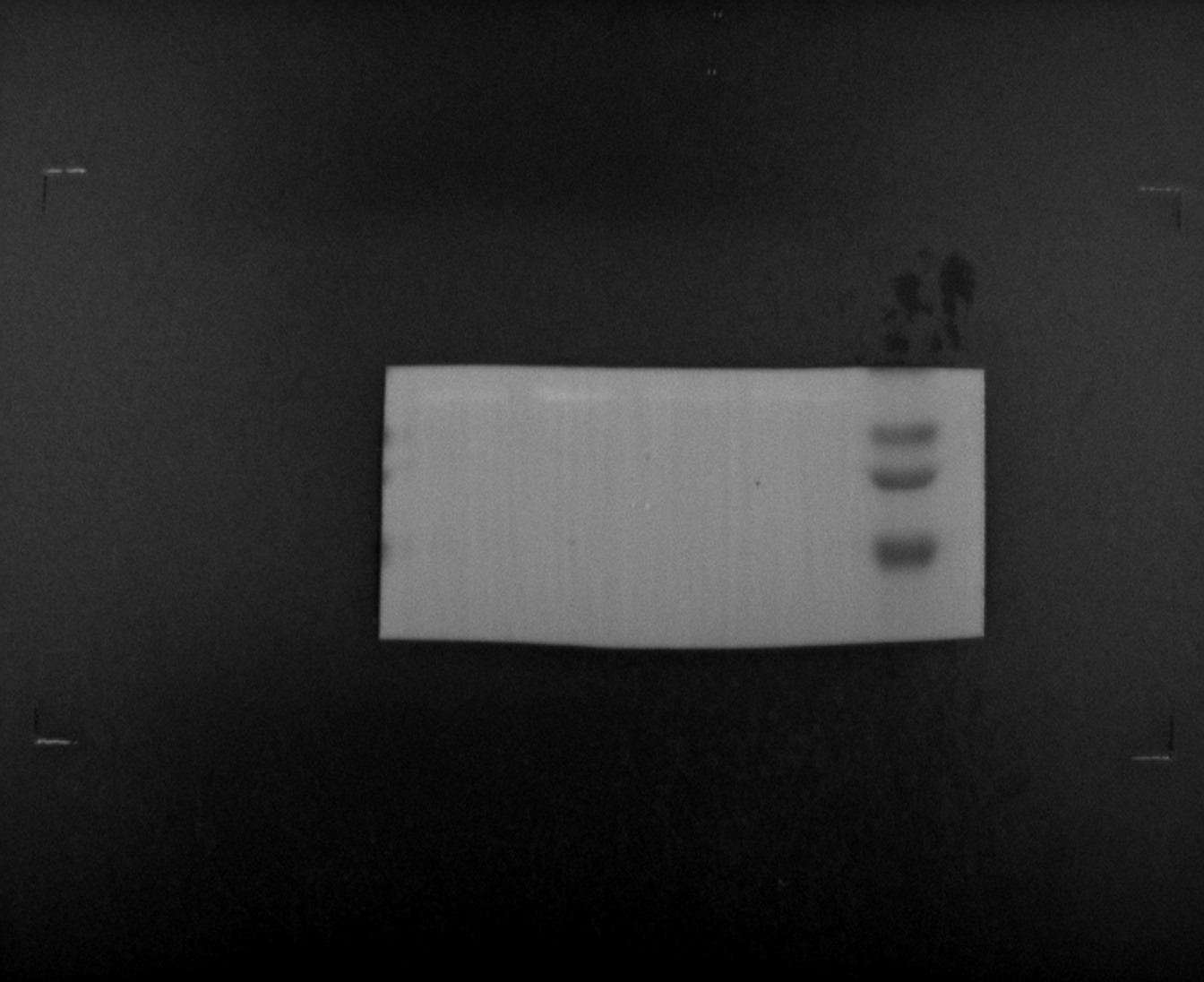

Supplement: Supplemental Information 7 [file peerj-12-17874-s007.zip › fig 4G/caspase1-3 (2).tif]

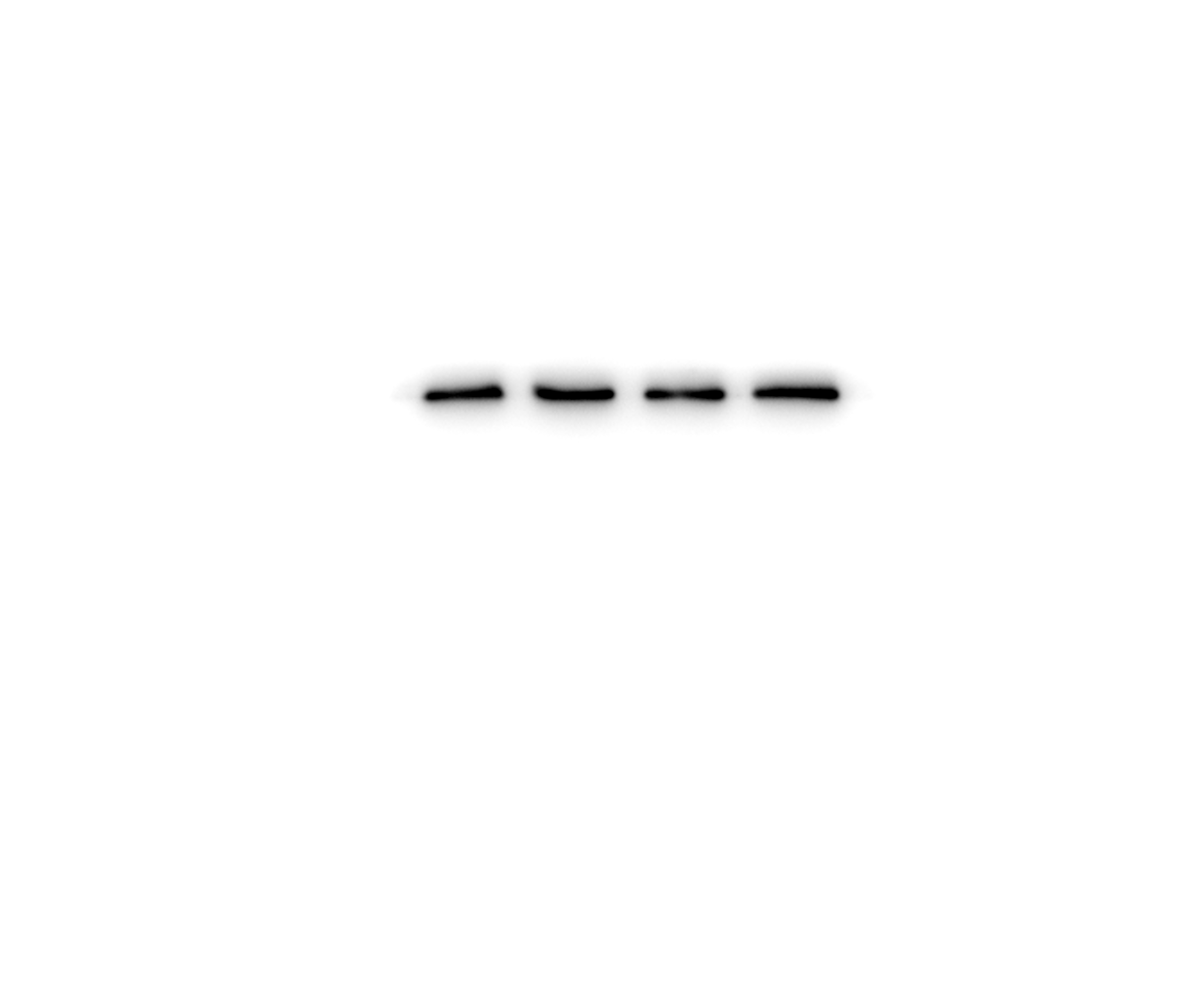

Supplement: Supplemental Information 7 [file peerj-12-17874-s007.zip › fig 4G/caspase1-3 (3).tif]

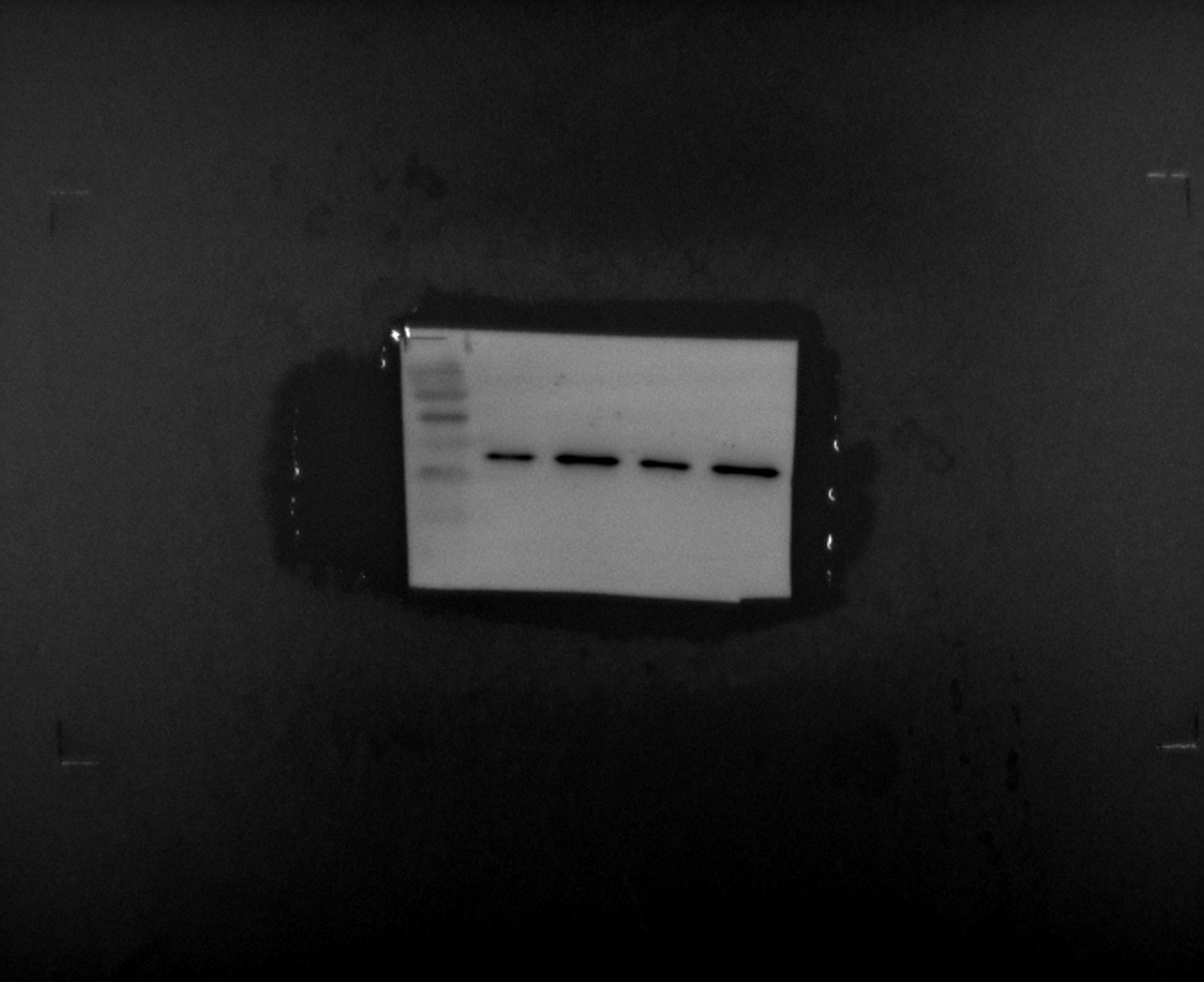

Supplement: Supplemental Information 7 [file peerj-12-17874-s007.zip › fig 4G/caspse1 (1).tif]

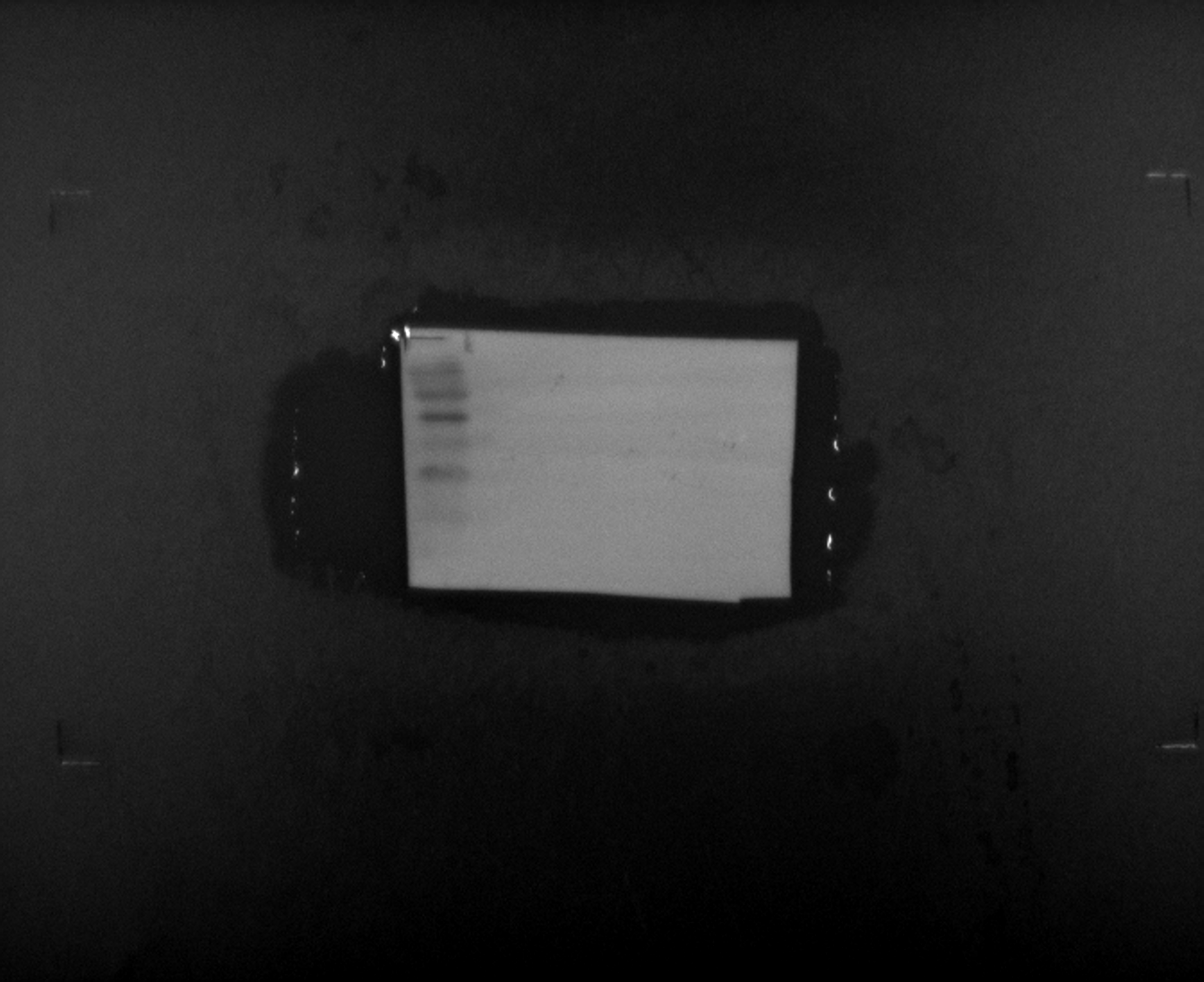

Supplement: Supplemental Information 7 [file peerj-12-17874-s007.zip › fig 4G/caspse1 (2).tif]

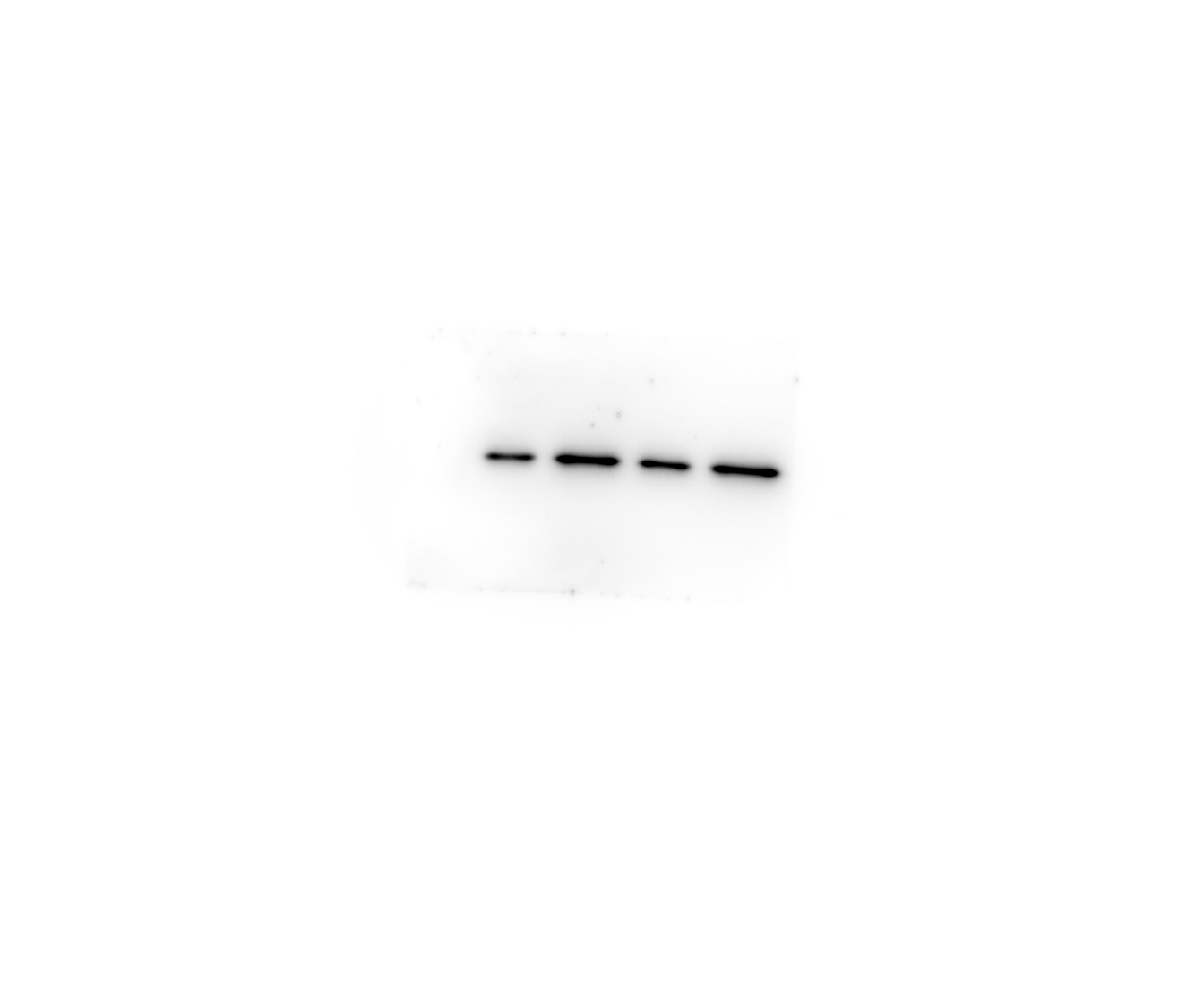

Supplement: Supplemental Information 7 [file peerj-12-17874-s007.zip › fig 4G/caspse1 (3).tif]

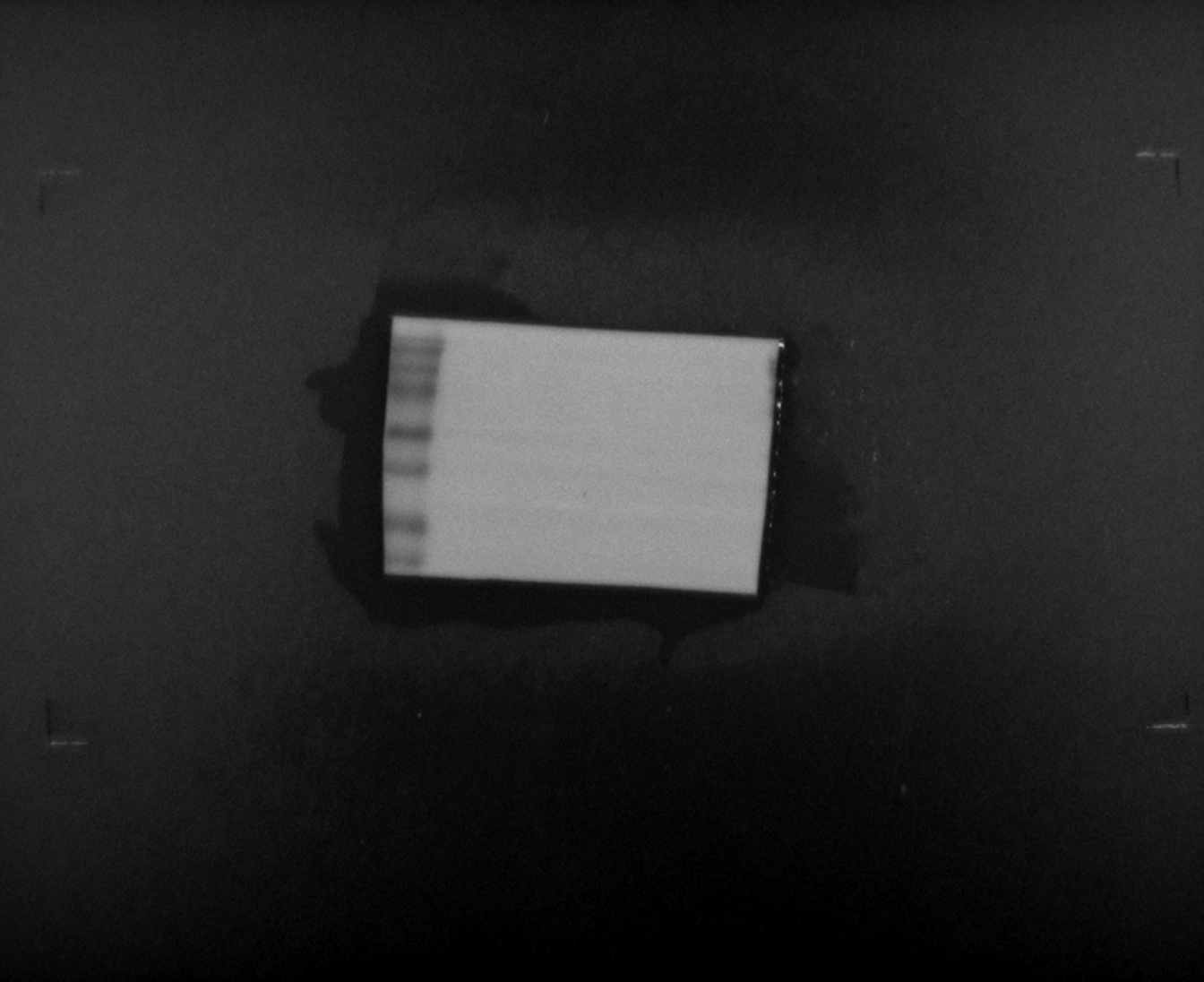

Supplement: Supplemental Information 7 [file peerj-12-17874-s007.zip › fig 4G/gapdh (1).tif]

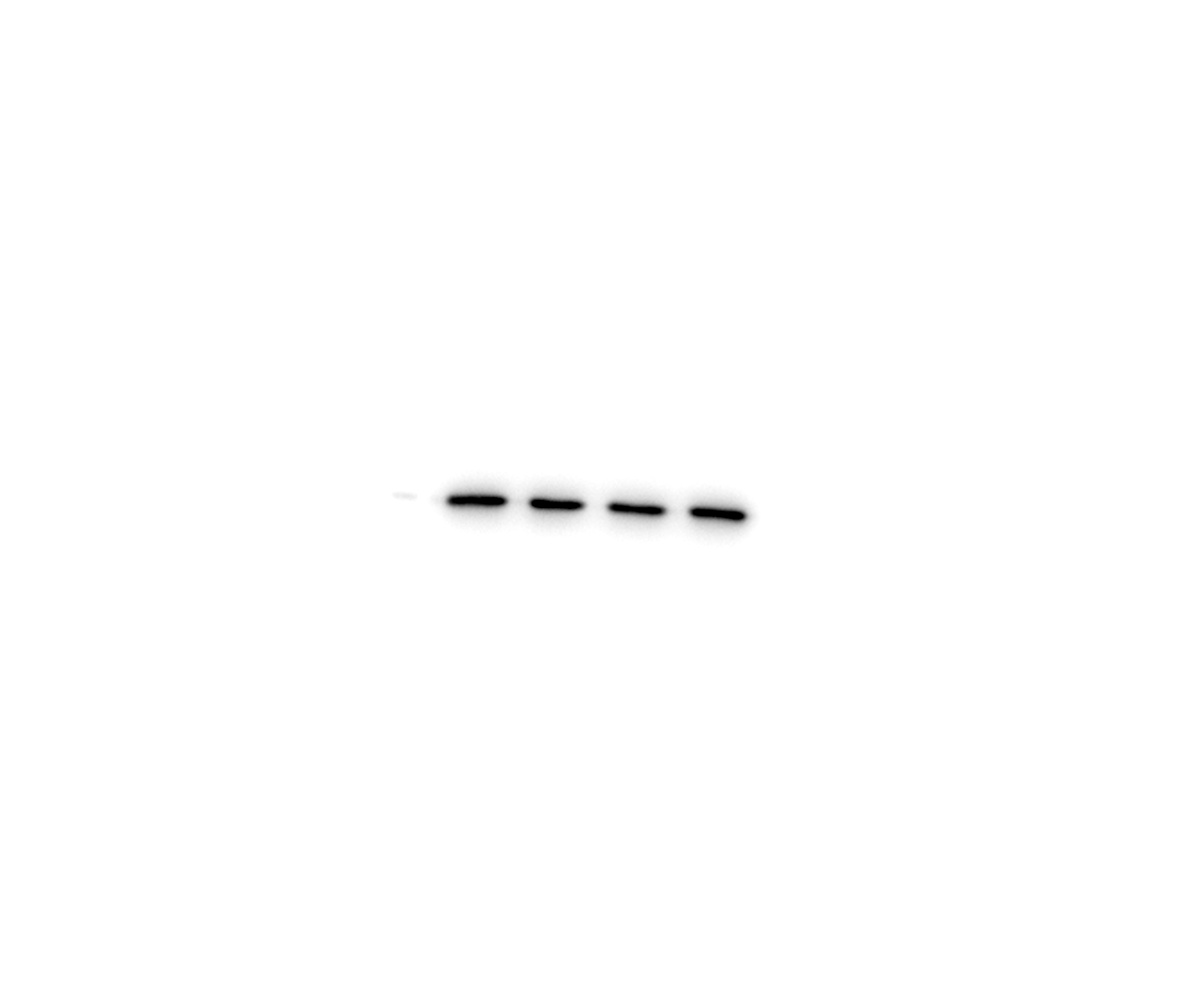

Supplement: Supplemental Information 7 [file peerj-12-17874-s007.zip › fig 4G/gapdh (2).tif]
