# Supplementary figures and images for: miR-605-3p may affect caerulein-induced ductal cell injury and pyroptosis in acute pancreatitis by targeting the DUOX2/NLRP3/NF-κB pathway (part 3 of 3)
Source: PeerJ. 2024 Aug 30;12:e17874. doi: 10.7717/peerj.17874 (PMC11368084; doi:10.7717/peerj.17874)

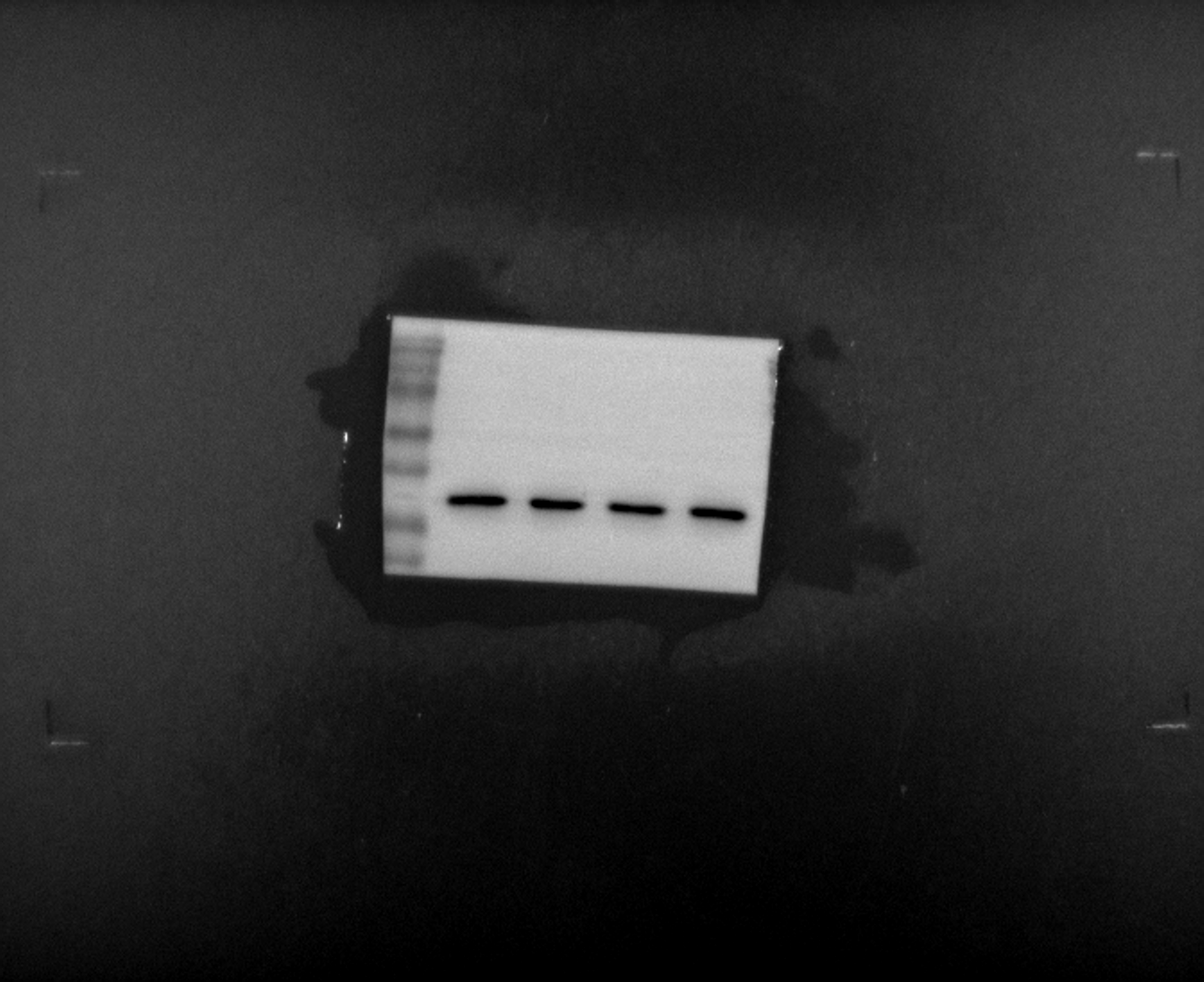

Supplement: Supplemental Information 7 [file peerj-12-17874-s007.zip › fig 4G/gapdh (3).tif]

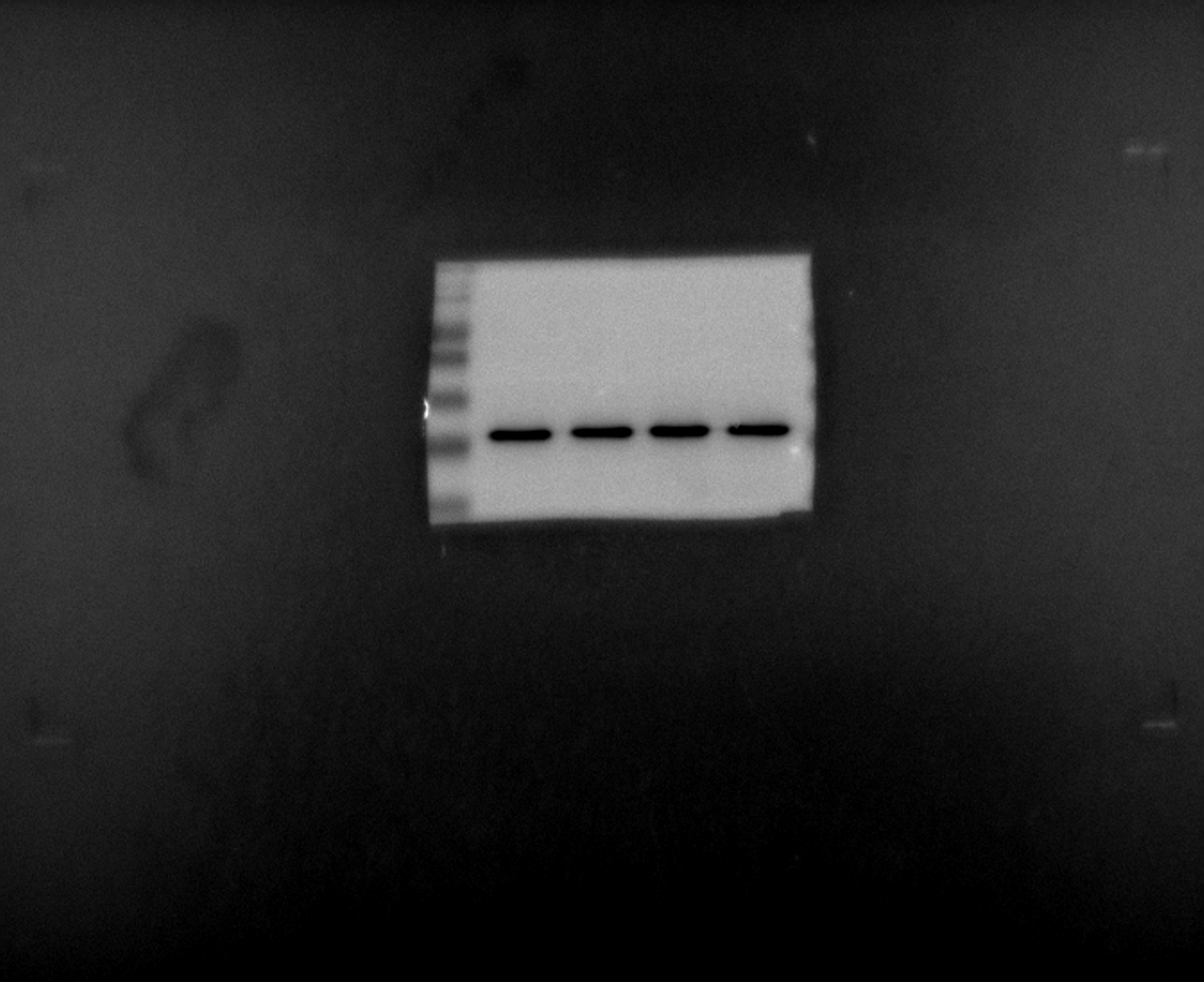

Supplement: Supplemental Information 7 [file peerj-12-17874-s007.zip › fig 4G/GAPDH-2 (1).tif]

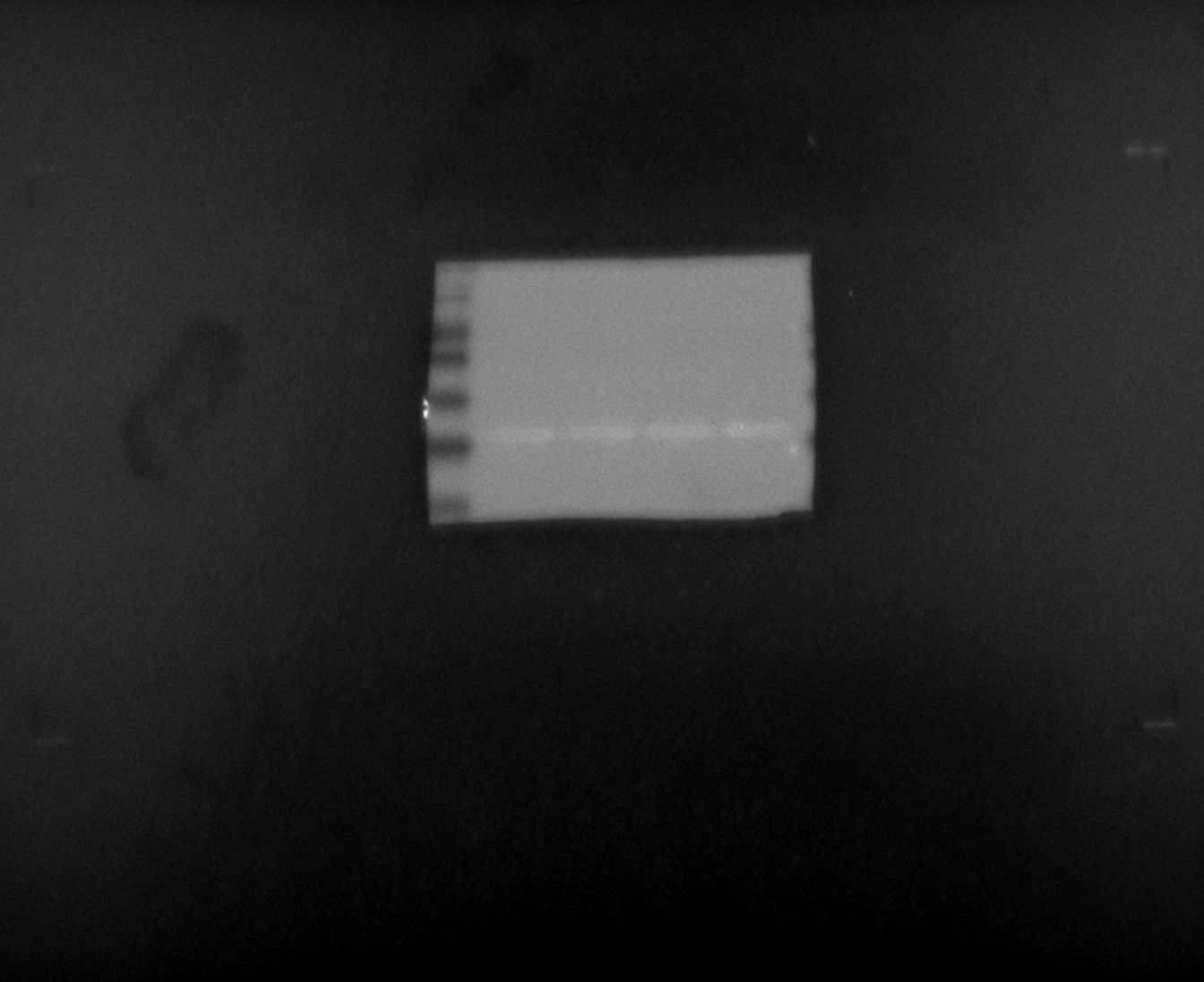

Supplement: Supplemental Information 7 [file peerj-12-17874-s007.zip › fig 4G/GAPDH-2 (2).tif]

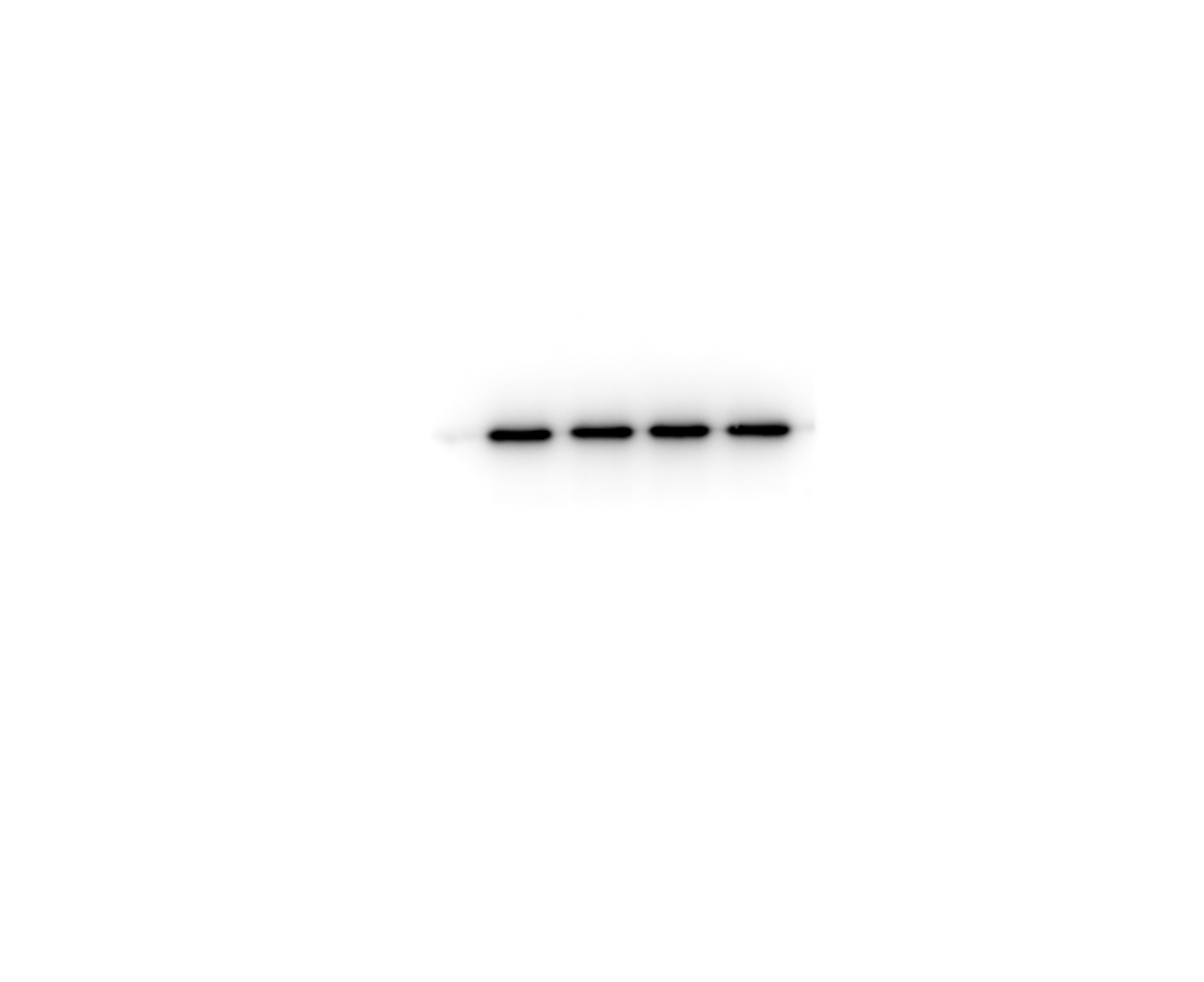

Supplement: Supplemental Information 7 [file peerj-12-17874-s007.zip › fig 4G/GAPDH-2 (3).tif]

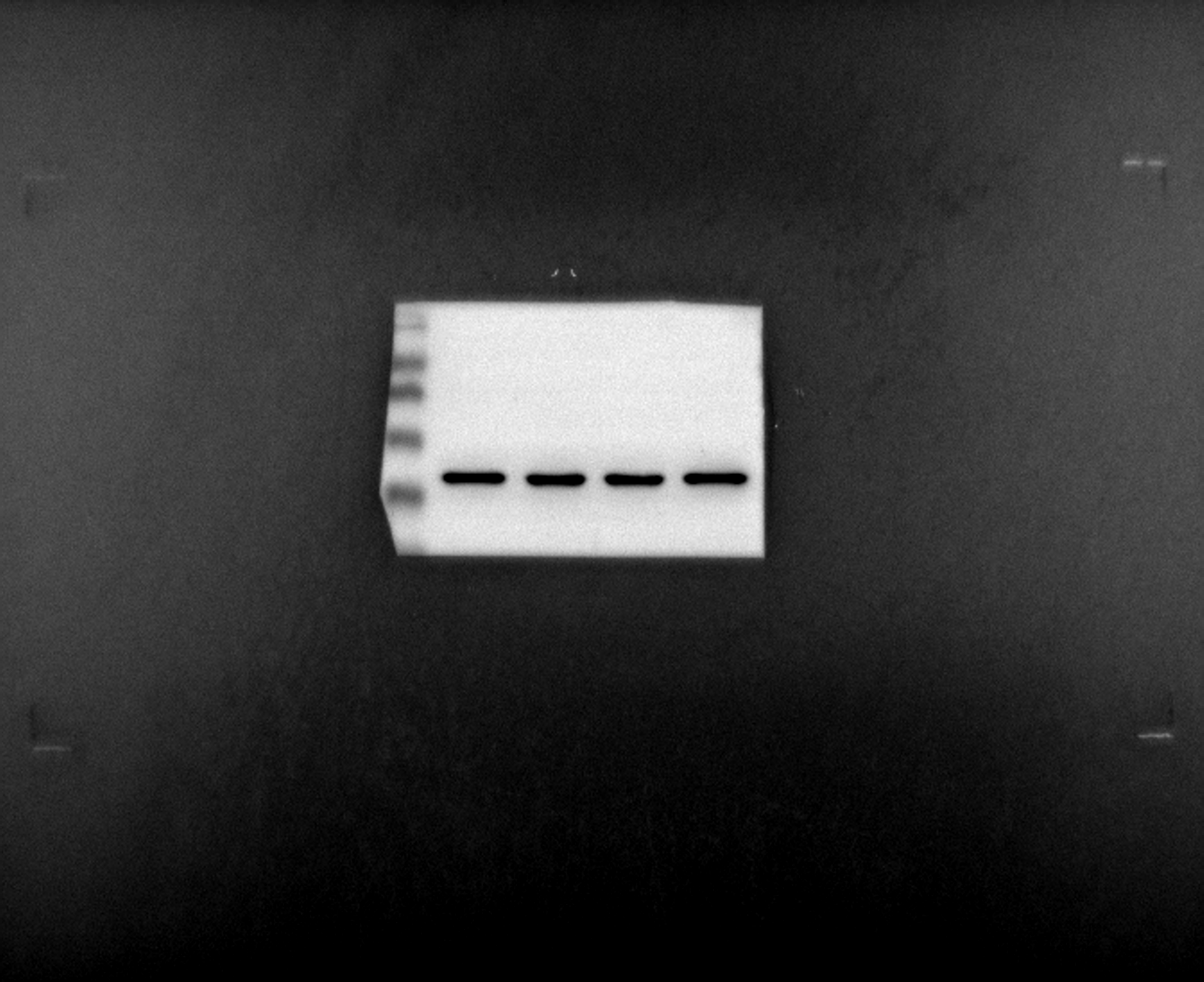

Supplement: Supplemental Information 7 [file peerj-12-17874-s007.zip › fig 4G/GAPDH-3 (1).tif]

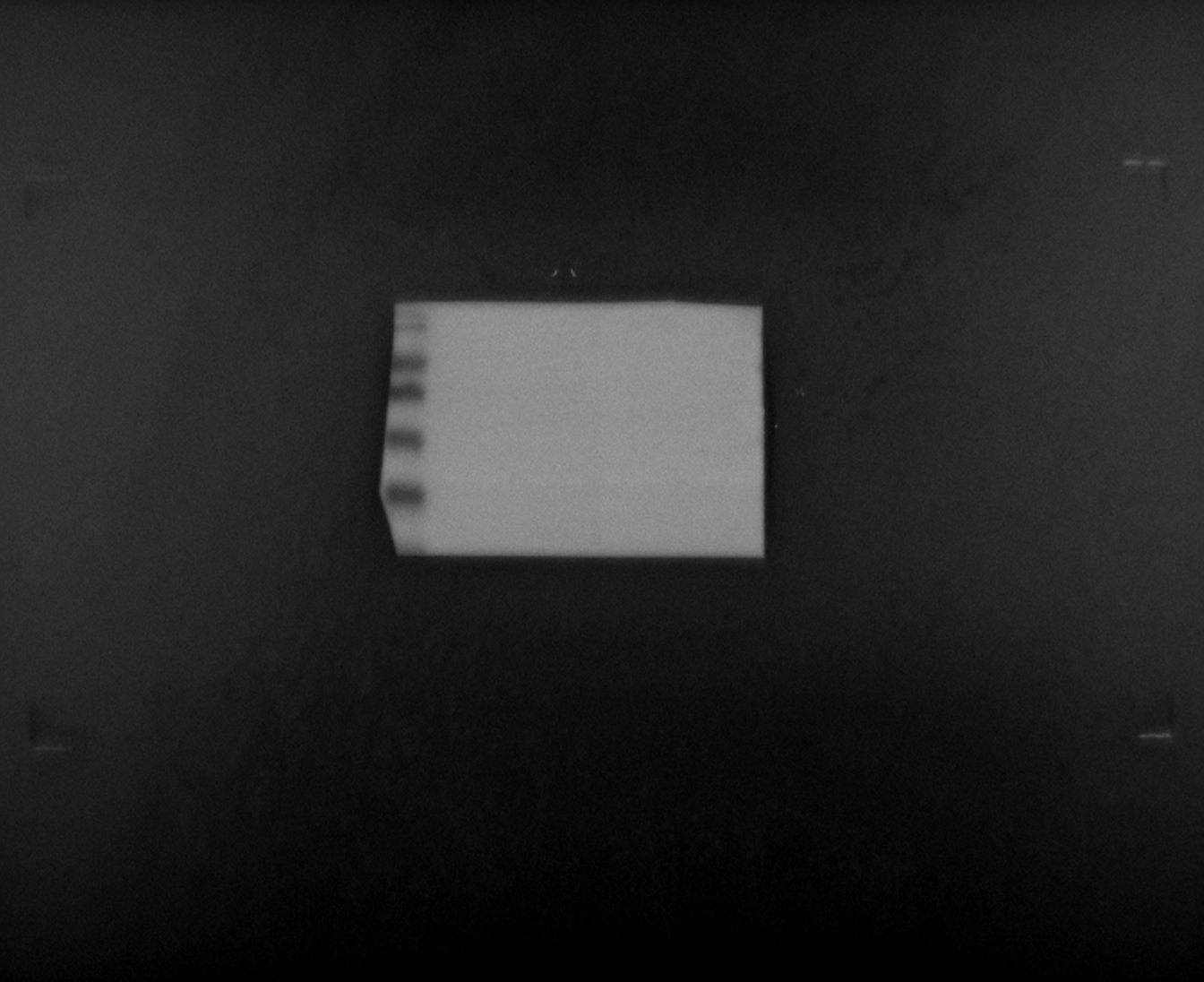

Supplement: Supplemental Information 7 [file peerj-12-17874-s007.zip › fig 4G/GAPDH-3 (2).tif]

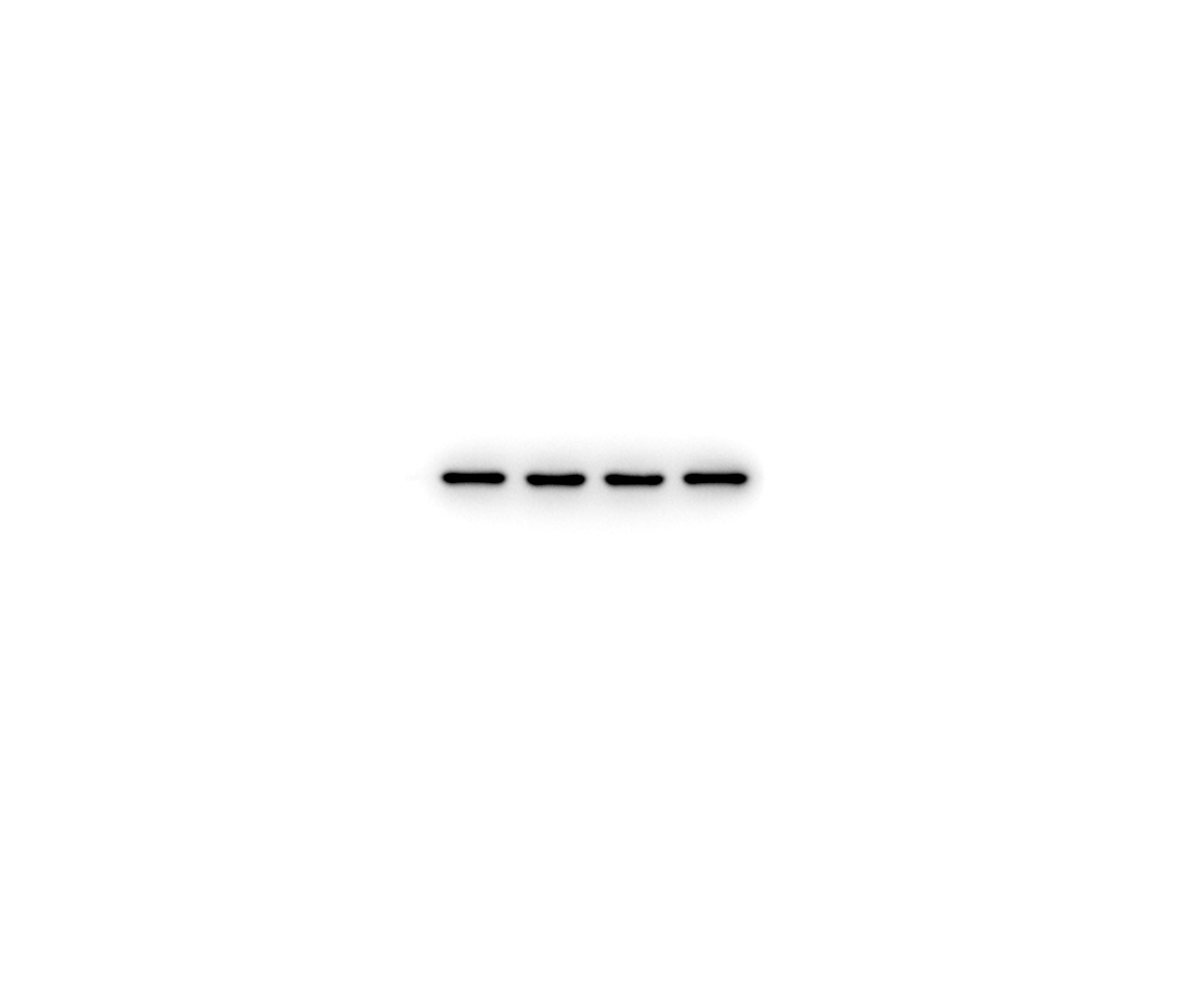

Supplement: Supplemental Information 7 [file peerj-12-17874-s007.zip › fig 4G/GAPDH-3 (3).tif]

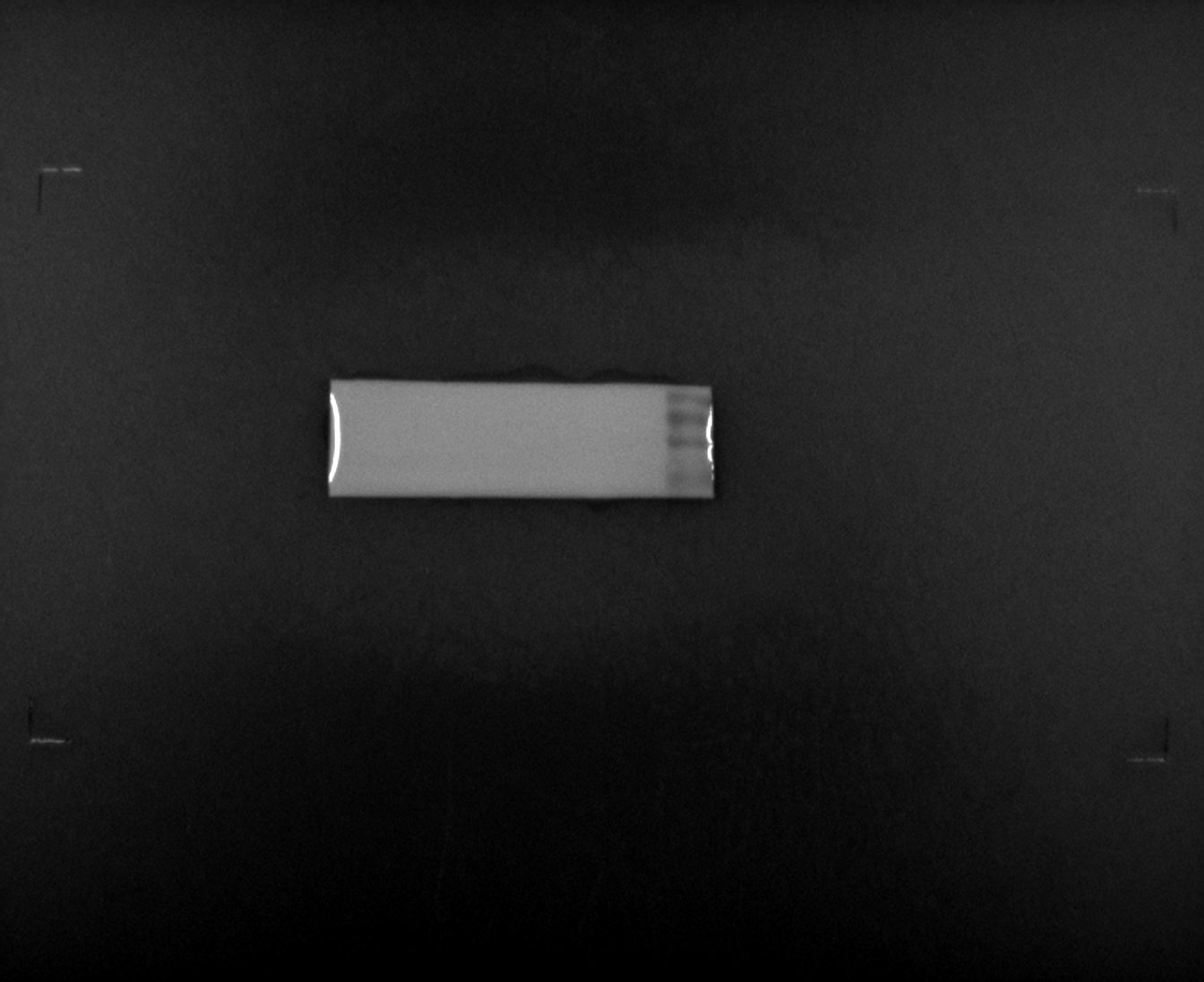

Supplement: Supplemental Information 7 [file peerj-12-17874-s007.zip › fig 4G/nlrp3-2 (1).tif]

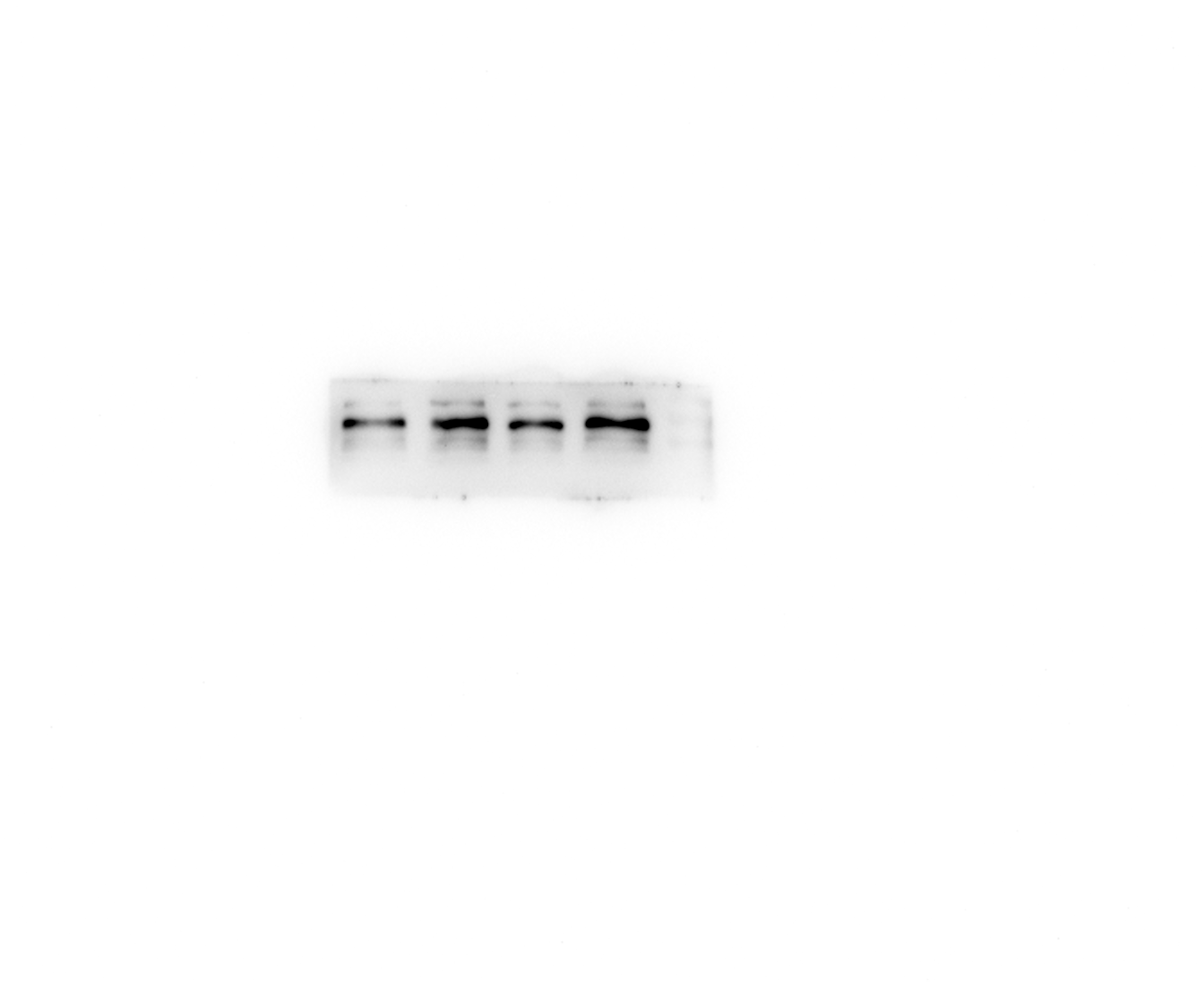

Supplement: Supplemental Information 7 [file peerj-12-17874-s007.zip › fig 4G/nlrp3-2 (2).tif]

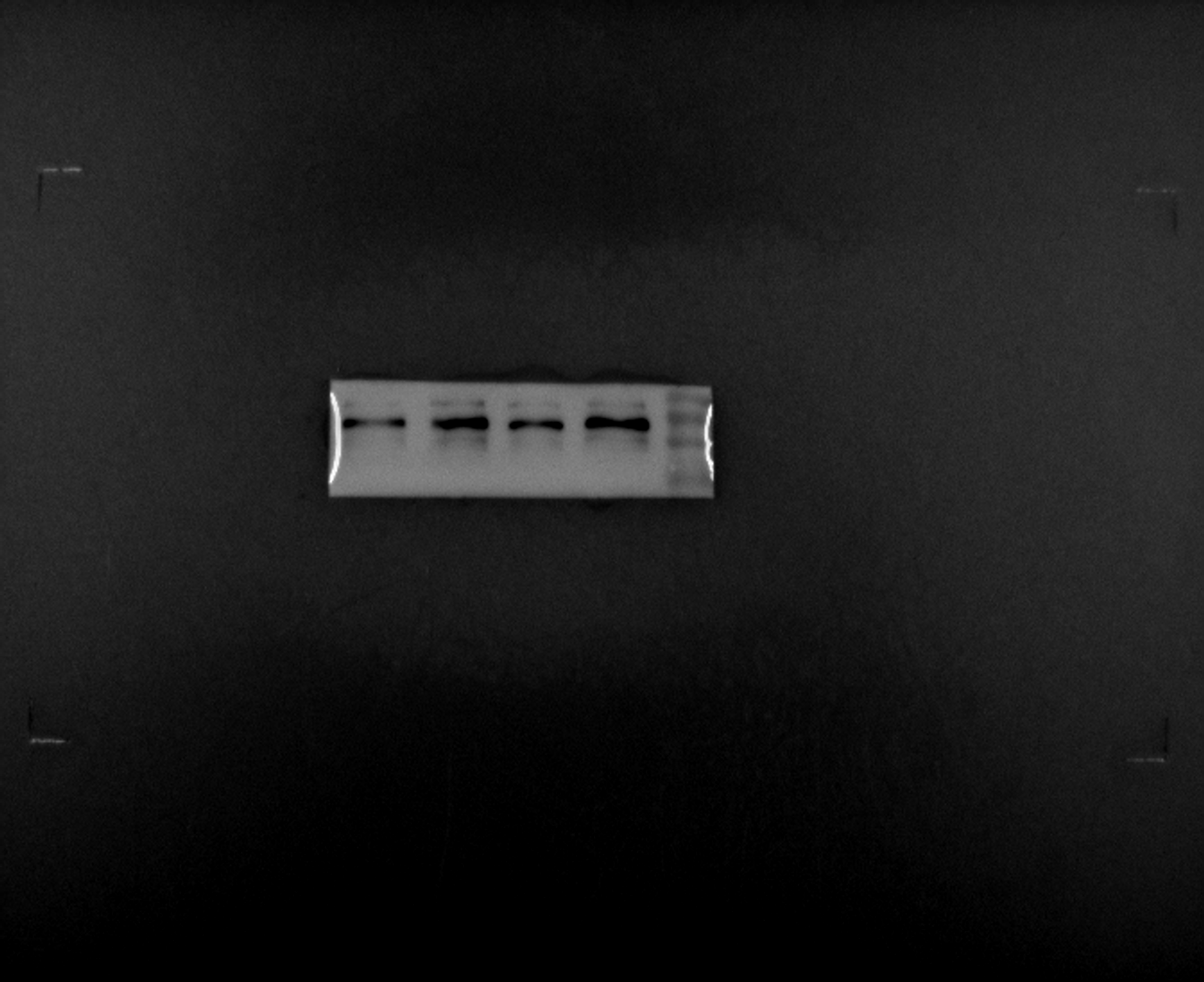

Supplement: Supplemental Information 7 [file peerj-12-17874-s007.zip › fig 4G/nlrp3-2 (3).tif]

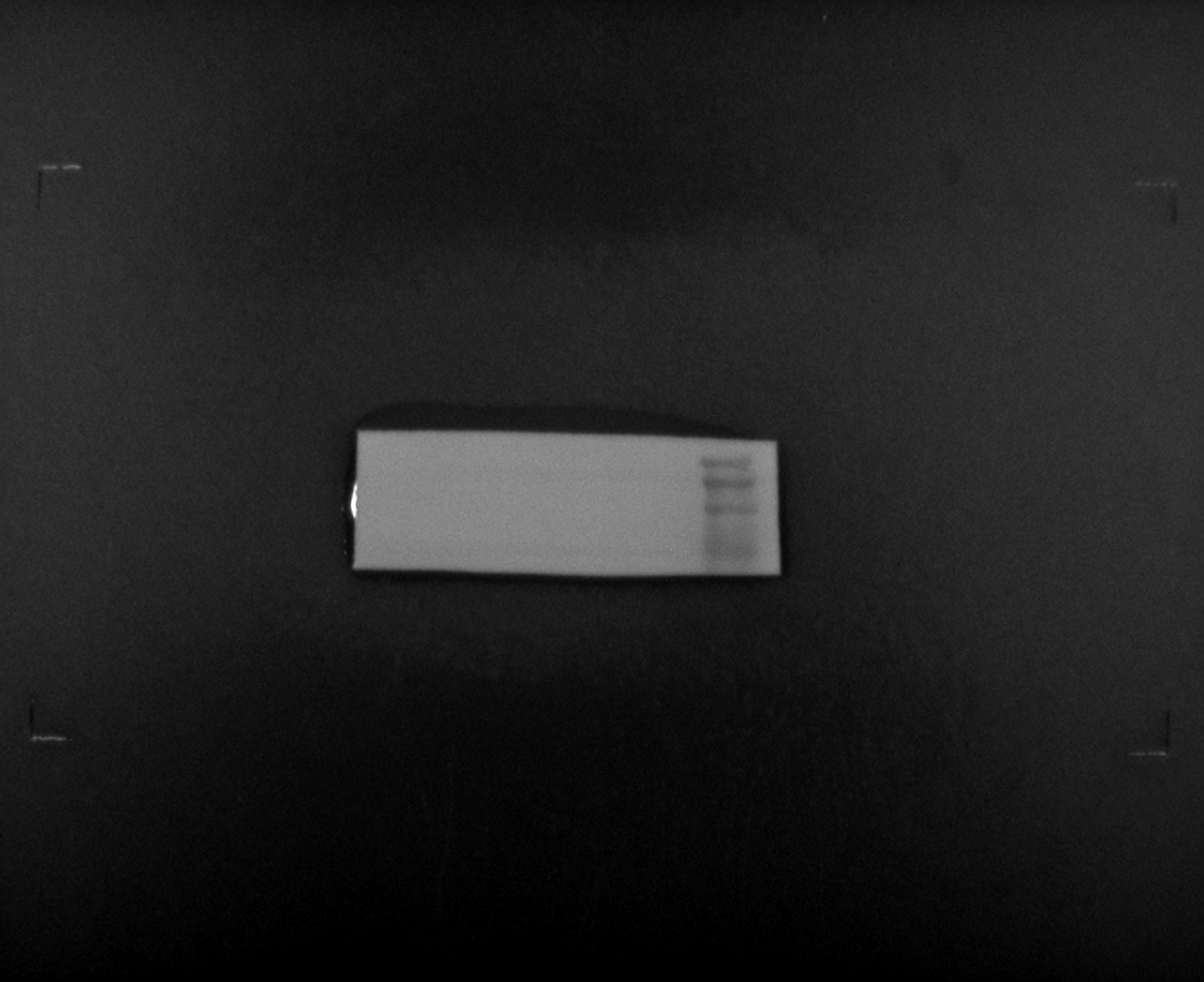

Supplement: Supplemental Information 7 [file peerj-12-17874-s007.zip › fig 4G/nlrp3-3 (1).tif]

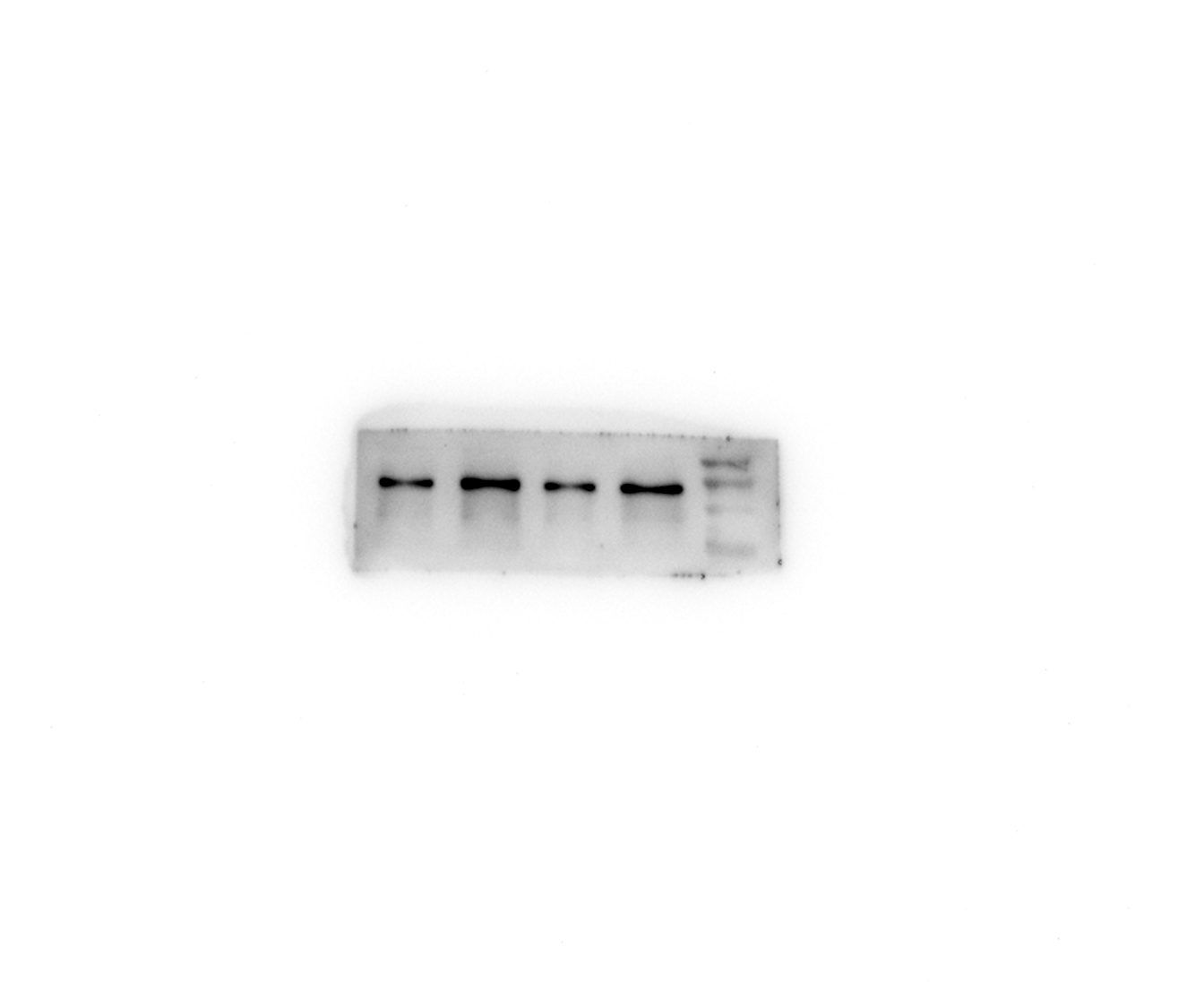

Supplement: Supplemental Information 7 [file peerj-12-17874-s007.zip › fig 4G/nlrp3-3 (2).tif]

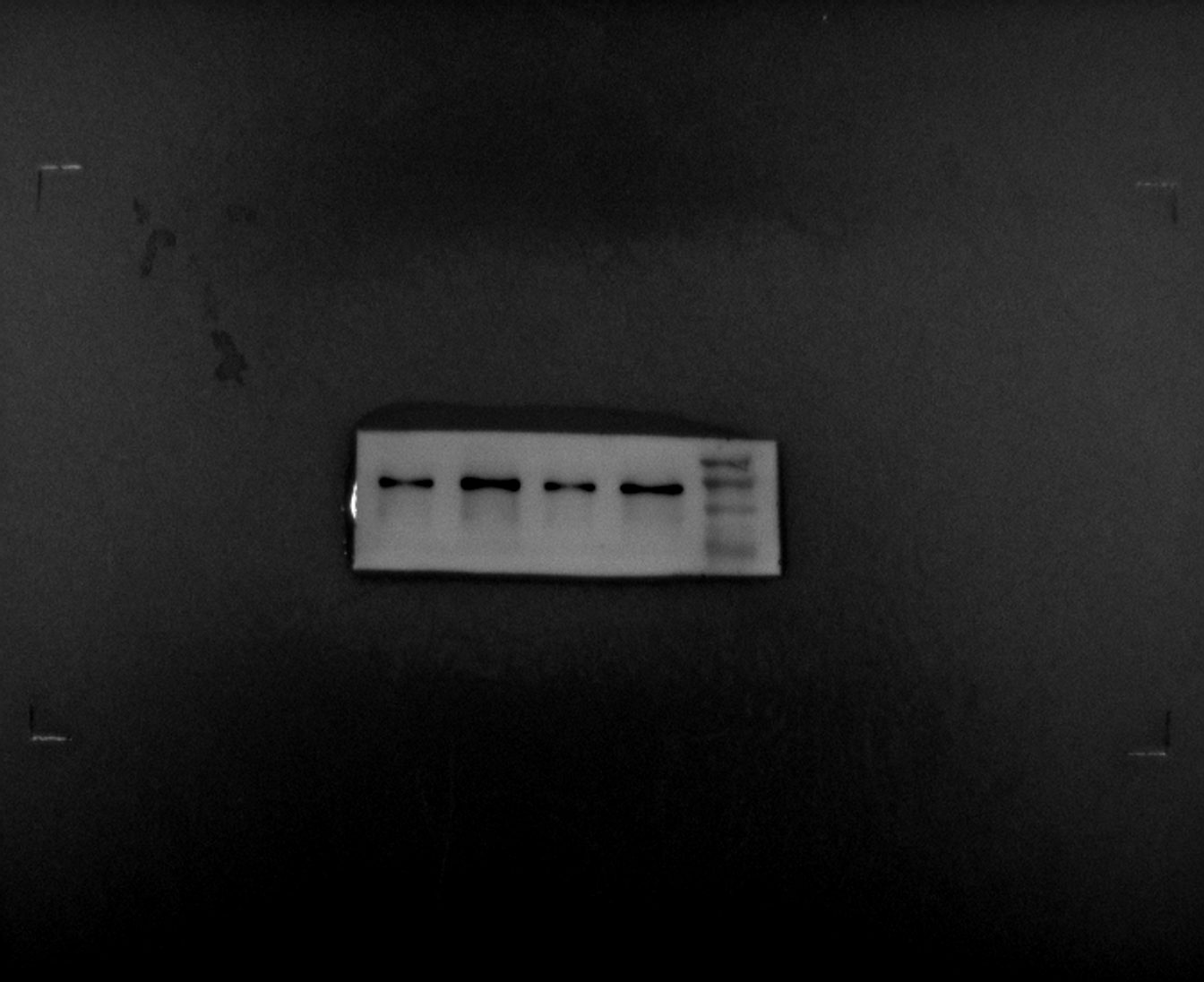

Supplement: Supplemental Information 7 [file peerj-12-17874-s007.zip › fig 4G/nlrp3-3 (3).tif]

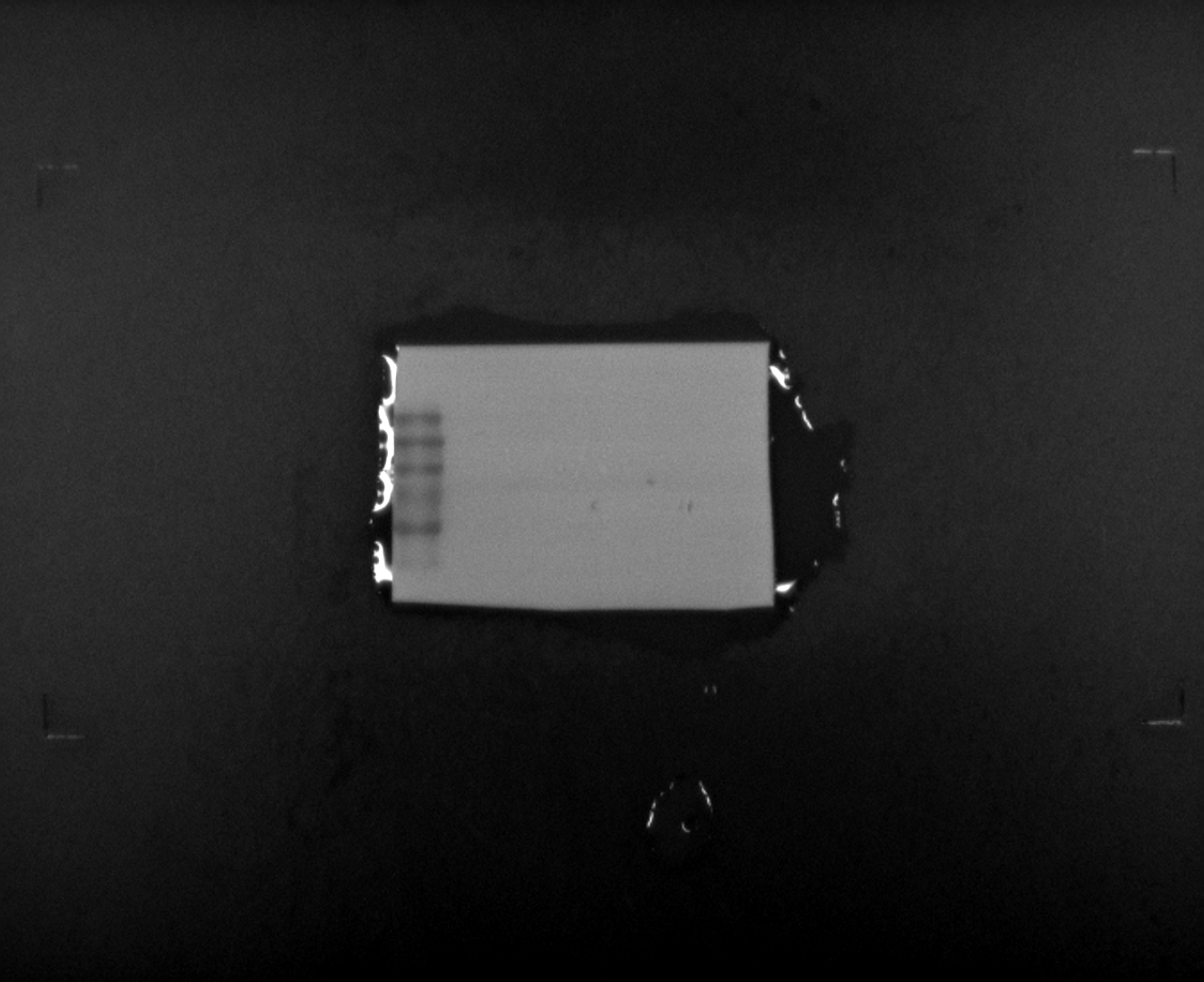

Supplement: Supplemental Information 7 [file peerj-12-17874-s007.zip › fig 4G/NRLP3 (1).tif]

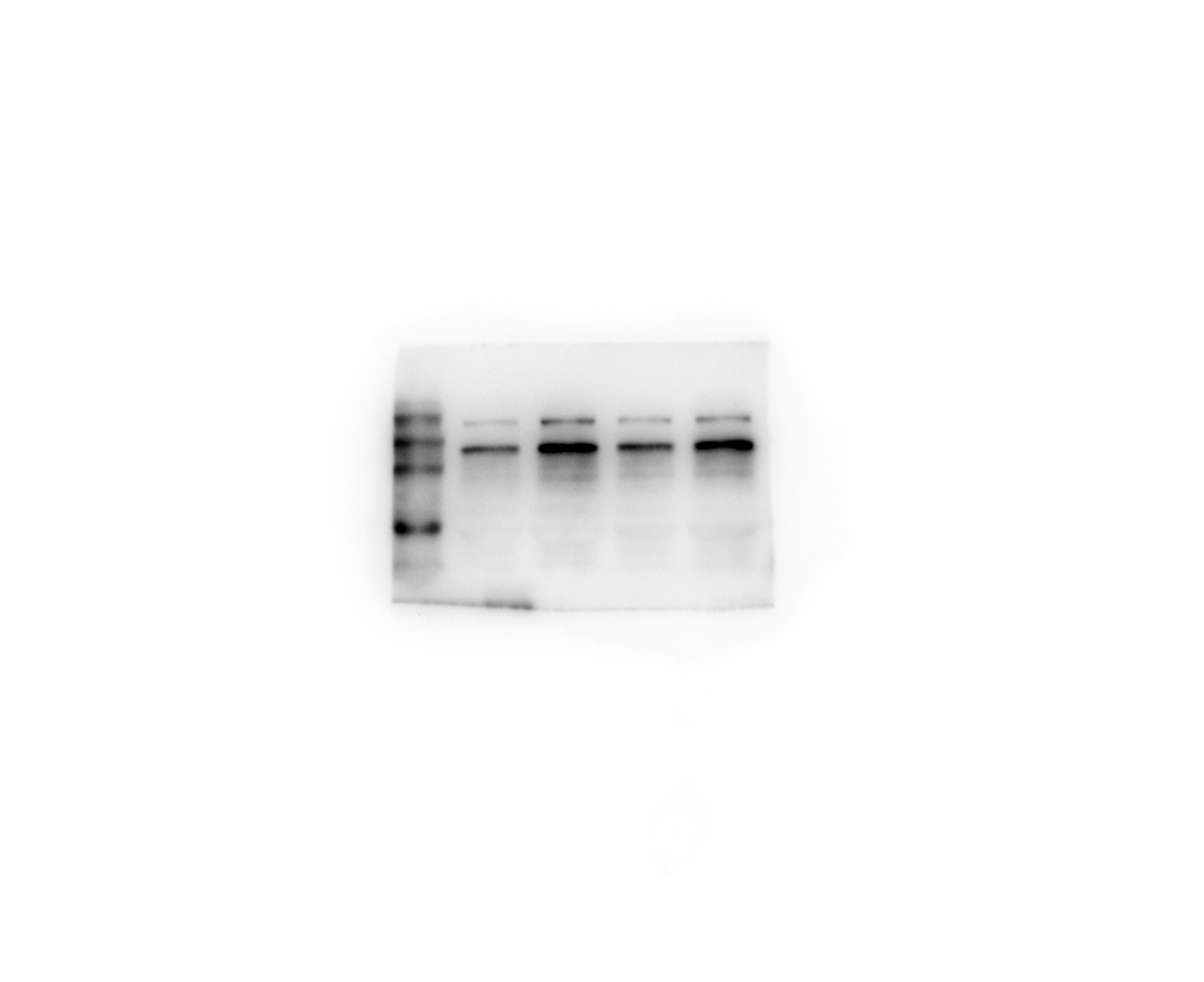

Supplement: Supplemental Information 7 [file peerj-12-17874-s007.zip › fig 4G/NRLP3 (2).tif]

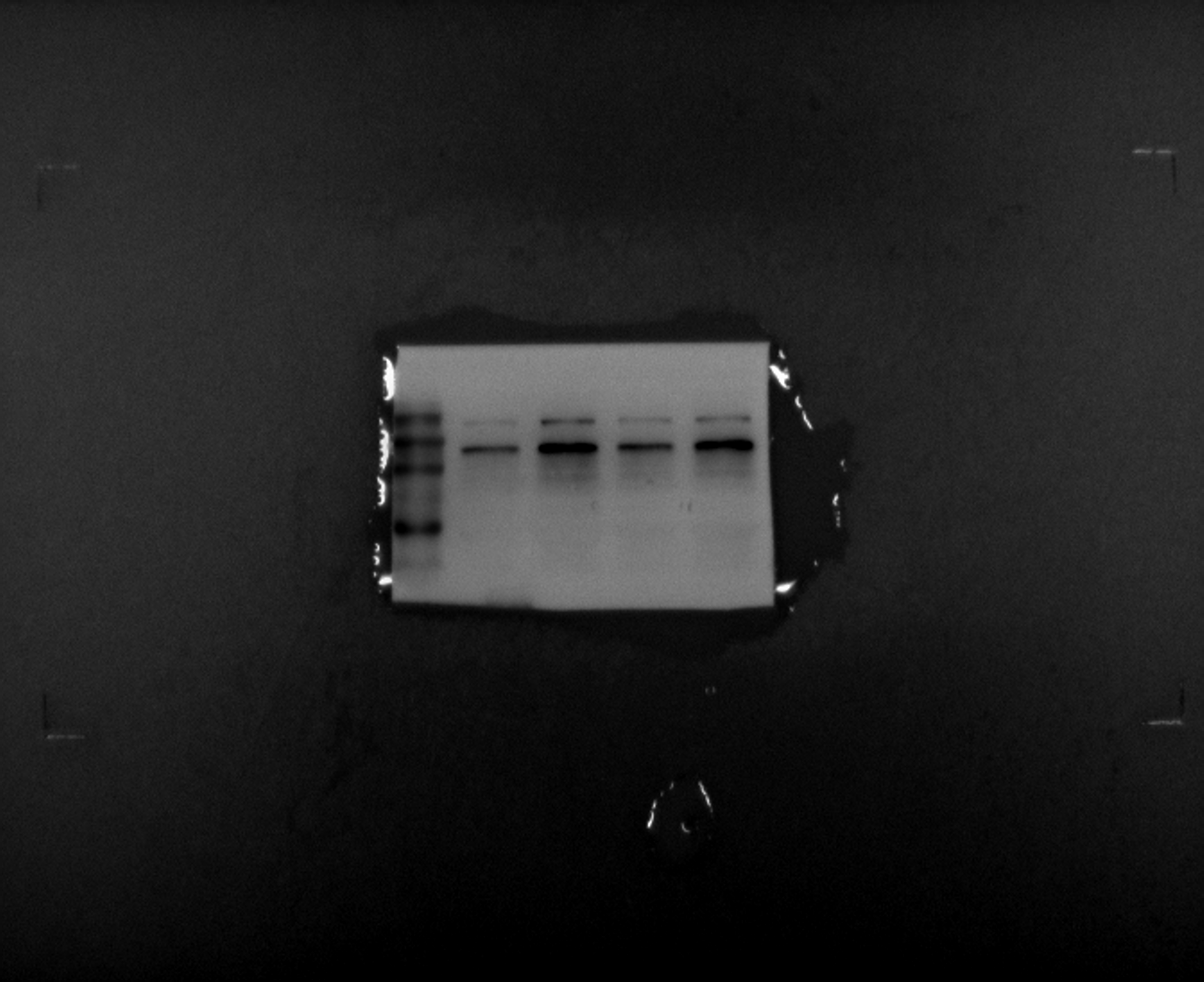

Supplement: Supplemental Information 7 [file peerj-12-17874-s007.zip › fig 4G/NRLP3 (3).tif]

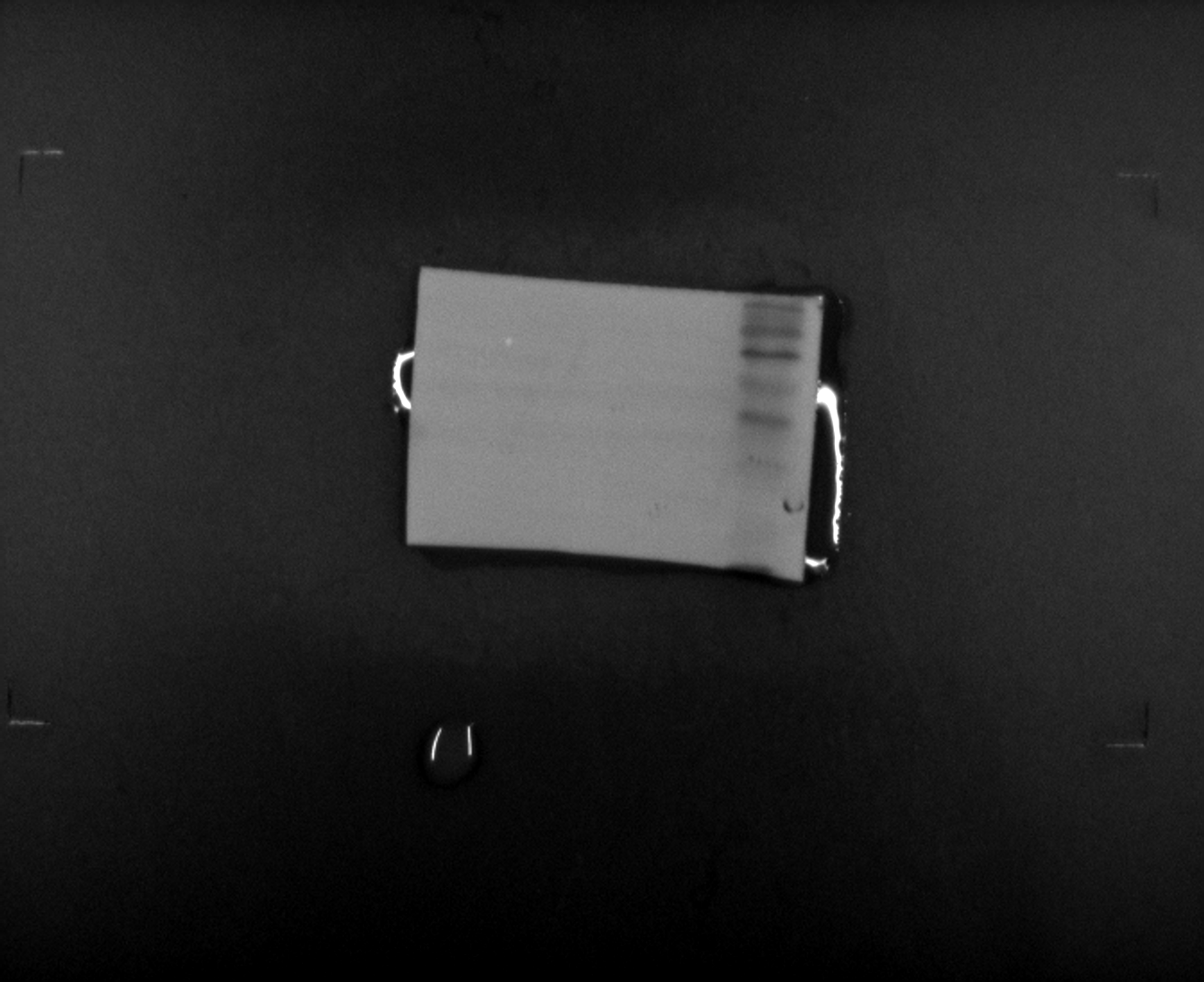

Supplement: Supplemental Information 7 [file peerj-12-17874-s007.zip › fig 4G/p65 (1).tif]

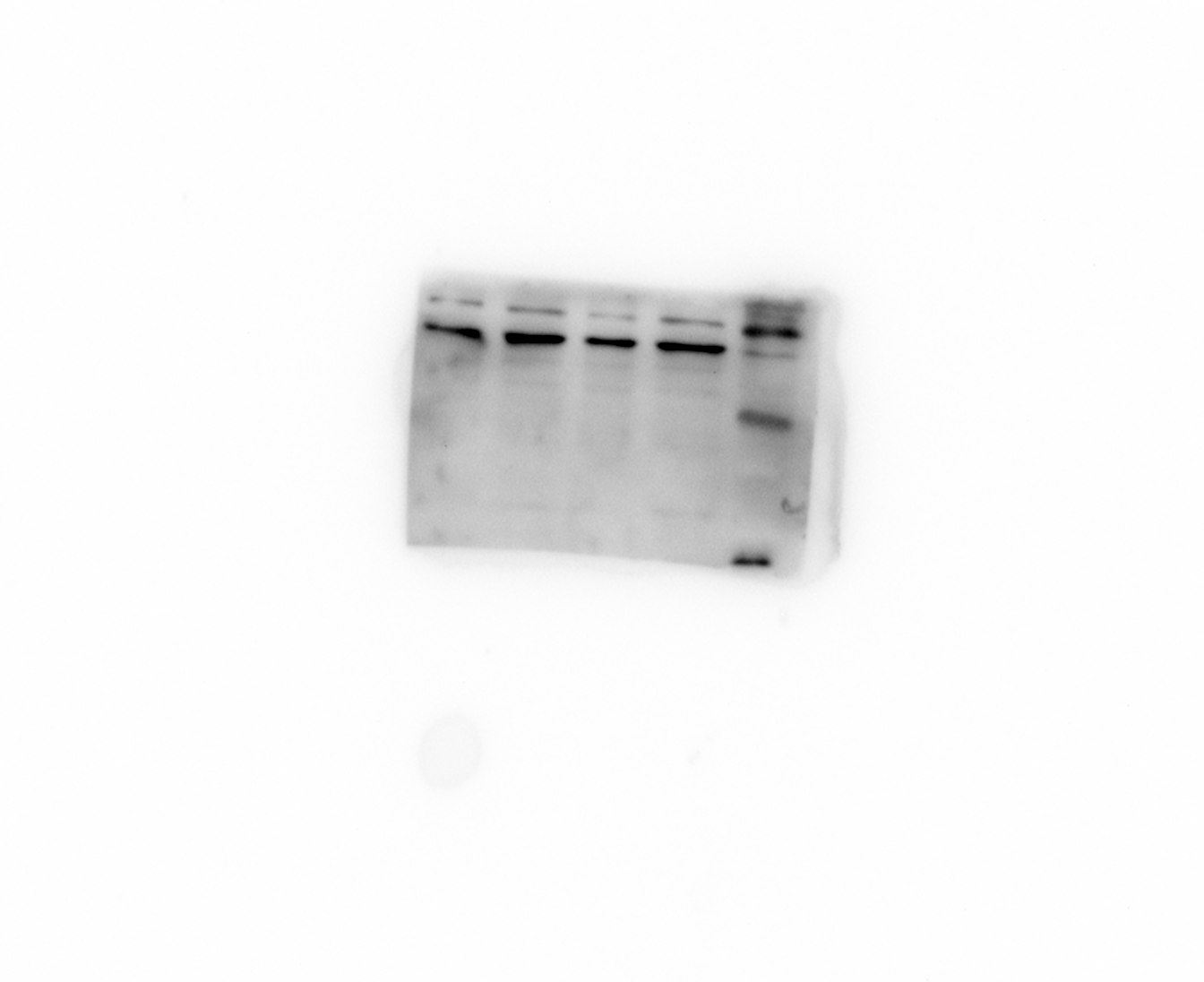

Supplement: Supplemental Information 7 [file peerj-12-17874-s007.zip › fig 4G/p65 (2).tif]

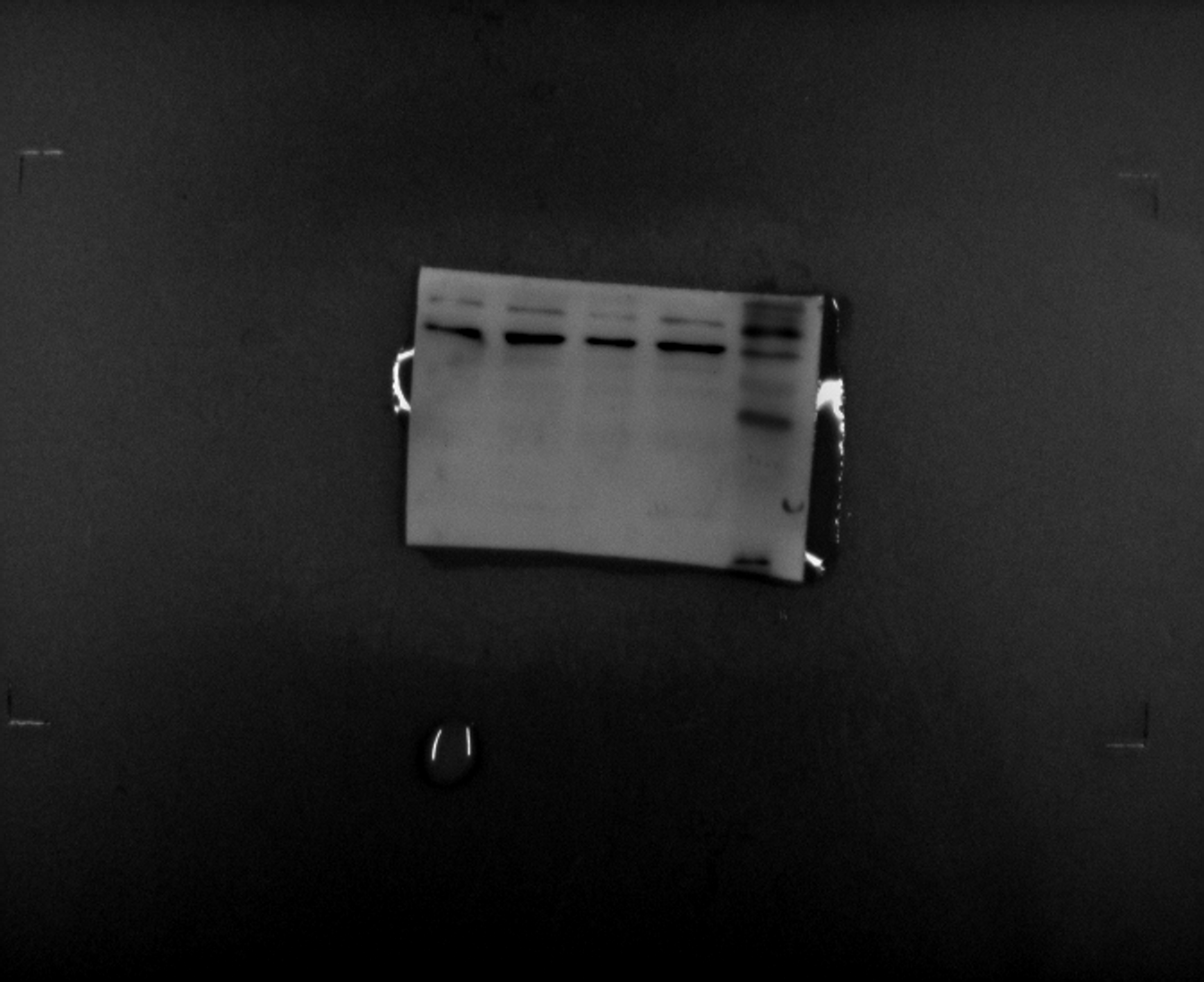

Supplement: Supplemental Information 7 [file peerj-12-17874-s007.zip › fig 4G/p65 (3).tif]

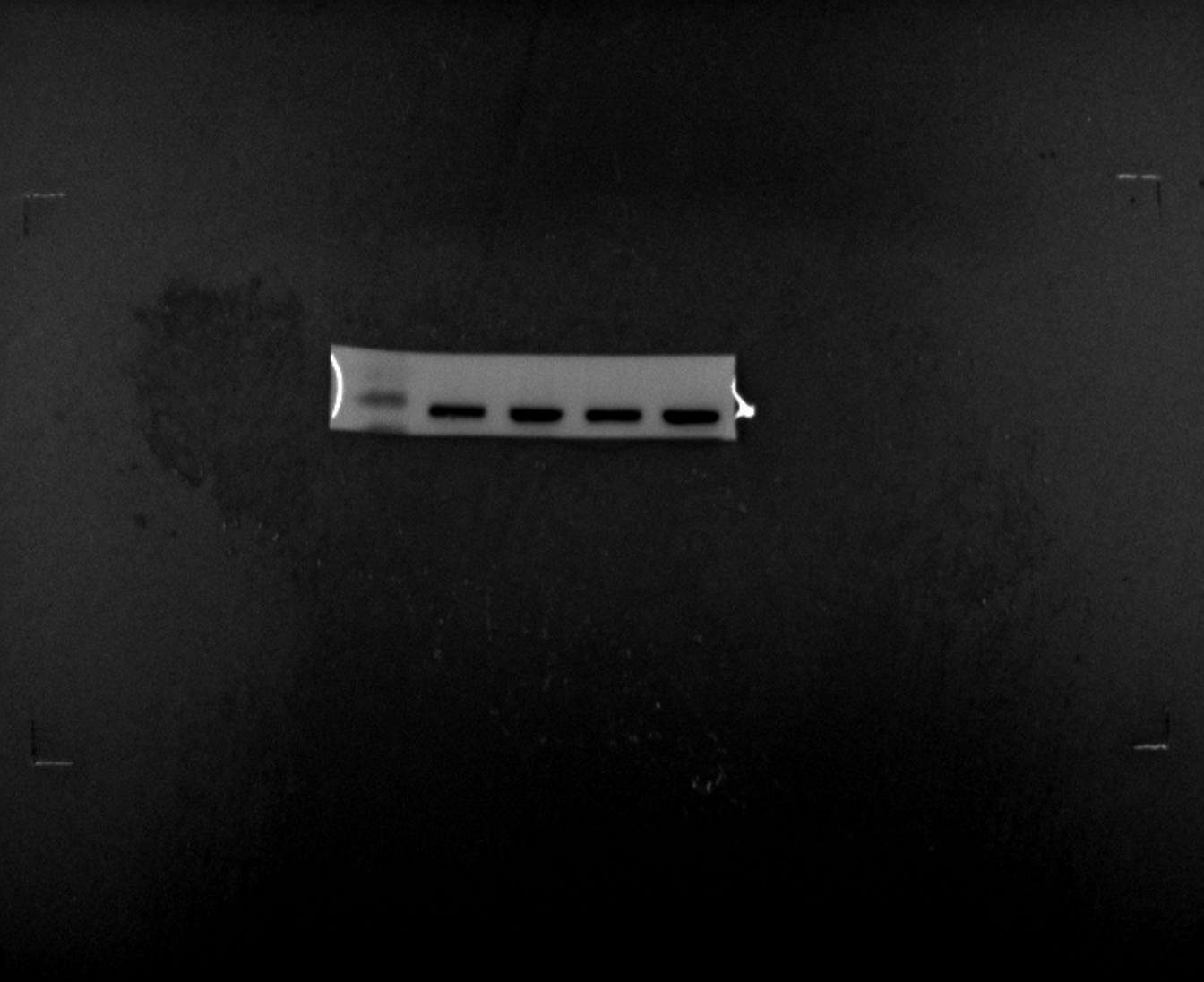

Supplement: Supplemental Information 7 [file peerj-12-17874-s007.zip › fig 4G/p-p65-2 (1).tif]

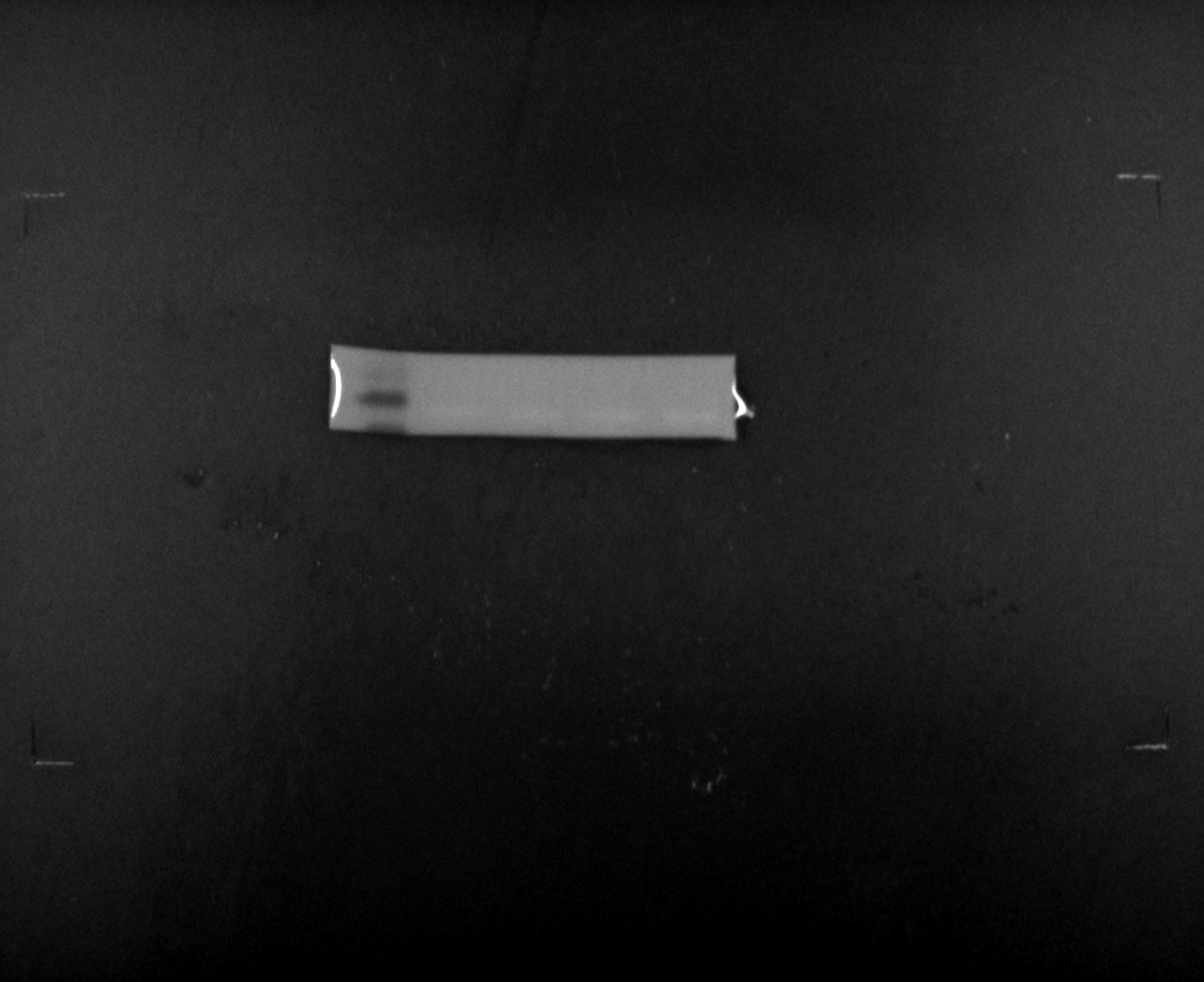

Supplement: Supplemental Information 7 [file peerj-12-17874-s007.zip › fig 4G/p-p65-2 (2).tif]

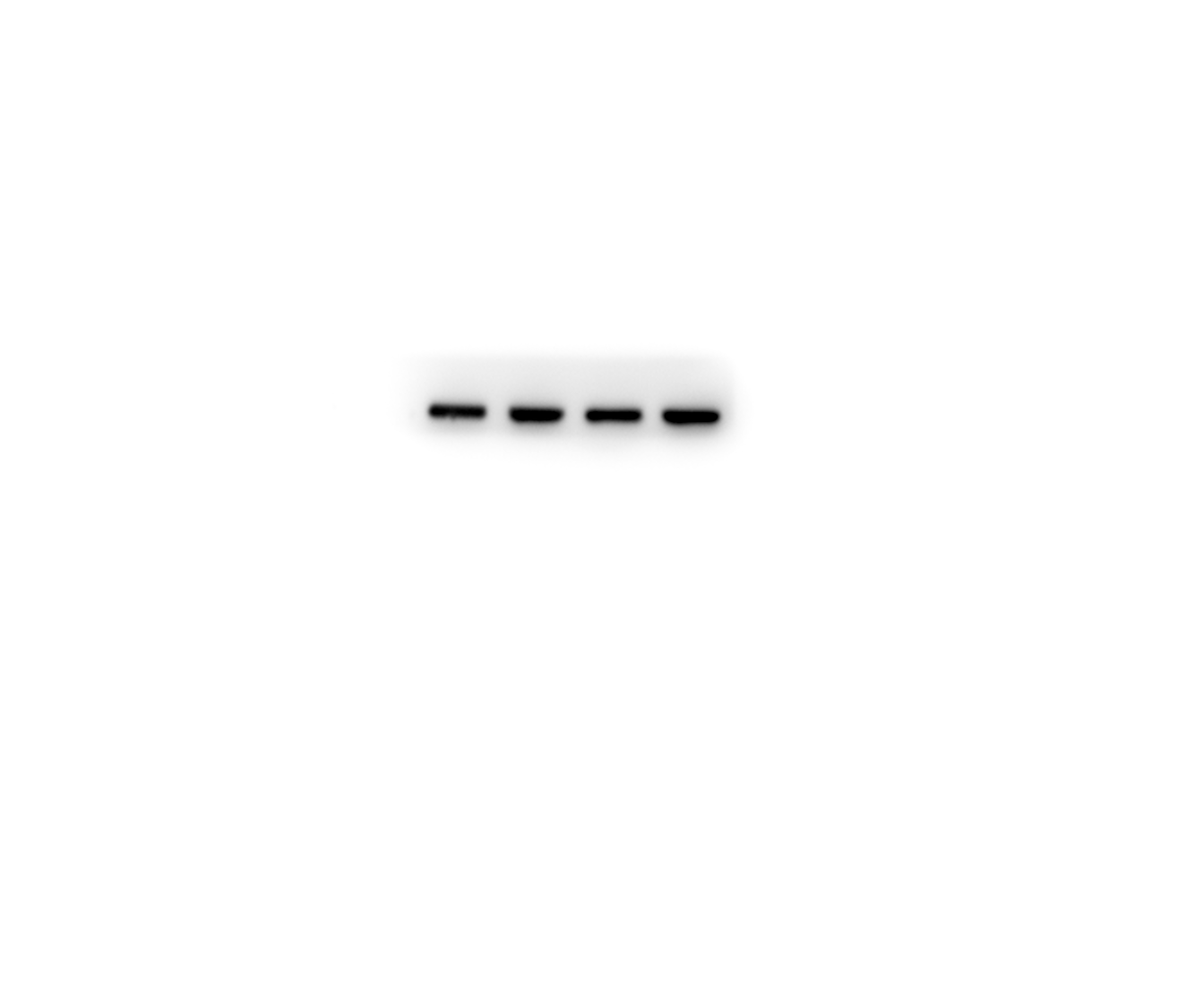

Supplement: Supplemental Information 7 [file peerj-12-17874-s007.zip › fig 4G/p-p65-2 (3).tif]

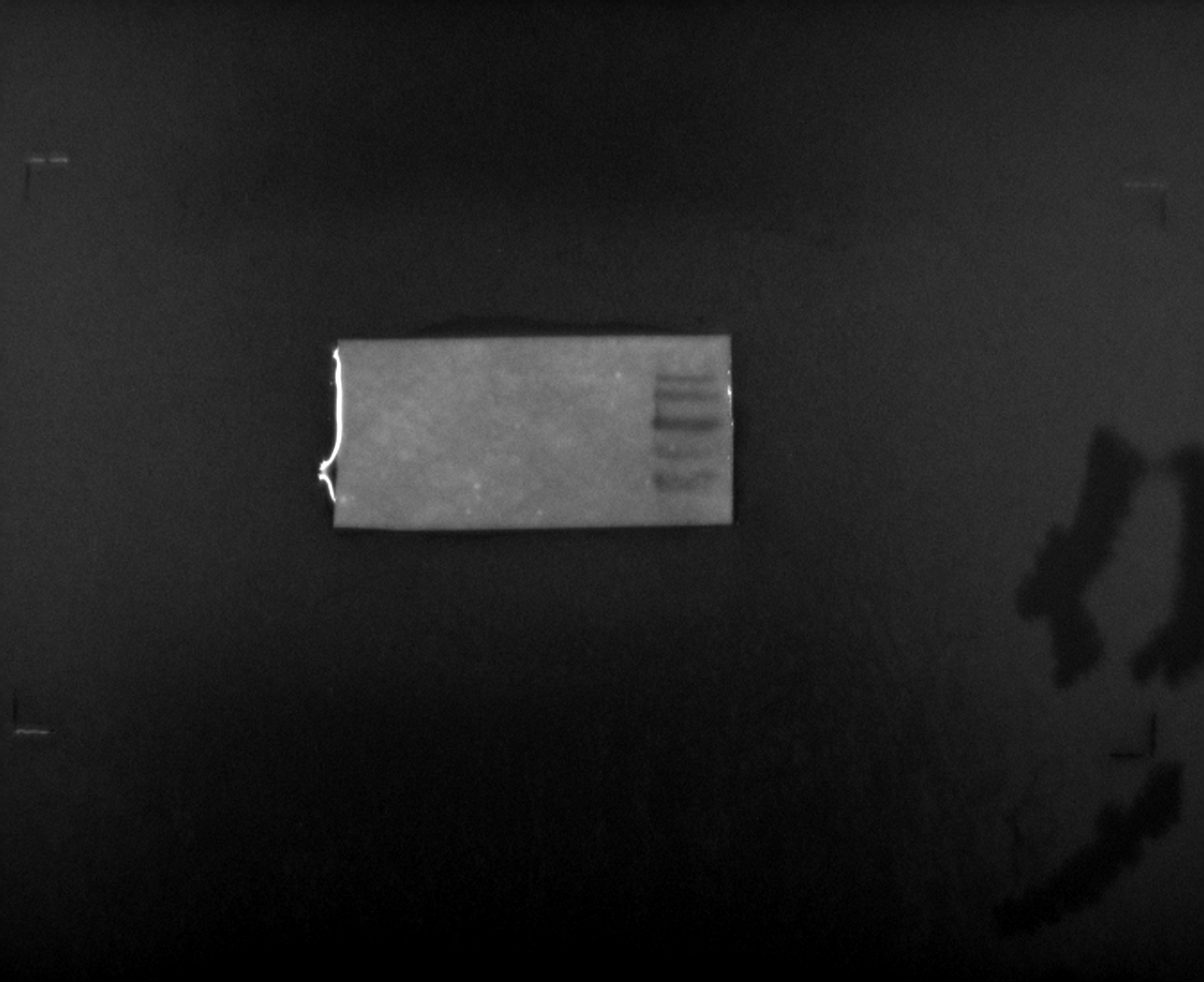

Supplement: Supplemental Information 7 [file peerj-12-17874-s007.zip › fig 4G/p-p65-3 (1).tif]

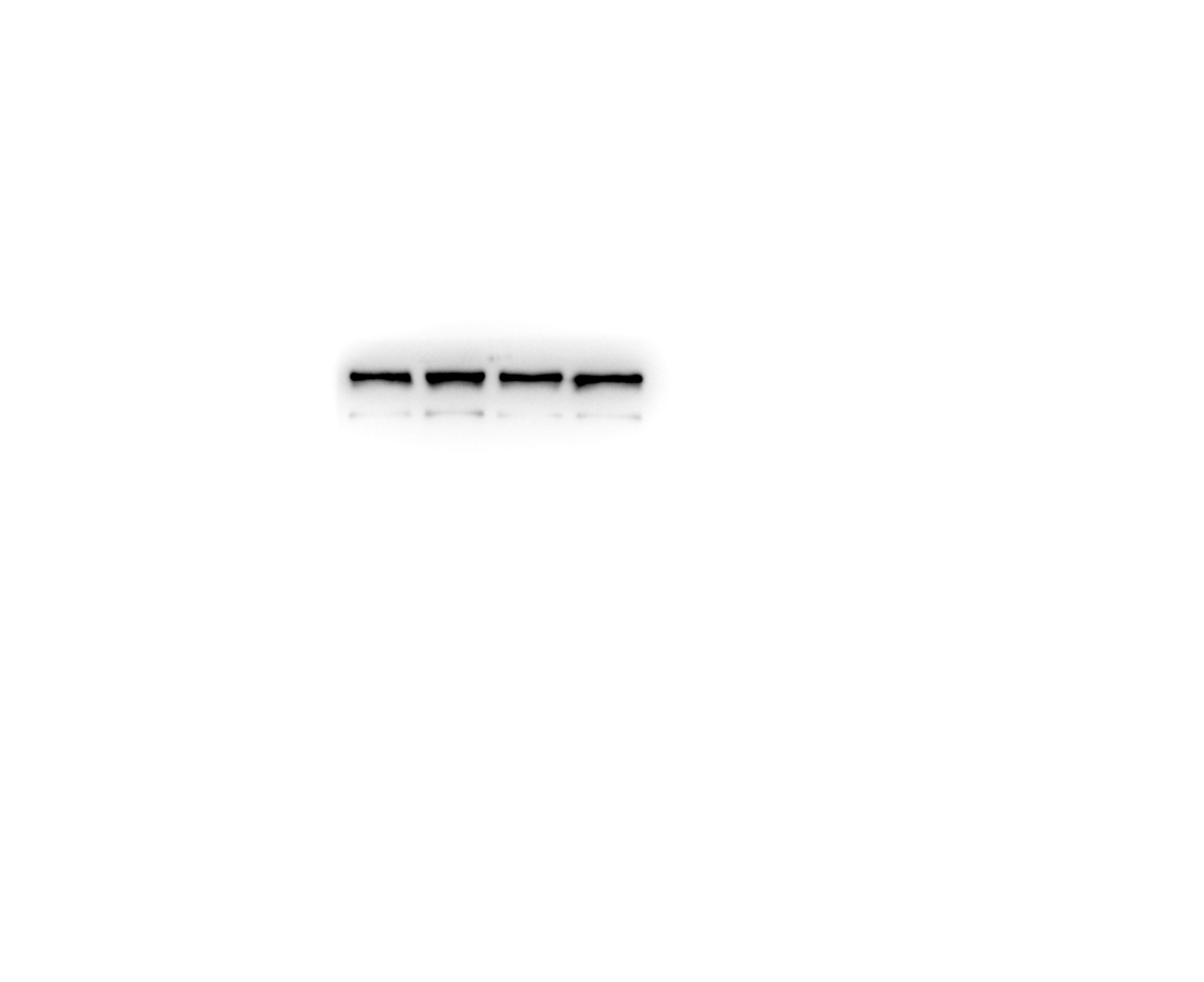

Supplement: Supplemental Information 7 [file peerj-12-17874-s007.zip › fig 4G/p-p65-3 (2).tif]

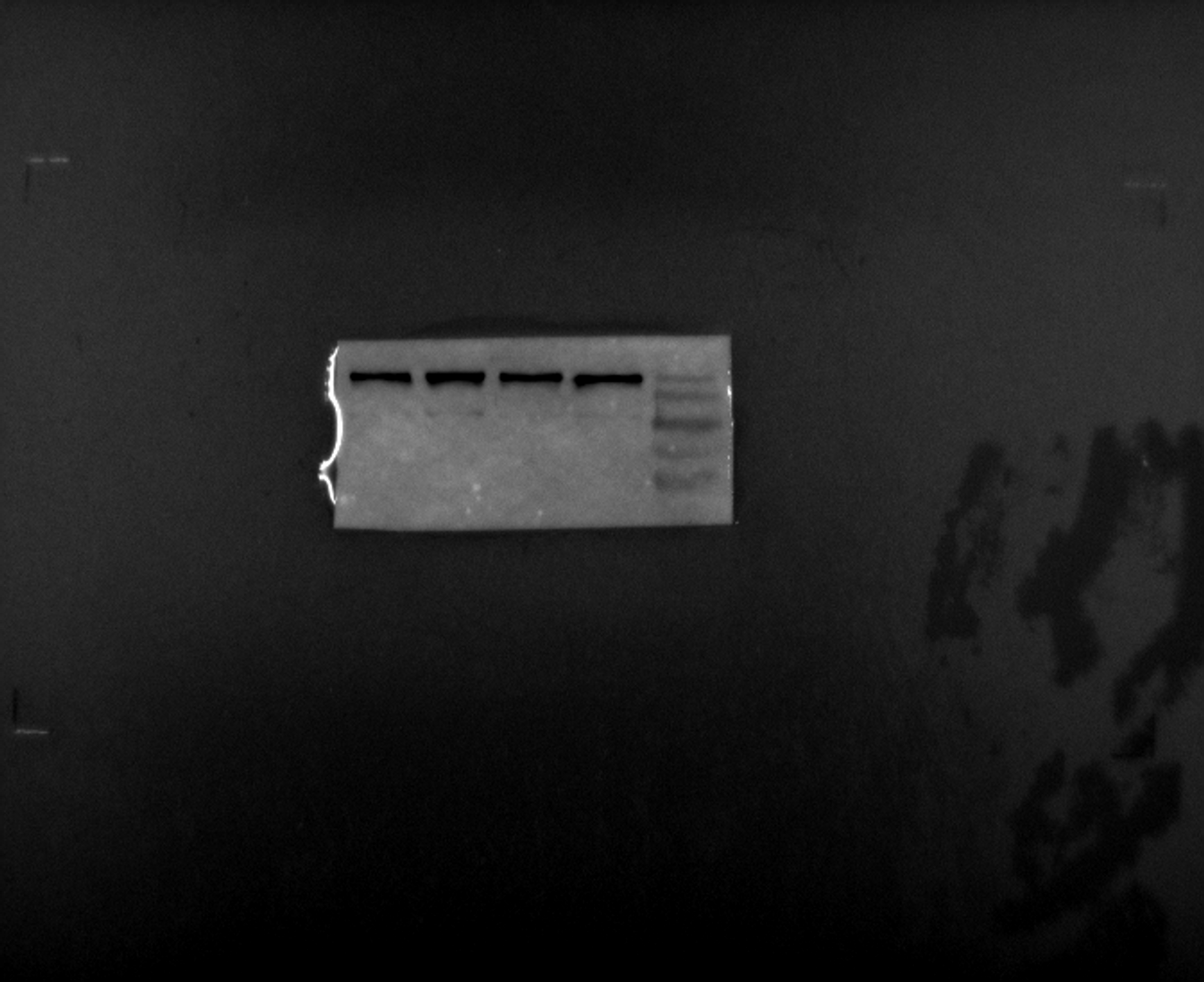

Supplement: Supplemental Information 7 [file peerj-12-17874-s007.zip › fig 4G/p-p65-3 (3).tif]
